# EMILIN-1 Suppresses Cell Proliferation through Altered Cell Cycle Regulation in Head and Neck Squamous Cell Carcinoma

**DOI:** 10.1016/j.ajpath.2025.01.010

**Published:** 2025-01-30

**Authors:** Pichaya Chanpanitkitchote, Jiratchaya Nuanpirom, Warut Pongsapich, Nithi Asavapanumas, Simone Mendler, Nadine Wiesmann, Juergen Brieger, Natini Jinawath

**Affiliations:** ∗ Program in Molecular Medicine, Faculty of Science, Mahidol University, Bangkok, Thailand; † Department of Biochemistry, Faculty of Science, Mahidol University, Bangkok, Thailand; ‡ Integrative Computational BioScience Center (ICBS), Mahidol University, Nakhon Pathom, Thailand; § Department of Otorhinolaryngology, Faculty of Medicine Siriraj Hospital, Mahidol University, Bangkok, Thailand; ¶ Chakri Naruebodindra Medical Institute, Faculty of Medicine Ramathibodi Hospital, Mahidol University, Bangkok, Thailand; ‖ Department of Otorhinolaryngology, University Medical Center Mainz, Mainz, Germany; ∗∗ Department of Oral and Maxillofacial Surgery, University Medical Center Mainz, Mainz, Germany; †† Program in Translational Medicine, Faculty of Medicine Ramathibodi Hospital, Mahidol University, Bangkok, Thailand

## Abstract

Extracellular matrix (ECM) proteins play an important role in the pathological processes of tumor development and progression. Elastic microfibril interface located protein-1 (EMILIN-1), an ECM glycoprotein, is linked to cell adhesion and migration. It was identified from head and neck squamous cell carcinoma (HNSCC) tissues that down-regulated EMILIN-1. It is associated with an increased risk of secondary primary malignancy development in HNSCC and hypothesized to function as a tumor suppressor in HNSCC. This study showed that EMILIN-1 expression in HNSCC tissues was specific to the stromal area, and secreted-EMILIN-1 level was higher in fibroblasts isolated from HNSCC tissues than in HNSCC cells. EMILIN-1 overexpression decreased cell proliferation, migration, and invasion in FaDu and CAL27 cells. Knockdown of EMILIN-1 in HNSCC cancer-associated fibroblasts induced cell proliferation and migration. The conditioned medium from EMILIN-1 knockdown cancer-associated fibroblasts increased HNSCC cell proliferation, and the co-culture system enhanced cancer cell migration and invasion. RNA-sequencing analysis revealed that the cell cycle and aurora kinase signaling were the most significant enrichment pathways, confirmed at the protein level. Furthermore, in an *in ovo* chick chorioallantoic membrane model, overexpression of EMILIN-1 in FaDu cells reduced tumor size and Ki-67–positivity and increased cleaved caspase-3–positive cells. These findings suggest that EMILIN-1 suppresses HNSCC growth partly through the down-regulation of cell cycle and aurora kinase signaling pathways.

## Graphical abstract

**Figure:**
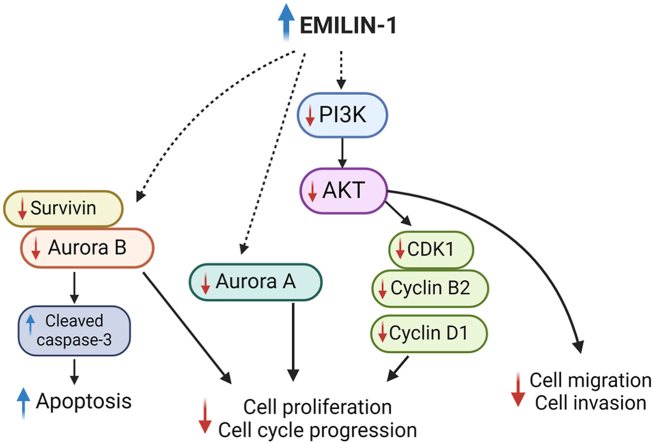

Head and neck squamous cell carcinoma (HNSCC) is the sixth most common leading cancer worldwide, with an incidence of more than 600,000 cases annually.^1^ HNSCC tumors originate from the epithelial cells in the tissue of the mucosal linings of the upper airway and food passages, including the oral cavity, tongue, pharynx, larynx, nasal cavity, and salivary glands.^2^ Consumption of alcohol, long-term tobacco use, and human papillomavirus (HPV) infection are considered major risk factors for HNSCC. Approximately 75% of HNSCC are associated with tobacco and alcohol consumption,^3^ whereas 15% to 20% are related to high-risk HPV infection.^4^ Standard treatment include surgery, radiotherapy, chemotherapy, or combination treatment.^5^ However, 40% to 60% of treated patients experience disease recurrence and treatment resistance,^6^ leading to a 5-year survival rate of just 40% to 50%.^5^ This highlights the need for a better understanding of HNSCC molecular biology to improve prognosis, diagnostics, and clinical therapeutic targets.

Some HNSCC patients are prone to developing a second primary malignancy (SPM), defined as a tumor occurring simultaneously with or after the initial diagnosis. Up to 24% of HNSCC patients develop SPM,^7^ primarily in those with prolonged exposure to carcinogens such as continuous smoking.^8^ SPM significantly impacts overall survival, ranking as the second leading cause of death in HNSCC patients.^7^ In a recent study by Bunbanjerdsuk et al^9^ (2019), proteomics and gene expression analysis of HNSCC patients identified down-regulated extracellular matrix proteins, including elastic microfibril interface located protein-1 (EMILIN-1), as potential biomarkers for SPM development.

EMILIN-1 is a glycoprotein found in the extracellular matrix that is associated with elastic fibers. EMILIN-1 can be characterized by the C-terminal end of the homologous globular domain of C1q (gC1q domain) region.^10^ Homotrimeric EMILIN-1 assembles into high molecular weight multimers via the gC1q domain.^11^ EMILIN-1 is typically found in the skin, blood vessel walls,^12^ intestines, lungs, lymph nodes, and lymphatic vessels.^13^

An association between EMILIN-1 and cancer suggests that this ECM glycoprotein could play a tumor-suppressive role in the tumor microenvironment (TME) by regulating cell growth and cell migration.^14^ The gC1q domain is involved in cell adhesion and migration via interaction with the α4β1 integrin^15^ and the closely related α9β1 integrin.^16^^,^^17^ EMILIN-1 suppresses the RAS-ERK signaling pathway through α4β1 integrin, leading to decreased cell proliferation and tumor cell growth in colon cancer.^18^ The absence of EMILIN-1 induces cell proliferation and tumor growth in skin tumors.^19^ Degradation and consequent loss of EMILIN-1/gC1q in the tumor environment promotes tumor initiation and progression in colon cancer.^20^ These findings show an interesting role played by EMILIN-1 in the TME in various cancers.

In this study, EMILIN-1 was overexpressed in HNSCC cells and knockdown EMILIN-1 was overexpressed in primary fibroblast cells to assess their effect on cell proliferation, migration, and invasion through transcriptomic analysis. The effect of EMILIN-1 on proliferation was also assessed *in ovo*. The results showed that EMILIN-1 suppressed cell proliferation by down-regulating cell cycle pathway protein.

## Materials and Methods

### Data Mining

*EMILIN1* expression data from the Cancer Genome Atlas Program (TCGA) database were obtained and analyzed via the TIMER2.0 (*http://timer.cistrome.org*, last accessed December 13, 2024) UCSC Xena analysis (*https://xena.ucsc.edu*, last accessed December 13, 2024) was used to analyze the gene expression of tumor subgroups and the Kaplan-Meier overall survival curve in the Genomic Data Commons (GDC) TCGA-HNSC dataset. The Kaplan-Meier plot was analyzed by dividing the samples into median and upper-lower quartiles. The Human Protein Atlas (HPA; *https://www.proteinatlas.org*, last accessed December 13, 2024) was used to obtain EMILIN1 expression data at a single-cell level as well as information on cancer cell lines, and HNSCC tissue from immunohistochemistry staining.

### Primary Fibroblast Isolation from Head and Neck Cancer Tissues

Human HNSCC tissue samples and their matching normal tissues were obtained from surgical resection at the Department of Otorhinolaryngology, Faculty of Medicine Siriraj Hospital, Mahidol University. Tissue samples from patients were processed under the ethical guidelines of Ramathibodi (protocol ID number: MURA2019/154) and Siriraj Hospital's Ethic Committees (protocol ID number: 234/2562). Primary human cancer associated fibroblasts (CAFs) and normal fibroblasts (NFs) were isolated from the HNSCC tissues and normal tissues of patients. CAF2 cells were isolated from hypopharynx carcinoma tissues, whereas CAF3 cells were isolated from oral carcinoma tissues. The tissues were washed five times with Dulbecco's modified eagle's medium (DMEM) with antibiotics (200 U/mL penicillin-streptomycin; Gibco, Thermo Fisher Scientific, Waltham, MA; 100 μg/mL Normocin; InvivoGen, San Diego, CA). The tissues were cut into small pieces and transferred into the gentleMACS C Tube containing an enzyme mix from the Tumor Dissociation Kit, human (130-095-929; Miltenyi Biotec, Bergisch Gladbach, Germany). The samples were incubated for 1 hour at 37°C under the 37C_h_TDK_3 program on the gentleMACS dissociator machine. The mixture was filtered through a 40-μm strainer, then centrifuged at 200 × *g* for 5 minutes and washed twice with DMEM. The cells were plated on a 6-well plate in 2 mL of DMEM supplemented with 10% fetal bovine serum (ES-009-B; Merck Millipore, Burlington, MA). The primary fibroblast cultures were incubated at 37°C. The media was initially replaced after 24 hours, then every third day thereafter. Immunofluorescence of CAF markers [α-smooth muscle actin (α-SMA) and fibroblast activation protein alpha (FAP-α)] was used for CAF characterization. All primary fibroblasts used in this study were under passage 10. All fibroblast cell–related assays were conducted in a minimum of three biological replicates.

### Cell Lines and Cell Culture

The human HNSCC cell lines SCC25, CAL27, and FaDu were purchased from ATCC (Manassas, VA). FaDu was cultured in Eagle's Minimum Essential Medium (30-2003; ATCC), whereas CAL27 was cultured in DMEM (30243.02; Cytiva, Marlborough, MA). SCC25 was cultured in DMEM/F12 (30023.02; Cytiva) and supplemented with 800 ng/mL hydrocortisone (H0888; Sigma-Aldrich, St. Louis, MO). All the cell lines were cultured in medium supplemented with 10% fetal bovine serum and 1% antibiotic-antimycotic (15240-062; Gibco, Thermo Fisher Scientific). Cell cultures were maintained at 37°C in a 5% humidified CO_2_ incubator. Before use, cell lines were verified by MycoAlert PLUS Mycoplasma Detection Kit (LT07-710; Lonza, Basel, Switzerland) to be free of mycoplasma. All assays related to cell lines were conducted in a minimum of three biological replicates. HNSCC cell lines used in experiments were maintained at no more than 15 passages from the original passage received from ATCC.

### Condition Medium and Secreted Protein Collection

Cells were washed twice with 1× phosphate-buffered saline (PBS) and incubated in serum-free medium at 37°C for 24 hours. Following incubation, the conditioned medium (CM) was carefully removed, and centrifuged at 4,000 × g for 5 minutes to eliminate suspended cells. The suspension of CM was concentrated by centrifugation at 4,000 × g for 15 minutes using 50k Amicon Ultra-15 Centrifugal Filter Units (UFC905008; Merck Millipore). The CM was aliquoted and stored at −80°C until used. The protein concentration of the CM was measured using a bicinchoninic acid protein assay kit (23225; Thermo Fisher Scientific).

### Real-Time PCR Analysis

RNA was extracted from whole-cell lysates using the RNeasy MiniKit (74104; Qiagen, Hilden, Germany). One μg of RNA was converted into cDNA using an iScript cDNA Synthesis Kit (1708891; Bio-Rad Laboratories, Hercules, CA). Real-time PCR was performed using a KAPA SYBR FAST Universal 2X qPCR Master Mix Kit (K4600; Sigma-Aldrich), per the instructions for specific primers (Table 1). Relative gene expression levels were measured using the ΔΔC_T_ calculation method.

**Table 1:** Primer Sequences

Primer	Forward	Reverse
EMILIN1	5′-CTCAAGGTGAACAGGGAGTGG-3′	5′-CACGCCTGTCTCTGGATCAT-3′
β-Actin	5′-GGCACCCAGCACAATGAAGATC-3′	5′-GTAACGCAACTAAGTCATAGTCCGC-3′

### Plasmids and Lentiviral Transduction

*EMILIN1* plasmid was purchased from GenScript (pcDNA3.1; Piscataway, NJ). The lentiviral plasmid backbone pLenti-CMV-MCS-GFP-SV-puro (#73582) was obtained from Addgene (Watertown, MA). To obtain an *EMILIN1* lentiviral expression vector, the cloning and primers designed for insert fragments with overlapping regions were performed using the Gibson DNA assembly method. Each fragment was combined using NEBuilder HiFi DNA Assembly (E5520; New England Biolabs, Ipswich, MA). Plasmid DNA was then transformed into NEB Stable Competent cells (C3040; New England Biolabs). Colony PCR was performed to confirm positive clones with an *EMILIN1* insert. All sequences were verified by DNA sequencing.

*EMILIN1* shRNA lentiviral vector pZIP-hCMV-ZsGreen-Puro and the control vector were purchased from transOMIC technologies (Huntsville, AL). The control vector expresses a non-targeting shRNA (scrambled shRNA) for human cells. The sequence of the shRNA used is listed in Table 2. Third-generation lentiviral vectors were used to transduce the virus into cells using Lipofectamine3000 Transfection Reagent (L3000008, Invitrogen; Thermo Fisher Scientific), followed by selection with 2 μg/mL puromycin antibiotic.

**Table 2:** shRNA Sequences

shRNA	Sense	Antisense
shControl	5′-AAGGCAGAAGTATGCAAAGCAT-3′	5′-TACGAAACGTATGAAGACGGAC-3′
shEMILIN1-14	5′-ACTCCCTCAATGACTCACTGAA-3′	5′-AAGTCACTCAGTAACTCCCTCG-3′
shEMILIN1-16	5′-AGCGGTTGGATCTGTTGGAGGA-3′	5′-AGGAGGTTGTCTAGGTTGGCGG-3′

### Cell Proliferation Assay

Cell viability was measured using CellTiter-Glo Luminescent Cell Viability Assay (G7570; Promega Corporation, Madison, WI). Cell proliferation was calculated as the relative percentage from day 0.

### Colony Formation Assay

Cells were seeded at 250 cells/well in a 6-well plate with complete medium. After culturing for 10 days, colonies were washed twice with PBS, fixed with 100% methanol for 30 minutes, and then stained with 0.2% crystal violet for 20 minutes.

### Wound Healing Assay

Cells were seeded at 3 × 10^4^ cells/well in a Culture-Insert 2 well (81176; ibidi, Gräfelfing, Germany). After the culture insert was removed, DMEM with 1% fetal bovine serum was added. Representative images were captured at 0 and 24 hours after the wound was created. The area of wound closure was analyzed using ImageJ software version 1.52 (*https://imagej.net*).

### Transwell Migration and Invasion Assay

Cells in serum-free medium were seeded 2 × 10^5^ cells/well in the upper chamber of a 24-well plate with 8.0-μm pore membranes (3422; Corning, Corning, NY). Complete medium with 10% fetal bovine serum was added to the lower chamber as the chemoattractant. After incubation at 37°C for 24 hours, nonmigrating cells on the upper surface of the membrane were removed with cotton swabs. Migrated cells were fixed with 100% methanol and stained with 0.2% crystal violet. Cells were captured under a microscope and analyzed using ImageJ software. Transwell invasion assays were performed as described above, except that cells were seeded in the upper chamber with 0.1 mg/mL Matrigel (356234; Corning).

For the insert co-culture system, EMILIN-1 knockdown CAF cells were seeded at 2 × 10⁵ cells/well in the bottom chamber of a 24-well plate, and cancer cells were added to the upper chamber. After incubation at 37°C for 48 hours, the migrated and invaded cancer cells were stained following the same protocol.

### Three-dimensional Tumor Spheroid Invasion Assay

Cells were cultured at a density of 10,000 cells/well in ULA 96-well round-bottom plates. After 3 days of tumor spheroid formation, 100 μL of Matrigel (354234; Corning) was added to induce invasion. Captured images were used to measure the area covered by the spheroids with ImageJ software. The change in spheroid area at each time point was calculated and plotted as a relative invasion to day 0.

### Cell Cycle Analysis

Cells were harvested, washed twice with ice-cold PBS, and subsequently fixed with 70% ethanol at −20°C for at least 2 hours. After fixation, the cells were resuspended, washed twice with ice-cold PBS, and then suspended in 500 μL of PBS containing 50 μg/mL propidium iodide (PI) and 100 μg/mL RNase. The cell suspension was incubated in the dark at 4°C for 30 minutes. Stained cells were analyzed with a FACS Canto flow cytometry (BD Biosciences, Franklin Lakes, NJ) using FlowJo software version 10 (FlowJo, Ashland, OR). The percentage of cells in each phase of cell cycle from flow cytometry was plotted in a bar graph.

### Apoptosis Assay

Apoptotic cells were determined using annexin V/PI staining. Cells were harvested and washed with 1X Annexin V Binding Buffer (V13246, Invitrogen). The cells were resuspended in 100 μL of 1X Annexin V Binding Buffer, then 5 μL of Annexin V-APC (A35110, Invitrogen), 5 μL of PBS containing 50 μg/mL PI, and 100 μg/mL RNase were added. The cell suspension was incubated on ice in the dark for 30 minutes and analyzed by FACS Canto flow cytometry using FlowJo software. The analysis of stained cells divided them into two groups: Q3 early apoptosis (annexin V^+^PI^−^) and Q2 late apoptosis (annexin V^+^PI^+^). The percentages of Q2 and Q3 populations from flow cytometry data were represented as a bar graph.

### Immunofluorescence Staining

Cells were cultured in 8-well Leb-Tek II Chamber Slides (154534; Thermo Fisher Scientific) and fixed with 4% paraformaldehyde for 15 minutes at room temperature, permeabilized with 0.1% TritonX-100/PBS, blocked with 5% Normal Goat Serum blocking solution (S1000; Vector Laboratories, Newark, CA) for 1 hour at room temperature, and then incubated with primary antibodies at 4°C overnight. The cells were washed and incubated with appropriate secondary antibodies in the dark for 1 hour at room temperature. After additional washing steps, the cells were mounted with VECTASHIELD Antifade Mounting Medium with DAPI (H1200; Vector Laboratories). Stained cells were visualized and pictured under a laser scanning confocal microscope (Zeiss LSM 900 with Airyscan; Zeiss, Oberkochen, Germany).

### Western Blot Analysis

**Supplemental Figure S1:**
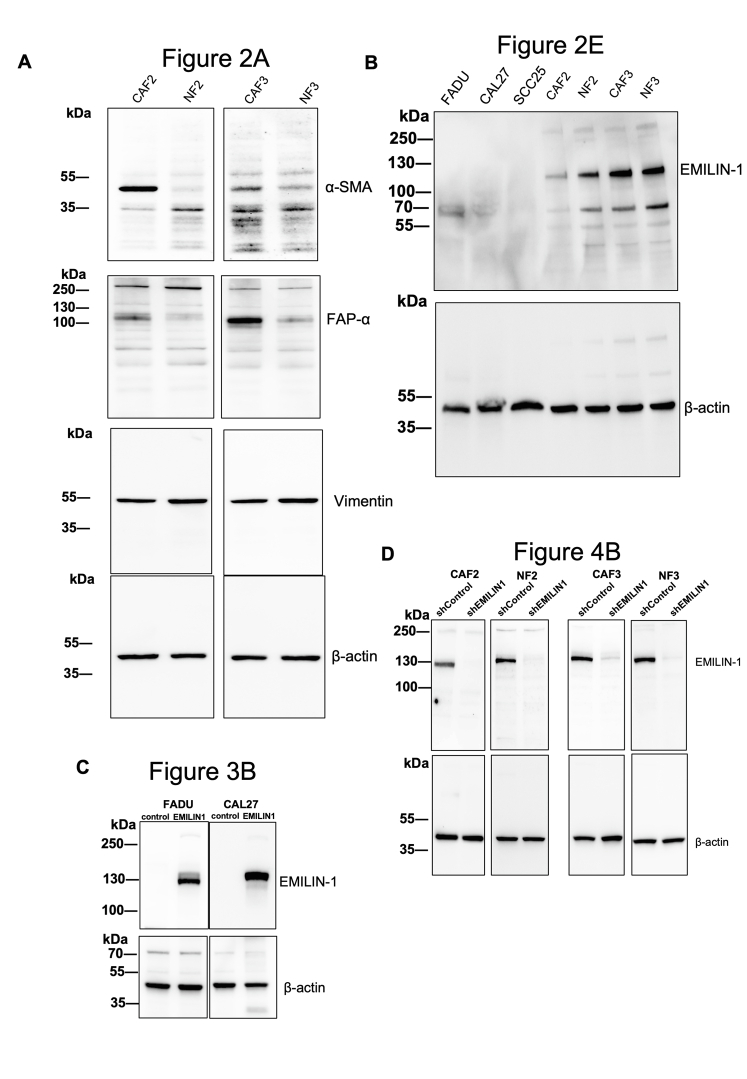
Full membrane images for Western blots corresponding to Figure 2A (**A**), Figure 2E (**B**), Figure 3B (**C**), and Figure 4B (**D**).

**Supplemental Figure S2:**
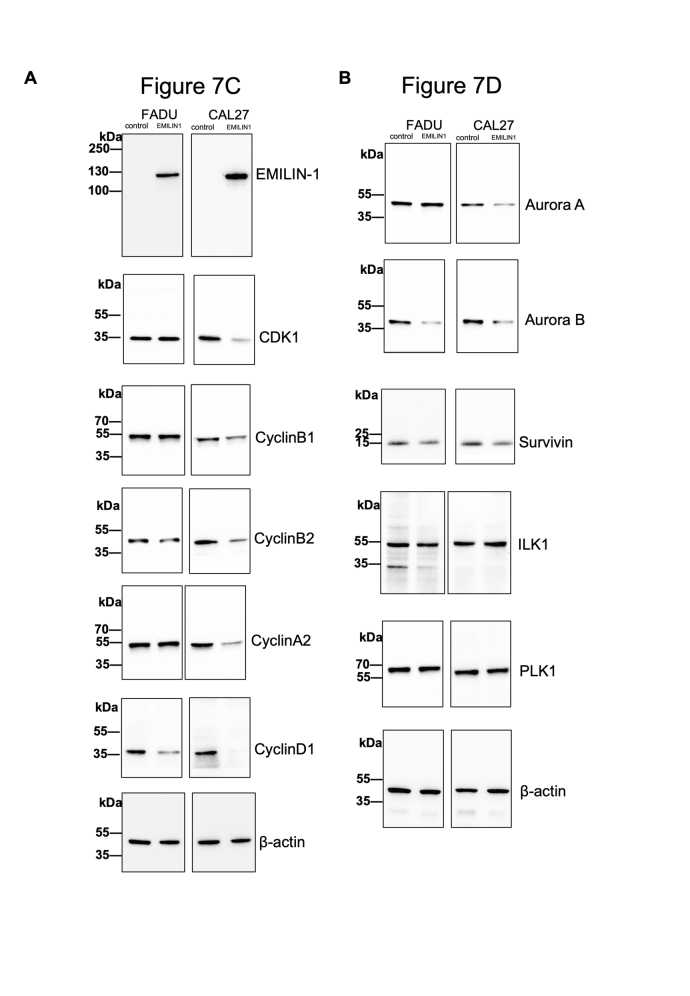
Full membrane images for Western blots corresponding to Figure 7C (**A**) and Figure 7D (**B**).

**Supplemental Figure S3:**
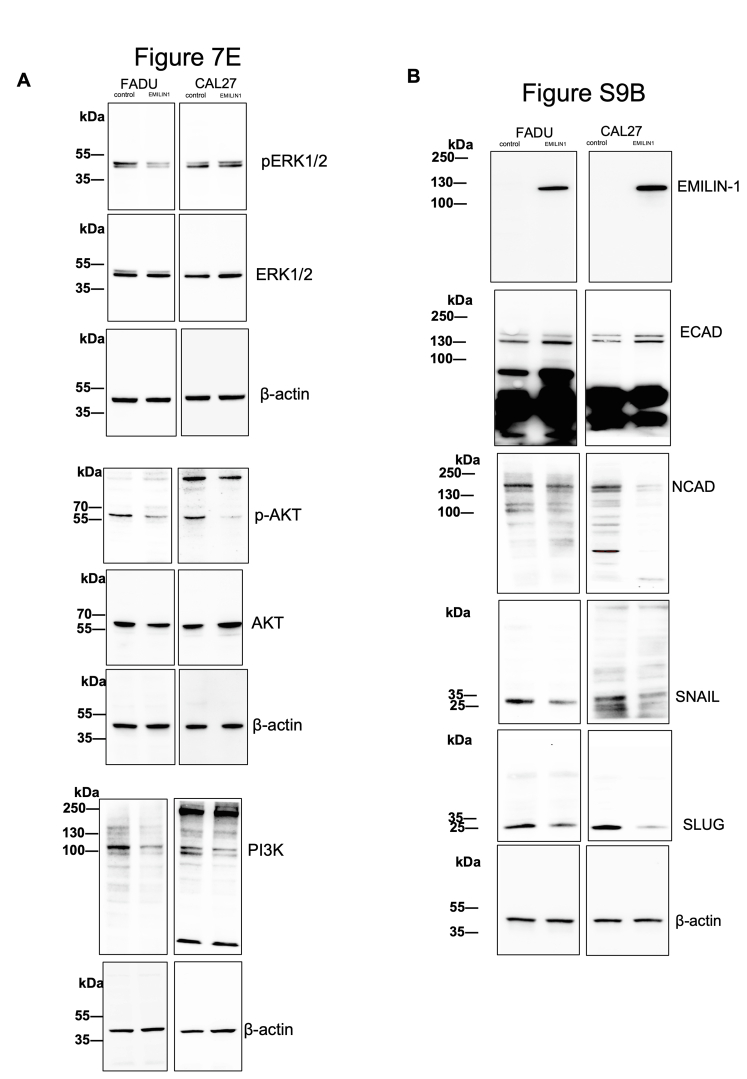
Full membrane images for Western blots corresponding to Figure 7E (**A**) and Supplemental Figure S9B (**B**).

**Supplemental Figure S4:**
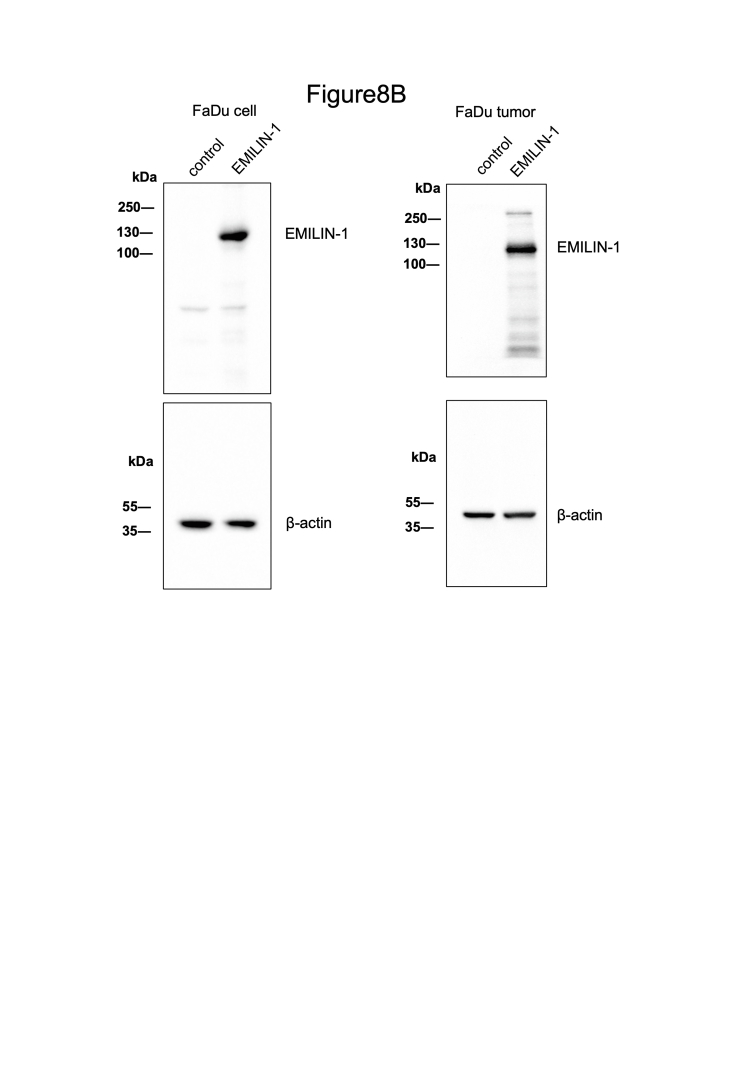
Full membrane images for Western blots corresponding to Figure 8B.

Cells were lysed with radioimmunoprecipitation assay lysis buffer containing Halt Protease and Phosphatase Inhibitor Cocktail (78440; Thermo Fisher Scientific). Twenty μg of protein were resolved by SDS-PAGE and transferred to a polyvinylidene difluoride membrane (1704274, Trans-blot Turbo RTA Transfer kit; Bio-Rad Laboratories). After blocking with 10% Blotting-Grade Blocker nonfat dry milk (1706404; Bio-Rad Laboratories) for 1 hour at room temperature, membranes were probed with the primary antibody at 4°C overnight and incubated with appropriate horseradish peroxidase–conjugated secondary antibodies for 1 hour at room temperature. β-Actin antibody was used as a loading control. Membranes were detected with Clarity max Western ECL substrate (1705062; Bio-Rad Laboratories) and visualized using the ChemiDoc Imaging system (Bio-Rad Laboratories). All antibodies used in this study are listed in Table 3. The full images for each membrane are provided in Supplemental Figures S1–S4.

**Table 3:** Antibodies Used in This Study

Target antigen	Vender	Catalog no.	Dilution
EMILIN1	Sigma-Aldrich	HPA002822	WB 1:5000 IF 1:200 IHC 1:200
β-Actin-HRP conjugated	Cell Signaling Technology	12262	WB 1:10,000
α-SMA	Thermo Fisher Scientific	MA5-11544	WB 1:500
α-SMA Alexa Fluor 555	Abcam	Ab202509	IF 1:200
α-SMA	Sigma-Aldrich	A2547	IHC 1:1500
Vimentin	Thermo Fisher Scientific	MA5-11880	WB 1:1000
Vimentin	Cell Signaling Technology	5741	IF 1:200
ITGB1	Abcam	30394	IF 1:200
Cleaved Caspase 3	Cell Signaling Technology	9661	IHC 1:50
Ki-67	Agilent	M7240	IHC 1:150
Pan-cytokeratin	Agilent	M0821	IHC 1:100
FAP-α	Abclonal	A6349	WB 1:500 IF 1:200
E-Cadherin	Cell Signaling Technology	3195	WB 1:5000
N-Cadherin	Cell Signaling Technology	13116	WB 1:1000
Snail	Cell Signaling Technology	3879	WB 1:1000
Slug	Cell Signaling Technology	9585	WB 1:1000
pErk1/2 (Thr202/Tyr204)	Cell Signaling Technology	4370	WB 1:1000
Erk1/2	Cell Signaling Technology	4695	WB 1:1000
pAkt (Ser473)	Cell Signaling Technology	4060	WB 1:1000
Akt	Cell Signaling Technology	9272	WB 1:1000
PI3K (p110α)	Cell Signaling Technology	4249	WB 1:1000
CDK1	Abcam	ab18	WB 1:1000
Cyclin B1	Cell Signaling Technology	12231	WB 1:1000
Cyclin B2	Abcam	ab185622	WB 1:1000
Cyclin A2	Cell Signaling Technology	67955	WB 1:1000
Cyclin D1	Cell Signaling Technology	55506	WB 1:1000
Aurora A	Cell Signaling Technology	14475	WB 1:1000
Aurora B	Cell Signaling Technology	3094	WB 1:1000
Survivin	Cell Signaling Technology	2808	WB 1:1000
ILK1	Cell Signaling Technology	3862	WB 1:1000
PLK1	Cell Signaling Technology	4513	WB 1:1000
Goat anti-rabbit IgG (H + L) secondary antibody, HRP	Thermo Fisher Scientific	31460	WB 1:10,000
Goat anti-mouse IgG (H + L) secondary antibody, HRP	Thermo Fisher Scientific	31430	WB 1:10,000
Goat anti-rabbit IgG (H + L) secondary antibody, DyLight 488	Thermo Fisher Scientific	35552	IF 1:500
Goat anti-mouse IgG (H + L) secondary antibody, DyLight 488	Thermo Fisher Scientific	35502	IF 1:500
Goat anti-mouse IgG (H + L) secondary antibody, DyLight 594	Thermo Fisher Scientific	35510	IF 1:500

α-SMA, α-smooth muscle actin; CDK1, cyclin-dependent kinase 1; FAP-α, fibroblast activation protein alpha; HRP, horseradish peroxidase; IF, immunofluorescence; IHC, immunohistochemistry; ILK1, integrin-linked kinase1; ITGB1, integrin beta-1; pAkt, phosphorylated AKT; pERK1/2, phosphorylated ERK1/2; PI3K, phosphatidylinositol 3-kinase; PLK1, polo-like kinase 1; WB, Western blot.

### RNA Sequencing and Bioinformatics Analysis

Total RNA was extracted from cells using the RNeasy Minikit (Qiagen). The quantity and quality of RNA were assessed using Nanodrop2000 Spectrophotometer (Thermo Fisher Scientific) and 2100 Bioanalyzer (Agilent, Santa Clara, CA). RNA sequencing was performed by Macrogen Korea on the Illumina sequencing platform with TruSeq Standard mRNA Library Prep Kit (Illumina, San Diego, CA). Paired-end sequenced reads were analyzed using CLC Genomics Workbench software version 21.0 (Qiagen), and fast-q files were quality control checked and mapped to the reference sequence (Homo sapiens GRCh38). Differential expression analysis compared the control and knockdown/overexpressed conditions in each cell. Differentially expressed genes (DEGs) were evaluated with a cutoff of the false discovery rate–adjusted *P* value <0.05 and |Log2Fold change| > 1. Gene Set Enrichment Analysis (GSEA) database (*https://www.gsea-msigdb.org*, last accessed December 13, 2024) and Cytoscape software version 3.9.0 (*https://cytoscape.org*) were utilized for gene set and pathway enrichment analysis.

### CAM Assay

Chick chorioallantoic membrane (CAM) assay was performed as described previously.^21^ Briefly, fertilized chicken eggs (Lohmann Deutschland GmbH & Co. KG, Neuwied, Germany) were incubated at 37.5°C. The CAM was exposed by creating a small window on top of the egg. Approximately 5 × 10^6^ FaDu cells were prepared in three-dimensional culture with Matrigel (356237; Corning) and placed on the upper CAM of 7-day-old chick embryos. The opening windows were sealed with paraffin tape. On the 14th day of the experiment, the tumor and surrounding CAM were harvested, fixed with 4% formaldehyde, and then embedded in paraffin.

### Immunohistochemistry Analysis

Three-μm thick tumor sections were stained with hematoxylin and eosin to confirm tumor growth in CAM. Subsequently, unstained sections were stained with EMILIN-1, cytokeratin (PanCK), Ki-67, cleaved caspase-3 (CC3) and α-SMA. DAB Chromogen Kit (K3468; Agilent) was used to visualize positive stains. Quantification of Ki-67 and CC3 expression was determined by counting positive nuclei against negative nuclei in at least three independent image fields and calculated as a percentage of positive cells. The tumor size was assessed by calculating the area using ImageJ software. The PanCK positive area was determined using the threshold method in ImageJ. The stromal area was calculated by subtracting the PanCK positive area from the overall tumor area, representing stromal cells in the surrounding tissue section.

### Data Analysis and Statistics

The experimental assays were performed independently in triplicate, with the specific number of samples indicated in the corresponding figure legends. Statistical analyses were done using GraphPad Prism software version 8.0.2 (GraphPad Software, Boston, MA). The following tests were applied: two-tailed unpaired *t*-tests for comparison between two groups; one-way analysis of variance with Tukey's multiple comparison test for comparisons involving more than two groups; two-way analysis of variance with Sidak's multiple comparisons test when comparing multiple groups with two variables. *P* < 0.05 was considered statistically significant.

### Data Availability

The RNA-seq data files referred to in this publication have been deposited in the NCBI's Gene Expression Omnibus (*https://www.ncbi.nlm.nih.gov/geo*; accession number GSE253677).

## Results

### Expression of EMILIN-1 in HNSCC Tissues and Cell Lines

**Figure 1:**
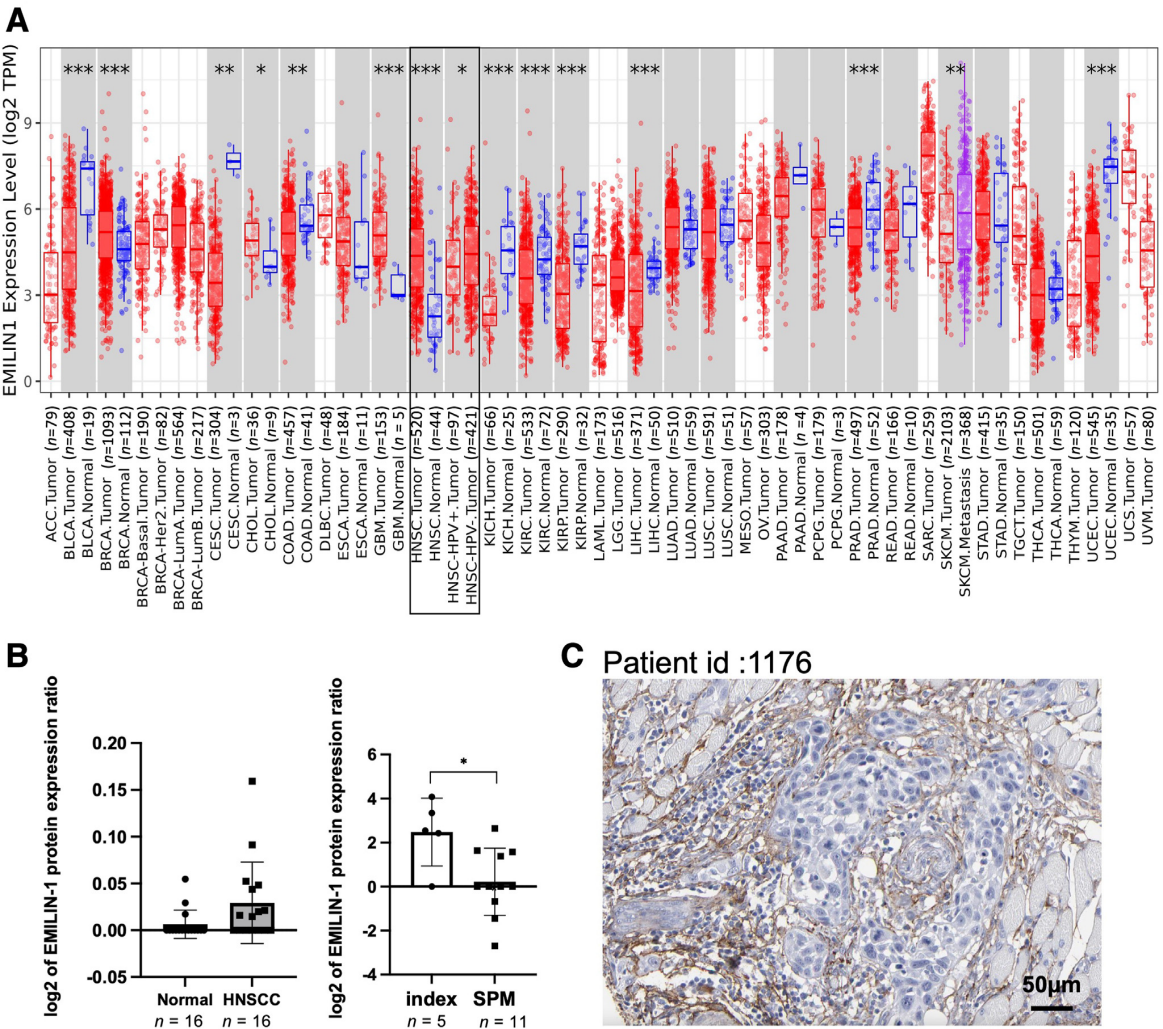
EMILIN-1 expression in cancer tissue. **A:***EMILIN1* expression in various cancer tissues compared with normal tissue from the TCGA dataset. **B:** The **black box** highlights data from head and neck squamous cell (HNSC) tumors. EMILIN-1 protein expression between paired HNSCC and normal tissue (left), EMILIN-1 protein expression between HNSCC tissue with second primary malignancy (SPM) development and without SPM (right). **C:** Immunohistochemical staining of EMILIN-1 in HNSCC tissue sample from the Human Protein Atlas database. *n* = 16 HNSCC and normal tissue (**B**); *n* = 11 SPM (**B**); *n* = 5 index (**B**). ∗*P* < 0.05, ∗∗*P* < 0.01, and ∗∗∗*P* < 0.001. Scale bar = 50 μm (**C**). TPM, transcripts per million.

**Supplemental Figure S5:**
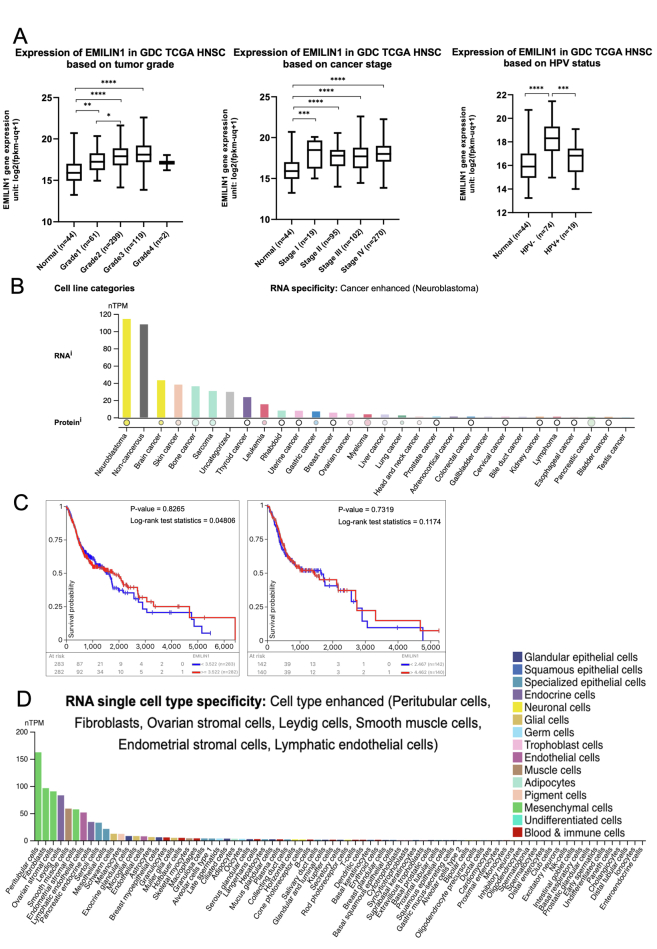
Data mining of *EMILIN1* in an open-source database. **A:** Comparison of *EMILIN1* expression based on tumor grade, cancer stage, and HPV status from the UCSC Xena-GDC TCGA-HNSC dataset. **B:** EMILIN1 expression in various cancer cell lines from Human Protein Atlas database. **C:** Overall survival plot from UCSC Xena-GDC TCGA-HNSC dataset. Kaplan-Meier curve with median (left) and quartiles (right) as cutoff. **D:** Single-cell RNA-seq analysis from the Human Protein Atlas database showing the various *EMILIN1* expression levels in specific cell types. ∗*P* < 0.05, ∗∗*P* < 0.01, ∗∗∗*P* < 0.001, and ∗∗∗∗*P* < 0.0001.

The TCGA database analysis showed higher expression of *EMILIN1* in 520 HNSCC tumor tissues compared to 44 normal epithelial tissues. *EMILIN1* levels were higher in HPV-negative HNSCC tumor tissues than in HPV-positive ones (Figure 1A). They significantly increased from Grade 1 to Grade 2 (Supplemental Figure S5A), with no differences among other grades or cancer stages. *EMILIN1* expression was generally low across cancer cell lines, except in neuroblastoma (Supplemental Figure S5B), and no significant correlation with HNSCC patient survival was observed (Supplemental Figure S5C).

As in a previous study,^9^ EMILIN-1 protein levels appeared to be higher in HNSCC than in normal epithelial tissues, although this difference was not statistically significant (Figure 1B). Primary HNSCC tissues from patients with SPM exhibited significantly lower EMILIN-1 expression than those from patients without SPM (index) (Figure 1B). Immunohistochemistry analysis from the Human Protein Atlas indicated that EMILIN-1 was not detected in HNSCC cells but was present in the extracellular matrix (Figure 1C). Furthermore, single-cell RNA-seq analysis from the Human Protein Atlas revealed that increased *EMILIN1* expression was specific to mesenchymal cell types, particularly in peritubular cells, fibroblasts, ovarian stromal cells, endometrial stromal cells, and smooth muscle cells (Supplemental Figure S5D).

**Figure 2:**
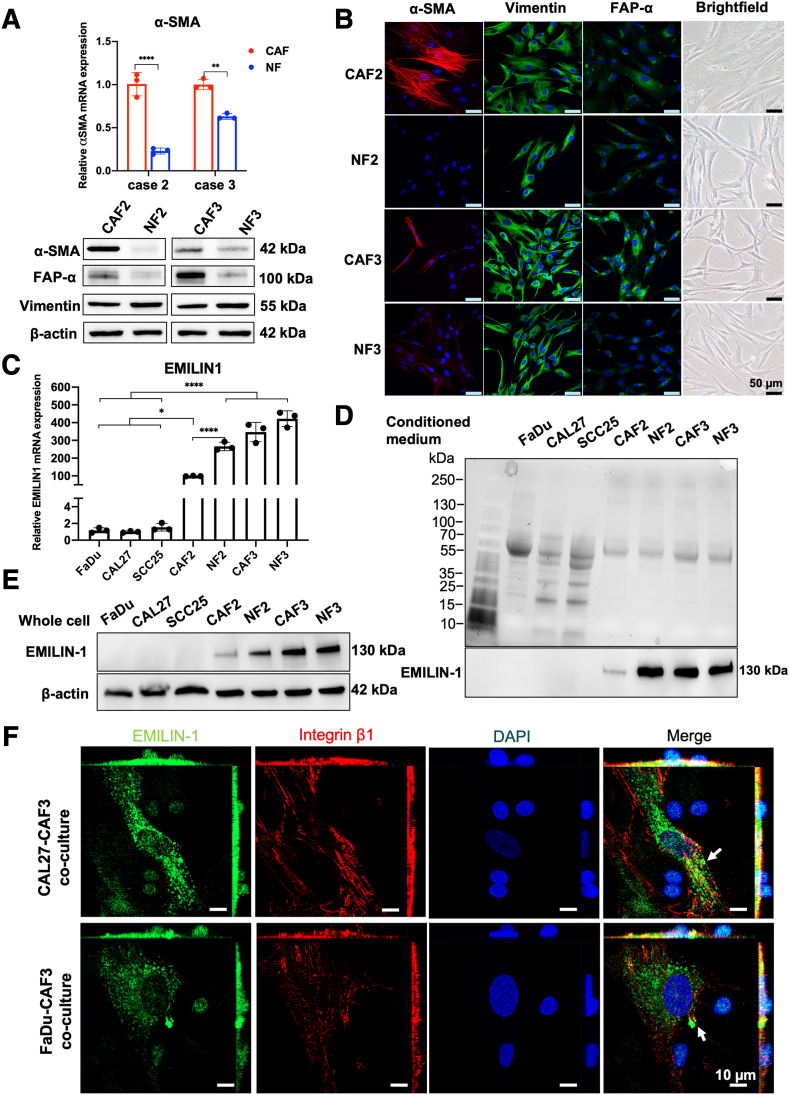
Primary fibroblast characterization and EMILIN-1 expression. **A:** mRNA level of α-SMA in cancer associated fibroblasts (CAFs) and normal fibroblasts (NFs) by quantitative RT-PCR (top). Protein expression of α-SMA, FAP-α, and vimentin in CAFs and NFs by immunoblotting using β-actin as an internal control (bottom). **B:** Representative immunofluorescence staining images of CAFs and NFs showing the expression of α-SMA, FAP-α, and vimentin. **C:** EMILIN1 mRNA expression in head and neck squamous cell carcinoma (HNSCC) cell line (FaDu, CAL27, SCC25) and primary fibroblast cells. **D** and **E:** EMILIN-1 protein expression in CM (**D**) and whole-cell lysate (**E**) collected from HNSCC cell line and primary fibroblast cells. **F:** Representative immunofluorescence staining images of CAF3 co-cultured with cancer cells. **White arrows** indicating EMILIN-1–expressing vesicle-like structures in CAFs. Data are expressed as means ± SD. *n* = 3. ∗*P* < 0.05 ∗∗*P* < 0.01, and ∗∗∗∗*P* < 0.0001. Scale bars: 50 μm (**B**); 10 μm (**F**).

Primary CAFs isolated from HNSCC tissues and NFs from matched normal epithelial tissues were used for further analyses. The expression of fibroblast markers was validated by quantitative RT-PCR and Western blot, showing a significant increase in both mRNA and protein levels of α-SMA in CAFs compared with NFs (*P* < 0.01) (Figures 2A). Immunofluorescence staining further confirmed the protein levels of these markers (Figure 2B). Both CAFs and NFs similarly expressed vimentin, a specific marker of stromal cells, whereas the expression of α-SMA and FAP-α in CAFs was higher than that in NFs.

Next, quantitative RT-PCR and Western blot analyses were performed to assess EMILIN-1 expression in HNSCC cell lines (FaDu, CAL27, SCC25), CAFs, and NFs isolated from HNSCC patients. EMILIN1 mRNA expression levels were significantly higher in fibroblast cells than in HNSCC cells (*P* < 0.0001) (Figure 2C). The expression of secreted EMILIN-1 in CM and whole-cell lysate was prominently observed in all fibroblast cells, whereas it was undetected in all HNSCC cell lines (Figure 2, D and E). Immunofluorescence staining revealed high expression of EMILIN-1 in the cytoplasm of CAFs. EMILIN-1 co-localized with integrin β1 at the cell membrane and in vesicle-like structures, indicating a possible ligand-receptor interaction (Figure 2F). Altogether, these findings confirm that EMILIN-1 is primarily produced by fibroblast cells and exhibits lower expression in HNSCC cell lines.

### EMILIN-1 Overexpression Suppresses Cell Proliferation, Migration, and Invasion in HNSCC Cells

**Figure 3:**
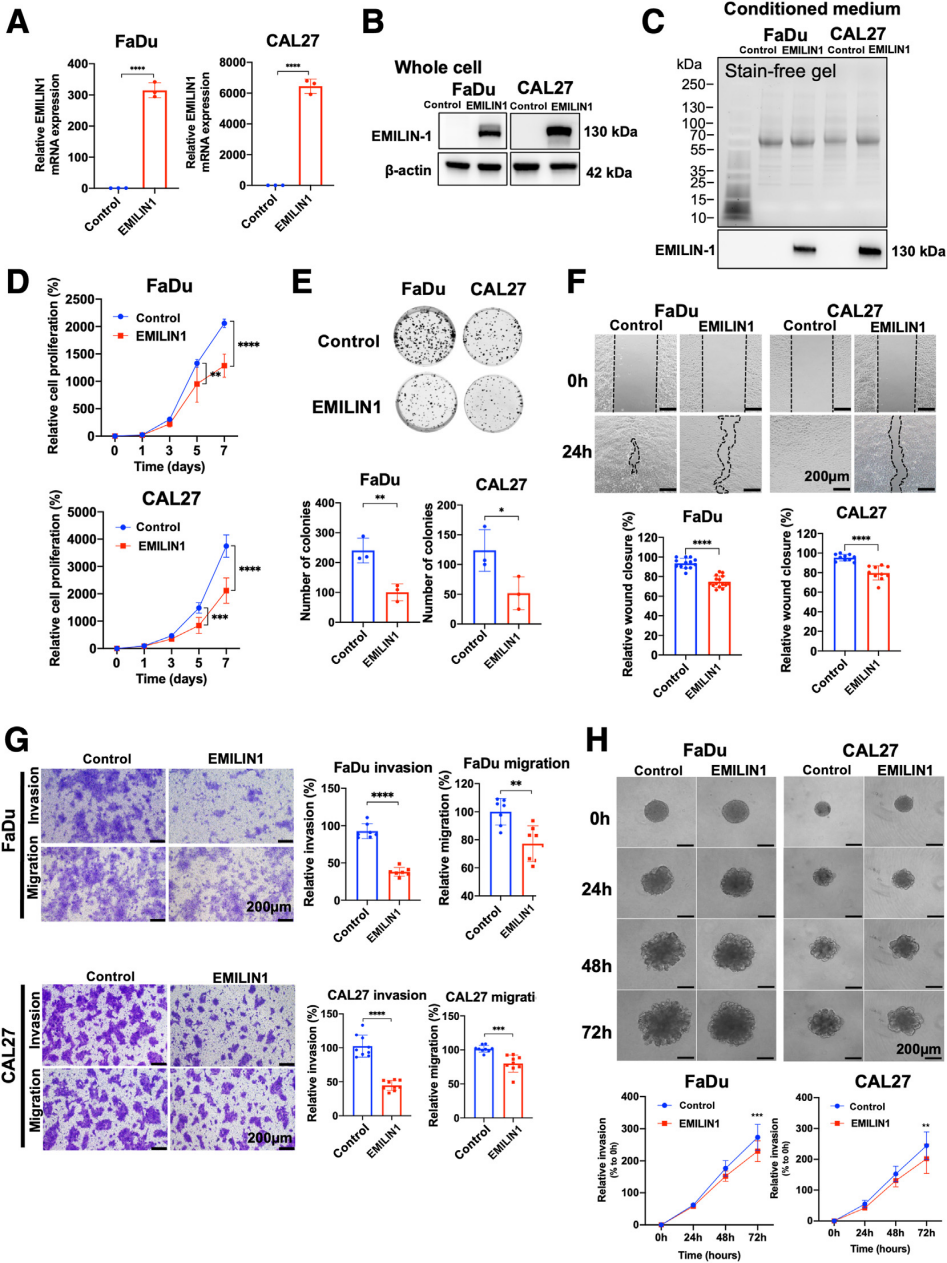
EMILIN-1 overexpression suppresses cell proliferation, migration, and invasion in head and neck squamous cell carcinoma (HNSCC) cells. EMILIN1 mRNA expression (**A**) and protein expression [whole cell (**B**) and CM (**C**)] confirm the overexpression. The effect of EMILIN-1 overexpression on cell proliferation (**D**), colony formation (**E**), wound healing (**black dashed line** indicates wound edge) (**F**), Transwell cell migration and invasion (**G**), and three-dimensional tumor spheroid invasion in FaDu and CAL27 cells (**H**). Data are expressed as means ± SD. *n* = 3. ∗*P* < 0.05 ∗∗*P* < 0.01, ∗∗∗*P* < 0.001, and ∗∗∗∗*P* < 0.0001. Scale bars = 200 μm (**F**–**H**).

To explore the potential role of EMILIN-1 as a tumor suppressor gene in HNSCC, stable EMILIN-1 overexpression was established in FaDu and CAL27 cell lines using lentiviral transduction. Successful overexpression was verified in both cell lysate and secreted protein by quantitative RT-PCR and Western blot analyses (*P* < 0.0001) (Figure 3, A–C). Cell proliferation was significantly inhibited after EMILIN-1 overexpression, starting from day 5 in both FaDu and CAL27 cells (*P* < 0.01) (Figure 3D). Colony formation assay showed a reduction in the number of colonies in EMILIN-1–overexpressing cells compared with controls in both FaDu and CAL27 cells (*P* < 0.05) (Figure 3E). Wound healing, Transwell migration, and invasion assays revealed decreased cell migration in EMILIN-1–overexpressing cells compared with control cells (*P* < 0.01) (Figure 3, F and G). Furthermore, three-dimensional spheroid invasion assay indicated that EMILIN-1 overexpression significantly reduced invasion in both FaDu and CAL27 cells (*P* < 0.01) (Figure 3H). Collectively, these results suggest that EMILIN-1 suppresses HNSCC cell proliferation, migration, and invasion.

### EMILIN-1 Knockdown Promotes Cell Proliferation and Migration in CAFs

**Figure 4:**
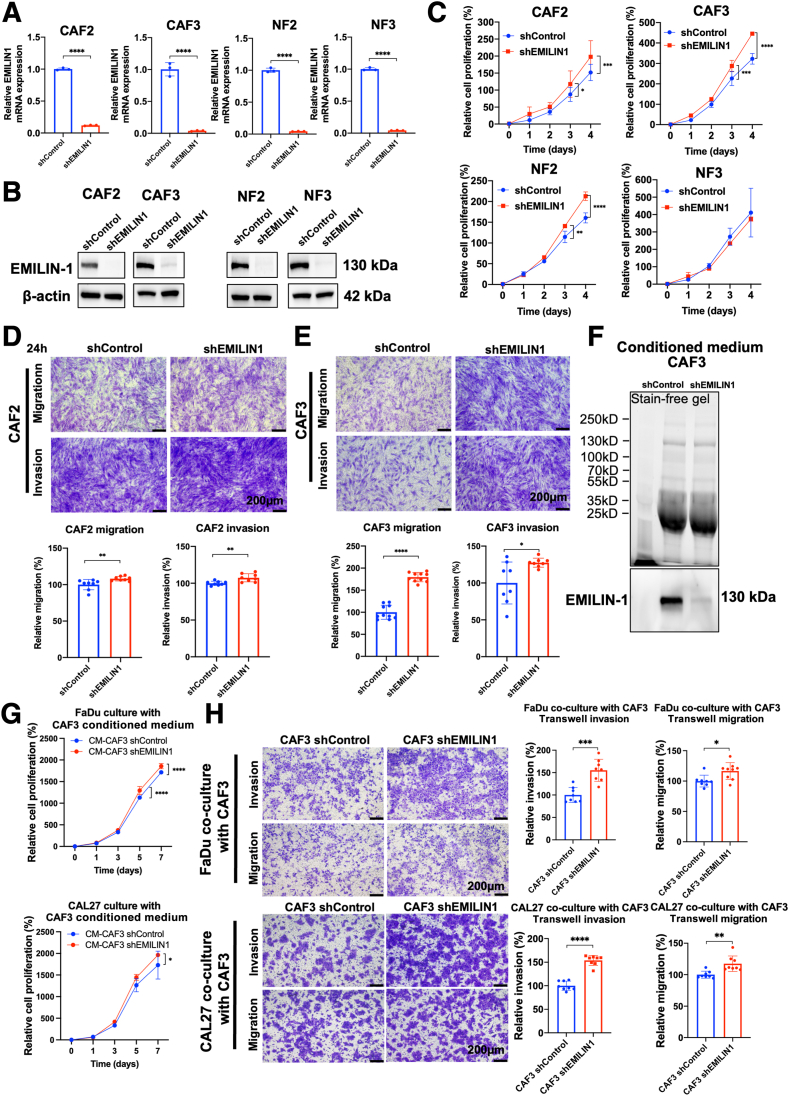
EMILIN-1 knockdown promotes cell proliferation and migration in cancer associated fibroblasts (CAFs). **A** and **B**: Expression of EMILIN-1 knockdown by shRNA confirmed both in mRNA expression (**A**) and protein expression levels (**B**). **C:** Effect of EMILIN-1 knockdown on cell proliferation in fibroblast cells. **D** and **E:** Effect of EMILIN-1 knockdown in CAFs on cell migration and invasion. **F:** EMILIN-1 protein expression in CAF3 cultured medium (CM) used in experiments. **G:** Cell proliferation of cancer cells cultured with EMILIN-1 knockdown CAF3-CM. **H:** Transwell migration and invasion of cancer cells co-cultured with EMILIN-1 knockdown CAF3 cell. Data are expressed as means ± SD. *n* = 3. ∗*P* < 0.05, ∗∗*P* < 0.01, ∗∗∗*P* < 0.001, and ∗∗∗∗*P* < 0.0001. Scale bars = 200 μm (**D**, **E**, and **H**).

**Supplemental Figure S6:**
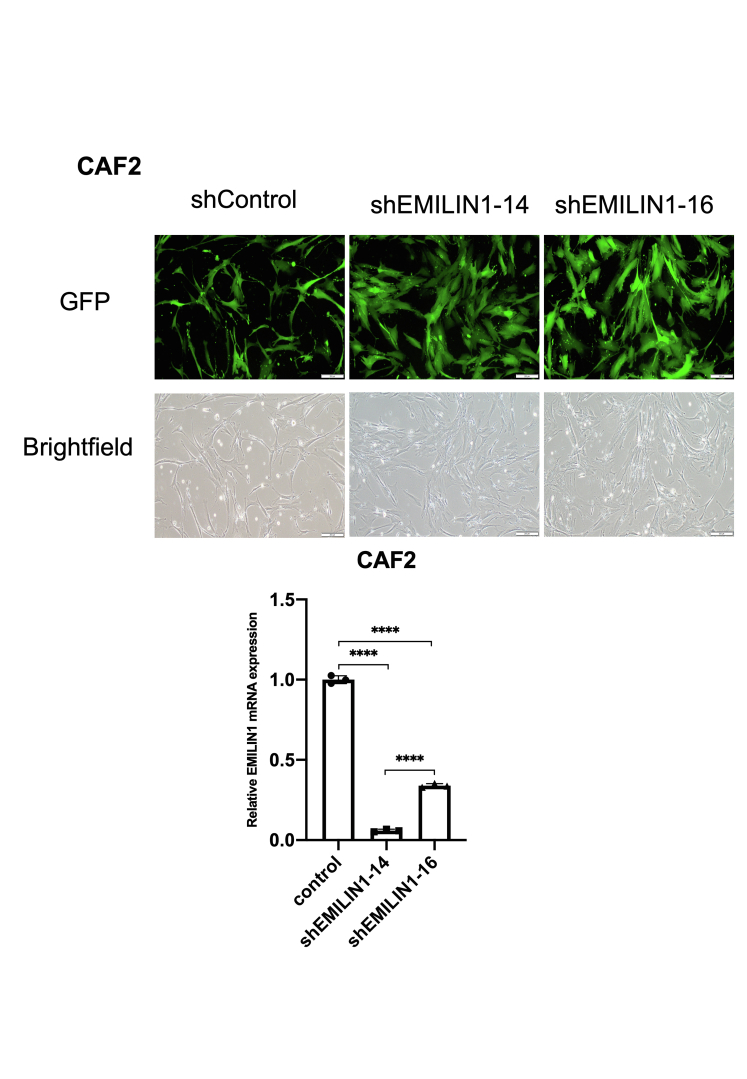
EMILIN-1 knockdown efficiency by shRNA. Representative images of GFP fluorescence and *EMILIN1* mRNA expression level show the knockdown efficiency of shControl compared with shEMILIN1-14 and shEMILIN1-16 in CAF2 cells. ∗∗∗∗*P* < 0.0001. Scale bars = 200 μm.

Given the higher expression of EMILIN-1 in fibroblasts compared with that in HNSCC cells, the effect of EMILIN-1 knockdown was further investigated in CAFs and NFs. Stable shEMILIN1 and shControl cells were generated using lentiviral transduction. The shEMILIN1-14 was chosen from two shRNAs due to its higher knockdown efficiency (*P* < 0.0001) (Supplemental Figure S6). Effective knockdown of EMILIN-1 across all fibroblast cells was confirmed by quantitative RT-PCR and Western blot analyses (*P* < 0.0001) (Figure 4, A and B). Increased cell proliferation with EMILIN-1 knockdown was observed in CAF2, CAF3, and NF2 cells starting from day 3 (*P* < 0.05) (Figure 4C). Additionally, Transwell migration and invasion assays demonstrated that EMILIN-1 knockdown notably increased cell migration and invasion in CAF2 and CAF3 cells (*P* < 0.05) (Figure 4, D and E). These results suggest that EMILIN-1 knockdown promotes cell proliferation in both CAFs and NFs isolated from head and neck tissue.

To further validate the effect of secreted EMILIN-1 on HNSCC cells, CM was collected from EMILIN-1 knockdown CAF3 and control CAF3 cells, concentrated and confirmed for EMILIN-1 protein (Figure 4F). The cancer cells were treated with these CM. The CM from EMILIN-1 knockdown CAFs increased HNSCC cell proliferation compared with the control CM by day 7 (*P* < 0.05) (Figure 4G). Furthermore, when co-culturing EMILIN-1 knockdown CAF3 and cancer cells using a Transwell insert system, FaDu and CAL27 cells in the upper chamber showed increased cell migration (*P* < 0.001) and invasion (*P* < 0.05) (Figure 4H). These results confirm the inhibitory effect of EMILIN-1 on cancer cell proliferation, migration, and invasion.

### Gene Expression Analyses Reveal EMILIN-1 as a Potential Regulator of Cell Cycle and Aurora Kinase Signaling Pathway

**Supplemental Figure S7:**
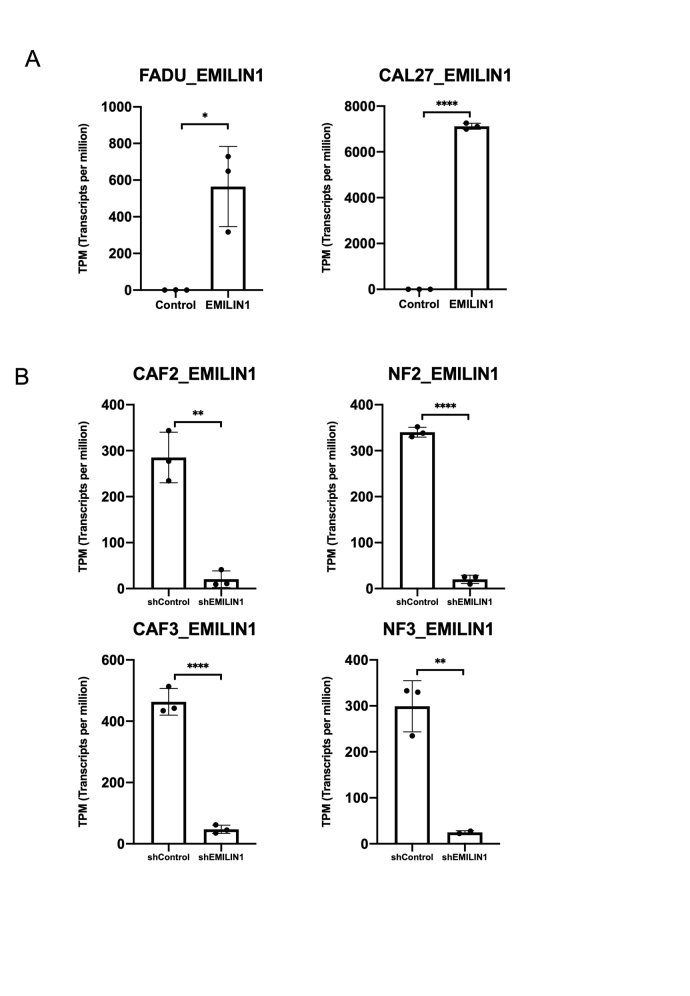
*EMILIN1* expression from RNA-seq analysis. **A:***EMILIN1* expression in FaDu and CAL27 cell lines after overexpressing EMILIN-1. **B:***EMILIN1* expression in all fibroblast cells after knockdown EMILIN-1. Data were analyzed from RNA sequencing. ∗*P* < 0.05, ∗∗*P* < 0.01, and ∗∗∗∗*P* < 0.0001.

**Supplemental Figure S8:**
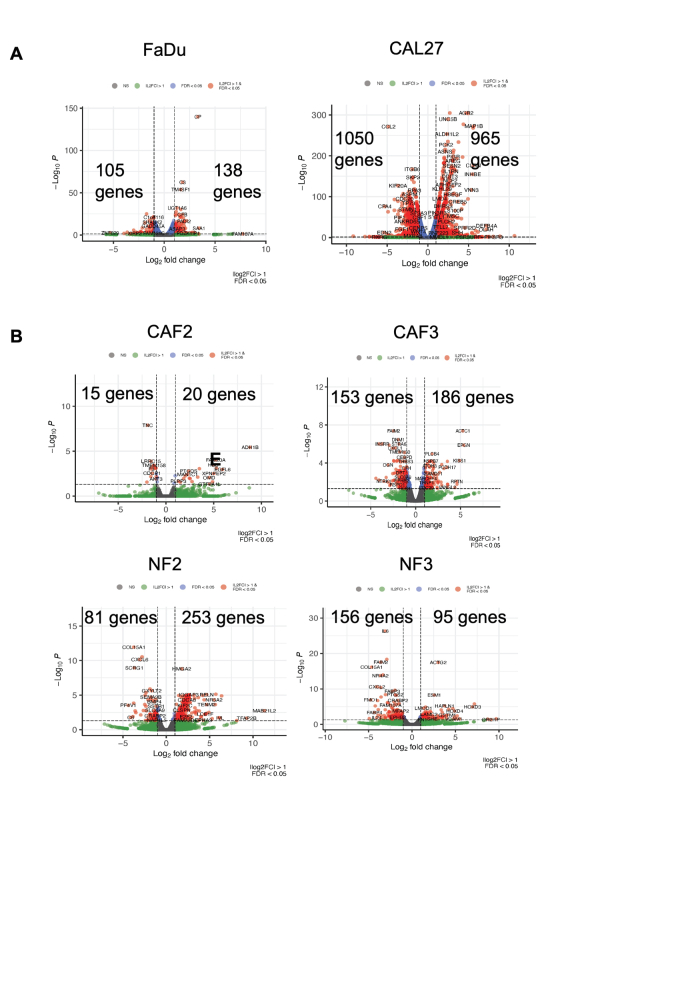
Volcano plot of all the genes from RNA-seq. Volcano plot of all genes from RNA-seq analysis in FaDu and CAL27 cell lines after overexpressing EMILIN-1 (**A**), all fibroblast cells after knockdown EMILIN-1 (**B**). The two **vertical dashed lines** correspond to log2(FC) ≥1 (right) and ≤−1 (left), and the **horizontal dashed line** represents false discovery rate = 0.05 as the threshold cutoff. Each plot labeled the number of DEGs of each cell.

RNA-seq analysis of EMILIN-1–overexpressing FaDu and CAL27 cell lines, as well as EMILIN-1 knockdown CAFs and NFs cells, was conducted to explore the underlying mechanisms of EMILIN-1 in HNSCC. Upregulation of *EMILIN1* was confirmed in FaDu and CAL27 cells, with higher transduction efficiency observed in CAL27 (Supplemental Figure S7A), while *EMILIN1* was evenly down-regulated in all fibroblast cells (Supplemental Figure S7B). Differential gene expression analysis identified both up-regulated and down-regulated DEGs for each cell type, as shown in the volcano plot (|log2-fold change| > 1, false discovery rate–adjusted *P* < 0.05) (Supplemental Figure S8) and listed in Supplemental Tables S1–S12.

**Figure 5:**
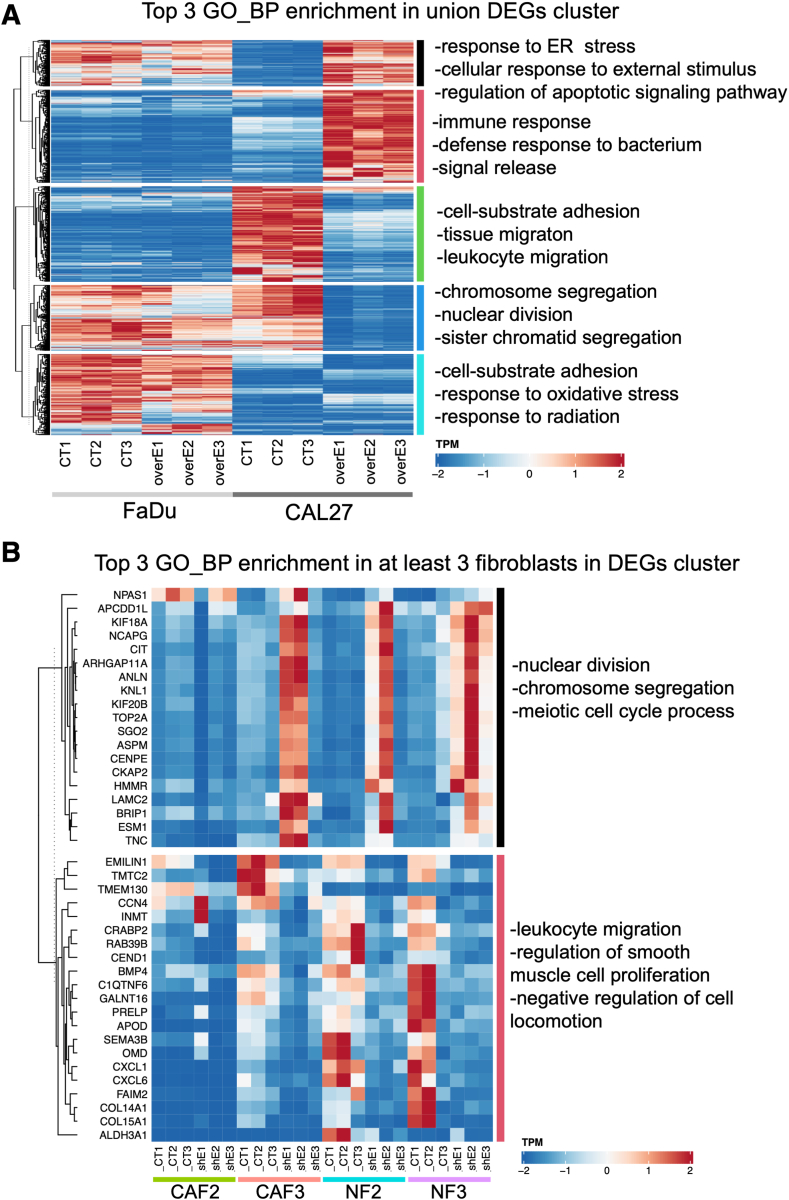
Enrichment gene set analysis of differentially expressed genes in EMILIN-1 overexpressed head and neck squamous cell carcinoma (HNSCC) cells and EMILIN-1 knockdown fibroblast cells. Heatmap of the GO-BP gene set enrichment cluster from intersected differentially expressed genes (DEGs) in FaDu and Cal27 cells (**A**) and DEGs from at least three fibroblast cells (**B**). ER, endoplasmic reticulum.

**Figure 6:**
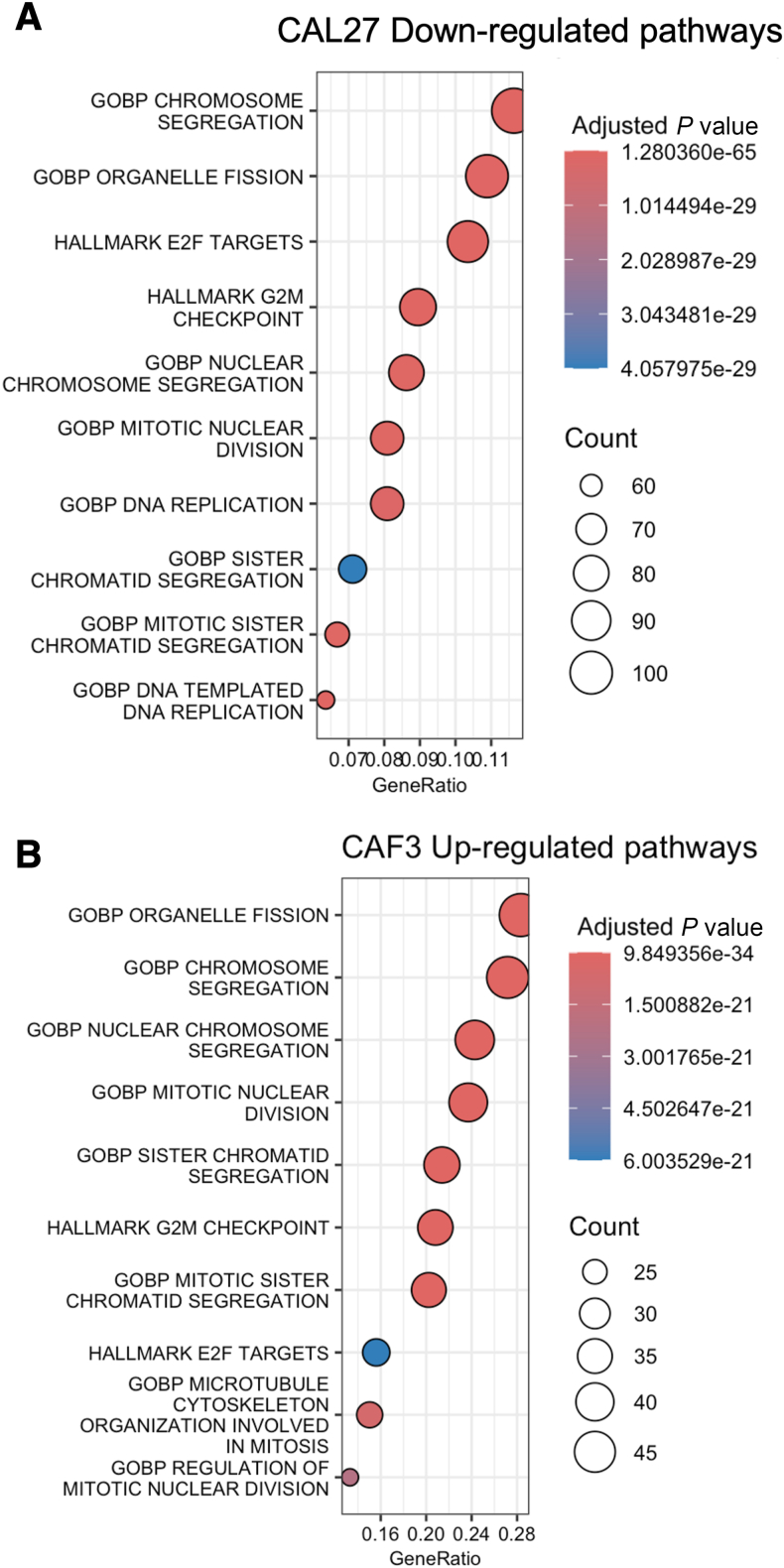
GSEA of differentially expressed genes in EMILIN-1–overexpressed HNSCC cells and EMILIN-1 knockdown fibroblast cells. The Hallmark, GO, and KEGG gene enrichment dot plots show a similar gene set in down-regulated differentially expressed genes (DEGs) from EMILIN-1-overexpressed CAL27 cells (**A**) and up-regulated DEGs from EMILIN-1-knockdown CAF3 cells (**B**).

**Supplemental Figure S9:**
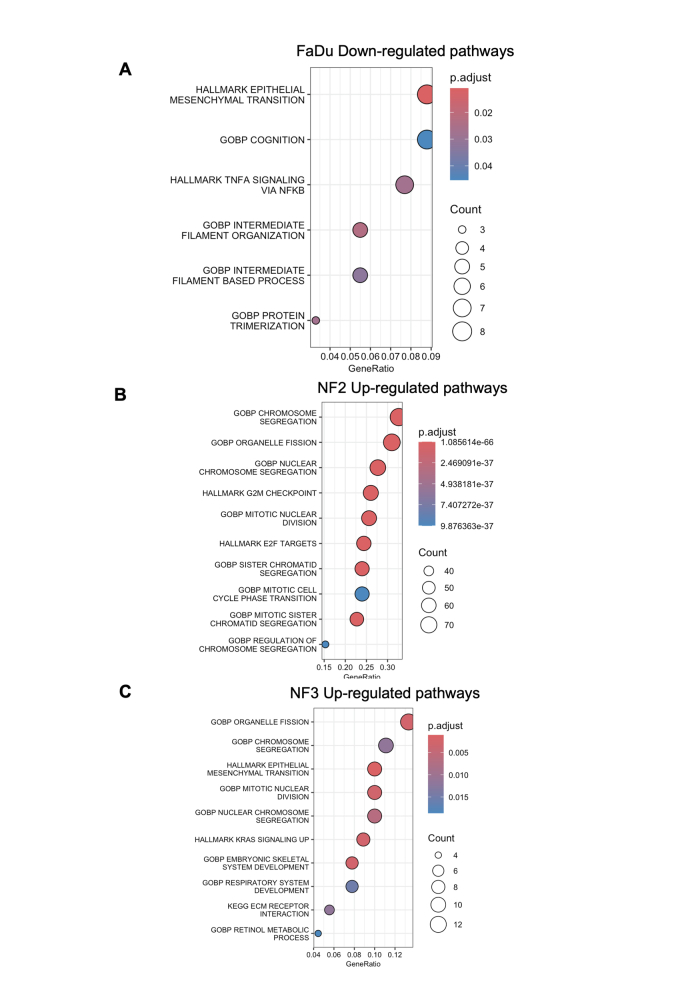
Top 10 enrichment in combined GO-Biological process/gseaKEGG/Hallmark gene set. The enrichment shows down-regulated DEGs in FaDu cells (**A**) and up-regulated DEGs in NF2 (**B**) and NF3 cells (**C**).

**Supplemental Figure S10:**
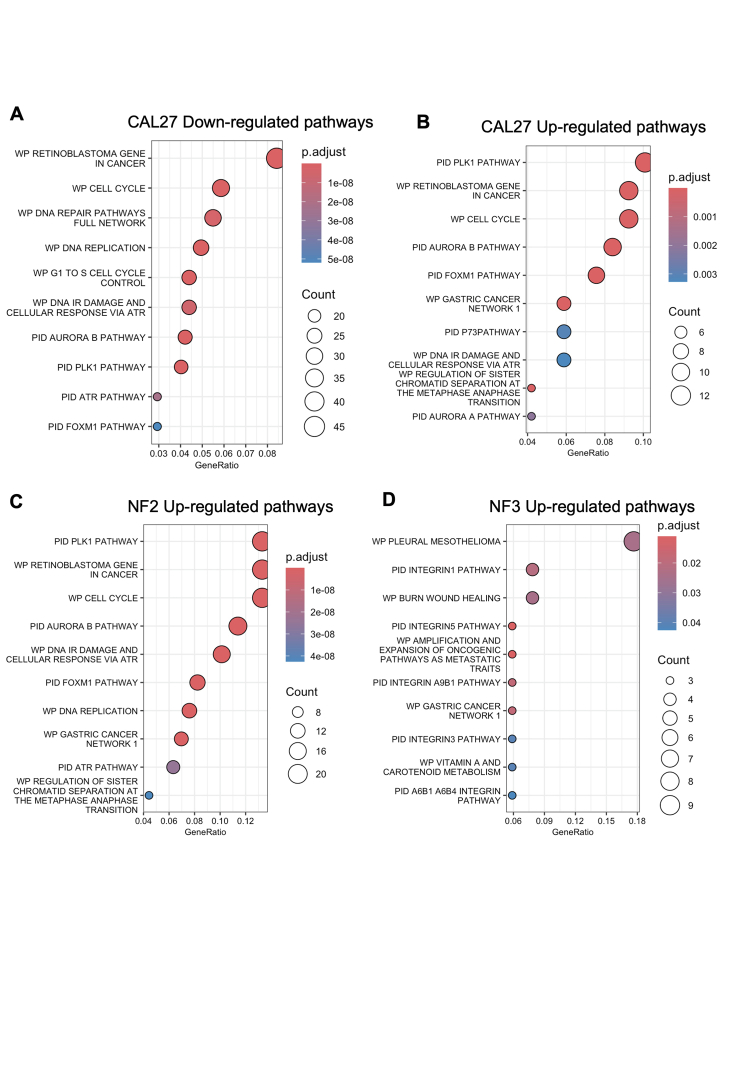
Top 10 enrichment in combined wiki pathway and PID gene set. Pathway analysis shows down-regulated DEGs in CAL27 cells (**A**) and up-regulated DEGs in CAF3 (**B**), NF2 (**C**), and NF3 cells (**D**).

**Supplemental Figure S11:**
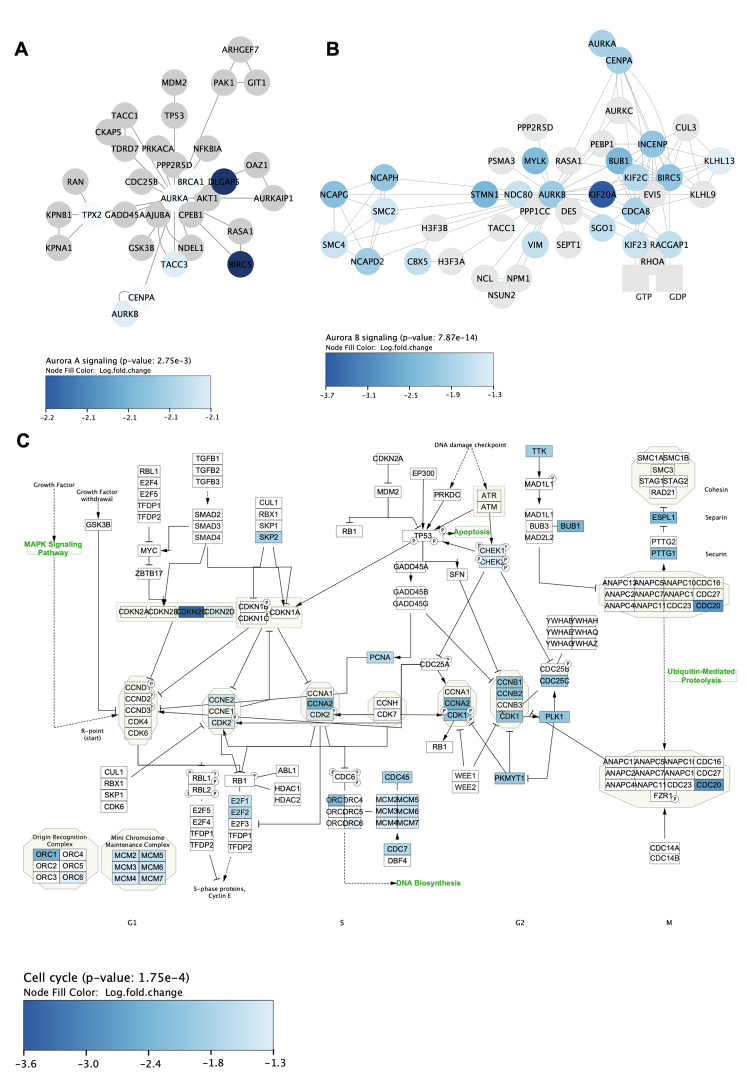
Pathway network analysis of EMILIN-1 in CAL27 cell. Pathway analysis from Cytoscape shows the association of *EMILIN1* with Aurora A signaling (**A**), Aurora B signaling (**B**), and cell cycle pathway in EMILIN-1 overexpressed CAL27 (**C**). The blue indicates down-regulated DEGs.

**Supplemental Figure S12:**
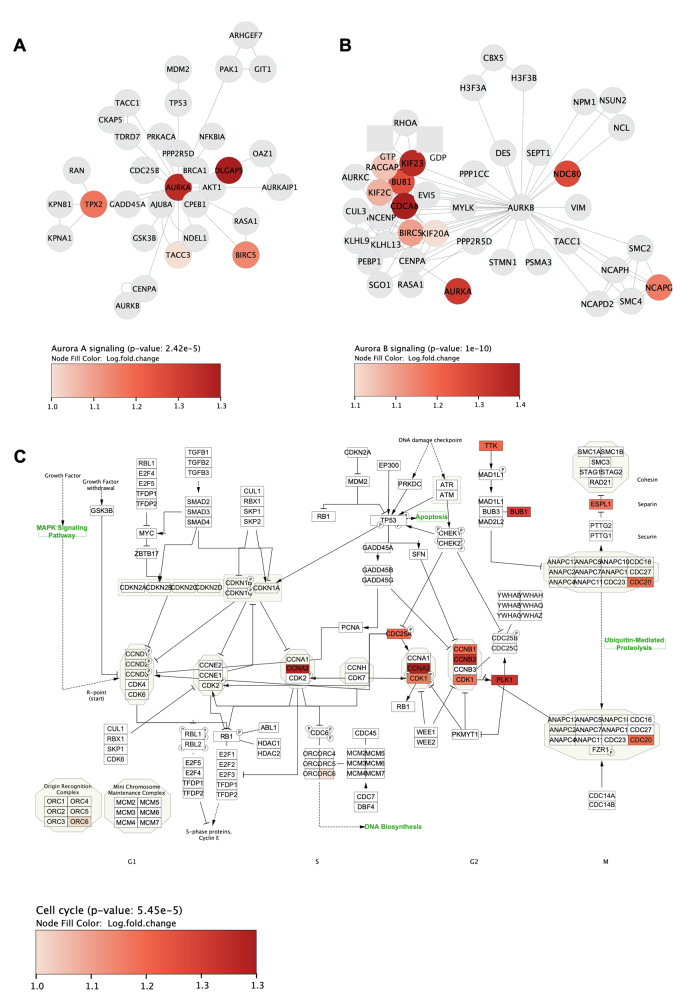
Pathway network analysis of EMILIN-1 in CAF3 cell. Pathway analysis from Cytoscape shows the association of *EMILIN1* with Aurora A signaling (**A**), Aurora B signaling (**B**), and cell cycle pathway in EMILIN-1 knockdown CAF3 (**C**). The red indicates up-regulated DEGs.

To identify the common enrichment gene sets in HNSCC cells, a heatmap of DEGs from EMILIN-1–overexpressing FaDu and CAL27 cells was generated and annotated with Gene Ontology (GO)-Biological Processes (BP) gene sets. A down-regulated gene cluster in at least one of the two cell lines (FaDu, CAL27) was identified, showing associations with chromosome segregation, nuclear division, and sister chromatid segregation (Figure 5A). Similarly, an up-regulated gene cluster in at least three of the four fibroblast cells (CAF2, CAF3, NF2, and NF3) was also linked to nuclear division, chromosome segregation, and meiotic cell cycle (Figure 5B). Additionally, GSEA using Hallmark, GO, KEGG gene sets showed significant enrichment of E2F Targets and G2M checkpoint in both down-regulated DEGs of CAL27 and up-regulated DEGs of CAF3 and NF2 (*P* < 0.0001) (Figure 6 and Supplemental Figure S9). For more specific pathway analysis, Cytoscape was used to further investigate enriched signaling pathways. Cell cycle and related gene pathways, including those involving Aurora B (*AURKB*), Aurora A (*AURKA*), and PLK1, were among the top 10 pathways in the down-regulated DEGs of CAL27 and up-regulated DEGs of CAF3 and NF2 (*P* < 0.001) (Supplemental Figure S10). The pathway networks of Aurora A, Aurora B, and cell cycle pathways are shown in Supplemental Figures S11 and S12. These findings suggest that cell cycle–related pathways are the most significantly enriched gene sets affected by EMILIN-1.

### EMILIN-1 Induces Cell Cycle Arrest and Apoptosis, with Aurora Kinases and PI3K/AKT Signaling Pathway Association

**Figure 7:**
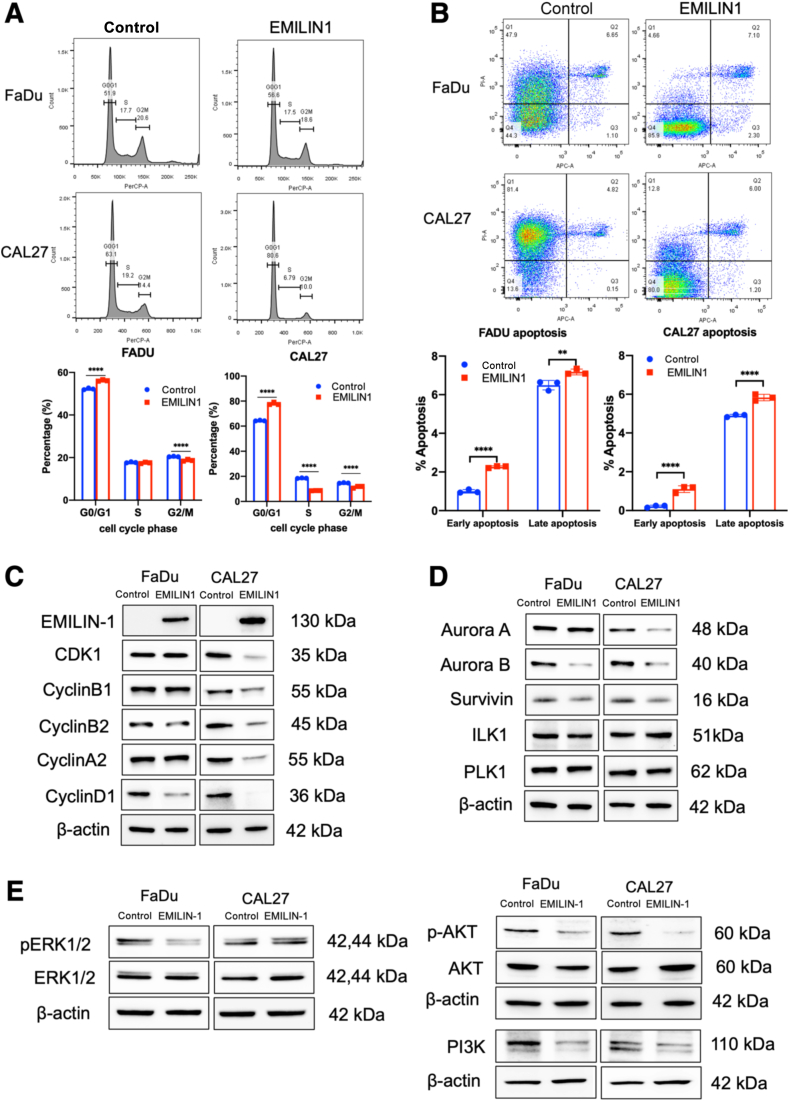
EMILIN-1 induces cell cycle arrest and apoptosis, with aurora kinases and PI3K/AKT signaling pathway association. **A** and **B:** Effect of EMILIN-1 on cell cycle distribution (**A**) and apoptosis assay in FaDu and CAL27 cells by flow cytometry (**B**). **C**–**E:** Protein expression of cell cycle regulator (**C**), aurora kinase signaling (**D**), and PI3K/AKT signaling in overexpressed EMILIN-1 cells (**E**). Data are expressed as means ± SD. *n* = 3. ∗∗*P* < 0.01, ∗∗∗∗*P* < 0.0001 two-way analysis of variance with Sidak's multiple comparisons test.

Results from RNA-sequencing analysis led to further investigation into the role of EMILIN-1 in cell cycle regulation. Cell cycle analysis showed that EMILIN-1 overexpression induced G0/G1 cell cycle arrest in both FaDu and CAL27 cells, with a significant increase in the G0/G1 phase and a corresponding decrease in other phases (*P* < 0.0001) (Figure 7A). Annexin V staining further demonstrated an increase in early and late apoptosis populations upon EMILIN-1 overexpression in FaDu and CAL27 cells (*P* < 0.001) (Figure 7B).

Western blot analysis was performed to evaluate the protein expression levels of cell cycle regulators and their signaling molecules. In CAL27 cells overexpressing EMILIN-1, the protein levels of cell cycle regulators such as CDK1, cyclin B1, cyclin B2, cyclin A2, and cyclin D1 were significantly reduced compared with controls. In FaDu cells, only cyclin B2 and cyclin D1 showed reduced expression (Figure 7C). Regarding aurora kinase signaling, EMILIN-1 overexpression led to decreased Aurora B protein levels in both FaDu and CAL27 cells, whereas Aurora A expression was reduced only in CAL27 cells. Survivin, a binding partner of Aurora B, exhibited a slight reduction in protein levels in both cell lines (Figure 7D). Additionally, previously reported EMILIN-1–related intracellular signaling pathways were investigated and it was found that EMILIN-1 overexpression resulted in reduced PI3K and p-AKT protein levels in both FaDu and CAL27 cells (Figure 7E).

**Supplemental Figure S13:**
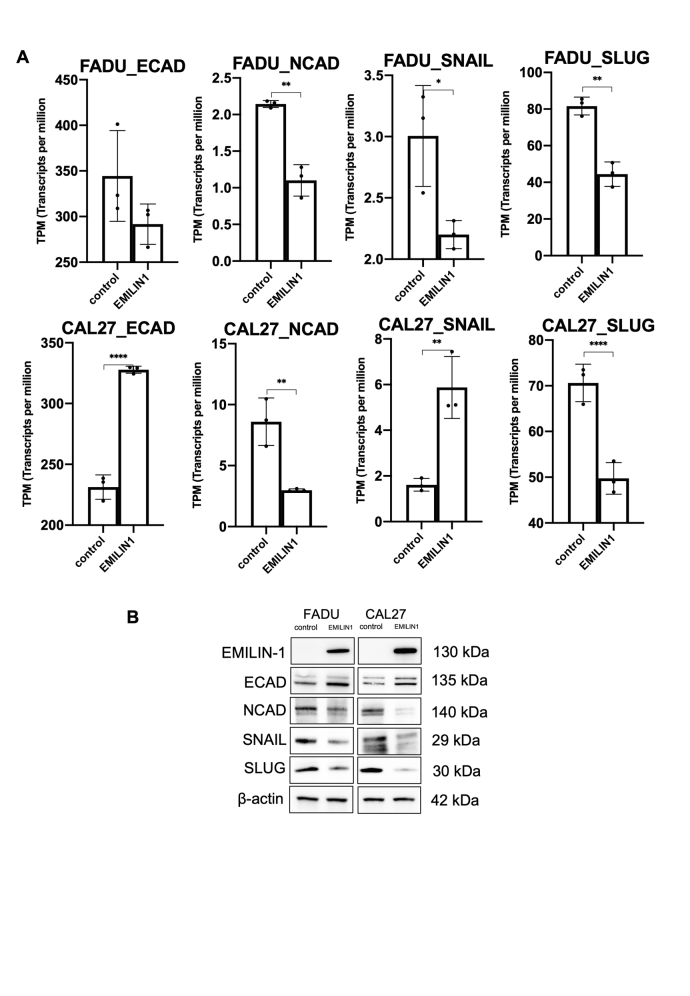
Effect of EMILIN-1 on EMT marker expression. EMT marker mRNA expression from RNA-seq analysis (**A**) and protein expression level from Western blot analysis in FaDu and CAL27 cells (**B**). ∗*P* < 0.05, ∗∗*P* < 0.01, and ∗∗∗∗*P* < 0.0001.

Given that EMILIN-1 suppresses HNSCC cell migration and invasion, its association with the epithelial-to-mesenchymal transition (EMT) process was also explored and inhibition of some marker proteins in FaDu and CAL27 cells overexpressing EMILIN-1 was observed (Supplemental Figure S13). These findings suggest that EMILIN1 induces cell cycle arrest and apoptosis, with the down-regulation of aurora kinase and PI3K/AKT signaling pathways.

### EMILIN-1 Overexpression Reduces Tumor Growth and Enhances Apoptosis *in Vivo*

**Figure 8:**
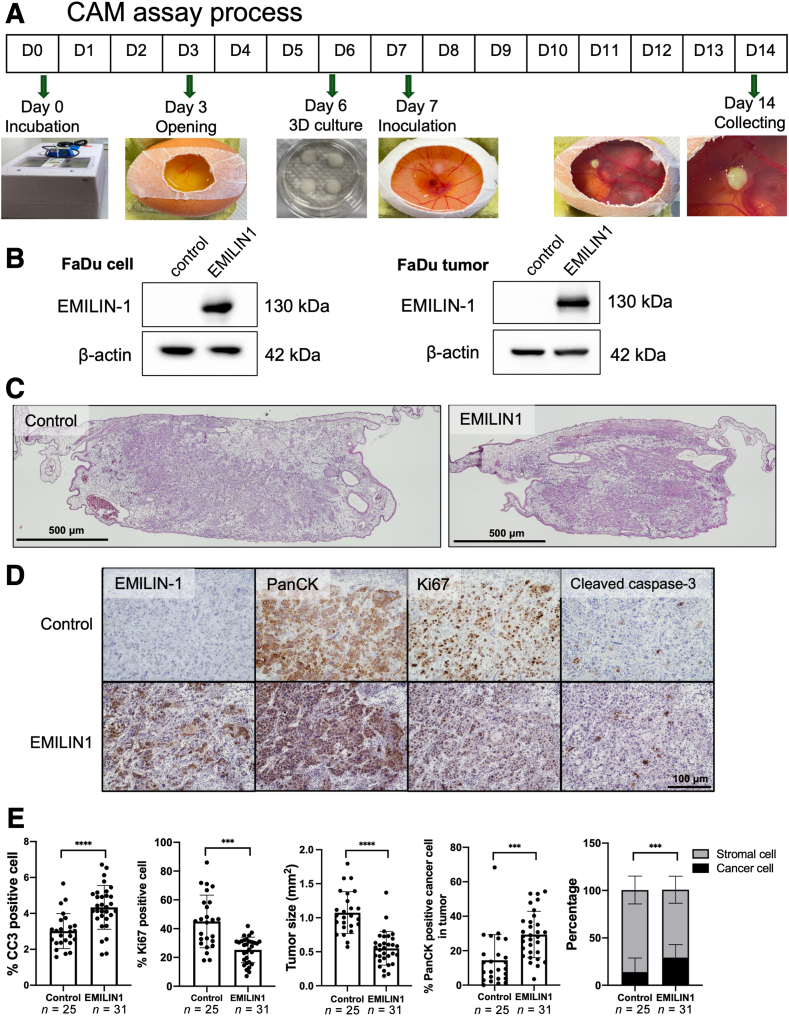
EMILIN-1 overexpression reduces tumor growth and enhances apoptosis *in vivo*. **A** and **B:** Experimental procedure of chick chorioallantoic membrane (CAM) assay (**A**) EMILIN-1 protein expression level in FaDu cells before inoculation and in CAM tumors after tumor growth (**B**). **C:** Hematoxylin and eosin staining of control tumors and EMILIN-1–overexpressed tumors showing cancer cells in dark purple. **D** and **E:** Immunohistochemistry analysis of EMILIN-1, Pan-Cytokeratin, Ki-67, and CC3 (**D**) with quantitative analysis of each marker (**E**). *n* = 25 control (**E**); *n* = 31 EMILIN-1 (**E**). ∗∗∗*P* < 0.001, ∗∗∗∗*P* < 0.0001. Scale bars: 500 μm (**C**); 100 μm (**D**).

The effect of EMILIN-1 on tumor development was evaluated in FaDu cells using the *in vivo* CAM assay, as illustrated in Figure 8A. The CAM assay was performed by inoculating a three-dimensional culture of transfected FaDu cells into chick embryos on day 7, and the growing tumors were collected on day 14 before preparing formalin-fixed paraffin-embedded tissue blocks. EMILIN-1 overexpression was confirmed in both transduced FaDu cells and FaDu tumors formed on the CAM (Figure 8B). Hematoxylin and eosin staining results showed successful tumor cell development attached to the CAM (Figure 8C). The implanted tumors were further stained with Pan-Cytokeratin to detect invading human cancer cells, Ki-67 to identify proliferating cells, and CC3 to detect apoptotic cells (*n* = 25 FaDu control tumors and *n* = 31 FaDu EMILIN-1 tumors).

Immunohistochemistry analysis revealed that tumors with EMILIN-1 overexpression exhibited a higher percentage of CC3-positive apoptotic cells compared with control tumors (*P* < 0.0001). Additionally, EMILIN-1 tumors showed a lower percentage of Ki-67–positive proliferating cells and smaller tumor sizes (*P* < 0.001). By contrast, control tumors contained more stromal and fibroblast cells, resulting in larger tumors but a lesser percentage of pan-cytokeratin–labeled cancer cells (Figure 8, D and E). These findings suggest that EMILIN-1 exerts a tumor growth inhibitory effect in chick embryo HNSCC tumors.

## Discussion

EMILIN-1, an ECM glycoprotein, has shown tumor-suppressive effects in colon cancer, melanoma, and breast cancer.^20^^,^^22^^,^^23^ Its protective role against SPM development in HNSCC has been reported.^9^ However, the roles of EMILIN-1 in HNSCC have never been studied, and the mechanisms by which EMILIN-1 signals cell activity remain elusive. This study investigated the effect of EMILIN-1 in HNSCC cell lines and CAFs. The findings demonstrated that EMILIN-1 is negatively associated with the cell cycle pathway in HNSCC and plays an important role in inhibiting cell proliferation both *in vitro* and *in vivo*.

The expression of EMILIN-1 varies across different cancer types. Normal fibroblasts and CAFs expressed high levels of both endogenous and secreted EMILIN-1, whereas HNSCC cell lines only expressed low levels. Contrary to its potential role as a candidate tumor suppressor, TCGA-HNSC data showed higher *EMILIN1* expression in tumors compared with normal epithelial tissues. Immunohistochemical analysis in HNSCC tissues demonstrated that EMILIN-1 predominantly localized in the tumor cell surrounding. Therefore, higher expression of *EMILIN1* in TCGA-HNSC tumors may partly be explained by the high proportion of fibroblasts present in the bulk tumor volume.^24^ *EMILIN1* is highly expressed in CAF, and the high EMILIN-1 expression at the tumor margins is associated with increased CD8^+^ T-cell infiltration in breast cancer.^25^ Moreover, EMILIN-1 can be degraded by neutrophil elastase and matrix metalloproteinases such as MMP3, MMP9, and MMP14 within the TME.^26^ Despite the up-regulation of *EMILIN1* expression, extensive fragmentation has been observed in ovarian tumors, leiomyosarcomas, and undifferentiated soft tissue sarcomas, suggesting that the protein may not be functional.^27^ In HNSCC, although increased *EMILIN1* expression was observed in tumor tissues, post-translational modifications of EMILIN-1 could have a greater impact on creating a TME that promotes cell proliferation.

This study demonstrated that EMILIN-1 overexpression exerted inhibitory effects on several characteristics of HNSCC cells. FaDu and CAL27 cells overexpressing EMILIN-1 showed reduced cell proliferation, migration, and invasion. Conversely, EMILIN-1 knockdown increased cell proliferation in CAFs and NF, as well as enhanced cell migration and invasion in CAFs. Moreover, the CM from EMILIN-1 knockdown CAF increased the proliferation of HNSCC cancer cells, whereas the co-culture system enhanced cancer cell migration and invasion. The reciprocal effects observed between EMILIN-1 knockdown and EMILIN-1–overexpressing cells suggest a potential tumor suppressor role in HNSCC. These findings align with previous studies showing that EMILIN-1 expression in the ECM reduces cell proliferation in both normal cells (dermal fibroblast and keratinocyte cells)^16^ and various cancer cells (colon, liver, melanoma cancer).^18^^,^^20^^,^^22^^,^^27^ Furthermore, using the *in vivo* CAM assay with FaDu cells, EMILIN-1–overexpressing tumors exhibited significant reductions in tumor growth and Ki-67–positive proliferative cells, along with an increase in the CC3-positive apoptotic cancer cells. These results suggest that, in addition to its role as a suppressor of tumor cell proliferation and migration, EMILIN-1 also possesses proapoptotic effects.

Further transcriptomic analyses of EMLIN-1–overexpressing and knockdown cells revealed that EMILIN-1 suppressed cell proliferation by inhibiting genes in the cell cycle pathway. Significant pathways identified from DEGs shared between down-regulated genes in EMILIN-1–overexpressing HNSCC cells and up-regulated genes in EMILIN-1 knockdown CAFs were linked to chromosome segregation, nuclear division, DNA replication, and Aurora A and B kinase signaling. Cell cycle pathway–related genes were the most enriched, which may be partly due to EMILIN-1's effect on proliferation. One limitation of the RNA-seq analysis in this study is that comparing gene expression changes following EMILIN-1 overexpression in HNSCC cell lines may be influenced by *in vitro* biases or artifacts from abnormally high gene expression. However, these findings were validated through cell cycle and apoptosis analyses, confirming that EMILIN-1 induces G0/G1 arrest and increases apoptosis in FaDu and CAL27 cells.

Consistent with RNA-seq results, EMILIN-1 overexpression down-regulated cell cycle regulatory proteins, including cyclin D1, a key regulator of G1-to-S phase transition.^28^ Cyclin D1 up-regulation is linked to higher tumor stage and lymph node metastasis in HNSCC.^29^ Previous studies have shown that cyclin D1 overexpression decreases the G1/G0 population, promoting cell proliferation and cisplatin resistance in HNSCC,^30^ whereas its knockdown enhances cisplatin-induced apoptosis in oral squamous cell carcinoma.^31^ Hence, EMILIN-1–induced inhibition of cyclin D1 may contribute to the G0/G1 cell cycle arrest and increased apoptosis in HNSCC.

EMILIN-1 overexpression was found to reduce PI3K and phosphorylated-AKT protein levels in both FaDu and CAL27 cells. The PI3K/AKT signaling pathway is widely known for its ability to promote tumor cell proliferation, migration, and survival across various cancers.^32^ EMILIN-1 inhibits dermal fibroblast and keratinocyte proliferation through α4/α9β1 integrin-induced PI3K/AKT and Erk1/2 pathway activation.^16^ Intracellular signaling induced by ECM adhesion through Erk and PI3K typically promotes self-sufficient growth via cyclin D1^33^; however, signals triggered by EMILIN-1 through α4/α9β1 integrins exert an inhibitory effect on cell proliferation.^16^ Thus, the suppression of cell cycle pathway–related genes and PI3K/AKT may be partially attributed to EMILIN-1's effect on cell proliferation and other cancer-related processes, such as migration and invasion.

EMILIN-1 significantly down-regulated the protein expression of Aurora A in CAL27 and Aurora B in both FaDu and CAL27 cells. Aurora kinases are crucial for mitotic cell cycle regulation: Aurora A in centrosome maturation and spindle formation, and Aurora B in chromosome segregation, checkpoint control, and cytokinesis. Downregulation of Aurora A and B expression has been associated with inhibited cell growth and increased apoptosis in HNSCC.^34^ Aurora A has been reported to promote cell migration and invasion in HNSCC cells through the AURKA/AKT/FAK signaling pathway^35^ and EMT in colon cancer.^36^ Similarly, Aurora B promotes cell proliferation, invasion and migration through the activation of PI3K/AKT signaling pathway in intrahepatic cholangiocarcinoma.^37^ Knockdown of Aurora B can suppress AKT/GSK3β/Snail1 signaling, reverse EMT, and reduce the metastatic capability of breast cancer cells *in vitro* and *in vivo*.^38^ Inhibiting aurora kinases has shown promise in preclinical and clinical trials for solid tumors, including HNSCC.^39^, ^40^, ^41^, ^42^ In terms of potential clinical application, using aurora kinase inhibitors to suppress cancer cell proliferation may be a feasible strategy for mimicking EMILIN-1 overexpression in HNSCC.

In conclusion, this study provides evidence that EMILIN-1 exerts tumor suppressive effects in HNSCC. EMILIN-1 suppresses cell proliferation, migration, and invasion in HNSCC by down-regulating the expression of cell cycle regulators and aurora kinases likely through the PI3K/AKT signaling pathway. This study is the first to demonstrate an association between EMILIN-1 and aurora kinase expression, providing insight into the molecular mechanisms of ECM-induced signaling pathways. These findings suggest the potential development of therapeutic interventions aimed at enhancing EMILIN-1 expression or inhibiting aurora kinase expression. Further studies investigating the relationship between EMILIN-1 and aurora kinases, as well as the role of EMILIN-1 in TME, should be added to enhance understanding of HNSCC tumorigenesis.

## Disclosure Statement

None declared.
