# Supplementary material for: EMILIN-1 Suppresses Cell Proliferation through Altered Cell Cycle Regulation in Head and Neck Squamous Cell Carcinoma
Source: Am J Pathol. 2025 Jan 30;195(5):995–1012. doi: 10.1016/j.ajpath.2025.01.010 (PMC12163418; doi:10.1016/j.ajpath.2025.01.010)
Supplement: Supplemental Table S1 [file mmc1.docx]

| **Supplemental Table S1** Upregulated genes of FaDu cell line with EMILIN-1 overexpression (Log2FC>1,FDR<0.05). (https://www.ensembl.org) | | |  |  |
| --- | --- | --- | --- | --- |
|  |  |  |  |  |
| **Gene** | **Database name** | **Identifier** | **Log2FC** | **FDR p-value** |
| *EMILIN1* | Elastin microfibril interfacer 1 | ENSG00000138080 | 9.62 | 0 |
| *FAM187A* | Ig-like V-type domain-containing protein FAM187A | ENSG00000214447 | 7.74 | 0.03 |
| *SAA1* | Serum amyloid A-1 protein | ENSG00000173432 | 3.43 | 7.73E-09 |
| *PTPRCAP* | Protein tyrosine phosphatase receptor type C-associated protein | ENSG00000213402 | 3.39 | 0.03 |
| *CP* | Ceruloplasmin | ENSG00000047457 | 3.31 | 1.39E-140 |
| *GNB3* | Guanine nucleotide-binding protein G(I)/G(S)/G(T) subunit beta-3 | ENSG00000111664 | 3.02 | 9.18E-03 |
| *SPTA1* | Spectrin alpha chain, erythrocytic 1 | ENSG00000163554 | 2.96 | 1.08E-03 |
| *IGLON5* | IgLON family member 5 | ENSG00000142549 | 2.88 | 5.66E-03 |
| *ALOX5* | Polyunsaturated fatty acid 5-lipoxygenase | ENSG00000012779 | 2.75 | 6.51E-04 |
| *FOXF1* | Forkhead box protein F1 | ENSG00000103241 | 2.7 | 1.00E-02 |
| *PREX2* | Phosphatidylinositol 3,4,5-trisphosphate-dependent Rac exchanger 2 protein | ENSG00000046889 | 2.68 | 0.02 |
| *PGLYRP3* | Peptidoglycan recognition protein 3 | ENSG00000159527 | 2.58 | 2.37E-03 |
| *CD36* | Platelet glycoprotein 4 | ENSG00000135218 | 2.36 | 0.04 |
| *PDZK1IP1* | PDZK1-interacting protein 1 | ENSG00000162366 | 2.35 | 6.64E-03 |
| *C8B* | Complement component C8 beta chain | ENSG00000021852 | 2.21 | 2.57E-03 |
| *KCNJ2* | Inward rectifier potassium channel 2 | ENSG00000123700 | 2.19 | 1.35E-17 |
| *H4C15* | H4 Clustered Histone 15 | ENSG00000270276 | 2.18 | 0.01 |
| *SAA2* | Serum amyloid A-2 protein | ENSG00000134339 | 2.15 | 0.02 |
| *MEI1* | Meiosis inhibitor protein 1 | ENSG00000167077 | 2.13 | 0.02 |
| *SFTPD* | Pulmonary surfactant-associated protein D | ENSG00000133661 | 2.13 | 8.25E-03 |
| *PLA2G4B* | Cytosolic phospholipase A2 beta | ENSG00000243708 | 2.1 | 9.50E-09 |
| *TRIM31* | E3 ubiquitin-protein ligase TRIM31 | ENSG00000204616 | 2.08 | 3.60E-03 |
| *HAL* | Histidine ammonia-lyase | ENSG00000084110 | 2.07 | 5.89E-04 |
| *SLC2A4* | Solute carrier family 2, facilitated glucose transporter member 4 | ENSG00000181856 | 2.01 | 0.04 |
| *DHRS9* | Dehydrogenase/reductase SDR family member 9 | ENSG00000073737 | 2 | 6.47E-08 |
| *ACE2* | Angiotensin-converting enzyme 2 | ENSG00000130234 | 1.96 | 5.98E-17 |
| *FUT9* | 4-galactosyl-N-acetylglucosaminide 3-alpha-L-fucosyltransferase 9 | ENSG00000172461 | 1.96 | 1.77E-11 |
| *GOLGA8J* | Golgin subfamily A member 8J | ENSG00000179938 | 1.96 | 0.05 |
| *PADI2* | Protein-arginine deiminase type-2 | ENSG00000117115 | 1.94 | 5.40E-17 |
| *VGLL1* | Transcription cofactor vestigial-like protein 1 | ENSG00000102243 | 1.94 | 6.65E-15 |
| *PCSK9* | Proprotein convertase subtilisin/kexin type 9 | ENSG00000169174 | 1.89 | 8.23E-21 |
| *COLCA2* | Colorectal cancer-associated protein 2 | ENSG00000214290 | 1.86 | 9.50E-09 |
| *CRISP3* | Cysteine-rich secretory protein 3 | ENSG00000096006 | 1.83 | 1.87E-05 |
| *TGIF2-RAB5IF* | TGFB-induced factor homeobox 2-RAB5IF readthrough | ENSG00000259399 | 1.81 | 0.03 |
| *C3* | Complement C3 | ENSG00000125730 | 1.8 | 4.02E-63 |
| *CFB* | Complement factor B | ENSG00000243649 | 1.8 | 5.88E-25 |
| *PCDHGA5* | Protocadherin gamma-A5 | ENSG00000253485 | 1.78 | 0.03 |
| *SLC40A1* | Solute carrier family 40 member 1 | ENSG00000138449 | 1.77 | 0.02 |
| *SLC34A3* | Sodium-dependent phosphate transport protein 2C | ENSG00000198569 | 1.76 | 0.04 |
| *ERICH3* | Glutamate Rich 3 | ENSG00000178965 | 1.73 | 0.04 |
| *FABP6* | Gastrotropin | ENSG00000170231 | 1.72 | 0.02 |
| *HTRA3* | Serine protease HTRA3 | ENSG00000170801 | 1.7 | 8.03E-14 |
| *BBOX1* | Gamma-butyrobetaine dioxygenase | ENSG00000129151 | 1.69 | 0.03 |
| *S100A7* | Protein S100-A7 | ENSG00000143556 | 1.68 | 0.04 |
| *CXCL17* | C-X-C motif chemokine 17 | ENSG00000189377 | 1.68 | 0.01 |
| *FGF19* | Fibroblast growth factor 19 | ENSG00000162344 | 1.68 | 8.33E-11 |
| *GCNT3* | Glucosaminyl (N-Acetyl) Transferase 3, Mucin Type | ENSG00000140297 | 1.67 | 3.86E-14 |
| *ESRRG* | Estrogen-related receptor gamma | ENSG00000196482 | 1.67 | 1.22E-06 |
| *CDH5* | Cadherin-5 | ENSG00000179776 | 1.66 | 9.35E-22 |
| *AKR1C3* | Aldo-keto reductase family 1 member C3 | ENSG00000196139 | 1.64 | 5.46E-31 |
| *SLC19A3* | Thiamine transporter 2 | ENSG00000135917 | 1.64 | 7.06E-05 |
| *TM4SF1* | Transmembrane 4 L6 family member 1 | ENSG00000169908 | 1.63 | 1.49E-54 |
| *ACSL5* | Long-chain-fatty-acid--CoA ligase 5 | ENSG00000197142 | 1.63 | 1.31E-06 |
| *CES1* | Liver carboxylesterase 1 | ENSG00000198848 | 1.63 | 0.03 |
| *P2RY6* | P2Y purinoceptor 6 | ENSG00000171631 | 1.61 | 0.03 |
| *C1S* | Complement C1s subcomponent | ENSG00000182326 | 1.6 | 2.43E-23 |
| *METTL7A* | Methyltransferase-like protein 7A | ENSG00000185432 | 1.6 | 4.48E-08 |
| *TCHHL1* | Trichohyalin-like protein 1 | ENSG00000182898 | 1.58 | 8.98E-09 |
| *FOS* | Proto-oncogene c-Fos | ENSG00000170345 | 1.55 | 8.12E-22 |
| *TREML4* | Trem-like transcript 4 protein | ENSG00000188056 | 1.54 | 0.04 |
| *IQCN* | IQ domain-containing protein N | ENSG00000130518 | 1.54 | 0.03 |
| *LIMS3* | LIM and senescent cell antigen-like-containing domain protein 3 | ENSG00000256977 | 1.53 | 0.03 |
| *GABRE* | Gamma-aminobutyric acid receptor subunit epsilon | ENSG00000102287 | 1.5 | 9.77E-08 |
| *MAML2* | Mastermind-like protein 2 | ENSG00000184384 | 1.49 | 8.15E-08 |
| *ST6GALNAC1* | Alpha-N-acetylgalactosaminide alpha-2,6-sialyltransferase 1 | ENSG00000070526 | 1.49 | 1.72E-09 |
| *AMOT* | Angiomotin | ENSG00000126016 | 1.48 | 3.11E-11 |
| *UBA7* | Ubiquitin-like modifier-activating enzyme 7 | ENSG00000182179 | 1.46 | 3.32E-09 |
| *EXOC3L4* | Exocyst complex component 3-like protein 4 | ENSG00000205436 | 1.46 | 0.05 |
| *ALDH1A1* | Aldehyde dehydrogenase 1A1 | ENSG00000165092 | 1.46 | 0.02 |
| *A2ML1* | Alpha-2-macroglobulin-like protein 1 | ENSG00000166535 | 1.45 | 4.60E-04 |
| *CNR1* | Cannabinoid receptor 1 | ENSG00000118432 | 1.44 | 2.26E-04 |
| *ALDH3B2* | Aldehyde dehydrogenase family 3 member B2 | ENSG00000132746 | 1.43 | 2.03E-16 |
| *BCO2* | Beta,beta-carotene 9',10'-oxygenase | ENSG00000197580 | 1.43 | 0.02 |
| *HMOX1* | Heme oxygenase 1 | ENSG00000100292 | 1.41 | 5.49E-25 |
| *WFDC2* | WAP four-disulfide core domain protein 2 | ENSG00000101443 | 1.41 | 9.05E-06 |
| *PRICKLE4* | Prickle-like protein 4 | ENSG00000278224 | 1.39 | 6.24E-03 |
| *UGT1A1* | UDP-glucuronosyltransferase 1A1 | ENSG00000241635 | 1.39 | 3.69E-05 |
| *IL21R* | Interleukin-21 receptor | ENSG00000103522 | 1.39 | 1.44E-04 |
| *TEX19* | Testis-expressed protein 19 | ENSG00000182459 | 1.38 | 0.01 |
| *INSIG1* | Insulin-induced gene 1 protein | ENSG00000186480 | 1.38 | 2.19E-11 |
| *STEAP4* | Metalloreductase STEAP4 | ENSG00000127954 | 1.36 | 2.46E-14 |
| *JAKMIP3* | Janus kinase and microtubule-interacting protein 3 | ENSG00000188385 | 1.36 | 3.33E-04 |
| *FOLH1* | Glutamate carboxypeptidase 2 | ENSG00000086205 | 1.33 | 1.08E-03 |
| *ADRB1* | Beta-1 adrenergic receptor | ENSG00000043591 | 1.33 | 0.04 |
| *GALM* | Galactose mutarotase | ENSG00000143891 | 1.33 | 0.02 |
| *GLRX* | Glutaredoxin-1 | ENSG00000173221 | 1.32 | 7.73E-05 |
| *LGALS9* | Galectin-9 | ENSG00000168961 | 1.27 | 3.98E-04 |
| *UGT1A6* | UDP-glucuronosyltransferase 1-6 | ENSG00000167165 | 1.27 | 1.04E-32 |
| *ASAP3* | Arf-GAP with SH3 domain, ANK repeat and PH domain-containing protein 3 | ENSG00000088280 | 1.27 | 6.09E-08 |
| *RARRES1* | Retinoic acid receptor responder protein 1 | ENSG00000118849 | 1.25 | 0.04 |
| *ACY3* | N-acyl-aromatic-L-amino acid amidohydrolase (carboxylate-forming) | ENSG00000132744 | 1.24 | 0.02 |
| *DEPP1* | Protein DEPP1 | ENSG00000165507 | 1.24 | 3.38E-03 |
| *SLX1B* | SLX1 structure-specific endonuclease subunit homolog B | ENSG00000181625 | 1.24 | 0.03 |
| *CTSS* | Cathepsin S | ENSG00000163131 | 1.21 | 2.25E-16 |
| *ACKR2* | Atypical chemokine receptor 2 | ENSG00000144648 | 1.21 | 0.03 |
| *C1R* | Complement C1r subcomponent | ENSG00000159403 | 1.2 | 4.50E-11 |
| *SCD* | Stearoyl-CoA desaturase | ENSG00000099194 | 1.19 | 2.09E-09 |
| *CABYR* | Calcium-binding tyrosine phosphorylation-regulated protein | ENSG00000154040 | 1.18 | 4.93E-14 |
| *AKR1C1* | Aldo-keto reductase family 1 member C1 | ENSG00000187134 | 1.18 | 1.87E-28 |
| *S100A9* | Protein S100-A9 | ENSG00000163220 | 1.15 | 3.79E-10 |
| *N4BP2L1* | NEDD4 Binding Protein 2 Like 1 | ENSG00000139597 | 1.14 | 5.10E-07 |
| *UPK2* | Uroplakin-2 | ENSG00000110375 | 1.14 | 1.22E-06 |
| *DIO2* | Type II iodothyronine deiodinase | ENSG00000211448 | 1.13 | 1.05E-06 |
| *FERMT3* | Fermitin family homolog 3 | ENSG00000149781 | 1.12 | 0.02 |
| *ATP6V1B1* | V-type proton ATPase subunit B, kidney isoform | ENSG00000116039 | 1.12 | 0.02 |
| *MSC* | Musculin | ENSG00000178860 | 1.12 | 2.97E-13 |
| *SOD2* | Superoxide dismutase [Mn], mitochondrial | ENSG00000112096 | 1.11 | 2.59E-25 |
| *BEST1* | Bestrophin-1 | ENSG00000167995 | 1.11 | 8.49E-24 |
| *CFI* | Complement factor I | ENSG00000205403 | 1.1 | 5.98E-13 |
| *STARD4* | StAR-related lipid transfer protein 4 | ENSG00000164211 | 1.1 | 1.21E-06 |
| *CSF1* | Macrophage colony-stimulating factor 1 | ENSG00000184371 | 1.1 | 8.48E-08 |
| *SLC39A8* | Metal cation symporter ZIP8 | ENSG00000138821 | 1.1 | 1.85E-14 |
| *GBP4* | Guanylate-binding protein 4 | ENSG00000162654 | 1.08 | 2.76E-08 |
| *VGLL3* | Transcription cofactor vestigial-like protein 3 | ENSG00000206538 | 1.07 | 0.05 |
| *UPK3B* | Uroplakin-3b | ENSG00000243566 | 1.07 | 2.49E-06 |
| *SAP25* | Histone deacetylase complex subunit SAP25 | ENSG00000205307 | 1.07 | 0.03 |
| *HS3ST3B1* | Heparan sulfate glucosamine 3-O-sulfotransferase 3B1 | ENSG00000125430 | 1.07 | 0.04 |
| *SECTM1* | Secreted and transmembrane protein 1 | ENSG00000141574 | 1.07 | 1.26E-04 |
| *FOSB* | Protein fosB | ENSG00000125740 | 1.07 | 2.69E-10 |
| *FGD3* | FYVE, RhoGEF and PH domain-containing protein 3 | ENSG00000127084 | 1.06 | 2.15E-07 |
| *SELENOP* | Selenoprotein P | ENSG00000250722 | 1.05 | 4.43E-10 |
| *C4B* | Complement C4-B | ENSG00000224389 | 1.05 | 0.02 |
| *APOD* | Apolipoprotein D | ENSG00000189058 | 1.05 | 8.27E-03 |
| *CALCRL* | Calcitonin gene-related peptide type 1 receptor | ENSG00000064989 | 1.04 | 0.02 |
| *CSKMT* | Citrate synthase-lysine N-methyltransferase CSKMT, mitochondrial | ENSG00000214756 | 1.03 | 0.03 |
| *DRAM1* | DNA damage-regulated autophagy modulator protein 1 | ENSG00000136048 | 1.03 | 5.68E-14 |
| *GPAT3* | Glycerol-3-phosphate acyltransferase 3 | ENSG00000138678 | 1.03 | 6.09E-08 |
| *MAN1A1* | Mannosyl-oligosaccharide 1,2-alpha-mannosidase IA | ENSG00000111885 | 1.03 | 2.41E-03 |
| *KIF5C* | Kinesin heavy chain isoform 5C | ENSG00000168280 | 1.02 | 3.88E-04 |
| *RNF128* | E3 ubiquitin-protein ligase RNF128 | ENSG00000133135 | 1.01 | 3.09E-03 |
| *ACSS1* | Acetyl-coenzyme A synthetase 2-like, mitochondrial | ENSG00000154930 | 1.01 | 6.59E-04 |
| *MUC4* | Mucin-4 | ENSG00000145113 | 1.01 | 3.45E-07 |
| *CCSER1* | Coiled-Coil Serine Rich Protein 1 | ENSG00000184305 | 1.01 | 9.91E-03 |
| *TJP3* | Tight junction protein ZO-3 | ENSG00000105289 | 1.01 | 4.38E-06 |
| *CCDC78* | Coiled-coil domain-containing protein 78 | ENSG00000162004 | 1.01 | 7.01E-04 |
| *AGR2* | Anterior gradient protein 2 homolog | ENSG00000106541 | 1 | 3.94E-04 |
| *INSYN1* | Inhibitory synaptic factor 1 | ENSG00000205363 | 1 | 0.01 |
| *SRPX2* | Sushi repeat-containing protein SRPX2 | ENSG00000102359 | 1 | 2.09E-08 |
