# Supplementary material for: EMILIN-1 Suppresses Cell Proliferation through Altered Cell Cycle Regulation in Head and Neck Squamous Cell Carcinoma
Source: Am J Pathol. 2025 Jan 30;195(5):995–1012. doi: 10.1016/j.ajpath.2025.01.010 (PMC12163418; doi:10.1016/j.ajpath.2025.01.010)
Supplement: Supplemental Table S2 [file mmc2.docx]

| **Supplemental Table S2** Downregulated genes of FaDu cell line with EMILIN-1 overexpression (Log2FC<-1,FDR<0.05). (https://www.ensembl.org) | | |  |  |
| --- | --- | --- | --- | --- |
|  |  |  |  |  |
| **Gene** | **Database name** | **Identifier** | **Log2FC** | **FDR p-value** |
| *ZNF829* | Zinc finger protein 829 | ENSG00000185869 | -5.33 | 2.44E-03 |
| *GAST* | Gastrin | ENSG00000184502 | -3.96 | 0.01 |
| *CDH11* | Cadherin-11 | ENSG00000140937 | -3.65 | 4.23E-04 |
| *KRTAP3-2* | Keratin-associated protein 3-2 | ENSG00000212900 | -3.38 | 0.02 |
| *SUN3* | SUN domain-containing protein 3 | ENSG00000164744 | -3.29 | 0.03 |
| *SI* | Sucrase-isomaltase, intestinal | ENSG00000090402 | -3.12 | 0.05 |
| *ZNF43* | Zinc finger protein 43 | ENSG00000198521 | -3 | 1.88E-06 |
| *IRAG1* | Inositol 1,4,5-triphosphate receptor associated 1 | ENSG00000072952 | -2.99 | 5.78E-03 |
| *XIRP2* | Xin actin-binding repeat-containing protein 2 | ENSG00000163092 | -2.91 | 0.01 |
| *PLGLB2* | Plasminogen Like B2 | ENSG00000125551 | -2.87 | 0.01 |
| *NDN* | Necdin | ENSG00000182636 | -2.75 | 4.23E-04 |
| *ZNF568* | Zinc finger protein 568 | ENSG00000198453 | -2.75 | 3.89E-03 |
| *GPC6* | Glypican-6 | ENSG00000183098 | -2.7 | 0.03 |
| *ACAP1* | Arf-GAP with coiled-coil, ANK repeat and PH domain-containing protein 1 | ENSG00000072818 | -2.69 | 0.05 |
| *CELF2* | CUGBP Elav-like family member 2 | ENSG00000048740 | -2.57 | 1.70E-03 |
| *TICAM2* | TIR domain-containing adapter molecule 2 | ENSG00000243414 | -2.54 | 9.88E-03 |
| *HES7* | Transcription factor HES-7 | ENSG00000179111 | -2.49 | 7.36E-03 |
| *CYP27C1* | Cytochrome P450 27C1 | ENSG00000186684 | -2.43 | 0.02 |
| *CAPSL* | Calcyphosin-like protein | ENSG00000152611 | -2.4 | 3.93E-03 |
| *MASP1* | Mannan-binding lectin serine protease 1 | ENSG00000127241 | -2.39 | 0.04 |
| *TRPV2* | Transient receptor potential cation channel subfamily V member 2 | ENSG00000187688 | -2.38 | 0.04 |
| *TSPAN18* | Tetraspanin-18 | ENSG00000157570 | -2.35 | 5.53E-03 |
| *NGF* | Beta-nerve growth factor | ENSG00000134259 | -2.33 | 6.59E-05 |
| *KRT75* | Keratin, type II cytoskeletal 75 | ENSG00000170454 | -2.3 | 7.85E-05 |
| *LGALS7B* | Galectin 7B | ENSG00000178934 | -2.23 | 7.70E-04 |
| *ROBO4* | Roundabout homolog 4 | ENSG00000154133 | -2.18 | 3.71E-03 |
| *CEACAM6* | Carcinoembryonic antigen-related cell adhesion molecule 6 | ENSG00000086548 | -2.16 | 4.30E-09 |
| *NEURL3* | E3 ubiquitin-protein ligase NEURL3 | ENSG00000163121 | -2.13 | 0.02 |
| *CTXN1* | Cortexin-1 | ENSG00000178531 | -2.13 | 8.63E-04 |
| *TNC* | Tenascin | ENSG00000041982 | -1.88 | 3.99E-15 |
| *MSR1* | Macrophage scavenger receptor types I and II | ENSG00000038945 | -1.8 | 0.02 |
| *KRT81* | Keratin, type II cuticular Hb1 | ENSG00000205426 | -1.79 | 2.89E-04 |
| *ADRA1B* | Alpha-1B adrenergic receptor | ENSG00000170214 | -1.78 | 0.04 |
| *ELFN2_2* | Extracellular Leucine Rich Repeat And Fibronectin Type III Domain Containing 2 | ENSG00000166897 | -1.77 | 2.97E-07 |
| *VEGFC* | Vascular endothelial growth factor C | ENSG00000150630 | -1.73 | 8.82E-26 |
| *HAS2* | Hyaluronan synthase 2 | ENSG00000170961 | -1.72 | 8.34E-04 |
| *SV2A* | Synaptic vesicle glycoprotein 2A | ENSG00000159164 | -1.7 | 3.87E-03 |
| *SLC5A4* | Solute carrier family 5 member 4 | ENSG00000100191 | -1.67 | 0.03 |
| *RASD2* | GTP-binding protein Rhes | ENSG00000100302 | -1.67 | 0.03 |
| *INA* | Alpha-internexin | ENSG00000148798 | -1.65 | 0.02 |
| *ALOX5AP* | Arachidonate 5-lipoxygenase-activating protein | ENSG00000132965 | -1.57 | 0.04 |
| *PCSK1N* | ProSAAS | ENSG00000102109 | -1.55 | 1.42E-04 |
| *KCNQ2* | Potassium voltage-gated channel subfamily KQT member 2 | ENSG00000075043 | -1.53 | 0.05 |
| *FHL1* | Four and a half LIM domains protein 1 | ENSG00000022267 | -1.51 | 3.07E-11 |
| *MYL9* | Myosin regulatory light polypeptide 9 | ENSG00000101335 | -1.5 | 8.55E-10 |
| *C4orf36* | Chromosome 4 Open Reading Frame 36 | ENSG00000163633 | -1.5 | 2.43E-03 |
| *CCDC103* | Coiled-coil domain-containing protein 103 | ENSG00000167131 | -1.49 | 2.72E-03 |
| *SPOCK1* | Testican-1 | ENSG00000152377 | -1.48 | 8.91E-18 |
| *RASL10B* | Ras-like protein family member 10B | ENSG00000270885 | -1.46 | 0.05 |
| *ZFP82* | Zinc finger protein 82 homolog | ENSG00000181007 | -1.44 | 0.01 |
| *PTPRZ1* | Receptor-type tyrosine-protein phosphatase zeta | ENSG00000106278 | -1.44 | 0.02 |
| *ADTRP* | Androgen-dependent TFPI-regulating protein | ENSG00000111863 | -1.42 | 1.38E-09 |
| *KRT13* | Keratin, type I cytoskeletal 13 | ENSG00000171401 | -1.42 | 1.25E-04 |
| *NGEF* | Ephexin-1 | ENSG00000066248 | -1.41 | 2.71E-07 |
| *RBP1* | Retinol-binding protein 1 | ENSG00000114115 | -1.39 | 7.37E-03 |
| *AFF2* | AF4/FMR2 family member 2 | ENSG00000155966 | -1.38 | 0.01 |
| *TAFA2* | Chemokine-like protein TAFA-2 | ENSG00000198673 | -1.3 | 0.04 |
| *KRT16* | Keratin, type I cytoskeletal 16 | ENSG00000186832 | -1.29 | 1.37E-14 |
| *IL11* | Interleukin-11 | ENSG00000095752 | -1.28 | 0.03 |
| *RAB3B* | Ras-related protein Rab-3B | ENSG00000169213 | -1.27 | 4.17E-03 |
| *EGR3* | Early growth response protein 3 | ENSG00000179388 | -1.26 | 7.77E-13 |
| *NHS* | Nance-Horan syndrome protein | ENSG00000188158 | -1.26 | 2.19E-20 |
| *TRABD2A* | Metalloprotease TIKI1 | ENSG00000186854 | -1.24 | 3.68E-03 |
| *IL1RL1* | Interleukin-1 receptor-like 1 | ENSG00000115602 | -1.24 | 2.48E-10 |
| *BRSK1* | Serine/threonine-protein kinase BRSK1 | ENSG00000160469 | -1.24 | 0.03 |
| *MMP3* | Stromelysin-1 | ENSG00000149968 | -1.24 | 1.12E-03 |
| *SVIP* | Small VCP/p97-interacting protein | ENSG00000198168 | -1.23 | 0.02 |
| *NPBWR1* | Neuropeptides B/W receptor type 1 | ENSG00000288611 | -1.22 | 9.38E-03 |
| *HTR7* | 5-hydroxytryptamine receptor 7 | ENSG00000148680 | -1.22 | 4.36E-04 |
| *PHLDA1* | Pleckstrin homology-like domain family A member 1 | ENSG00000139289 | -1.2 | 2.81E-14 |
| *SLIT1* | Slit homolog 1 protein | ENSG00000187122 | -1.2 | 0.02 |
| *CORO2B* | Coronin-2B | ENSG00000103647 | -1.19 | 4.66E-03 |
| *ROR1* | Inactive tyrosine-protein kinase transmembrane receptor ROR1 | ENSG00000185483 | -1.19 | 0.01 |
| *MTSS1* | Protein MTSS 1 | ENSG00000170873 | -1.19 | 3.16E-15 |
| *SH2D5* | SH2 domain-containing protein 5 | ENSG00000189410 | -1.19 | 4.14E-04 |
| *SHANK2* | SH3 and multiple ankyrin repeat domains protein 2 | ENSG00000162105 | -1.16 | 4.83E-16 |
| *ST6GALNAC3* | Alpha-N-acetylgalactosaminide alpha-2,6-sialyltransferase 3 | ENSG00000184005 | -1.16 | 9.63E-03 |
| *TMEM151A* | Transmembrane protein 151A | ENSG00000179292 | -1.15 | 9.68E-06 |
| *SPRY4* | Protein sprouty homolog 4 | ENSG00000187678 | -1.15 | 1.78E-12 |
| *SPRED3* | Sprouty-related, EVH1 domain-containing protein 3 | ENSG00000188766 | -1.14 | 0.01 |
| *FLNC* | Filamin-C | ENSG00000128591 | -1.1 | 2.39E-03 |
| *KLF10* | Krueppel-like factor 10 | ENSG00000155090 | -1.1 | 2.79E-14 |
| *BAALC* | Brain and acute leukemia cytoplasmic protein | ENSG00000164929 | -1.1 | 0.03 |
| *NEURL1* | E3 ubiquitin-protein ligase NEURL1 | ENSG00000107954 | -1.09 | 9.15E-06 |
| *ADAMTS6* | A disintegrin and metalloproteinase with thrombospondin motifs 6 | ENSG00000049192 | -1.08 | 7.92E-05 |
| *GADD45A* | Growth arrest and DNA damage-inducible protein GADD45 alpha | ENSG00000116717 | -1.08 | 3.07E-11 |
| *PRR7* | Proline-rich protein 7 | ENSG00000131188 | -1.07 | 6.82E-05 |
| *FOXD1* | Forkhead box protein D1 | ENSG00000251493 | -1.07 | 4.02E-06 |
| *PGF* | Placenta growth factor | ENSG00000119630 | -1.06 | 1.71E-03 |
| *ETS1* | Protein C-ets-1 | ENSG00000134954 | -1.05 | 2.79E-22 |
| *DUSP4* | Dual specificity protein phosphatase 4 | ENSG00000120875 | -1.04 | 2.86E-10 |
| *TIMP4* | Metalloproteinase inhibitor 4 | ENSG00000157150 | -1.04 | 3.14E-03 |
| *C1orf116* | Chromosome 1 Open Reading Frame 116 | ENSG00000182795 | -1.04 | 8.96E-22 |
| *FOSL1* | Fos-related antigen 1 | ENSG00000175592 | -1.03 | 3.18E-08 |
| *SYT7* | Synaptotagmin-7 | ENSG00000011347 | -1.03 | 5.68E-14 |
| *CSRNP1* | Cysteine/serine-rich nuclear protein 1 | ENSG00000144655 | -1.03 | 8.30E-07 |
| *COL12A1* | Collagen alpha-1(XII) chain | ENSG00000111799 | -1.03 | 9.73E-21 |
| *ADAMTS1* | A disintegrin and metalloproteinase with thrombospondin motifs 1 | ENSG00000154734 | -1.02 | 0.02 |
| *DNER* | Delta and Notch-like epidermal growth factor-related receptor | ENSG00000187957 | -1.02 | 0.02 |
| *ASIC1* | Acid-sensing ion channel 1 | ENSG00000110881 | -1.02 | 0.01 |
| *PXDN* | Peroxidasin homolog | ENSG00000130508 | -1.01 | 1.36E-04 |
| *FCMR* | Fas apoptotic inhibitory molecule 3 | ENSG00000162894 | -1.01 | 1.72E-05 |
| *NCF2* | Neutrophil cytosol factor 2 | ENSG00000116701 | -1.01 | 3.74E-05 |
| *CPA4* | Carboxypeptidase A4 | ENSG00000128510 | -1.01 | 0.04 |
| *FAM131C* | Protein FAM131C | ENSG00000185519 | -1.01 | 7.24E-03 |
