# Supplementary material for: EMILIN-1 Suppresses Cell Proliferation through Altered Cell Cycle Regulation in Head and Neck Squamous Cell Carcinoma
Source: Am J Pathol. 2025 Jan 30;195(5):995–1012. doi: 10.1016/j.ajpath.2025.01.010 (PMC12163418; doi:10.1016/j.ajpath.2025.01.010)
Supplement: Supplemental Table S3 [file mmc3.docx]

| **Supplemental Table S3** Upregulated genes of CAL27 cell line with EMILIN-1 overexpression (Log2FC>1,FDR<0.05). (https://www.ensembl.org) | | | | |
| --- | --- | --- | --- | --- |
|  |  |  |  |  |
| **Gene** | **Database name** | **Identifier** | **Log2FC** | **FDR p-value** |
| *EMILIN1* | Elastin microfibril interfacer 1 | ENSG00000138080 | 14.23 | 0 |
| *BLOC1S5-TXNDC5* | BLOC1S5-TXNDC5 readthrough (NMD candidate) (Fragment) | ENSG00000259040 | 10.64 | 7.37E-05 |
| *PAPPA2* | Pappalysin-2 | ENSG00000116183 | 8.84 | 8.45E-04 |
| *PRPH* | Peripherin | ENSG00000135406 | 8.03 | 2.74E-03 |
| *CCDC201* | Coiled-Coil Domain Containing 201 | ENSG00000283247 | 7.53 | 5.46E-03 |
| *PRSS53* | Serine protease 53 | ENSG00000151006 | 7.39 | 0.01 |
| *DEFB4A* | Beta-defensin 4A | ENSG00000171711 | 7.21 | 1.03E-29 |
| *PRSS1* | Trypsin-1 | ENSG00000204983 | 7.19 | 8.62E-03 |
| *OLAH* | S-acyl fatty acid synthase thioesterase, medium chain | ENSG00000152463 | 7.16 | 2.26E-20 |
| *DRC1* | Dynein regulatory complex protein 1 | ENSG00000157856 | 6.69 | 0.02 |
| *CDR1* | Cerebellar degeneration-related antigen 1 | ENSG00000288642 | 6.66 | 0.02 |
| *FAM47E-STBD1* | family with sequence similarity 47, member E | ENSG00000272414 | 6.57 | 0.02 |
| *LINGO2* | Leucine-rich repeat and immunoglobulin-like domain-containing nogo receptor-interacting protein 2 | ENSG00000174482 | 6.39 | 0.03 |
| *P3R3URF-PIK3R3* | P3R3URF-PIK3R3 Readthrough | ENSG00000278139 | 6.34 | 0.03 |
| *EVX1* | Homeobox even-skipped homolog protein 1 | ENSG00000106038 | 6.26 | 0.03 |
| *NRCAM* | Neuronal cell adhesion molecule | ENSG00000091129 | 6.15 | 0.03 |
| *B4GALNT2* | Beta-1,4 N-acetylgalactosaminyltransferase 2 | ENSG00000167080 | 6.15 | 2.03E-14 |
| *VIP* | VIP peptides | ENSG00000146469 | 5.98 | 0.04 |
| *PNPLA1* | Omega-hydroxyceramide transacylase | ENSG00000180316 | 5.91 | 0.04 |
| *IL1RL1* | Interleukin-1 receptor-like 1 | ENSG00000115602 | 5.87 | 5.73E-18 |
| *BARX1* | Homeobox protein BarH-like 1 | ENSG00000131668 | 5.85 | 0.04 |
| *LCE1F* | Late cornified envelope protein 1F | ENSG00000240386 | 5.78 | 0.04 |
| *APOD* | Apolipoprotein D | ENSG00000189058 | 5.78 | 0.04 |
| *NWD2* | NACHT and WD repeat domain containing 2 | ENSG00000174145 | 5.77 | 9.87E-08 |
| *ANKRD55* | Ankyrin repeat domain-containing protein 55 | ENSG00000164512 | 5.71 | 0.05 |
| *MAP1B* | Microtubule-associated protein 1B | ENSG00000131711 | 5.64 | 2.09E-273 |
| *CLGN* | Calmegin | ENSG00000153132 | 5.59 | 2.67E-177 |
| *FCGBP* | IgGFc-binding protein | ENSG00000275395 | 5.56 | 6.44E-269 |
| *INHBE* | Inhibin beta E chain | ENSG00000139269 | 5.49 | 1.10E-155 |
| *SV2B* | Synaptic vesicle glycoprotein 2B | ENSG00000185518 | 5.43 | 8.52E-13 |
| *SLC5A1* | Sodium/glucose cotransporter 1 | ENSG00000100170 | 5.41 | 6.57E-11 |
| *VNN3* | Vascular non-inflammatory molecule 3 | ENSG00000093134 | 5.34 | 3.36E-117 |
| *BHLHA15* | Class A basic helix-loop-helix protein 15 | ENSG00000180535 | 4.94 | 1.32E-305 |
| *IGFBP1* | Insulin-like growth factor-binding protein 1 | ENSG00000146678 | 4.93 | 2.32E-95 |
| *IRF4* | Interferon regulatory factor 4 | ENSG00000137265 | 4.86 | 2.93E-08 |
| *C11orf96* | Chromosome 11 Open Reading Frame 96 | ENSG00000187479 | 4.86 | 7.75E-07 |
| *FGF21* | Fibroblast growth factor 21 | ENSG00000105550 | 4.76 | 4.95E-05 |
| *AGR2* | Anterior gradient protein 2 homolog | ENSG00000106541 | 4.69 | 0 |
| *IL12B* | Interleukin-12 subunit beta | ENSG00000113302 | 4.65 | 6.72E-05 |
| *IL13RA2* | Interleukin-13 receptor subunit alpha-2 | ENSG00000123496 | 4.63 | 1.41E-07 |
| *SPRR2D* | Small proline-rich protein 2D | ENSG00000163216 | 4.55 | 1.01E-24 |
| *PECAM1* | Platelet endothelial cell adhesion molecule | ENSG00000261371 | 4.41 | 2.49E-04 |
| *MOV10L1* | RNA helicase Mov10l1 | ENSG00000073146 | 4.41 | 3.56E-83 |
| *LURAP1L* | Leucine rich adaptor protein 1-like | ENSG00000153714 | 4.37 | 6.24E-278 |
| *ASDURF* | ASNSD1 upstream open reading frame protein | ENSG00000286053 | 4.35 | 6.44E-05 |
| *KLHDC7B* | Kelch domain-containing protein 7B | ENSG00000130487 | 4.26 | 1.45E-197 |
| *BPIFC* | BPI fold-containing family C protein | ENSG00000184459 | 4.18 | 7.47E-05 |
| *CMKLR2* | Chemerin-like receptor 2 | ENSG00000183671 | 4.14 | 6.91E-71 |
| *CCNA1* | Cyclin-A1 | ENSG00000133101 | 4.12 | 5.24E-47 |
| *MKX* | Homeobox protein Mohawk | ENSG00000150051 | 4.12 | 3.23E-05 |
| *EPS8L3* | Epidermal growth factor receptor kinase substrate 8-like protein 3 | ENSG00000198758 | 4.1 | 1.53E-09 |
| *RGSL1* | Regulator of G-protein signaling protein-like | ENSG00000121446 | 4.01 | 0.01 |
| *SYN2* | Synapsin-2 | ENSG00000157152 | 3.95 | 2.02E-03 |
| *FAM187A* | Ig-like V-type domain-containing protein FAM187A | ENSG00000214447 | 3.94 | 7.01E-04 |
| *C3AR1* | C3a anaphylatoxin chemotactic receptor | ENSG00000171860 | 3.9 | 1.51E-06 |
| *SMOC1* | SPARC-related modular calcium-binding protein 1 | ENSG00000198732 | 3.87 | 0.02 |
| *TREM1* | Triggering receptor expressed on myeloid cells 1 | ENSG00000124731 | 3.77 | 5.84E-49 |
| *HID1* | Protein HID1 | ENSG00000167861 | 3.76 | 3.07E-234 |
| *ZFP57* | Zinc finger protein 57 homolog | ENSG00000204644 | 3.71 | 8.54E-05 |
| *GLB1L3* | Beta-galactosidase-1-like protein 3 | ENSG00000166105 | 3.68 | 9.83E-06 |
| *ATP6V0D2* | V-type proton ATPase subunit d 2 | ENSG00000147614 | 3.68 | 2.22E-09 |
| *ISLR2* | Immunoglobulin superfamily containing leucine-rich repeat protein 2 | ENSG00000167178 | 3.67 | 5.01E-16 |
| *CREB5* | Cyclic AMP-responsive element-binding protein 5 | ENSG00000146592 | 3.66 | 3.14E-88 |
| *SHH* | Sonic hedgehog protein | ENSG00000164690 | 3.62 | 4.89E-13 |
| *SOBP* | Sine oculis-binding protein homolog | ENSG00000112320 | 3.6 | 0.01 |
| *SEMA3B* | Semaphorin-3B | ENSG00000012171 | 3.6 | 4.35E-104 |
| *ALOX5AP* | Arachidonate 5-lipoxygenase-activating protein | ENSG00000132965 | 3.59 | 4.85E-49 |
| *LCN2* | Neutrophil gelatinase-associated lipocalin | ENSG00000148346 | 3.56 | 5.98E-194 |
| *SLC22A31* | Putative solute carrier family 22 member 31 | ENSG00000259803 | 3.56 | 0.03 |
| *KCNK15* | Potassium channel subfamily K member 15 | ENSG00000124249 | 3.55 | 1.58E-20 |
| *RGPD4* | RanBP2-like and GRIP domain-containing protein 4 | ENSG00000196862 | 3.55 | 3.18E-03 |
| *CHI3L2* | Chitinase-3-like protein 2 | ENSG00000064886 | 3.54 | 5.34E-15 |
| *H1-6* | Histone H1t | ENSG00000187475 | 3.53 | 5.78E-04 |
| *RUBCNL* | Protein associated with UVRAG as autophagy enhancer | ENSG00000102445 | 3.52 | 4.17E-18 |
| *LHX3* | LIM/homeobox protein Lhx3 | ENSG00000107187 | 3.5 | 9.30E-03 |
| *TNFSF18* | Tumor necrosis factor ligand superfamily member 18 | ENSG00000120337 | 3.5 | 2.29E-32 |
| *MUC15* | Mucin-15 | ENSG00000169550 | 3.5 | 3.28E-04 |
| *CST7* | Cystatin-F | ENSG00000077984 | 3.49 | 1.55E-04 |
| *DACT1* | Dapper homolog 1 | ENSG00000165617 | 3.47 | 7.85E-04 |
| *DCLK1* | Serine/threonine-protein kinase DCLK1 | ENSG00000133083 | 3.45 | 2.14E-17 |
| *CASP5* | Caspase-5 | ENSG00000137757 | 3.44 | 1.86E-50 |
| *FER1L6* | Fer-1-like protein 6 | ENSG00000214814 | 3.44 | 5.50E-10 |
| *CCNP* | Cyclin-P | ENSG00000105219 | 3.41 | 1.26E-17 |
| *GDF15* | Growth/differentiation factor 15 | ENSG00000130513 | 3.38 | 1.28E-164 |
| *S100P* | Protein S100-P | ENSG00000163993 | 3.35 | 9.49E-67 |
| *TIMP4* | Metalloproteinase inhibitor 4 | ENSG00000157150 | 3.29 | 7.77E-19 |
| *ADM2* | Protein ADM2 | ENSG00000128165 | 3.26 | 1.65E-167 |
| *EPGN* | Epigen | ENSG00000182585 | 3.25 | 1.79E-06 |
| *RXFP4* | Relaxin-3 receptor 2 | ENSG00000173080 | 3.25 | 2.01E-03 |
| *PRMT8* | Protein arginine N-methyltransferase 8 | ENSG00000111218 | 3.23 | 5.15E-04 |
| *LPO* | Lactoperoxidase | ENSG00000167419 | 3.22 | 3.54E-08 |
| *BEX2* | Protein BEX2 | ENSG00000133134 | 3.21 | 4.44E-109 |
| *FOXA2* | Hepatocyte nuclear factor 3-beta | ENSG00000125798 | 3.19 | 4.05E-53 |
| *SH3RF3* | E3 ubiquitin-protein ligase SH3RF3 | ENSG00000172985 | 3.19 | 9.01E-11 |
| *C15orf65* | Uncharacterized protein C15orf65 | ENSG00000261652 | 3.17 | 3.01E-29 |
| *TRIB3* | Tribbles homolog 3 | ENSG00000101255 | 3.16 | 4.99E-214 |
| *GDAP1L1* | Ganglioside-induced differentiation-associated protein 1-like 1 | ENSG00000124194 | 3.15 | 0.03 |
| *SYNE1* | Nesprin-1 | ENSG00000131018 | 3.15 | 7.27E-172 |
| *NECAB2* | N-terminal EF-hand calcium-binding protein 2 | ENSG00000103154 | 3.13 | 1.25E-17 |
| *NTNG1* | Netrin-G1 | ENSG00000162631 | 3.12 | 1.65E-51 |
| *PIGR* | Polymeric immunoglobulin receptor | ENSG00000162896 | 3.12 | 1.01E-198 |
| *MSC* | Musculin | ENSG00000178860 | 3.1 | 1.46E-53 |
| *HSPA5* | Endoplasmic reticulum chaperone BiP | ENSG00000044574 | 3.1 | 4.63E-208 |
| *FOXD4L5* | Forkhead box protein D4-like 5 | ENSG00000204779 | 3.08 | 0.03 |
| *RNASE7* | Ribonuclease 7 | ENSG00000165799 | 3.07 | 2.11E-189 |
| *AREG* | Amphiregulin | ENSG00000109321 | 3.07 | 3.64E-188 |
| *HBEGF* | Proheparin-binding EGF-like growth factor | ENSG00000113070 | 3.06 | 3.44E-106 |
| *GCNT3* | Beta-1,3-galactosyl-O-glycosyl-glycoprotein beta-1,6-N-acetylglucosaminyltransferase 3 | ENSG00000140297 | 3.05 | 2.76E-126 |
| *B3GALT5* | Beta-1,3-galactosyltransferase 5 | ENSG00000183778 | 3.03 | 7.76E-09 |
| *HKDC1* | Hexokinase HKDC1 | ENSG00000156510 | 3.02 | 3.31E-19 |
| *H3C13* | Histone H3.2 | ENSG00000183598 | 3.02 | 1.58E-04 |
| *H2BU1* | Histone H2B type 3-B | ENSG00000196890 | 3.02 | 1.63E-44 |
| *LAMP3* | Lysosome-associated membrane glycoprotein 3 | ENSG00000078081 | 3.01 | 5.77E-190 |
| *LRRC55* | Leucine-rich repeat-containing protein 55 | ENSG00000183908 | 3 | 4.95E-27 |
| *CHRNA9* | Neuronal acetylcholine receptor subunit alpha-9 | ENSG00000174343 | 3 | 0.04 |
| *SPP1* | Osteopontin | ENSG00000118785 | 3 | 9.91E-09 |
| *CYP4F3* | Cytochrome P450 4F3 | ENSG00000186529 | 3 | 1.66E-12 |
| *SH3BGR* | SH3 domain-binding glutamic acid-rich protein | ENSG00000185437 | 2.99 | 4.59E-19 |
| *ABCG1* | ATP-binding cassette sub-family G member 1 | ENSG00000160179 | 2.97 | 1.78E-160 |
| *ADAMTS4* | A disintegrin and metalloproteinase with thrombospondin motifs 4 | ENSG00000158859 | 2.97 | 1.02E-05 |
| *CDH15* | Cadherin-15 | ENSG00000129910 | 2.96 | 3.09E-05 |
| *MUC2* | Mucin-2 | ENSG00000198788 | 2.96 | 5.94E-16 |
| *CHI3L1* | Chitinase-3-like protein 1 | ENSG00000133048 | 2.96 | 9.13E-04 |
| *KRTAP5-1* | Keratin-associated protein 5-1 | ENSG00000205869 | 2.95 | 8.89E-03 |
| *SESN2* | Sestrin-2 | ENSG00000130766 | 2.93 | 3.03E-176 |
| *SCN9A* | Sodium channel protein type 9 subunit alpha | ENSG00000169432 | 2.93 | 2.87E-16 |
| *DNAJB9* | DnaJ homolog subfamily B member 9 | ENSG00000128590 | 2.92 | 6.69E-171 |
| *H3-2* | Histone HIST2H3PS2 | ENSG00000273213 | 2.91 | 2.27E-13 |
| *VNN2* | Vascular non-inflammatory molecule 2 | ENSG00000112303 | 2.91 | 5.08E-17 |
| *H2AC8* | H2A Clustered Histone 8 | ENSG00000277075 | 2.91 | 3.02E-15 |
| *OR10S1* | Olfactory receptor 10S1 | ENSG00000196248 | 2.89 | 1.37E-03 |
| *CHAC1* | Glutathione-specific gamma-glutamylcyclotransferase 1 | ENSG00000128965 | 2.89 | 8.85E-158 |
| *NCR3LG1* | Natural cytotoxicity triggering receptor 3 ligand 1 | ENSG00000188211 | 2.88 | 3.20E-34 |
| *ABCA6* | ATP-binding cassette sub-family A member 6 | ENSG00000154262 | 2.86 | 2.91E-07 |
| *IL1RN* | Interleukin-1 receptor antagonist protein | ENSG00000136689 | 2.84 | 1.27E-162 |
| *DNAJC12* | DnaJ homolog subfamily C member 12 | ENSG00000108176 | 2.83 | 1.23E-09 |
| *KIF21B* | Kinesin-like protein KIF21B | ENSG00000116852 | 2.83 | 4.30E-81 |
| *STC2* | Stanniocalcin-2 | ENSG00000113739 | 2.83 | 4.49E-171 |
| *CPXM1* | Probable carboxypeptidase X1 | ENSG00000088882 | 2.83 | 0.03 |
| *FYN* | Tyrosine-protein kinase Fyn | ENSG00000010810 | 2.82 | 3.35E-32 |
| *PDE10A* | cAMP and cAMP-inhibited cGMP 3',5'-cyclic phosphodiesterase 10A | ENSG00000112541 | 2.81 | 9.93E-05 |
| *CAPN13* | Calpain-13 | ENSG00000162949 | 2.81 | 0.02 |
| *FUT6* | 4-galactosyl-N-acetylglucosaminide 3-alpha-L-fucosyltransferase FUT6 | ENSG00000156413 | 2.81 | 3.28E-08 |
| *NUPR1* | Nuclear protein 1 | ENSG00000176046 | 2.81 | 5.00E-89 |
| *FUT3* | 3-galactosyl-N-acetylglucosaminide 4-alpha-L-fucosyltransferase FUT3 | ENSG00000171124 | 2.8 | 4.26E-156 |
| *CALML6* | Calmodulin-like protein 6 | ENSG00000169885 | 2.8 | 5.59E-06 |
| *PCDHA4* | Protocadherin alpha-4 | ENSG00000204967 | 2.78 | 0.03 |
| *ARHGAP9* | Rho GTPase-activating protein 9 | ENSG00000123329 | 2.77 | 9.09E-04 |
| *NCF2* | Neutrophil cytosol factor 2 | ENSG00000116701 | 2.75 | 6.17E-140 |
| *ID4* | DNA-binding protein inhibitor ID-4 | ENSG00000172201 | 2.74 | 2.94E-06 |
| *IL36A* | Interleukin-36 alpha | ENSG00000136694 | 2.73 | 4.42E-04 |
| *STXBP5L* | Syntaxin-binding protein 5-like | ENSG00000145087 | 2.73 | 0.04 |
| *SLC29A4* | Equilibrative nucleoside transporter 4 | ENSG00000164638 | 2.72 | 1.39E-47 |
| *KCNH2* | Potassium voltage-gated channel subfamily H member 2 | ENSG00000055118 | 2.72 | 0.01 |
| *ERBB4* | Receptor tyrosine-protein kinase erbB-4 | ENSG00000178568 | 2.72 | 0.03 |
| *PCDHAC2* | Protocadherin alpha-C2 | ENSG00000243232 | 2.71 | 3.19E-22 |
| *PNLIPRP3* | Pancreatic lipase-related protein 3 | ENSG00000203837 | 2.71 | 1.73E-52 |
| *RP1* | Oxygen-regulated protein 1 | ENSG00000104237 | 2.71 | 3.75E-04 |
| *ZMAT1* | Zinc finger matrin-type protein 1 | ENSG00000166432 | 2.7 | 0.03 |
| *HERPUD1* | Homocysteine-responsive endoplasmic reticulum-resident ubiquitin-like domain member 1 protein | ENSG00000051108 | 2.7 | 0 |
| *DDIT3* | DNA damage-inducible transcript 3 protein | ENSG00000175197 | 2.69 | 2.97E-150 |
| *EID3* | EP300-interacting inhibitor of differentiation 3 | ENSG00000255150 | 2.67 | 6.26E-12 |
| *SCN3B* | Sodium channel subunit beta-3 | ENSG00000166257 | 2.67 | 1.81E-04 |
| *CELF6* | CUGBP Elav-like family member 6 | ENSG00000140488 | 2.66 | 0.03 |
| *ANKK1* | Ankyrin repeat and protein kinase domain-containing protein 1 | ENSG00000170209 | 2.65 | 2.42E-15 |
| *ERICH2* | Glutamate-rich protein 2 | ENSG00000204334 | 2.64 | 6.21E-10 |
| *ADGRA1* | Adhesion G protein-coupled receptor A1 | ENSG00000197177 | 2.64 | 0.03 |
| *CCPG1* | Cell cycle progression protein 1 | ENSG00000260916 | 2.63 | 6.69E-148 |
| *ROS1* | Proto-oncogene tyrosine-protein kinase ROS | ENSG00000047936 | 2.63 | 6.01E-16 |
| *RORC* | Nuclear receptor ROR-gamma | ENSG00000143365 | 2.62 | 1.07E-03 |
| *CLCA4* | Calcium-activated chloride channel regulator 4 | ENSG00000016602 | 2.61 | 9.68E-11 |
| *AKR1C2* | Aldo-keto reductase family 1 member C2 | ENSG00000151632 | 2.6 | 1.45E-135 |
| *THSD7A* | Thrombospondin type-1 domain-containing protein 7A | ENSG00000005108 | 2.6 | 3.96E-04 |
| *TSLP* | Thymic stromal lymphopoietin | ENSG00000145777 | 2.6 | 9.70E-37 |
| *ENPP2* | Ectonucleotide pyrophosphatase/phosphodiesterase family member 2 | ENSG00000136960 | 2.6 | 8.34E-03 |
| *SLC10A5* | Sodium/bile acid cotransporter 5 | ENSG00000253598 | 2.59 | 4.53E-03 |
| *CST2* | Cystatin-SA | ENSG00000170369 | 2.59 | 0.02 |
| *RBCK1* | RanBP-type and C3HC4-type zinc finger-containing protein 1 | ENSG00000125826 | 2.59 | 8.45E-145 |
| *ST6GALNAC3* | Alpha-N-acetylgalactosaminide alpha-2,6-sialyltransferase 3 | ENSG00000184005 | 2.59 | 3.33E-10 |
| *MEIS3* | Homeobox protein Meis3 | ENSG00000105419 | 2.59 | 3.46E-04 |
| *ABCA12* | Glucosylceramide transporter ABCA12 | ENSG00000144452 | 2.58 | 1.44E-131 |
| *MASP1* | Mannan-binding lectin serine protease 1 | ENSG00000127241 | 2.57 | 0.05 |
| *C12orf71* | Chromosome 12 Open Reading Frame 71 | ENSG00000214700 | 2.56 | 0.01 |
| *ULBP1* | UL16-binding protein 1 | ENSG00000111981 | 2.54 | 9.81E-57 |
| *ETV5* | ETS translocation variant 5 | ENSG00000244405 | 2.53 | 8.78E-119 |
| *FCGR1B* | High affinity immunoglobulin gamma Fc receptor IB | ENSG00000198019 | 2.52 | 9.16E-03 |
| *TCIM* | Transcriptional and immune response regulator | ENSG00000176907 | 2.52 | 6.79E-38 |
| *SLC7A11* | Cystine/glutamate transporter | ENSG00000151012 | 2.52 | 8.20E-133 |
| *ST8SIA6* | Alpha-2,8-sialyltransferase 8F | ENSG00000148488 | 2.51 | 1.69E-17 |
| *ERFL* | ETS domain-containing transcription factor ERF-like | ENSG00000268041 | 2.51 | 0.03 |
| *PLAC1* | Placenta-specific protein 1 | ENSG00000170965 | 2.51 | 2.84E-11 |
| *IL34* | Interleukin-34 | ENSG00000157368 | 2.51 | 5.97E-10 |
| *CD22* | B-cell receptor CD22 | ENSG00000012124 | 2.51 | 2.05E-13 |
| *ARHGEF2* | Rho guanine nucleotide exchange factor 2 | ENSG00000116584 | 2.5 | 9.88E-130 |
| *SLC43A1* | Large neutral amino acids transporter small subunit 3 | ENSG00000149150 | 2.5 | 5.05E-38 |
| *SPTSSB* | Serine palmitoyltransferase small subunit B | ENSG00000196542 | 2.5 | 4.20E-19 |
| *PLEKHG1* | Pleckstrin homology domain-containing family G member 1 | ENSG00000120278 | 2.5 | 6.94E-20 |
| *PARM1* | Prostate androgen-regulated mucin-like protein 1 | ENSG00000169116 | 2.49 | 1.97E-41 |
| *ADAM23* | Disintegrin and metalloproteinase domain-containing protein 23 | ENSG00000114948 | 2.49 | 2.54E-10 |
| *VEGFA* | Vascular endothelial growth factor A | ENSG00000112715 | 2.48 | 9.26E-133 |
| *SCEL* | Sciellin | ENSG00000136155 | 2.47 | 1.37E-72 |
| *SMIM24* | Small integral membrane protein 24 | ENSG00000095932 | 2.47 | 0.02 |
| *CATSPERB* | Cation channel sperm-associated protein subunit beta | ENSG00000133962 | 2.47 | 8.61E-08 |
| *UNC5B* | Netrin receptor UNC5B | ENSG00000107731 | 2.47 | 1.50E-290 |
| *GALNT13* | Polypeptide N-acetylgalactosaminyltransferase 13 | ENSG00000144278 | 2.46 | 0.03 |
| *TEX37* | Testis-expressed sequence 37 protein | ENSG00000172073 | 2.46 | 4.07E-05 |
| *LARP6* | La-related protein 6 | ENSG00000166173 | 2.46 | 1.11E-235 |
| *FXYD1* | Phospholemman | ENSG00000266964 | 2.45 | 0.03 |
| *SPX* | Spexin | ENSG00000134548 | 2.45 | 0.03 |
| *H2BC7* | H2B Clustered Histone 7 | ENSG00000277224 | 2.44 | 6.33E-10 |
| *BAMBI* | BMP and activin membrane-bound inhibitor homolog | ENSG00000095739 | 2.44 | 1.72E-03 |
| *CD55* | Complement decay-accelerating factor | ENSG00000196352 | 2.42 | 1.31E-122 |
| *BEX4* | Protein BEX4 | ENSG00000102409 | 2.42 | 5.00E-20 |
| *H2BC18* | Histone H2B type 2-F | ENSG00000203814 | 2.42 | 1.52E-14 |
| *KRT80* | Keratin, type II cytoskeletal 80 | ENSG00000167767 | 2.41 | 3.58E-125 |
| *SAMD5* | Sterile alpha motif domain-containing protein 5 | ENSG00000203727 | 2.41 | 2.73E-03 |
| *NEURL1* | E3 ubiquitin-protein ligase NEURL1 | ENSG00000107954 | 2.39 | 3.90E-09 |
| *EBI3* | Interleukin-27 subunit beta | ENSG00000105246 | 2.38 | 1.10E-22 |
| *H2BC4* | H2B Clustered Histone 4 | ENSG00000180596 | 2.38 | 1.11E-49 |
| *AKR1D1* | Aldo-keto reductase family 1 member D1 | ENSG00000122787 | 2.38 | 5.75E-04 |
| *KRT23* | Keratin, type I cytoskeletal 23 | ENSG00000108244 | 2.38 | 7.22E-15 |
| *ERN1* | Serine/threonine-protein kinase/endoribonuclease IRE1 | ENSG00000178607 | 2.37 | 1.68E-118 |
| *H1-3* | Histone H1.3 | ENSG00000124575 | 2.37 | 1.91E-09 |
| *H2BC21* | Histone H2B type 2-E | ENSG00000184678 | 2.37 | 1.37E-87 |
| *H2AC6* | Histone H2A type 1-C | ENSG00000180573 | 2.37 | 1.22E-116 |
| *ODAD2* | Outer dynein arm-docking complex subunit 2 | ENSG00000169126 | 2.37 | 0.03 |
| *HSD17B2* | 17-beta-hydroxysteroid dehydrogenase type 2 | ENSG00000086696 | 2.36 | 1.02E-12 |
| *ATP1A2* | Sodium/potassium-transporting ATPase subunit alpha-2 | ENSG00000018625 | 2.36 | 1.98E-03 |
| *CCDC169-SOHLH2* | CCDC169-SOHLH2 readthrough | ENSG00000250709 | 2.36 | 9.63E-03 |
| *TIMP3* | Metalloproteinase inhibitor 3 | ENSG00000100234 | 2.35 | 3.01E-106 |
| *STRA6* | Receptor for retinol uptake STRA6 | ENSG00000137868 | 2.35 | 8.02E-107 |
| *PAEP* | Glycodelin | ENSG00000122133 | 2.35 | 0.01 |
| *ECM2* | Extracellular matrix protein 2 | ENSG00000106823 | 2.35 | 1.11E-10 |
| *DDR2* | Discoidin domain-containing receptor 2 | ENSG00000162733 | 2.34 | 2.55E-06 |
| *BEGAIN* | Brain-enriched guanylate kinase-associated protein | ENSG00000183092 | 2.34 | 4.36E-04 |
| *ALDH1L2* | Mitochondrial 10-formyltetrahydrofolate dehydrogenase | ENSG00000136010 | 2.33 | 4.28E-254 |
| *KRT7* | Keratin, type II cytoskeletal 7 | ENSG00000135480 | 2.32 | 4.67E-40 |
| *ATP13A4* | Probable cation-transporting ATPase 13A4 | ENSG00000127249 | 2.32 | 1.59E-07 |
| *H2BC8* | H2B Clustered Histone 8 | ENSG00000273802 | 2.31 | 1.08E-17 |
| *PCDH12* | Protocadherin-12 | ENSG00000113555 | 2.31 | 9.18E-09 |
| *THBD* | Thrombomodulin | ENSG00000178726 | 2.31 | 7.15E-96 |
| *FLRT1* | Leucine-rich repeat transmembrane protein FLRT1 | ENSG00000126500 | 2.31 | 6.85E-23 |
| *PRSS3* | Trypsin-3 | ENSG00000010438 | 2.3 | 2.61E-06 |
| *FICD* | Protein adenylyltransferase FICD | ENSG00000198855 | 2.29 | 6.06E-87 |
| *ARMC3* | Armadillo repeat-containing protein 3 | ENSG00000165309 | 2.29 | 2.85E-06 |
| *PTX3* | Pentraxin-related protein PTX3 | ENSG00000163661 | 2.29 | 6.43E-47 |
| *SLC4A9* | Anion exchange protein 4 | ENSG00000113073 | 2.28 | 0.01 |
| *PLCH2* | 1-phosphatidylinositol 4,5-bisphosphate phosphodiesterase eta-2 | ENSG00000149527 | 2.27 | 7.74E-40 |
| *HOXD1* | Homeobox protein Hox-D1 | ENSG00000128645 | 2.27 | 1.60E-25 |
| *ITGAX* | Integrin alpha-X | ENSG00000140678 | 2.27 | 2.70E-08 |
| *RADIL* | Ras-associating and dilute domain-containing protein | ENSG00000157927 | 2.26 | 0.05 |
| *C10orf90* | (E2-independent) E3 ubiquitin-conjugating enzyme FATS | ENSG00000154493 | 2.24 | 0.01 |
| *WARS1* | Tryptophan--tRNA ligase, cytoplasmic | ENSG00000140105 | 2.24 | 1.11E-108 |
| *CSF3* | Granulocyte colony-stimulating factor | ENSG00000108342 | 2.24 | 8.24E-38 |
| *PCK2* | Phosphoenolpyruvate carboxykinase [GTP], mitochondrial | ENSG00000100889 | 2.23 | 1.57E-227 |
| *BCL2A1* | Bcl-2-related protein A1 | ENSG00000140379 | 2.23 | 1.95E-14 |
| *H2AC19* | H2A Clustered Histone 19 | ENSG00000288859 | 2.22 | 2.13E-05 |
| *CSGALNACT1* | Chondroitin sulfate N-acetylgalactosaminyltransferase 1 | ENSG00000147408 | 2.21 | 1.01E-86 |
| *GOLGA6L2* | Golgin A6 Family Like 2 | ENSG00000174450 | 2.21 | 0.04 |
| *KCNT1* | Potassium channel subfamily T member 1 | ENSG00000107147 | 2.21 | 9.04E-07 |
| *COL21A1* | Collagen alpha-1(XXI) chain | ENSG00000124749 | 2.21 | 4.36E-32 |
| *P2RX7* | P2X purinoceptor 7 | ENSG00000089041 | 2.2 | 9.84E-93 |
| *TYRP1* | 5,6-dihydroxyindole-2-carboxylic acid oxidase | ENSG00000107165 | 2.2 | 0.01 |
| *ART3* | Ecto-ADP-ribosyltransferase 3 | ENSG00000156219 | 2.19 | 0.03 |
| *H4C8* | H4 Clustered Histone 8 | ENSG00000158406 | 2.19 | 2.72E-33 |
| *GFPT1* | Glutamine--fructose-6-phosphate aminotransferase [isomerizing] 1 | ENSG00000198380 | 2.19 | 3.06E-103 |
| *CTHRC1* | Collagen triple helix repeat-containing protein 1 | ENSG00000164932 | 2.19 | 1.30E-39 |
| *GRB10* | Growth factor receptor-bound protein 10 | ENSG00000106070 | 2.18 | 3.19E-93 |
| *NAMPT* | Nicotinamide phosphoribosyltransferase | ENSG00000105835 | 2.18 | 3.06E-103 |
| *PAPPA* | Pappalysin-1 | ENSG00000182752 | 2.18 | 3.28E-40 |
| *PTPRH* | Receptor-type tyrosine-protein phosphatase H | ENSG00000080031 | 2.17 | 1.26E-52 |
| *ADAM19* | Disintegrin and metalloproteinase domain-containing protein 19 | ENSG00000135074 | 2.17 | 1.92E-57 |
| *ATG9B* | Autophagy-related protein 9B | ENSG00000181652 | 2.16 | 1.21E-06 |
| *MAP1LC3B* | Microtubule-associated proteins 1A/1B light chain 3B | ENSG00000140941 | 2.15 | 5.43E-99 |
| *CCDC149* | Coiled-Coil Domain Containing 149 | ENSG00000181982 | 2.14 | 5.87E-39 |
| *H2BC15* | Histone H2B type 1-N | ENSG00000233822 | 2.14 | 5.20E-25 |
| *NEBL* | Nebulette | ENSG00000078114 | 2.14 | 3.22E-85 |
| *H1-5* | Histone H1.5 | ENSG00000184357 | 2.14 | 0.03 |
| *NKD2* | Protein naked cuticle homolog 2 | ENSG00000145506 | 2.14 | 5.31E-09 |
| *WIPI1* | WD repeat domain phosphoinositide-interacting protein 1 | ENSG00000070540 | 2.13 | 2.09E-189 |
| *H4C14* | H4 Clustered Histone 14 | ENSG00000270882 | 2.13 | 7.53E-86 |
| *GPCPD1* | Glycerophosphocholine phosphodiesterase GPCPD1 | ENSG00000125772 | 2.12 | 2.76E-216 |
| *UPP1* | Uridine phosphorylase 1 | ENSG00000183696 | 2.12 | 7.22E-97 |
| *LRAT* | Lecithin retinol acyltransferase | ENSG00000121207 | 2.12 | 3.68E-24 |
| *KRT79* | Keratin, type II cytoskeletal 79 | ENSG00000185640 | 2.11 | 0.02 |
| *LTF* | Lactotransferrin | ENSG00000012223 | 2.1 | 1.77E-90 |
| *ASNS* | Asparagine synthetase [glutamine-hydrolyzing] | ENSG00000070669 | 2.1 | 3.57E-211 |
| *H4C5* | H4 Clustered Histone 5 | ENSG00000276966 | 2.1 | 9.31E-06 |
| *MUC5B* | Mucin-5B | ENSG00000117983 | 2.1 | 0.03 |
| *FGF13* | Fibroblast growth factor 13 | ENSG00000129682 | 2.1 | 1.99E-08 |
| *AKR1C1* | Aldo-keto reductase family 1 member C1 | ENSG00000187134 | 2.09 | 4.44E-88 |
| *MEGF11* | Multiple epidermal growth factor-like domains protein 11 | ENSG00000157890 | 2.09 | 0.03 |
| *C7orf57* | Chromosome 7 Open Reading Frame 57 | ENSG00000164746 | 2.08 | 5.14E-07 |
| *MUC4* | Mucin-4 | ENSG00000145113 | 2.08 | 4.10E-80 |
| *PLA2G4E* | Cytosolic phospholipase A2 epsilon | ENSG00000188089 | 2.08 | 1.04E-21 |
| *FILIP1* | Filamin-A-interacting protein 1 | ENSG00000118407 | 2.07 | 1.44E-03 |
| *BEST1* | Bestrophin-1 | ENSG00000167995 | 2.07 | 4.29E-177 |
| *CXorf58* | Chromosome X Open Reading Frame 58 | ENSG00000165182 | 2.07 | 6.11E-03 |
| *DIRAS1* | GTP-binding protein Di-Ras1 | ENSG00000176490 | 2.07 | 6.92E-03 |
| *IL23A* | Interleukin-23 subunit alpha | ENSG00000110944 | 2.07 | 3.99E-10 |
| *RSPO4* | R-spondin-4 | ENSG00000101282 | 2.07 | 2.70E-12 |
| *NLRC4* | NLR family CARD domain-containing protein 4 | ENSG00000091106 | 2.07 | 0.01 |
| *ADGRE2* | Adhesion G protein-coupled receptor E2 | ENSG00000127507 | 2.07 | 1.82E-26 |
| *ARSF* | Arylsulfatase F | ENSG00000062096 | 2.06 | 3.27E-04 |
| *TENM1* | Teneurin-1 | ENSG00000009694 | 2.06 | 2.31E-06 |
| *TMC4* | Transmembrane channel-like protein 4 | ENSG00000167608 | 2.06 | 2.36E-47 |
| *MICAL2* | [F-actin]-monooxygenase MICAL2 | ENSG00000133816 | 2.05 | 3.02E-183 |
| *SRD5A3* | Polyprenol reductase | ENSG00000128039 | 2.04 | 1.06E-84 |
| *SLC9A2* | Sodium/hydrogen exchanger 2 | ENSG00000115616 | 2.04 | 1.26E-06 |
| *GAB2* | GRB2-associated-binding protein 2 | ENSG00000033327 | 2.03 | 5.51E-76 |
| *SLC6A14* | Sodium- and chloride-dependent neutral and basic amino acid transporter B(0+) | ENSG00000268104 | 2.02 | 1.42E-23 |
| *DMBT1* | Deleted in malignant brain tumors 1 protein | ENSG00000187908 | 2.02 | 2.13E-05 |
| *TMEM232* | Transmembrane protein 232 | ENSG00000186952 | 2.02 | 0.01 |
| *ANK2* | Ankyrin-2 | ENSG00000145362 | 2.02 | 1.19E-14 |
| *C6orf58* | Chromosome 6 Open Reading Frame 58 | ENSG00000184530 | 2.02 | 0.01 |
| *ERV3-1* | Endogenous retrovirus group 3 member 1 Env polyprotein | ENSG00000213462 | 2.02 | 1.62E-84 |
| *C15orf48* | Chromosome 15 Open Reading Frame 48 | ENSG00000166920 | 2.02 | 1.18E-41 |
| *SCGB1A1* | Uteroglobin | ENSG00000149021 | 2.01 | 2.10E-04 |
| *PYGB* | Glycogen phosphorylase, brain form | ENSG00000100994 | 2.01 | 9.41E-197 |
| *RHCG* | Ammonium transporter Rh type C | ENSG00000140519 | 2.01 | 6.69E-28 |
| *GREB1L* | GREB1-like protein | ENSG00000141449 | 2.01 | 0.02 |
| *KCNG1* | Potassium voltage-gated channel subfamily G member 1 | ENSG00000026559 | 2.01 | 4.52E-33 |
| *SPRY4* | Protein sprouty homolog 4 | ENSG00000187678 | 2 | 1.61E-21 |
| *ERO1B* | ERO1-like protein beta | ENSG00000086619 | 2 | 4.47E-80 |
| *TCN1* | Transcobalamin-1 | ENSG00000134827 | 2 | 4.19E-06 |
| *GOLT1A* | Vesicle transport protein GOT1A | ENSG00000174567 | 2 | 6.31E-16 |
| *TMEM217* | Transmembrane protein 217 | ENSG00000172738 | 2 | 4.95E-05 |
| *HYOU1* | Hypoxia up-regulated protein 1 | ENSG00000149428 | 2 | 2.29E-194 |
| *GPT2* | Alanine aminotransferase 2 | ENSG00000166123 | 1.99 | 6.12E-184 |
| *NPAS3* | Neuronal PAS domain-containing protein 3 | ENSG00000151322 | 1.99 | 1.95E-03 |
| *PDE9A* | High affinity cGMP-specific 3',5'-cyclic phosphodiesterase 9A | ENSG00000160191 | 1.98 | 3.99E-08 |
| *CDC42EP2* | Cdc42 effector protein 2 | ENSG00000149798 | 1.98 | 3.15E-71 |
| *ABCA10* | ATP-binding cassette sub-family A member 10 | ENSG00000154263 | 1.98 | 1.24E-24 |
| *TTC9* | Tetratricopeptide repeat protein 9A | ENSG00000133985 | 1.97 | 7.64E-41 |
| *ZNF853* | Zinc finger protein 853 | ENSG00000236609 | 1.97 | 0.02 |
| *HSP90B1* | Endoplasmin | ENSG00000166598 | 1.95 | 4.22E-83 |
| *MRGPRE* | Mas-related G-protein coupled receptor member E | ENSG00000184350 | 1.95 | 4.02E-03 |
| *XDH* | Xanthine dehydrogenase/oxidase | ENSG00000158125 | 1.95 | 1.10E-81 |
| *MIA2* | Melanoma inhibitory activity protein 2 | ENSG00000150527 | 1.95 | 2.44E-183 |
| *RYR3* | Ryanodine receptor 3 | ENSG00000198838 | 1.95 | 4.02E-05 |
| *GTPBP2* | GTP-binding protein 2 | ENSG00000172432 | 1.95 | 3.06E-182 |
| *POU2F2* | POU domain, class 2, transcription factor 2 | ENSG00000028277 | 1.94 | 5.97E-10 |
| *KCTD15* | BTB/POZ domain-containing protein KCTD15 | ENSG00000153885 | 1.93 | 3.20E-74 |
| *ARRDC4* | Arrestin domain-containing protein 4 | ENSG00000140450 | 1.93 | 2.26E-79 |
| *KRT86* | Keratin, type II cuticular Hb6 | ENSG00000170442 | 1.93 | 2.19E-08 |
| *N4BP3* | NEDD4-binding protein 3 | ENSG00000145911 | 1.93 | 1.28E-16 |
| *IFI44L* | Interferon-induced protein 44-like | ENSG00000137959 | 1.93 | 1.26E-36 |
| *ITGB3* | Integrin beta-3 | ENSG00000259207 | 1.93 | 0.05 |
| *GPAT3* | Glycerol-3-phosphate acyltransferase 3 | ENSG00000138678 | 1.92 | 5.31E-20 |
| *SLC6A9* | Sodium- and chloride-dependent glycine transporter 1 | ENSG00000196517 | 1.92 | 9.53E-171 |
| *SDCBP2* | Syntenin-2 | ENSG00000125775 | 1.92 | 2.56E-25 |
| *HAL* | Histidine ammonia-lyase | ENSG00000084110 | 1.91 | 7.00E-04 |
| *AKR1C3* | Aldo-keto reductase family 1 member C3 | ENSG00000196139 | 1.91 | 2.19E-47 |
| *CTXN1* | Cortexin-1 | ENSG00000178531 | 1.91 | 3.82E-08 |
| *H2AC15* | H2A clustered histone 15 | ENSG00000275221 | 1.91 | 5.20E-03 |
| *PRDM16* | Histone-lysine N-methyltransferase PRDM16 | ENSG00000142611 | 1.91 | 9.08E-17 |
| *H1-4* | Histone H1.4 | ENSG00000168298 | 1.91 | 1.32E-08 |
| *PTGS2* | Prostaglandin G/H synthase 2 | ENSG00000073756 | 1.9 | 6.04E-73 |
| *KCNJ11* | ATP-sensitive inward rectifier potassium channel 11 | ENSG00000187486 | 1.9 | 1.53E-03 |
| *DHRS3* | Short-chain dehydrogenase/reductase 3 | ENSG00000162496 | 1.9 | 2.05E-77 |
| *KRT83* | Keratin, type II cuticular Hb3 | ENSG00000170523 | 1.89 | 0.02 |
| *CCDC33* | Coiled-coil domain-containing protein 33 | ENSG00000140481 | 1.89 | 9.54E-06 |
| *BTBD19* | BTB domain containing 19 | ENSG00000222009 | 1.88 | 1.29E-03 |
| *RGS16* | Regulator of G-protein signaling 16 | ENSG00000143333 | 1.88 | 2.90E-62 |
| *STEAP2* | Metalloreductase STEAP2 | ENSG00000157214 | 1.88 | 2.33E-156 |
| *IL11* | Interleukin-11 | ENSG00000095752 | 1.87 | 1.13E-07 |
| *HRG* | Histidine-rich glycoprotein | ENSG00000113905 | 1.87 | 4.27E-03 |
| *CEBPG* | CCAAT/enhancer-binding protein gamma | ENSG00000153879 | 1.86 | 3.78E-164 |
| *IL36G* | Interleukin-36 gamma | ENSG00000136688 | 1.86 | 1.89E-26 |
| *DMGDH* | Dimethylglycine dehydrogenase, mitochondrial | ENSG00000132837 | 1.86 | 7.76E-07 |
| *ARG2* | Arginase-2, mitochondrial | ENSG00000081181 | 1.85 | 2.91E-67 |
| *SNAI1* | Zinc finger protein SNAI1 | ENSG00000124216 | 1.85 | 8.91E-12 |
| *H3C15* | H3 Clustered Histone 15 | ENSG00000203852 | 1.84 | 1.39E-07 |
| *EBF1* | Transcription factor COE1 | ENSG00000164330 | 1.84 | 0.03 |
| *WASL* | Neural Wiskott-Aldrich syndrome protein | ENSG00000106299 | 1.83 | 1.57E-162 |
| *RGS3* | Regulator of G-protein signaling 3 | ENSG00000138835 | 1.83 | 4.49E-144 |
| *TRIM31* | E3 ubiquitin-protein ligase TRIM31 | ENSG00000204616 | 1.82 | 1.34E-08 |
| *TRIB1* | Tribbles homolog 1 | ENSG00000173334 | 1.82 | 1.23E-42 |
| *MAP2* | Microtubule-associated protein 2 | ENSG00000078018 | 1.82 | 7.86E-29 |
| *CBX4* | E3 SUMO-protein ligase CBX4 | ENSG00000141582 | 1.82 | 2.07E-69 |
| *BDNF* | Brain-derived neurotrophic factor | ENSG00000176697 | 1.81 | 9.95E-10 |
| *H4C15* | H4 Clustered Histone 15 | ENSG00000270276 | 1.8 | 4.17E-21 |
| *PPP1R15A* | Protein phosphatase 1 regulatory subunit 15A | ENSG00000087074 | 1.8 | 1.07E-69 |
| *EDNRA* | Endothelin-1 receptor | ENSG00000151617 | 1.8 | 2.93E-16 |
| *DSCAM* | Down syndrome cell adhesion molecule | ENSG00000171587 | 1.8 | 2.01E-25 |
| *TRPV3* | Transient receptor potential cation channel subfamily V member 3 | ENSG00000167723 | 1.79 | 0.02 |
| *TBL1X* | F-box-like/WD repeat-containing protein TBL1X | ENSG00000101849 | 1.78 | 1.49E-64 |
| *FOXL2NB* | FOXL2 neighbor protein | ENSG00000206262 | 1.78 | 1.02E-09 |
| *C4orf19* | Uncharacterized protein C4orf19 | ENSG00000154274 | 1.78 | 8.13E-06 |
| *BCAN* | Brevican core protein | ENSG00000132692 | 1.78 | 2.96E-06 |
| *TMIE* | Transmembrane inner ear expressed protein | ENSG00000181585 | 1.78 | 4.42E-06 |
| *TMEM265* | Transmembrane protein 265 | ENSG00000281991 | 1.77 | 1.05E-05 |
| *XAF1* | XIAP-associated factor 1 | ENSG00000132530 | 1.77 | 5.48E-67 |
| *PKDCC* | Extracellular tyrosine-protein kinase PKDCC | ENSG00000162878 | 1.77 | 2.07E-07 |
| *MACROH2A2* | Core histone macro-H2A.2 | ENSG00000099284 | 1.77 | 0.01 |
| *RNF165* | E3 ubiquitin-protein ligase RNF165 | ENSG00000141622 | 1.77 | 3.54E-25 |
| *IL18R1* | Interleukin-18 receptor 1 | ENSG00000115604 | 1.76 | 2.94E-24 |
| *KCNT2* | Potassium channel subfamily T member 2 | ENSG00000162687 | 1.76 | 5.12E-03 |
| *NIPAL4* | Magnesium transporter NIPA4 | ENSG00000172548 | 1.76 | 2.91E-58 |
| *ADGRF4* | Adhesion G protein-coupled receptor F4 | ENSG00000153294 | 1.75 | 5.05E-57 |
| *PDIA4* | Protein disulfide-isomerase A4 | ENSG00000155660 | 1.75 | 6.16E-67 |
| *LRRC49* | Leucine-rich repeat-containing protein 49 | ENSG00000137821 | 1.75 | 4.17E-29 |
| *NYAP2* | Neuronal tyrosine-phosphorylated phosphoinositide-3-kinase adapter 2 | ENSG00000144460 | 1.75 | 0.02 |
| *MAFF* | Transcription factor MafF | ENSG00000185022 | 1.75 | 2.05E-64 |
| *PRKN* | E3 ubiquitin-protein ligase parkin | ENSG00000185345 | 1.75 | 9.03E-03 |
| *RNF145* | RING finger protein 145 | ENSG00000145860 | 1.74 | 2.80E-148 |
| *ADAM28* | Disintegrin and metalloproteinase domain-containing protein 28 | ENSG00000042980 | 1.74 | 2.22E-55 |
| *VGF* | Neurosecretory protein VGF | ENSG00000128564 | 1.74 | 2.26E-05 |
| *HSH2D* | Hematopoietic SH2 domain-containing protein | ENSG00000196684 | 1.74 | 9.18E-23 |
| *PMEPA1* | Protein TMEPAI | ENSG00000124225 | 1.74 | 3.27E-64 |
| *KLHL29* | Kelch-like protein 29 | ENSG00000119771 | 1.74 | 8.09E-120 |
| *SRMS* | Tyrosine-protein kinase Srms | ENSG00000125508 | 1.74 | 1.11E-05 |
| *TRPV6* | Transient receptor potential cation channel subfamily V member 6 | ENSG00000165125 | 1.74 | 3.97E-36 |
| *PSAT1* | Phosphoserine aminotransferase | ENSG00000135069 | 1.73 | 3.57E-65 |
| *EREG* | Proepiregulin | ENSG00000124882 | 1.73 | 2.49E-63 |
| *SLC30A1* | Zinc transporter 1 | ENSG00000170385 | 1.73 | 1.49E-131 |
| *SIRPB2* | Signal-regulatory protein beta-2 | ENSG00000196209 | 1.73 | 1.96E-03 |
| *CCDC71L* | Coiled-coil domain-containing protein 71L | ENSG00000253276 | 1.73 | 2.49E-63 |
| *H3C1* | Histone H3.1 | ENSG00000275714 | 1.72 | 0.03 |
| *ANXA6* | Annexin A6 | ENSG00000197043 | 1.72 | 8.08E-19 |
| *IP6K3* | Inositol hexakisphosphate kinase 3 | ENSG00000161896 | 1.72 | 1.30E-11 |
| *EVI2B* | Protein EVI2B | ENSG00000185862 | 1.72 | 8.07E-06 |
| *SRPX2* | Sushi repeat-containing protein SRPX2 | ENSG00000102359 | 1.72 | 2.55E-56 |
| *RGCC* | Regulator of cell cycle RGCC | ENSG00000102760 | 1.72 | 1.01E-17 |
| *CDC42EP1* | Cdc42 effector protein 1 | ENSG00000128283 | 1.72 | 2.24E-134 |
| *TMEM125* | Transmembrane protein 125 | ENSG00000179178 | 1.72 | 4.88E-54 |
| *CH25H* | Cholesterol 25-hydroxylase | ENSG00000138135 | 1.71 | 1.39E-08 |
| *SLC22A15* | Solute carrier family 22 member 15 | ENSG00000163393 | 1.71 | 1.07E-30 |
| *SLC24A2* | Sodium/potassium/calcium exchanger 2 | ENSG00000155886 | 1.71 | 2.18E-05 |
| *COL6A2* | Collagen alpha-2(VI) chain | ENSG00000142173 | 1.71 | 3.23E-57 |
| *BATF3* | Basic leucine zipper transcriptional factor ATF-like 3 | ENSG00000123685 | 1.71 | 3.07E-06 |
| *PHLDA1* | Pleckstrin homology-like domain family A member 1 | ENSG00000139289 | 1.71 | 4.65E-143 |
| *FOXD1* | Forkhead box protein D1 | ENSG00000251493 | 1.7 | 1.26E-30 |
| *LHFPL2* | LHFPL tetraspan subfamily member 2 protein | ENSG00000145685 | 1.7 | 2.08E-131 |
| *PLEKHG7* | Pleckstrin homology domain-containing family G member 7 | ENSG00000187510 | 1.7 | 1.76E-05 |
| *MILR1* | Allergin-1 | ENSG00000271605 | 1.7 | 4.00E-25 |
| *SLAMF7* | SLAM family member 7 | ENSG00000026751 | 1.7 | 6.32E-04 |
| *TTLL13P* | Tubulin polyglutamylase TTLL13P | ENSG00000213471 | 1.69 | 0.03 |
| *TIGD4* | Tigger transposable element-derived protein 4 | ENSG00000169989 | 1.68 | 0.03 |
| *SCNN1G* | Amiloride-sensitive sodium channel subunit gamma | ENSG00000166828 | 1.68 | 0.04 |
| *ZNF239* | Zinc finger protein 239 | ENSG00000196793 | 1.68 | 2.73E-21 |
| *MANF* | Mesencephalic astrocyte-derived neurotrophic factor | ENSG00000145050 | 1.67 | 3.63E-124 |
| *SH3TC2* | SH3 domain and tetratricopeptide repeat-containing protein 2 | ENSG00000169247 | 1.67 | 1.50E-55 |
| *NFE2L1* | Endoplasmic reticulum membrane sensor NFE2L1 | ENSG00000082641 | 1.67 | 1.39E-60 |
| *OSBP2* | Oxysterol-binding protein 2 | ENSG00000184792 | 1.66 | 2.56E-18 |
| *H1-2* | Histone H1.2 | ENSG00000187837 | 1.66 | 1.83E-122 |
| *TBC1D3D* | TBC1 domain family member 3D | ENSG00000274419 | 1.66 | 9.68E-03 |
| *FCAMR* | High affinity immunoglobulin alpha and immunoglobulin mu Fc receptor | ENSG00000162897 | 1.66 | 7.67E-03 |
| *SLC22A18AS* | Beckwith-Wiedemann syndrome chromosomal region 1 candidate gene B protein | ENSG00000254827 | 1.66 | 4.60E-15 |
| *GOLGA6A* | Golgin subfamily A member 6A | ENSG00000159289 | 1.65 | 0.03 |
| *BMP6* | Bone morphogenetic protein 6 | ENSG00000153162 | 1.65 | 8.10E-03 |
| *H2BC5* | Histone H2B type 1-D | ENSG00000158373 | 1.65 | 9.12E-45 |
| *CLDN7* | Claudin-7 | ENSG00000181885 | 1.65 | 1.83E-58 |
| *LCA5L* | Lebercilin-like protein | ENSG00000157578 | 1.65 | 3.15E-09 |
| *RASL11B* | Ras-like protein family member 11B | ENSG00000128045 | 1.64 | 5.52E-04 |
| *KIF26B* | Kinesin-like protein KIF26B | ENSG00000162849 | 1.64 | 2.10E-16 |
| *TMC5* | Transmembrane channel-like protein 5 | ENSG00000103534 | 1.64 | 9.36E-118 |
| *CRELD1* | Protein disulfide isomerase CRELD1 | ENSG00000163703 | 1.64 | 5.90E-39 |
| *MXD1* | Max dimerization protein 1 | ENSG00000059728 | 1.64 | 1.66E-56 |
| *ST6GALNAC1* | Alpha-N-acetylgalactosaminide alpha-2,6-sialyltransferase 1 | ENSG00000070526 | 1.63 | 8.61E-19 |
| *OSBPL6* | Oxysterol-binding protein-related protein 6 | ENSG00000079156 | 1.63 | 7.40E-11 |
| *SIM2* | Single-minded homolog 2 | ENSG00000159263 | 1.63 | 1.84E-17 |
| *H2BC6* | H2B Clustered Histone 6 | ENSG00000274290 | 1.63 | 6.15E-08 |
| *EPHA10* | Ephrin type-A receptor 10 | ENSG00000183317 | 1.63 | 8.51E-08 |
| *PIWIL4* | Piwi-like protein 4 | ENSG00000134627 | 1.62 | 8.49E-03 |
| *PRKG2* | cGMP-dependent protein kinase 2 | ENSG00000138669 | 1.62 | 6.61E-06 |
| *ADAMTS17* | A disintegrin and metalloproteinase with thrombospondin motifs 17 | ENSG00000140470 | 1.62 | 2.83E-04 |
| *RAB40A* | Ras-related protein Rab-40A | ENSG00000172476 | 1.62 | 4.78E-04 |
| *ARHGEF38* | Rho guanine nucleotide exchange factor 38 | ENSG00000236699 | 1.62 | 7.16E-03 |
| *DUSP8* | Dual specificity protein phosphatase 8 | ENSG00000184545 | 1.62 | 4.55E-19 |
| *ARSD* | Arylsulfatase D | ENSG00000006756 | 1.61 | 5.50E-27 |
| *ZG16B* | Zymogen granule protein 16 homolog B | ENSG00000162078 | 1.61 | 4.83E-18 |
| *KIZ* | Centrosomal protein kizuna | ENSG00000088970 | 1.61 | 2.85E-50 |
| *RDH10* | Retinol dehydrogenase 10 | ENSG00000121039 | 1.61 | 4.89E-44 |
| *DHRS2* | Dehydrogenase/reductase SDR family member 2, mitochondrial | ENSG00000100867 | 1.61 | 1.06E-06 |
| *MGLL* | Monoglyceride lipase | ENSG00000074416 | 1.6 | 1.73E-49 |
| *SMCO2* | Single-pass membrane and coiled-coil domain-containing protein 2 | ENSG00000165935 | 1.6 | 2.50E-04 |
| *FOSL1* | Fos-related antigen 1 | ENSG00000175592 | 1.6 | 3.12E-55 |
| *RUNX1* | Runt-related transcription factor 1 | ENSG00000159216 | 1.6 | 2.65E-123 |
| *NUCB2* | Nucleobindin-2 | ENSG00000070081 | 1.6 | 3.95E-119 |
| *RYR2* | Ryanodine receptor 2 | ENSG00000198626 | 1.6 | 1.73E-06 |
| *TTLL7* | Tubulin polyglutamylase TTLL7 | ENSG00000137941 | 1.59 | 7.83E-27 |
| *XBP1* | X-box-binding protein 1 | ENSG00000100219 | 1.59 | 5.05E-122 |
| *BCAS1* | Breast carcinoma-amplified sequence 1 | ENSG00000064787 | 1.59 | 6.55E-05 |
| *SEC11C* | Signal peptidase complex catalytic subunit SEC11C | ENSG00000166562 | 1.58 | 3.76E-48 |
| *PLK3* | Serine/threonine-protein kinase PLK3 | ENSG00000173846 | 1.58 | 1.47E-45 |
| *MERTK* | Tyrosine-protein kinase Mer | ENSG00000153208 | 1.58 | 4.93E-12 |
| *PLA2G4C* | Cytosolic phospholipase A2 gamma | ENSG00000105499 | 1.58 | 4.28E-17 |
| *IFRD1* | Interferon-related developmental regulator 1 | ENSG00000006652 | 1.57 | 3.29E-52 |
| *PROM1* | Prominin-1 | ENSG00000007062 | 1.57 | 3.58E-04 |
| *RORA* | Nuclear receptor ROR-alpha | ENSG00000069667 | 1.56 | 4.81E-06 |
| *S100A7A* | Protein S100-A7A | ENSG00000184330 | 1.56 | 5.12E-22 |
| *ALOXE3* | Hydroperoxide isomerase ALOXE3 | ENSG00000179148 | 1.56 | 5.59E-13 |
| *ANTXR2* | Anthrax toxin receptor 2 | ENSG00000163297 | 1.56 | 1.64E-46 |
| *TMEM156* | Transmembrane protein 156 | ENSG00000121895 | 1.56 | 4.93E-16 |
| *INHBC* | Inhibin beta C chain | ENSG00000175189 | 1.56 | 0.02 |
| *SOX9* | Transcription factor SOX-9 | ENSG00000125398 | 1.56 | 1.69E-51 |
| *SLFN5* | Schlafen family member 5 | ENSG00000166750 | 1.55 | 1.69E-52 |
| *CHST6* | Carbohydrate sulfotransferase 6 | ENSG00000183196 | 1.55 | 3.07E-08 |
| *CP* | Ceruloplasmin | ENSG00000047457 | 1.55 | 1.02E-14 |
| *ALOX12B* | Arachidonate 12-lipoxygenase, 12R-type | ENSG00000179477 | 1.54 | 7.55E-03 |
| *ALDH1L1* | Cytosolic 10-formyltetrahydrofolate dehydrogenase | ENSG00000144908 | 1.54 | 5.17E-04 |
| *ARL14EPL* | ADP Ribosylation Factor Like GTPase 14 Effector Protein Like | ENSG00000268223 | 1.54 | 0.02 |
| *TMPRSS7* | Transmembrane protease serine 7 | ENSG00000176040 | 1.54 | 0.02 |
| *MAT1A* | S-adenosylmethionine synthase isoform type-1 | ENSG00000151224 | 1.54 | 6.93E-03 |
| *PPP1R9A* | Neurabin-1 | ENSG00000158528 | 1.54 | 8.70E-14 |
| *DNAJC10* | DnaJ homolog subfamily C member 10 | ENSG00000077232 | 1.53 | 4.30E-51 |
| *STEAP1* | Metalloreductase STEAP1 | ENSG00000164647 | 1.53 | 2.30E-98 |
| *SERPINE2* | Glia-derived nexin | ENSG00000135919 | 1.53 | 2.48E-101 |
| *BEST4* | Bestrophin-4 | ENSG00000142959 | 1.53 | 0.02 |
| *C3orf80* | Uncharacterized membrane protein C3orf80 | ENSG00000180044 | 1.52 | 0.01 |
| *PLLP* | Plasmolipin | ENSG00000102934 | 1.52 | 5.77E-20 |
| *ERFE* | Erythroferrone | ENSG00000178752 | 1.52 | 8.42E-24 |
| *FAM166C* | Protein FAM166C | ENSG00000173557 | 1.52 | 0.03 |
| *IL20RB* | Interleukin-20 receptor subunit beta | ENSG00000174564 | 1.51 | 9.69E-111 |
| *GARS1* | Glycine--tRNA ligase | ENSG00000106105 | 1.51 | 4.58E-50 |
| *IFI6* | Interferon alpha-inducible protein 6 | ENSG00000126709 | 1.51 | 7.05E-18 |
| *GLI1* | Zinc finger protein GLI1 | ENSG00000111087 | 1.51 | 0.02 |
| *MMP24OS* | MMP24 Opposite Strand | ENSG00000126005 | 1.51 | 7.98E-11 |
| *LIPH* | Lipase member H | ENSG00000163898 | 1.5 | 8.73E-43 |
| *PDE2A* | cGMP-dependent 3',5'-cyclic phosphodiesterase | ENSG00000186642 | 1.5 | 9.80E-04 |
| *SPDEF* | SAM pointed domain-containing Ets transcription factor | ENSG00000124664 | 1.5 | 3.15E-12 |
| *SEL1L* | Protein sel-1 homolog 1 | ENSG00000071537 | 1.5 | 6.77E-110 |
| *JMY* | Junction-mediating and -regulatory protein | ENSG00000152409 | 1.49 | 4.37E-47 |
| *SMIM22* | Small integral membrane protein 22 | ENSG00000267795 | 1.49 | 1.17E-05 |
| *VNN1* | Pantetheinase | ENSG00000112299 | 1.49 | 2.30E-20 |
| *ITGA1* | Integrin alpha-1 | ENSG00000213949 | 1.49 | 1.45E-43 |
| *FKBP11* | Peptidyl-prolyl cis-trans isomerase FKBP11 | ENSG00000134285 | 1.49 | 6.24E-97 |
| *ST3GAL1* | CMP-N-acetylneuraminate-beta-galactosamide-alpha-2,3-sialyltransferase 1 | ENSG00000008513 | 1.48 | 1.41E-95 |
| *ZNF165* | Zinc finger protein 165 | ENSG00000197279 | 1.48 | 2.71E-28 |
| *GRIN1* | Glutamate receptor ionotropic, NMDA 1 | ENSG00000176884 | 1.47 | 4.28E-03 |
| *H3C10* | H3 Clustered Histone 10 | ENSG00000278828 | 1.47 | 1.60E-13 |
| *NAT8L* | N-acetylaspartate synthetase | ENSG00000185818 | 1.47 | 2.89E-08 |
| *NDRG2* | Protein NDRG2 | ENSG00000165795 | 1.47 | 7.03E-47 |
| *SERPINA3* | Alpha-1-antichymotrypsin | ENSG00000196136 | 1.47 | 1.81E-27 |
| *CHST2* | Carbohydrate sulfotransferase 2 | ENSG00000175040 | 1.46 | 1.86E-05 |
| *DCP1B* | mRNA-decapping enzyme 1B | ENSG00000151065 | 1.46 | 5.25E-03 |
| *SULT1E1* | Sulfotransferase 1E1 | ENSG00000109193 | 1.46 | 0.03 |
| *DNAJB11* | DnaJ homolog subfamily B member 11 | ENSG00000090520 | 1.46 | 2.69E-103 |
| *FKBP10* | Peptidyl-prolyl cis-trans isomerase FKBP10 | ENSG00000141756 | 1.46 | 3.73E-04 |
| *CYP4V2* | Cytochrome P450 4V2 | ENSG00000145476 | 1.46 | 6.68E-38 |
| *MX2* | Interferon-induced GTP-binding protein Mx2 | ENSG00000183486 | 1.46 | 1.58E-19 |
| *SELENOM* | Selenoprotein M | ENSG00000198832 | 1.46 | 4.78E-20 |
| *CRELD2* | Protein disulfide isomerase CRELD2 | ENSG00000184164 | 1.46 | 1.42E-44 |
| *GJA1* | Gap junction alpha-1 protein | ENSG00000152661 | 1.45 | 1.48E-44 |
| *TXNIP* | Thioredoxin-interacting protein | ENSG00000265972 | 1.45 | 6.90E-24 |
| *QPCT* | Glutaminyl-peptide cyclotransferase | ENSG00000115828 | 1.45 | 1.65E-19 |
| *H2AC11* | Histone H2A type 1 | ENSG00000196787 | 1.45 | 8.21E-09 |
| *MUC20* | Mucin-20 | ENSG00000176945 | 1.45 | 5.27E-45 |
| *LMO4* | LIM domain transcription factor LMO4 | ENSG00000143013 | 1.44 | 8.48E-96 |
| *EML1* | Echinoderm microtubule-associated protein-like 1 | ENSG00000066629 | 1.44 | 6.64E-16 |
| *PKHD1L1* | Fibrocystin-L | ENSG00000205038 | 1.44 | 4.92E-04 |
| *OLFM1* | Noelin | ENSG00000130558 | 1.44 | 6.46E-04 |
| *ARC* | Activity-regulated cytoskeleton-associated protein | ENSG00000198576 | 1.44 | 0.03 |
| *FOXL2* | Forkhead box protein L2 | ENSG00000183770 | 1.44 | 1.44E-12 |
| *SMKR1* | Small Lysine Rich Protein 1 | ENSG00000240204 | 1.44 | 4.99E-08 |
| *PALM2AKAP2* | PALM2 and AKAP2 fusion | ENSG00000157654 | 1.44 | 2.04E-05 |
| *H2AC18* | Histone H2A type 2-A | ENSG00000288825 | 1.44 | 4.05E-14 |
| *ARHGAP25* | Rho GTPase-activating protein 25 | ENSG00000163219 | 1.44 | 9.34E-10 |
| *SYNE3* | Nesprin-3 | ENSG00000176438 | 1.43 | 7.93E-06 |
| *RASGRP3* | Ras guanyl-releasing protein 3 | ENSG00000152689 | 1.43 | 1.58E-04 |
| *TMEM38A* | Trimeric intracellular cation channel type A | ENSG00000072954 | 1.42 | 1.98E-19 |
| *ARFGEF3* | Brefeldin A-inhibited guanine nucleotide-exchange protein 3 | ENSG00000112379 | 1.42 | 1.32E-42 |
| *ATF3* | Cyclic AMP-dependent transcription factor ATF-3 | ENSG00000162772 | 1.42 | 1.03E-26 |
| *PDIA3* | Protein disulfide-isomerase A3 | ENSG00000167004 | 1.42 | 9.80E-101 |
| *TMPRSS4* | Transmembrane protease serine 4 | ENSG00000137648 | 1.42 | 2.23E-19 |
| *RCAN1* | Calcipressin-1 | ENSG00000159200 | 1.42 | 3.66E-43 |
| *SDF2L1* | Stromal cell-derived factor 2-like protein 1 | ENSG00000128228 | 1.42 | 1.12E-39 |
| *LY96* | Lymphocyte antigen 96 | ENSG00000154589 | 1.42 | 3.76E-04 |
| *PVR* | Poliovirus receptor | ENSG00000073008 | 1.42 | 1.30E-96 |
| *HOXB9* | Homeobox protein Hox-B9 | ENSG00000170689 | 1.42 | 3.76E-18 |
| *ITIH4* | Inter-alpha-trypsin inhibitor heavy chain H4 | ENSG00000055955 | 1.42 | 4.94E-04 |
| *EBF4* | Transcription factor COE4 | ENSG00000088881 | 1.41 | 8.53E-12 |
| *ABCC3* | ATP-binding cassette sub-family C member 3 | ENSG00000108846 | 1.41 | 4.00E-92 |
| *SGTB* | Small glutamine-rich tetratricopeptide repeat-containing protein beta | ENSG00000197860 | 1.41 | 1.24E-39 |
| *FAM3C* | Protein FAM3C | ENSG00000196937 | 1.41 | 1.43E-43 |
| *FRY* | Protein furry homolog | ENSG00000073910 | 1.41 | 3.78E-04 |
| *SH3BGRL2* | SH3 domain-binding glutamic acid-rich-like protein 2 | ENSG00000198478 | 1.41 | 1.52E-36 |
| *PRRX2* | Paired mesoderm homeobox protein 2 | ENSG00000167157 | 1.4 | 1.71E-04 |
| *HIC1* | Hypermethylated in cancer 1 protein | ENSG00000177374 | 1.4 | 1.87E-17 |
| *HSPA13* | Heat shock 70 kDa protein 13 | ENSG00000155304 | 1.4 | 2.93E-92 |
| *PCSK1N* | ProSAAS | ENSG00000102109 | 1.4 | 2.46E-05 |
| *MRGPRX3* | Mas-related G-protein coupled receptor member X3 | ENSG00000179826 | 1.4 | 5.62E-05 |
| *RBP7* | Retinoid-binding protein 7 | ENSG00000162444 | 1.4 | 0.03 |
| *INPP5J* | Phosphatidylinositol 4,5-bisphosphate 5-phosphatase A | ENSG00000185133 | 1.4 | 4.96E-08 |
| *IRS2* | Insulin receptor substrate 2 | ENSG00000185950 | 1.4 | 3.57E-85 |
| *H1-0* | Histone H1.0 | ENSG00000189060 | 1.39 | 2.60E-42 |
| *PIGA* | Phosphatidylinositol N-acetylglucosaminyltransferase subunit A | ENSG00000165195 | 1.39 | 9.31E-40 |
| *GCOM1* | GRINL1A complex locus protein 1 | ENSG00000137878 | 1.39 | 1.08E-10 |
| *MKNK2* | MAP kinase-interacting serine/threonine-protein kinase 2 | ENSG00000099875 | 1.39 | 2.35E-42 |
| *H2AW* | Histone H2A type 3 | ENSG00000181218 | 1.39 | 1.68E-17 |
| *H4-16* | Histone H4 | ENSG00000197837 | 1.39 | 3.96E-09 |
| *PLCXD2* | PI-PLC X domain-containing protein 2 | ENSG00000240891 | 1.39 | 2.39E-16 |
| *SPATA18* | Mitochondria-eating protein | ENSG00000163071 | 1.39 | 5.73E-12 |
| *KRT81* | Keratin, type II cuticular Hb1 | ENSG00000205426 | 1.38 | 3.95E-05 |
| *CDKN1A* | Cyclin-dependent kinase inhibitor 1 | ENSG00000124762 | 1.38 | 8.98E-41 |
| *MMEL1* | Membrane metallo-endopeptidase-like 1 | ENSG00000142606 | 1.38 | 0.01 |
| *MYH14* | Myosin-14 | ENSG00000105357 | 1.38 | 6.18E-38 |
| *FUCA1* | Tissue alpha-L-fucosidase | ENSG00000179163 | 1.38 | 7.66E-36 |
| *TMEM154* | Transmembrane protein 154 | ENSG00000170006 | 1.38 | 1.17E-84 |
| *NIBAN1* | Protein Niban 1 | ENSG00000135842 | 1.38 | 2.00E-41 |
| *KDM7A* | Lysine-specific demethylase 7A | ENSG00000006459 | 1.37 | 1.58E-37 |
| *STX1A* | Syntaxin-1A | ENSG00000106089 | 1.37 | 7.17E-19 |
| *PYCR1* | Pyrroline-5-carboxylate reductase 1, mitochondrial | ENSG00000183010 | 1.37 | 3.13E-88 |
| *SEC24D* | Protein transport protein Sec24D | ENSG00000150961 | 1.37 | 2.53E-86 |
| *CLEC11A* | C-type lectin domain family 11 member A | ENSG00000105472 | 1.37 | 0.03 |
| *KRTAP4-1* | Keratin-associated protein 4-1 | ENSG00000198443 | 1.37 | 4.34E-03 |
| *ESRP1* | Epithelial splicing regulatory protein 1 | ENSG00000104413 | 1.36 | 2.79E-91 |
| *TNC* | Tenascin | ENSG00000041982 | 1.36 | 1.53E-40 |
| *ARHGEF37* | Rho guanine nucleotide exchange factor 37 | ENSG00000183111 | 1.36 | 1.20E-37 |
| *SLC1A1* | Excitatory amino acid transporter 3 | ENSG00000106688 | 1.35 | 7.83E-06 |
| *RNF187* | E3 ubiquitin-protein ligase RNF187 | ENSG00000168159 | 1.35 | 1.48E-88 |
| *OCLN* | Occludin | ENSG00000197822 | 1.35 | 1.39E-37 |
| *SEPTIN5* | Septin-5 | ENSG00000184702 | 1.35 | 1.59E-09 |
| *LARP1B* | La-related protein 1B | ENSG00000138709 | 1.35 | 3.39E-36 |
| *ZNF117* | Zinc finger protein 117 | ENSG00000152926 | 1.35 | 1.31E-37 |
| *E2F5* | Transcription factor E2F5 | ENSG00000133740 | 1.34 | 2.48E-34 |
| *HLA-B* | HLA class I histocompatibility antigen, B alpha chain | ENSG00000234745 | 1.34 | 2.68E-39 |
| *GLRX* | Glutaredoxin-1 | ENSG00000173221 | 1.33 | 7.48E-36 |
| *HUS1B* | Checkpoint protein HUS1B | ENSG00000188996 | 1.33 | 0.02 |
| *C4B* | Complement C4-B | ENSG00000224389 | 1.33 | 5.71E-07 |
| *GAREM1* | GRB2-associated and regulator of MAPK protein 1 | ENSG00000141441 | 1.33 | 4.52E-34 |
| *GCNT4* | Beta-1,3-galactosyl-O-glycosyl-glycoprotein beta-1,6-N-acetylglucosaminyltransferase 4 | ENSG00000176928 | 1.33 | 3.81E-10 |
| *DYSF* | Dysferlin | ENSG00000135636 | 1.33 | 2.10E-15 |
| *EGR2* | E3 SUMO-protein ligase EGR2 | ENSG00000122877 | 1.33 | 1.70E-12 |
| *MANSC1* | MANSC domain-containing protein 1 | ENSG00000111261 | 1.32 | 1.68E-16 |
| *CDC42EP5* | Cdc42 effector protein 5 | ENSG00000167617 | 1.32 | 5.09E-04 |
| *EGR4* | Early growth response protein 4 | ENSG00000135625 | 1.32 | 1.25E-03 |
| *SYVN1* | E3 ubiquitin-protein ligase synoviolin | ENSG00000162298 | 1.32 | 1.36E-84 |
| *CCL20* | C-C motif chemokine 20 | ENSG00000115009 | 1.32 | 2.67E-37 |
| *TMEM52* | Transmembrane protein 52 | ENSG00000178821 | 1.32 | 2.38E-08 |
| *ETV4* | ETS translocation variant 4 | ENSG00000175832 | 1.31 | 1.69E-34 |
| *KCNS1* | Potassium voltage-gated channel subfamily S member 1 | ENSG00000124134 | 1.31 | 3.01E-06 |
| *BICDL2* | BICD family-like cargo adapter 2 | ENSG00000162069 | 1.31 | 2.43E-72 |
| *SERPINB8* | Serpin B8 | ENSG00000166401 | 1.31 | 1.32E-33 |
| *NOD2* | Nucleotide-binding oligomerization domain-containing protein 2 | ENSG00000167207 | 1.31 | 1.24E-31 |
| *PYROXD1* | Pyridine nucleotide-disulfide oxidoreductase domain-containing protein 1 | ENSG00000121350 | 1.31 | 2.05E-34 |
| *ALG2* | Alpha-1,3/1,6-mannosyltransferase ALG2 | ENSG00000119523 | 1.3 | 1.59E-80 |
| *CCT6B* | T-complex protein 1 subunit zeta-2 | ENSG00000132141 | 1.3 | 1.35E-09 |
| *PABPC1L* | Polyadenylate-binding protein 1-like | ENSG00000101104 | 1.3 | 4.54E-34 |
| *MPZL3* | Myelin protein zero-like protein 3 | ENSG00000160588 | 1.3 | 1.63E-30 |
| *BACH2* | Transcription regulator protein BACH2 | ENSG00000112182 | 1.3 | 6.58E-04 |
| *H3C6* | H3 Clustered Histone 6 | ENSG00000274750 | 1.3 | 1.43E-16 |
| *RNF39* | RING finger protein 39 | ENSG00000204618 | 1.3 | 1.32E-11 |
| *H2BC11* | Histone H2B type 1-J | ENSG00000124635 | 1.3 | 4.48E-07 |
| *SERP1* | Stress-associated endoplasmic reticulum protein 1 | ENSG00000120742 | 1.3 | 1.24E-82 |
| *CPD* | Carboxypeptidase D | ENSG00000108582 | 1.3 | 6.31E-37 |
| *ABCA3* | Phospholipid-transporting ATPase ABCA3 | ENSG00000167972 | 1.3 | 3.57E-06 |
| *DNAJC3* | DnaJ homolog subfamily C member 3 | ENSG00000102580 | 1.3 | 1.89E-80 |
| *PCDH1* | Protocadherin-1 | ENSG00000156453 | 1.29 | 4.83E-78 |
| *TNFRSF9* | Tumor necrosis factor receptor superfamily member 9 | ENSG00000049249 | 1.29 | 6.80E-16 |
| *DNAJC1* | DnaJ homolog subfamily C member 1 | ENSG00000136770 | 1.29 | 2.60E-33 |
| *SFTPD* | Pulmonary surfactant-associated protein D | ENSG00000133661 | 1.29 | 0.02 |
| *XPOT* | Exportin-T | ENSG00000184575 | 1.29 | 1.38E-82 |
| *DUSP7* | Dual specificity protein phosphatase 7 | ENSG00000164086 | 1.29 | 9.00E-36 |
| *FOXQ1* | Forkhead box protein Q1 | ENSG00000164379 | 1.29 | 4.46E-72 |
| *LZTS3* | Leucine zipper putative tumor suppressor 3 | ENSG00000088899 | 1.29 | 8.69E-22 |
| *EGF* | Pro-epidermal growth factor | ENSG00000138798 | 1.29 | 3.60E-09 |
| *ATP2B1* | Plasma membrane calcium-transporting ATPase 1 | ENSG00000070961 | 1.29 | 4.50E-36 |
| *TXNRD1* | Thioredoxin reductase 1, cytoplasmic | ENSG00000198431 | 1.29 | 2.07E-36 |
| *FSCN2* | Fascin-2 | ENSG00000186765 | 1.29 | 3.02E-03 |
| *CLIC2* | Chloride intracellular channel protein 2 | ENSG00000155962 | 1.28 | 0.02 |
| *TUBE1* | Tubulin epsilon chain | ENSG00000074935 | 1.28 | 5.56E-34 |
| *RNF223* | RING finger protein 223 | ENSG00000237330 | 1.28 | 3.00E-12 |
| *TBC1D20* | TBC1 domain family member 20 | ENSG00000125875 | 1.28 | 3.49E-77 |
| *RET* | Proto-oncogene tyrosine-protein kinase receptor Ret | ENSG00000165731 | 1.28 | 4.45E-11 |
| *SYTL5* | Synaptotagmin-like protein 5 | ENSG00000147041 | 1.28 | 0.02 |
| *KCNN3* | Small conductance calcium-activated potassium channel protein 3 | ENSG00000143603 | 1.28 | 0.04 |
| *PLPP5* | Phospholipid phosphatase 5 | ENSG00000147535 | 1.28 | 3.57E-32 |
| *DUSP1* | Dual specificity protein phosphatase 1 | ENSG00000120129 | 1.28 | 8.30E-21 |
| *SLC37A2* | Glucose-6-phosphate exchanger SLC37A2 | ENSG00000134955 | 1.28 | 3.91E-31 |
| *CASK* | Peripheral plasma membrane protein CASK | ENSG00000147044 | 1.28 | 4.02E-79 |
| *CDH4* | Cadherin-4 | ENSG00000179242 | 1.28 | 6.71E-03 |
| *GALNT14* | Polypeptide N-acetylgalactosaminyltransferase 14 | ENSG00000158089 | 1.28 | 0.01 |
| *DCLK2* | Serine/threonine-protein kinase DCLK2 | ENSG00000170390 | 1.27 | 0.02 |
| *PDLIM3* | PDZ and LIM domain protein 3 | ENSG00000154553 | 1.27 | 5.02E-04 |
| *SRPRB* | Signal recognition particle receptor subunit beta | ENSG00000144867 | 1.27 | 1.96E-77 |
| *RAB6A* | Ras-related protein Rab-6A | ENSG00000175582 | 1.27 | 9.37E-80 |
| *C10orf67* | Uncharacterized protein C10orf67, mitochondrial | ENSG00000179133 | 1.27 | 5.80E-03 |
| *BCAT1* | Branched-chain-amino-acid aminotransferase, cytosolic | ENSG00000060982 | 1.27 | 4.93E-35 |
| *CCDC187* | Coiled-coil domain-containing protein 187 | ENSG00000260220 | 1.27 | 1.59E-12 |
| *TPBG* | Trophoblast glycoprotein | ENSG00000146242 | 1.27 | 4.39E-35 |
| *SYT7* | Synaptotagmin-7 | ENSG00000011347 | 1.27 | 4.35E-15 |
| *ZXDA* | Zinc finger X-linked protein ZXDA | ENSG00000198205 | 1.27 | 1.39E-12 |
| *CLDN3* | Claudin-3 | ENSG00000165215 | 1.27 | 0.03 |
| *ZNF350* | Zinc finger protein 350 | ENSG00000256683 | 1.27 | 1.78E-07 |
| *SCO2* | Protein SCO2 homolog, mitochondrial | ENSG00000284194 | 1.26 | 1.53E-23 |
| *ANKRD29* | Ankyrin repeat domain-containing protein 29 | ENSG00000154065 | 1.26 | 5.88E-08 |
| *GOLGA6L9* | Golgin subfamily A member 6-like protein 9 | ENSG00000197978 | 1.26 | 1.31E-24 |
| *FYB1* | FYN-binding protein 1 | ENSG00000082074 | 1.26 | 1.17E-74 |
| *ZBTB20* | Zinc finger and BTB domain-containing protein 20 | ENSG00000181722 | 1.26 | 2.89E-05 |
| *GSAP* | Gamma-secretase-activating protein | ENSG00000186088 | 1.25 | 4.89E-30 |
| *ABCA13* | ATP-binding cassette sub-family A member 13 | ENSG00000179869 | 1.25 | 1.47E-29 |
| *TCEA1* | Transcription elongation factor A protein 1 | ENSG00000187735 | 1.25 | 2.49E-34 |
| *IL20RA* | Interleukin-20 receptor subunit alpha | ENSG00000016402 | 1.25 | 1.91E-10 |
| *S100A7* | Protein S100-A7 | ENSG00000143556 | 1.25 | 7.66E-12 |
| *TCF24* | Transcription factor 24 | ENSG00000261787 | 1.24 | 0.04 |
| *FTCDNL1* | Formiminotransferase N-terminal subdomain-containing protein | ENSG00000226124 | 1.24 | 1.99E-03 |
| *WFDC5* | WAP four-disulfide core domain protein 5 | ENSG00000175121 | 1.24 | 1.51E-04 |
| *FBLN5* | Fibulin-5 | ENSG00000140092 | 1.24 | 8.79E-04 |
| *KLHL35* | Kelch-like protein 35 | ENSG00000149243 | 1.24 | 6.12E-05 |
| *CPEB3* | Cytoplasmic polyadenylation element-binding protein 3 | ENSG00000107864 | 1.24 | 1.21E-16 |
| *SEC61A1* | Protein transport protein Sec61 subunit alpha isoform 1 | ENSG00000058262 | 1.24 | 3.33E-76 |
| *SERPING1* | Plasma protease C1 inhibitor | ENSG00000149131 | 1.24 | 4.61E-10 |
| *ODF3B* | Outer dense fiber protein 3B | ENSG00000177989 | 1.24 | 1.78E-14 |
| *MTHFD2* | Bifunctional methylenetetrahydrofolate dehydrogenase/cyclohydrolase, mitochondrial | ENSG00000065911 | 1.24 | 5.29E-75 |
| *SIL1* | Nucleotide exchange factor SIL1 | ENSG00000120725 | 1.24 | 4.08E-68 |
| *MOCOS* | Molybdenum cofactor sulfurase | ENSG00000075643 | 1.24 | 6.67E-70 |
| *AKR1B15* | Aldo-keto reductase family 1 member B15 | ENSG00000227471 | 1.24 | 2.44E-03 |
| *GRM4* | Metabotropic glutamate receptor 4 | ENSG00000124493 | 1.23 | 0.03 |
| *TSPYL2* | Testis-specific Y-encoded-like protein 2 | ENSG00000184205 | 1.23 | 1.84E-30 |
| *PCDHGA2* | Protocadherin gamma-A2 | ENSG00000081853 | 1.23 | 0.02 |
| *SRXN1* | Sulfiredoxin-1 | ENSG00000271303 | 1.23 | 4.86E-73 |
| *CHST4* | Carbohydrate sulfotransferase 4 | ENSG00000140835 | 1.23 | 6.07E-04 |
| *ITPRIP* | Inositol 1,4,5-trisphosphate receptor-interacting protein | ENSG00000148841 | 1.23 | 3.47E-73 |
| *NRIP1* | Nuclear receptor-interacting protein 1 | ENSG00000180530 | 1.23 | 4.83E-73 |
| *CNGB3* | Cyclic nucleotide-gated cation channel beta-3 | ENSG00000170289 | 1.23 | 0.01 |
| *ABTB2* | Ankyrin repeat and BTB/POZ domain-containing protein 2 | ENSG00000166016 | 1.23 | 4.27E-32 |
| *PEAR1* | Platelet endothelial aggregation receptor 1 | ENSG00000187800 | 1.23 | 3.82E-28 |
| *GRPEL2* | GrpE protein homolog 2, mitochondrial | ENSG00000164284 | 1.23 | 1.09E-67 |
| *BVES* | Blood vessel epicardial substance | ENSG00000112276 | 1.23 | 2.18E-29 |
| *BMPR1B* | Bone morphogenetic protein receptor type-1B | ENSG00000138696 | 1.23 | 1.17E-07 |
| *FAM71F2* | Family with Sequence Similarity 71 member F2 | ENSG00000205085 | 1.22 | 6.09E-05 |
| *GABRR2* | Gamma-aminobutyric acid receptor subunit rho-2 | ENSG00000111886 | 1.22 | 0.02 |
| *FAM83E* | Protein FAM83E | ENSG00000105523 | 1.22 | 1.20E-08 |
| *LAMA2* | Laminin subunit alpha-2 | ENSG00000196569 | 1.22 | 3.97E-03 |
| *RNF43* | E3 ubiquitin-protein ligase RNF43 | ENSG00000108375 | 1.22 | 3.94E-68 |
| *ADGRA2* | Adhesion G protein-coupled receptor A2 | ENSG00000020181 | 1.22 | 1.06E-03 |
| *EXOSC6* | Exosome complex component MTR3 | ENSG00000223496 | 1.22 | 1.41E-68 |
| *CFAP69* | Cilia- and flagella-associated protein 69 | ENSG00000105792 | 1.22 | 1.33E-10 |
| *LBH* | Protein LBH | ENSG00000213626 | 1.22 | 4.32E-06 |
| *ABHD5* | 1-acylglycerol-3-phosphate O-acyltransferase ABHD5 | ENSG00000011198 | 1.22 | 2.86E-30 |
| *ICA1L* | Islet cell autoantigen 1-like protein | ENSG00000163596 | 1.22 | 2.27E-04 |
| *FBXO25* | F-box only protein 25 | ENSG00000147364 | 1.21 | 2.07E-63 |
| *ZFAND2A* | AN1-type zinc finger protein 2A | ENSG00000178381 | 1.21 | 9.16E-30 |
| *TOX2* | TOX high mobility group box family member 2 | ENSG00000124191 | 1.21 | 1.08E-63 |
| *LENG9* | Leukocyte receptor cluster member 9 | ENSG00000275183 | 1.21 | 8.15E-15 |
| *TMEM88* | Transmembrane protein 88 | ENSG00000167874 | 1.21 | 1.51E-03 |
| *TRABD2A* | Metalloprotease TIKI1 | ENSG00000186854 | 1.2 | 2.05E-05 |
| *CERS3* | Ceramide synthase 3 | ENSG00000154227 | 1.2 | 6.61E-61 |
| *LHX5* | LIM/homeobox protein Lhx5 | ENSG00000089116 | 1.2 | 2.10E-07 |
| *NGF* | Beta-nerve growth factor | ENSG00000134259 | 1.2 | 2.91E-04 |
| *STRC* | Stereocilin | ENSG00000242866 | 1.2 | 2.74E-03 |
| *SLPI* | Antileukoproteinase | ENSG00000124107 | 1.2 | 4.35E-15 |
| *STAT4* | Signal transducer and activator of transcription 4 | ENSG00000138378 | 1.2 | 1.23E-11 |
| *GGT1* | Glutathione hydrolase 1 proenzyme | ENSG00000100031 | 1.2 | 1.34E-26 |
| *BTG1* | Protein BTG1 | ENSG00000133639 | 1.2 | 3.76E-31 |
| *CCDC110* | Coiled-coil domain-containing protein 110 | ENSG00000168491 | 1.2 | 0.02 |
| *HM13* | Minor histocompatibility antigen H13 | ENSG00000101294 | 1.2 | 1.32E-69 |
| *PNRC1* | Proline-rich nuclear receptor coactivator 1 | ENSG00000146278 | 1.19 | 2.32E-27 |
| *UBE2J1* | Ubiquitin-conjugating enzyme E2 J1 | ENSG00000198833 | 1.19 | 4.03E-68 |
| *DYNLT4* | Dynein light chain Tctex-type 4 | ENSG00000188396 | 1.19 | 0.04 |
| *TRIM36* | E3 ubiquitin-protein ligase TRIM36 | ENSG00000152503 | 1.19 | 1.14E-07 |
| *TNFRSF10B* | Tumor necrosis factor receptor superfamily member 10B | ENSG00000120889 | 1.19 | 2.49E-68 |
| *PRELID3A* | PRELI domain containing protein 3A | ENSG00000141391 | 1.19 | 4.56E-12 |
| *CLEC12B* | C-type lectin domain family 12 member B | ENSG00000256660 | 1.19 | 0.02 |
| *SQSTM1* | Sequestosome-1 | ENSG00000161011 | 1.19 | 3.43E-31 |
| *FIBIN* | Fin bud initiation factor homolog | ENSG00000176971 | 1.19 | 8.10E-14 |
| *NPAS2* | Neuronal PAS domain-containing protein 2 | ENSG00000170485 | 1.19 | 4.29E-29 |
| *IL17D* | Interleukin-17D | ENSG00000172458 | 1.19 | 1.58E-13 |
| *SH3KBP1* | SH3 domain-containing kinase-binding protein 1 | ENSG00000147010 | 1.18 | 1.24E-29 |
| *P2RX4* | P2X purinoceptor 4 | ENSG00000135124 | 1.18 | 2.17E-63 |
| *SLC6A15* | Sodium-dependent neutral amino acid transporter B(0)AT2 | ENSG00000072041 | 1.18 | 1.41E-29 |
| *MICOS10-NBL1* | MICOS10-NBL1 Readthrough | ENSG00000270136 | 1.18 | 4.29E-03 |
| *SRCIN1* | SRC kinase signaling inhibitor 1 | ENSG00000277363 | 1.18 | 2.34E-06 |
| *FAM228B* | Family With Sequence Similarity 228 Member B | ENSG00000219626 | 1.18 | 8.58E-04 |
| *PPP2R5B* | Serine/threonine-protein phosphatase 2A 56 kDa regulatory subunit beta isoform | ENSG00000068971 | 1.18 | 7.56E-29 |
| *ACTBL2* | Beta-actin-like protein 2 | ENSG00000169067 | 1.18 | 3.96E-09 |
| *IFI27* | Interferon alpha-inducible protein 27, mitochondrial | ENSG00000165949 | 1.18 | 4.30E-15 |
| *DNAAF8* | Dynein axonemal assembly factor 8 | ENSG00000166246 | 1.18 | 0.04 |
| *SLC16A6* | Monocarboxylate transporter 7 | ENSG00000108932 | 1.17 | 8.80E-04 |
| *KBTBD11* | Kelch Repeat And BTB Domain Containing 11 | ENSG00000176595 | 1.17 | 9.51E-04 |
| *CLIP4* | CAP-Gly domain-containing linker protein 4 | ENSG00000115295 | 1.17 | 9.98E-66 |
| *WDR45* | WD repeat domain phosphoinositide-interacting protein 4 | ENSG00000196998 | 1.17 | 1.64E-59 |
| *NIPAL2* | NIPA-like protein 2 | ENSG00000104361 | 1.16 | 1.60E-26 |
| *PLEKHG4* | Puratrophin-1 | ENSG00000196155 | 1.16 | 2.38E-11 |
| *B4GALNT3* | Beta-1,4-N-acetylgalactosaminyltransferase 3 | ENSG00000139044 | 1.16 | 4.42E-12 |
| *C8orf44-SGK3* | C8orf44-SGK3 readthrough | ENSG00000288602 | 1.16 | 4.30E-04 |
| *USP36* | Ubiquitin carboxyl-terminal hydrolase 36 | ENSG00000055483 | 1.16 | 7.12E-29 |
| *TIMP1* | Metalloproteinase inhibitor 1 | ENSG00000102265 | 1.15 | 5.75E-25 |
| *ACTL10* | Actin-like protein 10 | ENSG00000288649 | 1.15 | 7.77E-11 |
| *PFN4* | Profilin-4 | ENSG00000176732 | 1.15 | 0.03 |
| *AHR* | Aryl hydrocarbon receptor | ENSG00000106546 | 1.15 | 1.54E-29 |
| *CEACAM19* | Carcinoembryonic antigen-related cell adhesion molecule 19 | ENSG00000186567 | 1.15 | 2.34E-20 |
| *PIK3R3* | Phosphatidylinositol 3-kinase regulatory subunit gamma | ENSG00000117461 | 1.15 | 1.82E-61 |
| *KLF11* | Krueppel-like factor 11 | ENSG00000172059 | 1.15 | 3.02E-27 |
| *SYTL2* | Synaptotagmin-like protein 2 | ENSG00000137501 | 1.15 | 9.10E-10 |
| *RAB3IL1* | Guanine nucleotide exchange factor for Rab-3A | ENSG00000167994 | 1.15 | 3.10E-24 |
| *MAP1LC3B2* | Microtubule-associated proteins 1A/1B light chain 3 beta 2 | ENSG00000258102 | 1.15 | 1.21E-03 |
| *CTIF* | CBP80/20-dependent translation initiation factor | ENSG00000134030 | 1.15 | 9.19E-27 |
| *LRRC37A3* | Leucine-rich repeat-containing protein 37A3 | ENSG00000176809 | 1.15 | 2.66E-09 |
| *PER2* | Period circadian protein homolog 2 | ENSG00000132326 | 1.15 | 3.35E-27 |
| *DNAH5* | Dynein axonemal heavy chain 5 | ENSG00000039139 | 1.14 | 1.94E-24 |
| *FAM86B1* | Putative protein N-methyltransferase FAM86B1 | ENSG00000186523 | 1.14 | 1.06E-15 |
| *GCLC* | Glutamate--cysteine ligase catalytic subunit | ENSG00000001084 | 1.14 | 4.12E-63 |
| *ORAI2* | Protein orai-2 | ENSG00000160991 | 1.14 | 1.38E-55 |
| *DPYSL2* | Dihydropyrimidinase-related protein 2 | ENSG00000092964 | 1.14 | 4.28E-06 |
| *RAB38* | Ras-related protein Rab-38 | ENSG00000123892 | 1.14 | 1.31E-62 |
| *SLC1A5* | Neutral amino acid transporter B(0) | ENSG00000105281 | 1.14 | 7.89E-29 |
| *CPLX1* | Complexin-1 | ENSG00000168993 | 1.14 | 3.78E-06 |
| *PI3* | Elafin | ENSG00000124102 | 1.14 | 6.23E-28 |
| *CR2* | Complement receptor type 2 | ENSG00000117322 | 1.14 | 2.55E-03 |
| *HRNR* | Hornerin | ENSG00000197915 | 1.14 | 1.79E-12 |
| *PLPPR2* | Phospholipid phosphatase-related protein type 2 | ENSG00000105520 | 1.13 | 2.04E-21 |
| *ZFAND3* | AN1-type zinc finger protein 3 | ENSG00000156639 | 1.13 | 3.19E-62 |
| *PGM3* | Phosphoacetylglucosamine mutase | ENSG00000013375 | 1.13 | 1.06E-60 |
| *SARS1* | Serine--tRNA ligase, cytoplasmic | ENSG00000031698 | 1.13 | 9.65E-63 |
| *SARM1* | NAD(+) hydrolase SARM1 | ENSG00000004139 | 1.13 | 0.02 |
| *BEND3* | BEN domain-containing protein 3 | ENSG00000178409 | 1.13 | 1.80E-24 |
| *ISG20* | Interferon-stimulated gene 20 kDa protein | ENSG00000172183 | 1.12 | 7.63E-11 |
| *ZDHHC14* | Palmitoyltransferase ZDHHC14 | ENSG00000175048 | 1.12 | 8.95E-24 |
| *FAM86B2* | Putative protein N-methyltransferase FAM86B2 | ENSG00000145002 | 1.12 | 8.27E-09 |
| *CLDN12* | Claudin-12 | ENSG00000157224 | 1.12 | 6.53E-61 |
| *PPIB* | Peptidyl-prolyl cis-trans isomerase B | ENSG00000166794 | 1.12 | 5.52E-62 |
| *TNFRSF12A* | Tumor necrosis factor receptor superfamily member 12A | ENSG00000006327 | 1.12 | 2.09E-27 |
| *CREB3L2* | Cyclic AMP-responsive element-binding protein 3-like protein 2 | ENSG00000182158 | 1.12 | 1.21E-59 |
| *GOLGA7B* | Golgin subfamily A member 7B | ENSG00000155265 | 1.11 | 1.16E-14 |
| *C19orf71* | Chromosome 19 Open Reading Frame 71 | ENSG00000183397 | 1.11 | 0.02 |
| *PCDHGA4* | Protocadherin gamma-A4 | ENSG00000262576 | 1.11 | 1.31E-07 |
| *TYMP* | Thymidine phosphorylase | ENSG00000025708 | 1.11 | 5.17E-27 |
| *SLC35C1* | GDP-fucose transporter 1 | ENSG00000181830 | 1.11 | 3.20E-52 |
| *TVP23C* | Golgi apparatus membrane protein TVP23 homolog C | ENSG00000175106 | 1.11 | 1.04E-22 |
| *TRAM1* | Translocating chain-associated membrane protein 1 | ENSG00000067167 | 1.1 | 2.04E-60 |
| *CFB* | Complement factor B | ENSG00000243649 | 1.1 | 7.85E-14 |
| *CANX* | Calnexin | ENSG00000127022 | 1.1 | 3.50E-27 |
| *ELOVL4* | Elongation of very long chain fatty acids protein 4 | ENSG00000118402 | 1.1 | 7.97E-10 |
| *JAK1* | Tyrosine-protein kinase JAK1 | ENSG00000162434 | 1.1 | 8.02E-60 |
| *PARP6* | Protein mono-ADP-ribosyltransferase PARP6 | ENSG00000137817 | 1.1 | 6.24E-55 |
| *SMIM13* | Small integral membrane protein 13 | ENSG00000224531 | 1.1 | 1.34E-25 |
| *IL24* | Interleukin-24 | ENSG00000162892 | 1.1 | 5.15E-03 |
| *RAP1GAP2* | Rap1 GTPase-activating protein 2 | ENSG00000132359 | 1.1 | 1.83E-06 |
| *CCDC92* | Coiled-coil domain-containing protein 92 | ENSG00000119242 | 1.1 | 1.76E-23 |
| *PLXNA3* | Plexin-A3 | ENSG00000130827 | 1.1 | 8.37E-55 |
| *PLEKHA6* | Pleckstrin homology domain-containing family A member 6 | ENSG00000143850 | 1.1 | 2.32E-24 |
| *NDUFA4L2* | NADH dehydrogenase [ubiquinone] 1 alpha subcomplex subunit 4-like 2 | ENSG00000185633 | 1.1 | 0.04 |
| *AIFM2* | Ferroptosis suppressor protein 1 | ENSG00000042286 | 1.1 | 7.27E-55 |
| *LSR* | Lipolysis-stimulated lipoprotein receptor | ENSG00000105699 | 1.1 | 1.31E-26 |
| *BEAN1* | Protein BEAN1 | ENSG00000166546 | 1.1 | 2.15E-03 |
| *GADD45A* | Growth arrest and DNA damage-inducible protein GADD45 alpha | ENSG00000116717 | 1.09 | 9.35E-26 |
| *SEPHS2* | Selenide, water dikinase 2 | ENSG00000179918 | 1.09 | 4.17E-57 |
| *GPLD1* | Phosphatidylinositol-glycan-specific phospholipase D | ENSG00000112293 | 1.09 | 1.54E-06 |
| *AKNA* | Microtubule organization protein AKNA | ENSG00000106948 | 1.09 | 8.43E-25 |
| *DSG3* | Desmoglein-3 | ENSG00000134757 | 1.09 | 2.48E-58 |
| *PCDHB15* | Protocadherin beta-15 | ENSG00000113248 | 1.09 | 0.01 |
| *GAL* | Galanin peptides | ENSG00000069482 | 1.09 | 5.06E-10 |
| *CYP2J2* | Cytochrome P450 2J2 | ENSG00000134716 | 1.09 | 5.95E-08 |
| *FEZ1* | Fasciculation and elongation protein zeta-1 | ENSG00000149557 | 1.09 | 2.43E-23 |
| *SH2D2A* | SH2 domain-containing protein 2A | ENSG00000027869 | 1.09 | 8.68E-06 |
| *ABCA1* | Phospholipid-transporting ATPase ABCA1 | ENSG00000165029 | 1.08 | 4.31E-25 |
| *P4HB* | Protein disulfide-isomerase | ENSG00000185624 | 1.08 | 3.77E-26 |
| *PPIL6* | Probable inactive peptidyl-prolyl cis-trans isomerase-like 6 | ENSG00000185250 | 1.08 | 8.82E-04 |
| *ADAP1* | Arf-GAP with dual PH domain-containing protein 1 | ENSG00000105963 | 1.08 | 7.40E-25 |
| *SH3PXD2B* | SH3 and PX domain-containing protein 2B | ENSG00000174705 | 1.08 | 1.46E-56 |
| *CD163L1* | Scavenger receptor cysteine-rich type 1 protein M160 | ENSG00000177675 | 1.08 | 2.26E-07 |
| *PRDM1* | PR domain zinc finger protein 1 | ENSG00000057657 | 1.08 | 2.57E-14 |
| *SLC38A1* | Sodium-coupled neutral amino acid transporter 1 | ENSG00000111371 | 1.08 | 7.19E-26 |
| *TCP11L2* | T-complex protein 11-like protein 2 | ENSG00000166046 | 1.08 | 2.12E-23 |
| *GOLGA6L10* | Golgin A6 Family Like 10 | ENSG00000278662 | 1.08 | 1.81E-11 |
| *KYNU* | Kynureninase | ENSG00000115919 | 1.08 | 9.45E-26 |
| *ERVMER34-1* | Endogenous retroviral envelope protein HEMO | ENSG00000226887 | 1.07 | 7.59E-11 |
| *RPS6KA2* | Ribosomal protein S6 kinase alpha-2 | ENSG00000071242 | 1.07 | 5.06E-25 |
| *ZNF467* | Zinc finger protein 467 | ENSG00000181444 | 1.07 | 1.11E-03 |
| *FUT1* | Galactoside alpha-(1,2)-fucosyltransferase 1 | ENSG00000174951 | 1.07 | 5.69E-17 |
| *RHOBTB1* | Rho-related BTB domain-containing protein 1 | ENSG00000072422 | 1.07 | 1.42E-05 |
| *UAP1* | UDP-N-acetylhexosamine pyrophosphorylase | ENSG00000117143 | 1.07 | 6.74E-25 |
| *GADD45G* | Growth arrest and DNA damage-inducible protein GADD45 gamma | ENSG00000130222 | 1.07 | 3.15E-03 |
| *DOC2B* | Double C2-like domain-containing protein beta | ENSG00000272636 | 1.07 | 1.63E-11 |
| *TMED5* | Transmembrane emp24 domain-containing protein 5 | ENSG00000117500 | 1.07 | 5.79E-56 |
| *EGR3* | Early growth response protein 3 | ENSG00000179388 | 1.07 | 1.47E-07 |
| *PRR22* | Proline Rich 22 | ENSG00000212123 | 1.07 | 4.67E-03 |
| *PHYHIP* | Phytanoyl-CoA hydroxylase-interacting protein | ENSG00000168490 | 1.07 | 0.02 |
| *SYTL1* | Synaptotagmin-like protein 1 | ENSG00000142765 | 1.07 | 1.91E-51 |
| *EIF4EBP1* | Eukaryotic translation initiation factor 4E-binding protein 1 | ENSG00000187840 | 1.07 | 1.73E-52 |
| *PDIA6* | Protein disulfide-isomerase A6 | ENSG00000143870 | 1.07 | 9.94E-57 |
| *H2BC12* | Histone H2B type 1-K | ENSG00000197903 | 1.07 | 8.11E-51 |
| *AARS1* | Alanine--tRNA ligase, cytoplasmic | ENSG00000090861 | 1.06 | 1.77E-56 |
| *SRSF8* | Serine/arginine-rich splicing factor 8 | ENSG00000263465 | 1.06 | 1.53E-51 |
| *AZGP1* | Zinc-alpha-2-glycoprotein | ENSG00000160862 | 1.06 | 3.26E-03 |
| *SMOX* | Spermine oxidase | ENSG00000088826 | 1.06 | 1.56E-22 |
| *DAB2* | Disabled homolog 2 | ENSG00000153071 | 1.06 | 4.64E-22 |
| *SAMD4A* | Protein Smaug homolog 1 | ENSG00000020577 | 1.06 | 2.46E-22 |
| *ZBED3* | Zinc finger BED domain-containing protein 3 | ENSG00000132846 | 1.06 | 7.80E-21 |
| *DUSP4* | Dual specificity protein phosphatase 4 | ENSG00000120875 | 1.06 | 3.74E-23 |
| *EFNB2* | Ephrin-B2 | ENSG00000125266 | 1.06 | 4.39E-24 |
| *GGACT* | Gamma-glutamylaminecyclotransferase | ENSG00000134864 | 1.06 | 4.58E-10 |
| *STX19* | Syntaxin-19 | ENSG00000178750 | 1.06 | 2.92E-03 |
| *DCHS2* | Protocadherin-23 | ENSG00000197410 | 1.06 | 0.03 |
| *COX6B2* | Cytochrome c oxidase subunit 6B2 | ENSG00000160471 | 1.05 | 8.10E-03 |
| *HEY1* | Hairy/enhancer-of-split related with YRPW motif protein 1 | ENSG00000164683 | 1.05 | 4.47E-07 |
| *FBXL16* | F-box/LRR-repeat protein 16 | ENSG00000127585 | 1.05 | 5.12E-06 |
| *AMIGO2* | Amphoterin-induced protein 2 | ENSG00000139211 | 1.05 | 1.98E-53 |
| *PTPN12* | Tyrosine-protein phosphatase non-receptor type 12 | ENSG00000127947 | 1.05 | 2.18E-24 |
| *OVOL1* | Putative transcription factor Ovo-like 1 | ENSG00000172818 | 1.05 | 2.60E-18 |
| *PRNP* | Major prion protein | ENSG00000171867 | 1.05 | 9.23E-55 |
| *CYB561* | Transmembrane ascorbate-dependent reductase CYB561 | ENSG00000008283 | 1.05 | 3.51E-53 |
| *SPAG17* | Sperm-associated antigen 17 | ENSG00000155761 | 1.05 | 0.02 |
| *SLC44A5* | Choline transporter-like protein 5 | ENSG00000137968 | 1.05 | 2.80E-21 |
| *SELENOS* | Selenoprotein S | ENSG00000131871 | 1.05 | 7.81E-50 |
| *PLCB2* | 1-phosphatidylinositol 4,5-bisphosphate phosphodiesterase beta-2 | ENSG00000137841 | 1.05 | 6.41E-04 |
| *C3* | Complement C3 | ENSG00000125730 | 1.05 | 1.80E-24 |
| *NR1D1* | Nuclear receptor subfamily 1 group D member 1 | ENSG00000126368 | 1.04 | 3.75E-21 |
| *NELL2* | Protein kinase C-binding protein NELL2 | ENSG00000184613 | 1.04 | 5.74E-05 |
| *SAA2-SAA4* | SAA2-SAA4 Readthrough | ENSG00000255071 | 1.04 | 3.13E-20 |
| *BORCS5* | BLOC-1-related complex subunit 5 | ENSG00000165714 | 1.04 | 1.48E-20 |
| *RGL1* | Ral guanine nucleotide dissociation stimulator-like 1 | ENSG00000143344 | 1.04 | 6.07E-08 |
| *PPP2R2C* | Serine/threonine-protein phosphatase 2A 55 kDa regulatory subunit B gamma isoform | ENSG00000074211 | 1.04 | 1.61E-15 |
| *CSRNP2* | Cysteine/serine-rich nuclear protein 2 | ENSG00000110925 | 1.04 | 1.81E-22 |
| *ADAM12* | Disintegrin and metalloproteinase domain-containing protein 12 | ENSG00000148848 | 1.04 | 4.82E-46 |
| *XK* | Membrane transport protein XK | ENSG00000047597 | 1.04 | 1.78E-06 |
| *CXCL2* | C-X-C motif chemokine 2 | ENSG00000081041 | 1.04 | 7.76E-23 |
| *PGLYRP3* | Peptidoglycan recognition protein 3 | ENSG00000159527 | 1.04 | 6.06E-08 |
| *NPDC1* | Neural proliferation differentiation and control protein 1 | ENSG00000107281 | 1.04 | 1.03E-48 |
| *PRR15* | Proline-rich protein 15 | ENSG00000176532 | 1.04 | 5.27E-11 |
| *PLAUR* | Urokinase plasminogen activator surface receptor | ENSG00000011422 | 1.04 | 5.12E-22 |
| *KCNQ4* | Potassium voltage-gated channel subfamily KQT member 4 | ENSG00000117013 | 1.04 | 3.53E-03 |
| *COL9A2* | Collagen alpha-2(IX) chain | ENSG00000049089 | 1.03 | 8.01E-13 |
| *IL12RB2* | Interleukin-12 receptor subunit beta-2 | ENSG00000081985 | 1.03 | 2.95E-07 |
| *MYADM* | Myeloid-associated differentiation marker | ENSG00000179820 | 1.03 | 7.35E-03 |
| *IDUA* | Alpha-L-iduronidase | ENSG00000127415 | 1.03 | 1.11E-19 |
| *PLSCR1* | Phospholipid scramblase 1 | ENSG00000188313 | 1.03 | 1.04E-52 |
| *LIF* | Leukemia inhibitory factor | ENSG00000128342 | 1.03 | 1.34E-51 |
| *ATF4* | Cyclic AMP-dependent transcription factor ATF-4 | ENSG00000128272 | 1.03 | 8.28E-24 |
| *ZNF777* | Zinc finger protein 777 | ENSG00000196453 | 1.03 | 2.24E-44 |
| *LTO1* | Protein LTO1 homolog | ENSG00000149716 | 1.03 | 2.47E-22 |
| *HRH1* | Histamine H1 receptor | ENSG00000196639 | 1.03 | 2.36E-13 |
| *SIPA1L1* | Signal-induced proliferation-associated 1-like protein 1 | ENSG00000197555 | 1.03 | 1.93E-50 |
| *DBN1* | Drebrin | ENSG00000113758 | 1.03 | 1.39E-48 |
| *HSPA9* | Stress-70 protein, mitochondrial | ENSG00000113013 | 1.03 | 1.40E-23 |
| *NSUN7* | Putative methyltransferase NSUN7 | ENSG00000179299 | 1.03 | 3.11E-11 |
| *ZNF275* | Zinc finger protein 275 | ENSG00000063587 | 1.03 | 1.44E-20 |
| *PKN1* | Serine/threonine-protein kinase N1 | ENSG00000123143 | 1.02 | 4.10E-50 |
| *RWDD2A* | RWD domain-containing protein 2A | ENSG00000013392 | 1.02 | 3.03E-11 |
| *BLNK* | B-cell linker protein | ENSG00000095585 | 1.02 | 5.63E-05 |
| *IL1A* | Interleukin-1 alpha | ENSG00000115008 | 1.02 | 1.89E-23 |
| *BATF2* | Basic leucine zipper transcriptional factor ATF-like 2 | ENSG00000168062 | 1.02 | 1.45E-08 |
| *WNT5A* | Protein Wnt-5a | ENSG00000114251 | 1.02 | 1.13E-22 |
| *NKX2-8* | Homeobox protein Nkx-2.8 | ENSG00000136327 | 1.02 | 7.02E-03 |
| *SDR16C5* | Epidermal retinol dehydrogenase 2 | ENSG00000170786 | 1.02 | 3.82E-05 |
| *FEM1B* | Protein fem-1 homolog B | ENSG00000169018 | 1.02 | 6.29E-51 |
| *SULT2B1* | Sulfotransferase 2B1 | ENSG00000088002 | 1.02 | 0.03 |
| *TMTC1* | Protein O-mannosyl-transferase TMTC1 | ENSG00000133687 | 1.02 | 5.76E-13 |
| *CD46* | Membrane cofactor protein | ENSG00000117335 | 1.02 | 2.75E-23 |
| *SNX22* | Sorting nexin-22 | ENSG00000157734 | 1.02 | 5.20E-03 |
| *BACH1* | Transcription regulator protein BACH1 | ENSG00000156273 | 1.02 | 5.77E-50 |
| *WNT16* | Protein Wnt-16 | ENSG00000002745 | 1.02 | 6.93E-03 |
| *DET1* | DET1 homolog | ENSG00000140543 | 1.01 | 1.41E-03 |
| *LONRF1* | LON peptidase N-terminal domain and RING finger protein 1 | ENSG00000154359 | 1.01 | 1.27E-18 |
| *HLA-A* | HLA class I histocompatibility antigen, A alpha chain | ENSG00000206503 | 1.01 | 5.23E-23 |
| *LTBP3* | Latent-transforming growth factor beta-binding protein 3 | ENSG00000168056 | 1.01 | 3.44E-47 |
| *N4BP2* | NEDD4-binding protein 2 | ENSG00000078177 | 1.01 | 1.75E-20 |
| *KMO* | Kynurenine 3-monooxygenase | ENSG00000117009 | 1.01 | 6.50E-09 |
| *HDAC9* | Histone deacetylase 9 | ENSG00000048052 | 1.01 | 4.18E-50 |
| *CLCN3* | H(+)/Cl(-) exchange transporter 3 | ENSG00000109572 | 1.01 | 2.72E-22 |
| *BTC* | Probetacellulin | ENSG00000174808 | 1.01 | 2.65E-05 |
| *DNAJB2* | DnaJ homolog subfamily B member 2 | ENSG00000135924 | 1.01 | 3.41E-45 |
| *SLC25A45* | Solute carrier family 25 member 45 | ENSG00000162241 | 1.01 | 8.14E-09 |
| *MAML3* | Mastermind-like protein 3 | ENSG00000196782 | 1.01 | 2.51E-05 |
| *MTURN* | Maturin | ENSG00000180354 | 1.01 | 1.69E-21 |
| *ARHGAP31* | Rho GTPase-activating protein 31 | ENSG00000031081 | 1.01 | 0.04 |
| *TMEM140* | Transmembrane protein 140 | ENSG00000146859 | 1 | 6.54E-43 |
| *CPEB2* | Cytoplasmic polyadenylation element-binding protein 2 | ENSG00000137449 | 1 | 8.52E-12 |
| *UGDH* | UDP-glucose 6-dehydrogenase | ENSG00000109814 | 1 | 2.27E-48 |
| *DAPP1* | Dual adapter for phosphotyrosine and 3-phosphotyrosine and 3-phosphoinositide | ENSG00000070190 | 1 | 4.37E-20 |
| *ERP44* | Endoplasmic reticulum resident protein 44 | ENSG00000023318 | 1 | 2.21E-49 |
| *RPS6KA3* | Ribosomal protein S6 kinase alpha-3 | ENSG00000177189 | 1 | 9.06E-49 |
