# Supplementary material for: EMILIN-1 Suppresses Cell Proliferation through Altered Cell Cycle Regulation in Head and Neck Squamous Cell Carcinoma
Source: Am J Pathol. 2025 Jan 30;195(5):995–1012. doi: 10.1016/j.ajpath.2025.01.010 (PMC12163418; doi:10.1016/j.ajpath.2025.01.010)
Supplement: Supplemental Table S4 [file mmc4.docx]

| **Supplemental Table S4** Downregulated genes of CAL27 cell line with EMILIN-1 overexpression (Log2FC<-1,FDR<0.05). (https://www.ensembl.org) | | | |  |  |  |
| --- | --- | --- | --- | --- | --- | --- |
|  |  |  |  |  |  |  |
| **Gene** | **Database name** | **Identifier** | **Log2FC** | **FDR p-value** |  |  |
| *TRIM6-TRIM34* | Tripartite Motif Containing 6-34 | ENSG00000258588 | -9.17 | 3.73E-04 |  |  |
| *AMIGO3* | Amphoterin-induced protein 3 | ENSG00000176020 | -7.71 | 4.07E-03 |  |  |
| *RIMBP3B* | RIMS-binding protein 3B | ENSG00000274600 | -7.19 | 6.63E-03 |  |  |
| *SPDYE17* | Speedy/RINGO Cell Cycle Regulator Family Member E17 | ENSG00000186645 | -7.17 | 7.01E-03 |  |  |
| *ERP27* | Endoplasmic reticulum resident protein 27 | ENSG00000139055 | -6.8 | 0.01 |  |  |
| *GNB3* | Guanine nucleotide-binding protein G(I)/G(S)/G(T) subunit beta-3 | ENSG00000111664 | -6.55 | 0.02 |  |  |
| *SPAAR* | Small regulatory polypeptide of amino acid response | ENSG00000235387 | -6.52 | 0.02 |  |  |
| *KRT74* | Keratin, type II cytoskeletal 74 | ENSG00000170484 | -6.5 | 0.02 |  |  |
| *GALNT5* | Polypeptide N-acetylgalactosaminyltransferase 5 | ENSG00000136542 | -6.29 | 0.02 |  |  |
| *UGT1A5* | UDP-glucuronosyltransferase 1A5 | ENSG00000288705 | -6.2 | 0.03 |  |  |
| *CAPSL* | Calcyphosin-like protein | ENSG00000152611 | -6.17 | 0.03 |  |  |
| *SCN1A* | Sodium channel protein type 1 subunit alpha | ENSG00000144285 | -6.12 | 0.03 |  |  |
| *TBC1D3G* | TBC1 domain family member 3G | ENSG00000260287 | -6.08 | 0.04 |  |  |
| *ASXL3* | Putative Polycomb group protein ASXL3 | ENSG00000141431 | -6.06 | 0.03 |  |  |
| *KCNK10* | Potassium channel subfamily K member 10 | ENSG00000100433 | -6.01 | 0.03 |  |  |
| *RXRG* | Retinoic acid receptor RXR-gamma | ENSG00000143171 | -5.95 | 0.03 |  |  |
| *ZG16* | Zymogen granule membrane protein 16 | ENSG00000174992 | -5.88 | 0.03 |  |  |
| *GALNT16* | Polypeptide N-acetylgalactosaminyltransferase 16 | ENSG00000100626 | -5.52 | 0.05 |  |  |
| *EDN2* | Endothelin-2 | ENSG00000127129 | -5.37 | 5.67E-13 |  |  |
| *CPA4* | Carboxypeptidase A4 | ENSG00000128510 | -5.28 | 5.18E-77 |  |  |
| *FAM178B* | Family With Sequence Similarity 178 Member B | ENSG00000168754 | -4.98 | 5.32E-09 |  |  |
| *METTL7A* | Methyltransferase-like protein 7A | ENSG00000185432 | -4.88 | 8.14E-64 |  |  |
| *CCL2* | C-C motif chemokine 2 | ENSG00000108691 | -4.82 | 3.76E-272 |  |  |
| *KRT1* | Keratin, type II cytoskeletal 1 | ENSG00000167768 | -4.82 | 4.42E-05 |  |  |
| *MEOX1* | Homeobox protein MOX-1 | ENSG00000005102 | -4.82 | 5.73E-05 |  |  |
| *VXN* | Vexin | ENSG00000169085 | -4.51 | 7.43E-07 |  |  |
| *CCR10* | C-C chemokine receptor type 10 | ENSG00000184451 | -4.31 | 4.13E-04 |  |  |
| *ADCY10* | Adenylate cyclase type 10 | ENSG00000143199 | -4.17 | 0.01 |  |  |
| *KRT13* | Keratin, type I cytoskeletal 13 | ENSG00000171401 | -4.11 | 2.24E-67 |  |  |
| *VSNL1* | Visinin-like protein 1 | ENSG00000163032 | -4.09 | 7.06E-94 |  |  |
| *TMEM270* | Transmembrane protein 270 | ENSG00000175877 | -3.97 | 2.83E-03 |  |  |
| *CALML5* | Calmodulin-like protein 5 | ENSG00000178372 | -3.96 | 2.35E-03 |  |  |
| *LRRN4* | Leucine-rich repeat neuronal protein 4 | ENSG00000125872 | -3.9 | 1.81E-16 |  |  |
| *NRGN* | Neurogranin | ENSG00000154146 | -3.84 | 4.50E-04 |  |  |
| *CYB5A* | Cytochrome b5 | ENSG00000166347 | -3.69 | 7.68E-06 |  |  |
| *MAP2K6* | Dual specificity mitogen-activated protein kinase kinase 6 | ENSG00000108984 | -3.67 | 1.35E-57 |  |  |
| *ZNF488* | Zinc finger protein 488 | ENSG00000265763 | -3.67 | 1.10E-89 |  |  |
| *KIF20A* | Kinesin-like protein KIF20A | ENSG00000112984 | -3.66 | 1.42E-127 |  |  |
| *ARMH4* | Armadillo-like helical domain-containing protein 4 | ENSG00000139971 | -3.61 | 2.26E-04 |  |  |
| *SERPINB10* | Serpin B10 | ENSG00000242550 | -3.6 | 1.35E-03 |  |  |
| *KLHL32* | Kelch-like protein 32 | ENSG00000186231 | -3.59 | 0.03 |  |  |
| *KCNJ6* | G protein-activated inward rectifier potassium channel 2 | ENSG00000157542 | -3.57 | 8.22E-07 |  |  |
| *REEP1* | Receptor expression-enhancing protein 1 | ENSG00000068615 | -3.57 | 2.84E-06 |  |  |
| *CDKN2C* | Cyclin-dependent kinase 4 inhibitor C | ENSG00000123080 | -3.56 | 3.98E-66 |  |  |
| *ZAP70* | Tyrosine-protein kinase ZAP-70 | ENSG00000115085 | -3.55 | 9.28E-03 |  |  |
| *PLEKHS1* | Pleckstrin Homology Domain Containing S1 | ENSG00000148735 | -3.54 | 7.38E-18 |  |  |
| *C11orf86* | Chromosome 11 Open Reading Frame 86 | ENSG00000173237 | -3.49 | 3.57E-04 |  |  |
| *PIF1* | ATP-dependent DNA helicase PIF1 | ENSG00000140451 | -3.48 | 1.18E-49 |  |  |
| *ANKRD2* | Ankyrin repeat domain-containing protein 2 | ENSG00000165887 | -3.45 | 5.66E-22 |  |  |
| *NPSR1* | Neuropeptide S receptor | ENSG00000187258 | -3.45 | 0.01 |  |  |
| *WDR38* | WD repeat-containing protein 38 | ENSG00000136918 | -3.44 | 0.01 |  |  |
| *SLC6A16* | Orphan sodium- and chloride-dependent neurotransmitter transporter NTT5 | ENSG00000063127 | -3.44 | 0.01 |  |  |
| *BCO1* | Beta,beta-carotene 15,15'-dioxygenase | ENSG00000135697 | -3.43 | 4.81E-04 |  |  |
| *CERKL* | Ceramide kinase-like protein | ENSG00000188452 | -3.43 | 4.70E-04 |  |  |
| *OR52I2* | Olfactory receptor 52I2 | ENSG00000226288 | -3.4 | 0.05 |  |  |
| *SCRT1* | Transcriptional repressor scratch 1 | ENSG00000261678 | -3.4 | 0.04 |  |  |
| *TEX15* | Testis-expressed protein 15 | ENSG00000133863 | -3.36 | 1.59E-04 |  |  |
| *HMGN5* | High mobility group nucleosome-binding domain-containing protein 5 | ENSG00000198157 | -3.27 | 3.13E-06 |  |  |
| *MYL2* | Myosin regulatory light chain 2, ventricular/cardiac muscle isoform | ENSG00000111245 | -3.27 | 1.89E-03 |  |  |
| *KCNC1* | Potassium voltage-gated channel subfamily C member 1 | ENSG00000129159 | -3.25 | 0.02 |  |  |
| *C1QTNF2* | Complement C1q tumor necrosis factor-related protein 2 | ENSG00000145861 | -3.2 | 3.55E-05 |  |  |
| *SLC5A5* | Sodium/iodide cotransporter | ENSG00000105641 | -3.2 | 8.37E-03 |  |  |
| *ARHGDIB* | Rho GDP-dissociation inhibitor 2 | ENSG00000111348 | -3.19 | 4.71E-78 |  |  |
| *FGF1* | Fibroblast growth factor 1 | ENSG00000113578 | -3.17 | 3.52E-21 |  |  |
| *PLCD4* | 1-phosphatidylinositol 4,5-bisphosphate phosphodiesterase delta-4 | ENSG00000115556 | -3.17 | 4.27E-04 |  |  |
| *TYMS* | Thymidylate synthase | ENSG00000176890 | -3.17 | 9.96E-103 |  |  |
| *SPC24* | Kinetochore protein Spc24 | ENSG00000161888 | -3.16 | 5.84E-86 |  |  |
| *MMP13* | Collagenase 3 | ENSG00000137745 | -3.14 | 1.31E-34 |  |  |
| *SSUH2* | Protein SSUH2 homolog | ENSG00000125046 | -3.14 | 0.03 |  |  |
| *OLFML2B* | Olfactomedin-like protein 2B | ENSG00000162745 | -3.12 | 7.58E-09 |  |  |
| *JPH2* | Junctophilin-2 | ENSG00000149596 | -3.11 | 4.39E-69 |  |  |
| *FXYD2* | Sodium/potassium-transporting ATPase subunit gamma | ENSG00000137731 | -3.1 | 0.02 |  |  |
| *CC2D2B* | Protein CC2D2B | ENSG00000188649 | -3.05 | 0.03 |  |  |
| *CGNL1* | Cingulin-like protein 1 | ENSG00000128849 | -3.05 | 1.49E-10 |  |  |
| *SLC2A5* | Solute carrier family 2, facilitated glucose transporter member 5 | ENSG00000142583 | -3.04 | 1.84E-10 |  |  |
| *SFRP1* | Secreted frizzled-related protein 1 | ENSG00000104332 | -3.03 | 4.67E-05 |  |  |
| *ANLN* | Anillin | ENSG00000011426 | -3.02 | 3.45E-108 |  |  |
| *TENT5B* | Terminal nucleotidyltransferase 5B | ENSG00000158246 | -3.01 | 5.59E-65 |  |  |
| *IQSEC3* | IQ motif and SEC7 domain-containing protein 3 | ENSG00000120645 | -3.01 | 6.93E-03 |  |  |
| *KRTDAP* | Keratinocyte differentiation-associated protein | ENSG00000188508 | -2.98 | 0.04 |  |  |
| *CLDN5* | Claudin-5 | ENSG00000184113 | -2.98 | 0.04 |  |  |
| *DIO2* | Type II iodothyronine deiodinase | ENSG00000211448 | -2.98 | 3.69E-12 |  |  |
| *ZNF385B* | Zinc finger protein 385B | ENSG00000144331 | -2.98 | 0.04 |  |  |
| *TRIM63* | E3 ubiquitin-protein ligase TRIM63 | ENSG00000158022 | -2.97 | 0.03 |  |  |
| *GOLGA8F* | Putative golgin subfamily A member 8F/8G | ENSG00000153684 | -2.97 | 0.04 |  |  |
| *IFNL1* | Interferon lambda-1 | ENSG00000182393 | -2.97 | 2.83E-03 |  |  |
| *IL7R* | Interleukin-7 receptor subunit alpha | ENSG00000168685 | -2.96 | 2.92E-66 |  |  |
| *KLRG1* | Killer cell lectin-like receptor subfamily G member 1 | ENSG00000139187 | -2.96 | 4.85E-04 |  |  |
| *CDCA3* | Cell division cycle-associated protein 3 | ENSG00000111665 | -2.95 | 1.21E-44 |  |  |
| *TMEM269* | Transmembrane protein 269 | ENSG00000274386 | -2.92 | 1.77E-03 |  |  |
| *ANPEP* | Aminopeptidase N | ENSG00000166825 | -2.91 | 9.20E-03 |  |  |
| *CD34* | Hematopoietic progenitor cell antigen CD34 | ENSG00000174059 | -2.9 | 5.05E-17 |  |  |
| *TNNI2* | Troponin I, fast skeletal muscle | ENSG00000130598 | -2.9 | 4.34E-04 |  |  |
| *TNNI3* | Troponin I, cardiac muscle | ENSG00000129991 | -2.89 | 0.03 |  |  |
| *LHX2* | LIM/homeobox protein Lhx2 | ENSG00000106689 | -2.88 | 3.24E-13 |  |  |
| *ZNF502* | Zinc finger protein 502 | ENSG00000196653 | -2.88 | 0.02 |  |  |
| *RDM1* | RAD52 motif-containing protein 1 | ENSG00000278023 | -2.88 | 4.00E-16 |  |  |
| *LMO3* | LIM domain only protein 3 | ENSG00000048540 | -2.87 | 4.80E-05 |  |  |
| *ARHGAP33* | Rho GTPase-activating protein 33 | ENSG00000004777 | -2.86 | 1.22E-41 |  |  |
| *CDC20* | Cell division cycle protein 20 homolog | ENSG00000117399 | -2.85 | 2.31E-95 |  |  |
| *CORO1A* | Coronin-1A | ENSG00000102879 | -2.84 | 2.54E-36 |  |  |
| *ALPP* | Alkaline phosphatase, placental type | ENSG00000163283 | -2.83 | 1.62E-13 |  |  |
| *PCLAF* | PCNA-associated factor | ENSG00000166803 | -2.8 | 1.32E-66 |  |  |
| *GPX3* | Glutathione peroxidase 3 | ENSG00000211445 | -2.8 | 3.73E-13 |  |  |
| *ANXA13* | Annexin A13 | ENSG00000104537 | -2.79 | 1.89E-03 |  |  |
| *ANKRD20A4P* | Ankyrin Repeat Domain 20 Family Member A4, Pseudogene | ENSG00000172014 | -2.79 | 1.57E-04 |  |  |
| *RIMBP3* | RIMS-binding protein 3A | ENSG00000275793 | -2.78 | 1.12E-05 |  |  |
| *POU2AF1* | POU domain class 2-associating factor 1 | ENSG00000110777 | -2.77 | 3.93E-04 |  |  |
| *SOX2* | Transcription factor SOX-2 | ENSG00000181449 | -2.77 | 3.95E-53 |  |  |
| *GTSE1* | G2 and S phase-expressed protein 1 | ENSG00000075218 | -2.77 | 3.98E-53 |  |  |
| *DMC1* | Meiotic recombination protein DMC1/LIM15 homolog | ENSG00000100206 | -2.73 | 1.08E-04 |  |  |
| *LGALS1* | Galectin-1 | ENSG00000100097 | -2.72 | 2.24E-58 |  |  |
| *MXRA5* | Matrix-remodeling-associated protein 5 | ENSG00000101825 | -2.72 | 1.24E-54 |  |  |
| *GPR50* | Melatonin-related receptor | ENSG00000102195 | -2.71 | 0.03 |  |  |
| *DLGAP5* | Disks large-associated protein 5 | ENSG00000126787 | -2.71 | 5.27E-71 |  |  |
| *PROCA1* | Protein PROCA1 | ENSG00000167525 | -2.68 | 2.83E-08 |  |  |
| *FGD3* | FYVE, RhoGEF and PH domain-containing protein 3 | ENSG00000127084 | -2.68 | 1.71E-31 |  |  |
| *KCNN1* | Small conductance calcium-activated potassium channel protein 1 | ENSG00000105642 | -2.67 | 1.48E-03 |  |  |
| *KLHL38* | Kelch-like protein 38 | ENSG00000175946 | -2.66 | 3.35E-03 |  |  |
| *THBS1* | Thrombospondin-1 | ENSG00000137801 | -2.66 | 7.38E-84 |  |  |
| *FGL1* | Fibrinogen-like protein 1 | ENSG00000104760 | -2.64 | 2.94E-07 |  |  |
| *VGLL1* | Transcription cofactor vestigial-like protein 1 | ENSG00000102243 | -2.64 | 2.29E-03 |  |  |
| *PIMREG* | Protein PIMREG | ENSG00000129195 | -2.62 | 4.13E-32 |  |  |
| *MYBL1* | Myb-related protein A | ENSG00000185697 | -2.61 | 3.00E-67 |  |  |
| *SOWAHA* | Sosondowah Ankyrin Repeat Domain Family Member A | ENSG00000198944 | -2.61 | 1.05E-08 |  |  |
| *CENPI* | Centromere protein I | ENSG00000102384 | -2.6 | 8.70E-60 |  |  |
| *TBC1D3I* | TBC1 domain family member 3I | ENSG00000274933 | -2.6 | 6.67E-03 |  |  |
| *E2F8* | Transcription factor E2F8 | ENSG00000129173 | -2.6 | 3.13E-44 |  |  |
| *STON1* | Stonin-1 | ENSG00000243244 | -2.6 | 3.67E-17 |  |  |
| *CCDC74B* | Coiled-Coil Domain Containing 74B | ENSG00000152076 | -2.59 | 3.39E-08 |  |  |
| *ANKRD65* | Ankyrin Repeat Domain 65 | ENSG00000235098 | -2.58 | 4.30E-40 |  |  |
| *FGF22* | Fibroblast growth factor 22 | ENSG00000070388 | -2.56 | 0.02 |  |  |
| *RBFOX3* | RNA binding protein fox-1 homolog 3 | ENSG00000167281 | -2.56 | 0.02 |  |  |
| *AHRR* | Aryl hydrocarbon receptor repressor | ENSG00000063438 | -2.56 | 8.59E-03 |  |  |
| *RASGRP2* | RAS guanyl-releasing protein 2 | ENSG00000068831 | -2.56 | 1.42E-09 |  |  |
| *KIFC1* | Kinesin-like protein KIFC1 | ENSG00000237649 | -2.55 | 2.60E-59 |  |  |
| *PRCD* | Photoreceptor disk component PRCD | ENSG00000214140 | -2.55 | 0.04 |  |  |
| *RCSD1* | CapZ-interacting protein | ENSG00000198771 | -2.55 | 3.89E-42 |  |  |
| *TCAF2* | TRPM8 channel-associated factor 2 | ENSG00000170379 | -2.53 | 2.60E-48 |  |  |
| *SCARA3* | Scavenger receptor class A member 3 | ENSG00000168077 | -2.52 | 8.40E-12 |  |  |
| *PRB3* | Basic salivary proline-rich protein 3 | ENSG00000197870 | -2.52 | 1.39E-03 |  |  |
| *ACBD7* | Acyl-CoA-binding domain-containing protein 7 | ENSG00000176244 | -2.51 | 4.78E-30 |  |  |
| *STPG3* | Protein STPG3 | ENSG00000197768 | -2.51 | 0.05 |  |  |
| *PTGFR* | Prostaglandin F2-alpha receptor | ENSG00000122420 | -2.5 | 0.03 |  |  |
| *CCDC197* | Uncharacterized protein CCDC197 | ENSG00000175699 | -2.5 | 7.21E-04 |  |  |
| *FBXO43* | F-box only protein 43 | ENSG00000156509 | -2.5 | 7.17E-11 |  |  |
| *NPR1* | Atrial natriuretic peptide receptor 1 | ENSG00000169418 | -2.5 | 4.01E-03 |  |  |
| *ANK1* | Ankyrin-1 | ENSG00000029534 | -2.5 | 1.72E-14 |  |  |
| *KCNK12* | Potassium channel subfamily K member 12 | ENSG00000184261 | -2.49 | 9.66E-05 |  |  |
| *LZTS1* | Leucine zipper putative tumor suppressor 1 | ENSG00000061337 | -2.49 | 0.04 |  |  |
| *STMN1* | Stathmin | ENSG00000117632 | -2.48 | 1.43E-69 |  |  |
| *SH3BGRL* | SH3 domain-binding glutamic acid-rich-like protein | ENSG00000131171 | -2.48 | 1.21E-04 |  |  |
| *EFEMP1* | EGF-containing fibulin-like extracellular matrix protein 1 | ENSG00000115380 | -2.47 | 1.56E-57 |  |  |
| *TK1* | Thymidine kinase, cytosolic | ENSG00000167900 | -2.47 | 3.17E-41 |  |  |
| *HTRA3* | Serine protease HTRA3 | ENSG00000170801 | -2.46 | 2.95E-38 |  |  |
| *CCNA2* | Cyclin-A2 | ENSG00000145386 | -2.45 | 1.44E-74 |  |  |
| *IL16* | Pro-interleukin-16 | ENSG00000172349 | -2.45 | 1.41E-06 |  |  |
| *KRT14* | Keratin, type I cytoskeletal 14 | ENSG00000186847 | -2.45 | 2.60E-92 |  |  |
| *ORC1* | Origin recognition complex subunit 1 | ENSG00000085840 | -2.44 | 1.65E-102 |  |  |
| *IGFL1* | Insulin growth factor-like family member 1 | ENSG00000188293 | -2.44 | 4.86E-11 |  |  |
| *IPCEF1* | Interactor protein for cytohesin exchange factors 1 | ENSG00000074706 | -2.44 | 8.85E-03 |  |  |
| *P2RY6* | P2Y purinoceptor 6 | ENSG00000171631 | -2.44 | 1.36E-35 |  |  |
| *C1QTNF3* | Complement C1q tumor necrosis factor-related protein 3 | ENSG00000082196 | -2.44 | 0.04 |  |  |
| *DPF1* | Zinc finger protein neuro-d4 | ENSG00000011332 | -2.43 | 1.05E-14 |  |  |
| *BUB1* | Mitotic checkpoint serine/threonine-protein kinase BUB1 | ENSG00000169679 | -2.42 | 2.48E-66 |  |  |
| *ERCC6L* | DNA excision repair protein ERCC-6-like | ENSG00000186871 | -2.42 | 8.25E-49 |  |  |
| *NODAL* | Nodal homolog | ENSG00000156574 | -2.41 | 0.03 |  |  |
| *ALOX15* | Polyunsaturated fatty acid lipoxygenase ALOX15 | ENSG00000161905 | -2.41 | 2.54E-12 |  |  |
| *OLFML2A* | Olfactomedin-like protein 2A | ENSG00000185585 | -2.4 | 3.77E-57 |  |  |
| *HMGB1P1* | Putative high mobility group protein B1-like 1 | ENSG00000124097 | -2.4 | 6.15E-03 |  |  |
| *SPAG5* | Sperm-associated antigen 5 | ENSG00000076382 | -2.4 | 3.71E-59 |  |  |
| *MYLK* | Myosin light chain kinase, smooth muscle | ENSG00000065534 | -2.39 | 5.29E-53 |  |  |
| *RNF222* | RING finger protein 222 | ENSG00000189051 | -2.39 | 0.05 |  |  |
| *CXCL9* | C-X-C motif chemokine 9 | ENSG00000138755 | -2.38 | 0.05 |  |  |
| *NRN1* | Neuritin | ENSG00000124785 | -2.38 | 0.01 |  |  |
| *KRT5* | Keratin, type II cytoskeletal 5 | ENSG00000186081 | -2.37 | 1.16E-61 |  |  |
| *KRT4* | Keratin, type II cytoskeletal 4 | ENSG00000170477 | -2.37 | 7.30E-11 |  |  |
| *KIF18B* | Kinesin-like protein KIF18B | ENSG00000186185 | -2.36 | 1.84E-58 |  |  |
| *RAB4B-EGLN2* | RAB4B-EGLN2 readthrough | ENSG00000171570 | -2.36 | 0.01 |  |  |
| *TOP2A* | DNA topoisomerase 2-alpha | ENSG00000131747 | -2.36 | 3.53E-56 |  |  |
| *TP73* | Tumor protein p73 | ENSG00000078900 | -2.35 | 8.60E-85 |  |  |
| *AMOT* | Angiomotin | ENSG00000126016 | -2.35 | 1.24E-06 |  |  |
| *HJURP* | Holliday junction recognition protein | ENSG00000123485 | -2.34 | 3.28E-43 |  |  |
| *RAB3A* | Ras-related protein Rab-3A | ENSG00000105649 | -2.34 | 4.28E-21 |  |  |
| *FAM83D* | Protein FAM83D | ENSG00000101447 | -2.34 | 2.04E-65 |  |  |
| *FAM72B* | Protein FAM72B | ENSG00000188610 | -2.33 | 1.36E-66 |  |  |
| *ITM2A* | Integral membrane protein 2A | ENSG00000078596 | -2.33 | 0.03 |  |  |
| *CAND2* | Cullin-associated NEDD8-dissociated protein 2 | ENSG00000144712 | -2.33 | 0.03 |  |  |
| *LMNB1* | Lamin-B1 | ENSG00000113368 | -2.33 | 4.40E-113 |  |  |
| *FAM111B* | Serine protease FAM111B | ENSG00000189057 | -2.32 | 2.43E-98 |  |  |
| *RAB7B* | Ras-related protein Rab-7b | ENSG00000276600 | -2.31 | 4.59E-34 |  |  |
| *PBK* | Lymphokine-activated killer T-cell-originated protein kinase | ENSG00000168078 | -2.31 | 3.25E-44 |  |  |
| *NCAPG* | Condensin complex subunit 3 | ENSG00000109805 | -2.31 | 4.01E-52 |  |  |
| *MYBL2* | Myb-related protein B | ENSG00000101057 | -2.31 | 7.83E-47 |  |  |
| *PDZK1* | Na(+)/H(+) exchange regulatory cofactor NHE-RF3 | ENSG00000174827 | -2.3 | 6.50E-06 |  |  |
| *LUM* | Lumican | ENSG00000139329 | -2.3 | 8.05E-12 |  |  |
| *GAS2L3* | GAS2-like protein 3 | ENSG00000139354 | -2.29 | 1.02E-92 |  |  |
| *MND1* | Meiotic nuclear division protein 1 homolog | ENSG00000121211 | -2.29 | 1.46E-21 |  |  |
| *MKI67* | Proliferation marker protein Ki-67 | ENSG00000148773 | -2.29 | 7.17E-45 |  |  |
| *SLC6A12* | Sodium- and chloride-dependent betaine transporter | ENSG00000111181 | -2.29 | 6.27E-09 |  |  |
| *SPC25* | Kinetochore protein Spc25 | ENSG00000152253 | -2.28 | 7.25E-37 |  |  |
| *PSRC1* | Proline/serine-rich coiled-coil protein 1 | ENSG00000134222 | -2.28 | 4.02E-53 |  |  |
| *HRCT1* | Histidine-rich carboxyl terminus protein 1 | ENSG00000196196 | -2.28 | 0.04 |  |  |
| *ASPM* | Abnormal spindle-like microcephaly-associated protein | ENSG00000066279 | -2.27 | 2.86E-106 |  |  |
| *ESPL1* | Separin | ENSG00000135476 | -2.27 | 4.83E-49 |  |  |
| *RAD54L* | DNA repair and recombination protein RAD54-like | ENSG00000085999 | -2.27 | 6.00E-48 |  |  |
| *RNF112* | RING finger protein 112 | ENSG00000128482 | -2.27 | 1.14E-07 |  |  |
| *C21orf58* | Chromosome 21 Open Reading Frame 58 | ENSG00000160298 | -2.26 | 1.82E-34 |  |  |
| *SALL2* | Sal-like protein 2 | ENSG00000165821 | -2.25 | 9.67E-08 |  |  |
| *ITGA2B* | Integrin alpha-IIb | ENSG00000005961 | -2.25 | 5.60E-05 |  |  |
| *CENPF* | Centromere protein F | ENSG00000117724 | -2.25 | 6.62E-67 |  |  |
| *BUB1B* | Mitotic checkpoint serine/threonine-protein kinase BUB1 beta | ENSG00000156970 | -2.24 | 5.41E-102 |  |  |
| *BNC2* | Zinc finger protein basonuclin-2 | ENSG00000173068 | -2.24 | 1.52E-03 |  |  |
| *SIX2* | Homeobox protein SIX2 | ENSG00000170577 | -2.24 | 1.67E-10 |  |  |
| *LIMS2* | LIM and senescent cell antigen-like-containing domain protein 2 | ENSG00000072163 | -2.24 | 3.54E-07 |  |  |
| *PCDH10* | Protocadherin-10 | ENSG00000138650 | -2.24 | 1.84E-03 |  |  |
| *ADAMTS15* | A disintegrin and metalloproteinase with thrombospondin motifs 15 | ENSG00000166106 | -2.24 | 3.15E-07 |  |  |
| *RIPOR3* | RIPOR family member 3 | ENSG00000042062 | -2.23 | 7.20E-94 |  |  |
| *GPR19* | Probable G-protein coupled receptor 19 | ENSG00000183150 | -2.23 | 4.01E-03 |  |  |
| *CDKN3* | Cyclin-dependent kinase inhibitor 3 | ENSG00000100526 | -2.23 | 3.06E-48 |  |  |
| *KNL1* | Kinetochore scaffold 1 | ENSG00000137812 | -2.23 | 3.82E-65 |  |  |
| *RRM2* | Ribonucleoside-diphosphate reductase subunit M2 | ENSG00000171848 | -2.23 | 1.74E-48 |  |  |
| *NUF2* | Kinetochore protein Nuf2 | ENSG00000143228 | -2.23 | 1.80E-87 |  |  |
| *CNTN1* | Contactin-1 | ENSG00000018236 | -2.22 | 1.75E-34 |  |  |
| *ASRGL1* | Isoaspartyl peptidase/L-asparaginase | ENSG00000162174 | -2.22 | 5.21E-07 |  |  |
| *MESP2* | Mesoderm posterior protein 2 | ENSG00000188095 | -2.22 | 8.62E-08 |  |  |
| *ANGPT1* | Angiopoietin-1 | ENSG00000154188 | -2.22 | 1.83E-19 |  |  |
| *CXCL14* | C-X-C motif chemokine 14 | ENSG00000145824 | -2.21 | 8.33E-05 |  |  |
| *HCAR2* | Hydroxycarboxylic acid receptor 2 | ENSG00000182782 | -2.21 | 1.46E-78 |  |  |
| *NUSAP1* | Nucleolar and spindle-associated protein 1 | ENSG00000137804 | -2.21 | 9.27E-72 |  |  |
| *PRC1* | Protein regulator of cytokinesis 1 | ENSG00000198901 | -2.2 | 4.39E-77 |  |  |
| *CENPE* | Centromere-associated protein E | ENSG00000138778 | -2.2 | 3.57E-47 |  |  |
| *HCAR3* | Hydroxycarboxylic acid receptor 3 | ENSG00000255398 | -2.2 | 7.14E-33 |  |  |
| *CD101* | Immunoglobulin superfamily member 2 | ENSG00000134256 | -2.19 | 1.25E-08 |  |  |
| *DNA2* | DNA replication ATP-dependent helicase/nuclease DNA2 | ENSG00000138346 | -2.19 | 9.75E-83 |  |  |
| *ESCO2* | N-acetyltransferase ESCO2 | ENSG00000171320 | -2.18 | 1.09E-32 |  |  |
| *FKBP1B* | Peptidyl-prolyl cis-trans isomerase FKBP1B | ENSG00000119782 | -2.18 | 0.01 |  |  |
| *KIF5C* | Kinesin heavy chain isoform 5C | ENSG00000168280 | -2.18 | 4.39E-09 |  |  |
| *NCAPH* | Condensin complex subunit 2 | ENSG00000121152 | -2.18 | 6.95E-92 |  |  |
| *CTSV* | Cathepsin L2 | ENSG00000136943 | -2.18 | 2.08E-44 |  |  |
| *OIP5* | Protein Mis18-beta | ENSG00000104147 | -2.18 | 1.46E-34 |  |  |
| *BIRC5* | Baculoviral IAP repeat-containing protein 5 | ENSG00000089685 | -2.18 | 6.52E-47 |  |  |
| *MYB* | Transcriptional activator Myb | ENSG00000118513 | -2.17 | 1.72E-25 |  |  |
| *INCENP* | Inner centromere protein | ENSG00000149503 | -2.17 | 9.38E-96 |  |  |
| *PTTG1* | Securin | ENSG00000164611 | -2.16 | 7.39E-65 |  |  |
| *CIT* | Citron Rho-interacting kinase | ENSG00000122966 | -2.16 | 5.45E-49 |  |  |
| *KIF4B* | Chromosome-associated kinesin KIF4B | ENSG00000226650 | -2.16 | 8.15E-03 |  |  |
| *SGO2* | Shugoshin 2 | ENSG00000163535 | -2.15 | 5.93E-63 |  |  |
| *PRR11* | Proline-rich protein 11 | ENSG00000068489 | -2.15 | 4.83E-97 |  |  |
| *TAGLN3* | Transgelin-3 | ENSG00000144834 | -2.13 | 5.83E-03 |  |  |
| *CCNB1* | G2/mitotic-specific cyclin-B1 | ENSG00000134057 | -2.13 | 2.27E-96 |  |  |
| *ASF1B* | Histone chaperone ASF1B | ENSG00000105011 | -2.13 | 5.33E-52 |  |  |
| *HELLS* | Lymphoid-specific helicase | ENSG00000119969 | -2.13 | 2.88E-89 |  |  |
| *CDK1* | Cyclin-dependent kinase 1 | ENSG00000170312 | -2.12 | 6.51E-55 |  |  |
| *CCNF* | Cyclin-F | ENSG00000162063 | -2.12 | 4.43E-53 |  |  |
| *UGT1A1* | UDP-glucuronosyltransferase 1A1 | ENSG00000241635 | -2.11 | 3.94E-03 |  |  |
| *UBE2T* | Ubiquitin-conjugating enzyme E2 T | ENSG00000077152 | -2.11 | 1.75E-85 |  |  |
| *FAM72D* | Protein FAM72D | ENSG00000215784 | -2.11 | 4.91E-60 |  |  |
| *PLK1* | Serine/threonine-protein kinase PLK1 | ENSG00000166851 | -2.11 | 1.79E-38 |  |  |
| *LAG3* | Lymphocyte activation gene 3 protein | ENSG00000089692 | -2.11 | 0.02 |  |  |
| *ASB9* | Ankyrin repeat and SOCS box protein 9 | ENSG00000102048 | -2.1 | 4.83E-11 |  |  |
| *MAFA* | Transcription factor MafA | ENSG00000182759 | -2.1 | 7.20E-04 |  |  |
| *FBXO24* | F-box only protein 24 | ENSG00000106336 | -2.1 | 2.15E-03 |  |  |
| *SULT1A3* | Sulfotransferase 1A3 | ENSG00000261052 | -2.1 | 4.44E-88 |  |  |
| *ADAM11* | Disintegrin and metalloproteinase domain-containing protein 11 | ENSG00000073670 | -2.1 | 1.08E-08 |  |  |
| *ZBTB7C* | Zinc finger and BTB domain-containing protein 7C | ENSG00000184828 | -2.1 | 3.44E-03 |  |  |
| *NEK2* | Serine/threonine-protein kinase Nek2 | ENSG00000117650 | -2.1 | 1.10E-43 |  |  |
| *VWF* | von Willebrand factor | ENSG00000110799 | -2.1 | 7.70E-08 |  |  |
| *SOX18* | Transcription factor SOX-18 | ENSG00000203883 | -2.09 | 1.52E-06 |  |  |
| *CDH5* | Cadherin-5 | ENSG00000179776 | -2.09 | 2.46E-45 |  |  |
| *KIF14* | Kinesin-like protein KIF14 | ENSG00000118193 | -2.09 | 4.58E-47 |  |  |
| *SYT8* | Synaptotagmin-8 | ENSG00000149043 | -2.09 | 8.98E-11 |  |  |
| *UBE2C* | Ubiquitin-conjugating enzyme E2 C | ENSG00000175063 | -2.09 | 3.69E-59 |  |  |
| *AURKB* | Aurora kinase B | ENSG00000178999 | -2.08 | 3.30E-31 |  |  |
| *IL2RB* | Interleukin-2 receptor subunit beta | ENSG00000100385 | -2.08 | 6.22E-03 |  |  |
| *CEACAM5* | Carcinoembryonic antigen-related cell adhesion molecule 5 | ENSG00000105388 | -2.08 | 8.21E-05 |  |  |
| *TACC3* | Transforming acidic coiled-coil-containing protein 3 | ENSG00000013810 | -2.08 | 2.25E-74 |  |  |
| *CCNB2* | G2/mitotic-specific cyclin-B2 | ENSG00000157456 | -2.08 | 1.07E-88 |  |  |
| *NEIL3* | Endonuclease 8-like 3 | ENSG00000109674 | -2.08 | 2.66E-27 |  |  |
| *TP53AIP1* | p53-regulated apoptosis-inducing protein 1 | ENSG00000120471 | -2.08 | 7.42E-10 |  |  |
| *CKAP2L* | Cytoskeleton-associated protein 2-like | ENSG00000169607 | -2.08 | 6.08E-88 |  |  |
| *LYNX1* | Ly-6/neurotoxin-like protein 1 | ENSG00000180155 | -2.08 | 6.55E-04 |  |  |
| *SPTA1* | Spectrin alpha chain, erythrocytic 1 | ENSG00000163554 | -2.08 | 0.05 |  |  |
| *TNFAIP8L1* | Tumor necrosis factor alpha-induced protein 8-like protein 1 | ENSG00000185361 | -2.07 | 2.14E-56 |  |  |
| *TICRR* | Treslin | ENSG00000140534 | -2.07 | 2.53E-58 |  |  |
| *C16orf86* | Chromosome 16 Open Reading Frame 86 | ENSG00000159761 | -2.06 | 1.50E-03 |  |  |
| *FAM72C* | Protein FAM72C | ENSG00000263513 | -2.06 | 8.85E-73 |  |  |
| *LIG1* | DNA ligase 1 | ENSG00000105486 | -2.06 | 5.01E-84 |  |  |
| *CDC25C* | M-phase inducer phosphatase 3 | ENSG00000158402 | -2.06 | 6.17E-35 |  |  |
| *IL20* | Interleukin-20 | ENSG00000162891 | -2.06 | 6.16E-10 |  |  |
| *GINS1* | DNA replication complex GINS protein PSF1 | ENSG00000101003 | -2.06 | 4.78E-81 |  |  |
| *KIF4A* | Chromosome-associated kinesin KIF4A | ENSG00000090889 | -2.06 | 4.41E-37 |  |  |
| *SCG2* | Secretogranin-2 | ENSG00000171951 | -2.06 | 0.01 |  |  |
| *CPT1C* | Carnitine O-palmitoyltransferase 1, brain isoform | ENSG00000169169 | -2.06 | 8.69E-03 |  |  |
| *ERC2* | ERC protein 2 | ENSG00000187672 | -2.05 | 0.04 |  |  |
| *TMEM255A* | Transmembrane protein 255A | ENSG00000125355 | -2.05 | 1.53E-24 |  |  |
| *ARHGEF39* | Rho guanine nucleotide exchange factor 39 | ENSG00000137135 | -2.05 | 1.76E-43 |  |  |
| *POLE2* | DNA polymerase epsilon subunit 2 | ENSG00000100479 | -2.05 | 5.54E-42 |  |  |
| *MCM10* | Protein MCM10 homolog | ENSG00000065328 | -2.04 | 3.48E-38 |  |  |
| *ZWINT* | ZW10 interactor | ENSG00000122952 | -2.04 | 2.20E-62 |  |  |
| *NRM* | Nurim | ENSG00000137404 | -2.04 | 4.97E-35 |  |  |
| *ARHGAP11B* | Inactive Rho GTPase-activating protein 11B | ENSG00000285077 | -2.04 | 2.16E-13 |  |  |
| *TCF19* | Transcription factor 19 | ENSG00000137310 | -2.04 | 5.08E-82 |  |  |
| *RIMS3* | Regulating synaptic membrane exocytosis protein 3 | ENSG00000117016 | -2.04 | 1.14E-31 |  |  |
| *DEPDC1B* | DEP domain-containing protein 1B | ENSG00000035499 | -2.03 | 1.50E-45 |  |  |
| *KIF11* | Kinesin-like protein KIF11 | ENSG00000138160 | -2.03 | 6.04E-54 |  |  |
| *ENTPD8* | Ectonucleoside triphosphate diphosphohydrolase 8 | ENSG00000188833 | -2.03 | 4.25E-05 |  |  |
| *RBM24* | RNA-binding protein 24 | ENSG00000112183 | -2.03 | 5.36E-22 |  |  |
| *FAP* | Prolyl endopeptidase FAP | ENSG00000078098 | -2.03 | 0.04 |  |  |
| *NCAPD2* | Condensin complex subunit 1 | ENSG00000010292 | -2.03 | 4.82E-88 |  |  |
| *APOL4* | Apolipoprotein L4 | ENSG00000100336 | -2.02 | 1.55E-05 |  |  |
| *ZNF670-ZNF695* | ZNF670-ZNF695 readthrough | ENSG00000135747 | -2.02 | 0.05 |  |  |
| *CCDC74A* | Coiled-coil domain-containing protein 74A | ENSG00000163040 | -2.02 | 2.81E-05 |  |  |
| *GINS4* | DNA replication complex GINS protein SLD5 | ENSG00000147536 | -2.02 | 3.03E-57 |  |  |
| *HMGB2* | High mobility group protein B2 | ENSG00000164104 | -2.01 | 2.30E-84 |  |  |
| *FGFBP1* | Fibroblast growth factor-binding protein 1 | ENSG00000137440 | -2.01 | 1.73E-84 |  |  |
| *PKMYT1* | Membrane-associated tyrosine- and threonine-specific cdc2-inhibitory kinase | ENSG00000127564 | -2 | 1.62E-51 |  |  |
| *KIF15* | Kinesin-like protein KIF15 | ENSG00000163808 | -2 | 9.13E-36 |  |  |
| *CDCA2* | Cell division cycle-associated protein 2 | ENSG00000184661 | -2 | 1.47E-45 |  |  |
| *MAB21L1* | Putative nucleotidyltransferase MAB21L1 | ENSG00000180660 | -2 | 1.43E-11 |  |  |
| *KRT32* | Keratin, type I cuticular Ha2 | ENSG00000108759 | -2 | 7.39E-03 |  |  |
| *GPR20* | G-protein coupled receptor 20 | ENSG00000204882 | -2 | 0.01 |  |  |
| *CAMK2A* | Calcium/calmodulin-dependent protein kinase type II subunit alpha | ENSG00000070808 | -2 | 1.13E-15 |  |  |
| *CDH23* | Cadherin-23 | ENSG00000107736 | -1.99 | 2.05E-15 |  |  |
| *SUSD2* | Sushi domain-containing protein 2 | ENSG00000099994 | -1.99 | 2.11E-19 |  |  |
| *CLSPN* | Claspin | ENSG00000092853 | -1.99 | 3.42E-75 |  |  |
| *CAV1* | Caveolin-1 | ENSG00000105974 | -1.98 | 1.45E-85 |  |  |
| *TUBB1* | Tubulin beta-1 chain | ENSG00000101162 | -1.98 | 2.98E-04 |  |  |
| *CENPA* | Histone H3-like centromeric protein A | ENSG00000115163 | -1.98 | 5.53E-36 |  |  |
| *PRR20G* | Proline Rich 20G | ENSG00000239620 | -1.98 | 0.05 |  |  |
| *ANKRD20A2P* | Ankyrin Repeat Domain 20 Family Member A2, Pseudogene | ENSG00000183148 | -1.98 | 2.81E-03 |  |  |
| *C18orf54* | Chromosome 18 Open Reading Frame 54 | ENSG00000166845 | -1.98 | 8.48E-22 |  |  |
| *TRHDE* | Thyrotropin-releasing hormone-degrading ectoenzyme | ENSG00000072657 | -1.97 | 0.01 |  |  |
| *P3H2* | Prolyl 3-hydroxylase 2 | ENSG00000090530 | -1.97 | 2.32E-79 |  |  |
| *CCBE1* | Collagen and calcium-binding EGF domain-containing protein 1 | ENSG00000183287 | -1.96 | 3.92E-25 |  |  |
| *MAD2L1* | Mitotic spindle assembly checkpoint protein MAD2A | ENSG00000164109 | -1.96 | 1.06E-74 |  |  |
| *ACAT2* | Acetyl-CoA acetyltransferase, cytosolic | ENSG00000120437 | -1.96 | 2.49E-80 |  |  |
| *HMMR* | Hyaluronan mediated motility receptor | ENSG00000072571 | -1.96 | 5.15E-53 |  |  |
| *AURKA* | Aurora kinase A | ENSG00000087586 | -1.95 | 2.39E-80 |  |  |
| *KCNK3* | Potassium channel subfamily K member 3 | ENSG00000171303 | -1.95 | 2.02E-04 |  |  |
| *IL22RA1* | Interleukin-22 receptor subunit alpha-1 | ENSG00000142677 | -1.95 | 1.04E-20 |  |  |
| *SHCBP1* | SHC SH2 domain-binding protein 1 | ENSG00000171241 | -1.95 | 5.14E-75 |  |  |
| *HSPA2* | Heat shock-related 70 kDa protein 2 | ENSG00000126803 | -1.94 | 1.85E-142 |  |  |
| *RECQL4* | ATP-dependent DNA helicase Q4 | ENSG00000160957 | -1.94 | 3.15E-48 |  |  |
| *KREMEN2* | Kremen protein 2 | ENSG00000131650 | -1.94 | 3.17E-73 |  |  |
| *CXorf49B* | Chromosome X Open Reading Frame 49B | ENSG00000215113 | -1.93 | 6.72E-03 |  |  |
| *CNPY4* | Protein canopy homolog 4 | ENSG00000166997 | -1.93 | 1.16E-24 |  |  |
| *ARSI* | Arylsulfatase I | ENSG00000183876 | -1.93 | 5.84E-75 |  |  |
| *TPX2* | Targeting protein for Xklp2 | ENSG00000088325 | -1.93 | 1.43E-79 |  |  |
| *MAB21L4* | Mab-21 Like 4 | ENSG00000172478 | -1.93 | 1.94E-07 |  |  |
| *KIAA1549L* | UPF0606 protein KIAA1549L | ENSG00000110427 | -1.92 | 1.09E-04 |  |  |
| *CDCA8* | Borealin | ENSG00000134690 | -1.92 | 2.46E-36 |  |  |
| *CEP55* | Centrosomal protein of 55 kDa | ENSG00000138180 | -1.92 | 2.62E-41 |  |  |
| *TGM5* | Protein-glutamine gamma-glutamyltransferase 5 | ENSG00000104055 | -1.92 | 1.81E-04 |  |  |
| *CENPK* | Centromere protein K | ENSG00000123219 | -1.92 | 1.93E-68 |  |  |
| *HSPA1L* | Heat shock 70 kDa protein 1-like | ENSG00000204390 | -1.92 | 7.03E-12 |  |  |
| *RIMBP2* | RIMS-binding protein 2 | ENSG00000060709 | -1.92 | 1.92E-05 |  |  |
| *LTB* | Lymphotoxin-beta | ENSG00000227507 | -1.92 | 2.25E-03 |  |  |
| *ITGB6* | Integrin beta-6 | ENSG00000115221 | -1.91 | 4.87E-168 |  |  |
| *C1QTNF6* | Complement C1q tumor necrosis factor-related protein 6 | ENSG00000133466 | -1.9 | 1.27E-69 |  |  |
| *OXCT2* | Succinyl-CoA:3-ketoacid coenzyme A transferase 2, mitochondrial | ENSG00000198754 | -1.9 | 0.01 |  |  |
| *HLA-DMB* | HLA class II histocompatibility antigen, DM beta chain | ENSG00000242574 | -1.9 | 8.41E-27 |  |  |
| *IGFLR1* | IGF-like family receptor 1 | ENSG00000126246 | -1.9 | 5.65E-08 |  |  |
| *CDC45* | Cell division control protein 45 homolog | ENSG00000093009 | -1.9 | 4.03E-70 |  |  |
| *MTMR11* | Myotubularin-related protein 11 | ENSG00000014914 | -1.89 | 1.61E-20 |  |  |
| *SKIDA1* | SKI/DACH Domain Containing 1 | ENSG00000180592 | -1.89 | 4.05E-04 |  |  |
| *PADI3* | Protein-arginine deiminase type-3 | ENSG00000142619 | -1.88 | 1.80E-26 |  |  |
| *MAMDC2* | MAM domain-containing protein 2 | ENSG00000165072 | -1.88 | 1.24E-21 |  |  |
| *CHRNA3* | Neuronal acetylcholine receptor subunit alpha-3 | ENSG00000080644 | -1.88 | 0.03 |  |  |
| *TTK* | Dual specificity protein kinase TTK | ENSG00000112742 | -1.88 | 6.82E-71 |  |  |
| *SIX3* | Homeobox protein SIX3 | ENSG00000138083 | -1.88 | 0.01 |  |  |
| *HMSD* | Serpin-like protein HMSD | ENSG00000221887 | -1.88 | 2.10E-05 |  |  |
| *GINS2* | DNA replication complex GINS protein PSF2 | ENSG00000131153 | -1.87 | 1.05E-43 |  |  |
| *HMCN1* | Hemicentin-1 | ENSG00000143341 | -1.86 | 3.29E-03 |  |  |
| *PSG8* | Pregnancy-specific beta-1-glycoprotein 8 | ENSG00000124467 | -1.86 | 4.05E-04 |  |  |
| *NUDT1* | Oxidized purine nucleoside triphosphate hydrolase | ENSG00000106268 | -1.86 | 1.92E-41 |  |  |
| *RRM1* | Ribonucleoside-diphosphate reductase large subunit | ENSG00000167325 | -1.86 | 5.59E-74 |  |  |
| *COCH* | Cochlin | ENSG00000100473 | -1.86 | 2.47E-06 |  |  |
| *NXPH3* | Neurexophilin-3 | ENSG00000182575 | -1.86 | 9.84E-04 |  |  |
| *UHRF1* | E3 ubiquitin-protein ligase UHRF1 | ENSG00000276043 | -1.86 | 7.82E-72 |  |  |
| *FAM72A* | Protein FAM72A | ENSG00000196550 | -1.85 | 2.62E-61 |  |  |
| *ZNF608* | Zinc finger protein 608 | ENSG00000168916 | -1.85 | 6.34E-66 |  |  |
| *POLD1* | DNA polymerase delta catalytic subunit | ENSG00000062822 | -1.84 | 2.60E-148 |  |  |
| *CENPM* | Centromere protein M | ENSG00000100162 | -1.84 | 2.62E-25 |  |  |
| *TG* | Thyroglobulin | ENSG00000042832 | -1.84 | 1.69E-04 |  |  |
| *ZGRF1* | Protein ZGRF1 | ENSG00000138658 | -1.84 | 2.47E-26 |  |  |
| *SESN3* | Sestrin-3 | ENSG00000149212 | -1.84 | 1.02E-58 |  |  |
| *SEMA6B* | Semaphorin-6B | ENSG00000167680 | -1.84 | 5.42E-37 |  |  |
| *RBP3* | Retinol-binding protein 3 | ENSG00000265203 | -1.84 | 0.04 |  |  |
| *EXO1* | Exonuclease 1 | ENSG00000174371 | -1.84 | 1.51E-48 |  |  |
| *CLCA2* | Calcium-activated chloride channel regulator 2 | ENSG00000137975 | -1.83 | 1.39E-21 |  |  |
| *SKP2* | S-phase kinase-associated protein 2 | ENSG00000145604 | -1.83 | 5.45E-148 |  |  |
| *REXO5* | RNA exonuclease 5 | ENSG00000005189 | -1.83 | 8.13E-22 |  |  |
| *VAV3* | Guanine nucleotide exchange factor VAV3 | ENSG00000134215 | -1.83 | 9.19E-65 |  |  |
| *MXRA8* | Matrix remodeling-associated protein 8 | ENSG00000162576 | -1.83 | 8.47E-05 |  |  |
| *FOXM1* | Forkhead box protein M1 | ENSG00000111206 | -1.82 | 2.81E-27 |  |  |
| *FSD1* | Fibronectin type III and SPRY domain-containing protein 1 | ENSG00000105255 | -1.82 | 2.60E-03 |  |  |
| *SLC16A2* | Monocarboxylate transporter 8 | ENSG00000147100 | -1.82 | 2.43E-65 |  |  |
| *ATP1B2* | Sodium/potassium-transporting ATPase subunit beta-2 | ENSG00000129244 | -1.81 | 0.04 |  |  |
| *AGBL3* | Cytosolic carboxypeptidase 3 | ENSG00000146856 | -1.81 | 1.94E-04 |  |  |
| *ODAD1* | Outer dynein arm-docking complex subunit 1 | ENSG00000105479 | -1.81 | 8.10E-03 |  |  |
| *NCAPG2* | Condensin-2 complex subunit G2 | ENSG00000146918 | -1.81 | 8.76E-68 |  |  |
| *POLA2* | DNA polymerase alpha subunit B | ENSG00000014138 | -1.81 | 5.24E-66 |  |  |
| *WDR76* | WD repeat-containing protein 76 | ENSG00000092470 | -1.8 | 4.92E-62 |  |  |
| *HLA-DOA* | HLA class II histocompatibility antigen, DO alpha chain | ENSG00000204252 | -1.8 | 1.28E-03 |  |  |
| *PRIM1* | DNA primase small subunit | ENSG00000198056 | -1.8 | 1.45E-61 |  |  |
| *CAVIN2* | Caveolae-associated protein 2 | ENSG00000168497 | -1.8 | 5.65E-36 |  |  |
| *MPP1* | 55 kDa erythrocyte membrane protein | ENSG00000130830 | -1.79 | 3.18E-37 |  |  |
| *E2F2* | Transcription factor E2F2 | ENSG00000007968 | -1.79 | 2.36E-20 |  |  |
| *RAD54B* | DNA repair and recombination protein RAD54B | ENSG00000197275 | -1.79 | 5.38E-27 |  |  |
| *BCL11B* | B-cell lymphoma/leukemia 11B | ENSG00000127152 | -1.79 | 6.30E-28 |  |  |
| *REEP2* | Receptor expression-enhancing protein 2 | ENSG00000132563 | -1.79 | 0.02 |  |  |
| *SCN4B* | Sodium channel subunit beta-4 | ENSG00000177098 | -1.78 | 3.09E-04 |  |  |
| *RDH5* | Retinol dehydrogenase 5 | ENSG00000135437 | -1.78 | 3.88E-03 |  |  |
| *MMP12* | Macrophage metalloelastase | ENSG00000262406 | -1.78 | 2.98E-06 |  |  |
| *FSTL5* | Follistatin-related protein 5 | ENSG00000168843 | -1.78 | 0.04 |  |  |
| *NNMT* | Nicotinamide N-methyltransferase | ENSG00000166741 | -1.78 | 7.06E-06 |  |  |
| *GDPD3* | Lysophospholipase D GDPD3 | ENSG00000102886 | -1.77 | 3.00E-07 |  |  |
| *SPARC* | SPARC | ENSG00000113140 | -1.77 | 9.97E-26 |  |  |
| *DHFR* | Dihydrofolate reductase | ENSG00000228716 | -1.77 | 2.01E-66 |  |  |
| *PLIN4* | Perilipin-4 | ENSG00000167676 | -1.77 | 3.71E-19 |  |  |
| *KIF2C* | Kinesin-like protein KIF2C | ENSG00000142945 | -1.77 | 2.68E-35 |  |  |
| *HSD3B7* | 3 beta-hydroxysteroid dehydrogenase type 7 | ENSG00000099377 | -1.76 | 3.02E-37 |  |  |
| *CSPG4* | Chondroitin sulfate proteoglycan 4 | ENSG00000173546 | -1.75 | 8.58E-09 |  |  |
| *CYTH4* | Cytohesin-4 | ENSG00000100055 | -1.75 | 7.57E-03 |  |  |
| *CDC7* | Cell division cycle 7-related protein kinase | ENSG00000097046 | -1.75 | 1.43E-54 |  |  |
| *CPA5* | Carboxypeptidase A5 | ENSG00000158525 | -1.74 | 0.04 |  |  |
| *VASH2* | Tubulinyl-Tyr carboxypeptidase 2 | ENSG00000143494 | -1.74 | 1.32E-03 |  |  |
| *S1PR5* | Sphingosine 1-phosphate receptor 5 | ENSG00000180739 | -1.74 | 2.51E-23 |  |  |
| *WDR62* | WD repeat-containing protein 62 | ENSG00000075702 | -1.74 | 2.77E-29 |  |  |
| *GRIP2* | Glutamate receptor-interacting protein 2 | ENSG00000144596 | -1.74 | 0.01 |  |  |
| *TICAM2* | TIR domain-containing adapter molecule 2 | ENSG00000243414 | -1.73 | 4.53E-06 |  |  |
| *PTN* | Pleiotrophin | ENSG00000105894 | -1.73 | 3.53E-21 |  |  |
| *COL4A2* | Collagen alpha-2(IV) chain | ENSG00000134871 | -1.73 | 1.05E-64 |  |  |
| *DEPDC1* | DEP domain-containing protein 1A | ENSG00000024526 | -1.73 | 1.63E-62 |  |  |
| *HASPIN* | Serine/threonine-protein kinase haspin | ENSG00000177602 | -1.73 | 6.16E-39 |  |  |
| *HEG1* | Protein HEG homolog 1 | ENSG00000173706 | -1.73 | 1.50E-63 |  |  |
| *PRIMA1* | Proline-rich membrane anchor 1 | ENSG00000175785 | -1.73 | 4.65E-03 |  |  |
| *ZNF695* | Zinc finger protein 695 | ENSG00000197472 | -1.73 | 1.90E-08 |  |  |
| *FST* | Follistatin | ENSG00000134363 | -1.73 | 1.50E-32 |  |  |
| *C10orf105* | Uncharacterized protein C10orf105 | ENSG00000214688 | -1.72 | 2.32E-04 |  |  |
| *ACACB* | Acetyl-CoA carboxylase 2 | ENSG00000076555 | -1.72 | 4.67E-21 |  |  |
| *GLT8D2* | Glycosyltransferase 8 domain-containing protein 2 | ENSG00000120820 | -1.72 | 2.09E-15 |  |  |
| *KPNA2* | Importin subunit alpha-1 | ENSG00000182481 | -1.72 | 1.33E-63 |  |  |
| *FBP1* | Fructose-1,6-bisphosphatase 1 | ENSG00000165140 | -1.72 | 5.09E-06 |  |  |
| *KIF18A* | Kinesin-like protein KIF18A | ENSG00000121621 | -1.71 | 3.45E-59 |  |  |
| *RTN4RL1* | Reticulon-4 receptor-like 1 | ENSG00000185924 | -1.71 | 3.49E-37 |  |  |
| *TMEM14A* | Transmembrane protein 14A | ENSG00000096092 | -1.71 | 4.97E-23 |  |  |
| *COL4A1* | Collagen alpha-1(IV) chain | ENSG00000187498 | -1.71 | 5.49E-136 |  |  |
| *ATP8B3* | Phospholipid-transporting ATPase IK | ENSG00000130270 | -1.7 | 6.70E-07 |  |  |
| *DUSP9* | Dual specificity protein phosphatase 9 | ENSG00000130829 | -1.7 | 2.32E-26 |  |  |
| *EME1* | Crossover junction endonuclease EME1 | ENSG00000154920 | -1.7 | 3.20E-23 |  |  |
| *ADAD2* | Adenosine deaminase domain-containing protein 2 | ENSG00000140955 | -1.7 | 0.04 |  |  |
| *MDH1B* | Putative malate dehydrogenase 1B | ENSG00000138400 | -1.7 | 0.02 |  |  |
| *SGO1* | Shugoshin 1 | ENSG00000129810 | -1.69 | 1.80E-20 |  |  |
| *CRIP1* | Cysteine-rich protein 1 | ENSG00000213145 | -1.69 | 8.12E-08 |  |  |
| *SLC22A17* | Solute carrier family 22 member 17 | ENSG00000092096 | -1.69 | 0.01 |  |  |
| *CFAP57* | Cilia And Flagella Associated Protein 57 | ENSG00000243710 | -1.68 | 2.79E-06 |  |  |
| *ESR2* | Estrogen receptor beta | ENSG00000140009 | -1.68 | 4.34E-05 |  |  |
| *LYPD5* | Ly6/PLAUR domain-containing protein 5 | ENSG00000159871 | -1.68 | 7.74E-16 |  |  |
| *KCNJ15* | ATP-sensitive inward rectifier potassium channel 15 | ENSG00000157551 | -1.67 | 1.69E-56 |  |  |
| *RACGAP1* | Rac GTPase-activating protein 1 | ENSG00000161800 | -1.67 | 2.81E-59 |  |  |
| *TROAP* | Tastin | ENSG00000135451 | -1.67 | 2.88E-28 |  |  |
| *PCNA* | Proliferating cell nuclear antigen | ENSG00000132646 | -1.67 | 3.44E-59 |  |  |
| *CKS2* | Cyclin-dependent kinases regulatory subunit 2 | ENSG00000123975 | -1.67 | 5.73E-58 |  |  |
| *NMU* | Neuromedin-U | ENSG00000109255 | -1.66 | 1.85E-06 |  |  |
| *CBX5* | Chromobox protein homolog 5 | ENSG00000094916 | -1.66 | 9.13E-120 |  |  |
| *C1orf21* | Uncharacterized protein C1orf21 | ENSG00000116667 | -1.65 | 9.32E-48 |  |  |
| *DSCC1* | Sister chromatid cohesion protein DCC1 | ENSG00000136982 | -1.65 | 7.33E-29 |  |  |
| *VIM* | Vimentin | ENSG00000026025 | -1.65 | 3.71E-03 |  |  |
| *RAB26* | Ras-related protein Rab-26 | ENSG00000167964 | -1.65 | 8.65E-32 |  |  |
| *RPA3* | Replication protein A 14 kDa subunit | ENSG00000106399 | -1.65 | 2.63E-116 |  |  |
| *USP51* | Ubiquitin carboxyl-terminal hydrolase 51 | ENSG00000247746 | -1.65 | 0.02 |  |  |
| *CDT1* | DNA replication factor Cdt1 | ENSG00000167513 | -1.65 | 9.48E-55 |  |  |
| *ENC1* | Ectoderm-neural cortex protein 1 | ENSG00000171617 | -1.65 | 9.92E-55 |  |  |
| *ARHGAP11A* | Rho GTPase-activating protein 11A | ENSG00000198826 | -1.65 | 5.75E-57 |  |  |
| *CEACAM6* | Carcinoembryonic antigen-related cell adhesion molecule 6 | ENSG00000086548 | -1.65 | 3.11E-03 |  |  |
| *TUBA1B* | Tubulin alpha-1B chain | ENSG00000123416 | -1.64 | 1.45E-41 |  |  |
| *LY6D* | Lymphocyte antigen 6D | ENSG00000167656 | -1.64 | 1.16E-24 |  |  |
| *CLIC3* | Chloride intracellular channel protein 3 | ENSG00000169583 | -1.64 | 1.75E-15 |  |  |
| *DIAPH3* | Protein diaphanous homolog 3 | ENSG00000139734 | -1.64 | 1.41E-52 |  |  |
| *MTFR2* | Mitochondrial fission regulator 2 | ENSG00000146410 | -1.64 | 2.78E-31 |  |  |
| *BNIPL* | Bcl-2/adenovirus E1B 19 kDa-interacting protein 2-like protein | ENSG00000163141 | -1.64 | 2.21E-03 |  |  |
| *RAD51AP1* | RAD51-associated protein 1 | ENSG00000111247 | -1.63 | 8.62E-33 |  |  |
| *IL11RA* | Interleukin-11 receptor subunit alpha | ENSG00000137070 | -1.63 | 2.88E-05 |  |  |
| *KCNH3* | Potassium voltage-gated channel subfamily H member 3 | ENSG00000135519 | -1.63 | 1.58E-04 |  |  |
| *SLC7A8* | Large neutral amino acids transporter small subunit 2 | ENSG00000092068 | -1.63 | 7.87E-24 |  |  |
| *FGFR3* | Fibroblast growth factor receptor 3 | ENSG00000068078 | -1.63 | 6.26E-34 |  |  |
| *GCNT2* | N-acetyllactosaminide beta-1,6-N-acetylglucosaminyl-transferase | ENSG00000111846 | -1.62 | 1.26E-05 |  |  |
| *COL1A2* | Collagen alpha-2(I) chain | ENSG00000164692 | -1.62 | 7.26E-11 |  |  |
| *FEZF1* | Fez family zinc finger protein 1 | ENSG00000128610 | -1.62 | 8.30E-04 |  |  |
| *EDAR* | Tumor necrosis factor receptor superfamily member EDAR | ENSG00000135960 | -1.62 | 4.63E-03 |  |  |
| *FANCB* | Fanconi anemia group B protein | ENSG00000181544 | -1.62 | 1.30E-14 |  |  |
| *C20orf204* | Chromosome 20 Open Reading Frame 204 | ENSG00000196421 | -1.62 | 0.03 |  |  |
| *CNFN* | Cornifelin | ENSG00000105427 | -1.62 | 3.77E-05 |  |  |
| *CDCA5* | Sororin | ENSG00000146670 | -1.61 | 2.91E-30 |  |  |
| *CDCA7* | Cell division cycle-associated protein 7 | ENSG00000144354 | -1.61 | 3.73E-53 |  |  |
| *CCDC65* | Dynein regulatory complex subunit 2 | ENSG00000139537 | -1.61 | 8.03E-03 |  |  |
| *SLC9A9* | Sodium/hydrogen exchanger 9 | ENSG00000181804 | -1.6 | 8.54E-06 |  |  |
| *FANCD2* | Fanconi anemia group D2 protein | ENSG00000144554 | -1.6 | 1.09E-40 |  |  |
| *POLQ* | DNA polymerase theta | ENSG00000051341 | -1.6 | 5.64E-31 |  |  |
| *SYT16* | Synaptotagmin-16 | ENSG00000139973 | -1.6 | 1.14E-06 |  |  |
| *C16orf54* | Transmembrane protein C16orf54 | ENSG00000185905 | -1.59 | 0.05 |  |  |
| *NUAK2* | NUAK family SNF1-like kinase 2 | ENSG00000163545 | -1.59 | 3.55E-49 |  |  |
| *E2F1* | Transcription factor E2F1 | ENSG00000101412 | -1.59 | 4.26E-49 |  |  |
| *KIF20B* | Kinesin-like protein KIF20B | ENSG00000138182 | -1.59 | 4.91E-53 |  |  |
| *GNG11* | Guanine nucleotide-binding protein G(I)/G(S)/G(O) subunit gamma-11 | ENSG00000127920 | -1.59 | 7.20E-11 |  |  |
| *NDC80* | Kinetochore protein NDC80 homolog | ENSG00000080986 | -1.59 | 9.47E-38 |  |  |
| *FLVCR2* | Feline leukemia virus subgroup C receptor-related protein 2 | ENSG00000119686 | -1.58 | 3.53E-05 |  |  |
| *MELK* | Maternal embryonic leucine zipper kinase | ENSG00000165304 | -1.58 | 3.58E-52 |  |  |
| *KNTC1* | Kinetochore-associated protein 1 | ENSG00000184445 | -1.58 | 1.50E-114 |  |  |
| *SAP25* | Histone deacetylase complex subunit SAP25 | ENSG00000205307 | -1.58 | 1.02E-03 |  |  |
| *SKA3* | Spindle and kinetochore-associated protein 3 | ENSG00000165480 | -1.58 | 2.11E-30 |  |  |
| *TIAM2* | Rho guanine nucleotide exchange factor TIAM2 | ENSG00000146426 | -1.58 | 9.46E-04 |  |  |
| *PDE1C* | Calcium/calmodulin-dependent 3',5'-cyclic nucleotide phosphodiesterase 1C | ENSG00000154678 | -1.58 | 0.02 |  |  |
| *PRR36* | Proline Rich 36 | ENSG00000183248 | -1.58 | 0.01 |  |  |
| *FCGR2A* | Low affinity immunoglobulin gamma Fc region receptor II-a | ENSG00000143226 | -1.58 | 0.02 |  |  |
| *ATAD2* | ATPase family AAA domain-containing protein 2 | ENSG00000156802 | -1.58 | 7.96E-53 |  |  |
| *NID1* | Nidogen-1 | ENSG00000116962 | -1.57 | 2.51E-03 |  |  |
| *TEDC2* | Tubulin epsilon and delta complex protein 2 | ENSG00000162062 | -1.57 | 2.03E-19 |  |  |
| *KLRC2* | NKG2-C type II integral membrane protein | ENSG00000205809 | -1.57 | 1.28E-03 |  |  |
| *CIITA* | MHC class II transactivator | ENSG00000179583 | -1.57 | 1.82E-47 |  |  |
| *CCNI2* | Cyclin-I2 | ENSG00000205089 | -1.57 | 6.66E-03 |  |  |
| *CENPH* | Centromere protein H | ENSG00000153044 | -1.57 | 3.88E-48 |  |  |
| *APLN* | Apelin | ENSG00000171388 | -1.57 | 6.86E-04 |  |  |
| *NFAM1* | NFAT activation molecule 1 | ENSG00000235568 | -1.57 | 3.27E-11 |  |  |
| *KCNJ10* | ATP-sensitive inward rectifier potassium channel 10 | ENSG00000177807 | -1.57 | 0.03 |  |  |
| *AOC3* | Membrane primary amine oxidase | ENSG00000131471 | -1.57 | 0.02 |  |  |
| *PLK4* | Serine/threonine-protein kinase PLK4 | ENSG00000142731 | -1.57 | 3.62E-22 |  |  |
| *EEPD1* | Endonuclease/exonuclease/phosphatase family domain-containing protein 1 | ENSG00000122547 | -1.57 | 5.99E-14 |  |  |
| *ZNF519* | Zinc finger protein 519 | ENSG00000175322 | -1.56 | 3.81E-17 |  |  |
| *TFCP2L1* | Transcription factor CP2-like protein 1 | ENSG00000115112 | -1.56 | 2.01E-47 |  |  |
| *MCM5* | DNA replication licensing factor MCM5 | ENSG00000100297 | -1.56 | 3.77E-52 |  |  |
| *SEPTIN4* | Septin-4 | ENSG00000108387 | -1.56 | 0.04 |  |  |
| *KIF23* | Kinesin-like protein KIF23 | ENSG00000137807 | -1.56 | 1.63E-34 |  |  |
| *TONSL* | Tonsoku-like protein | ENSG00000160949 | -1.56 | 2.02E-46 |  |  |
| *SMC4* | Structural maintenance of chromosomes protein 4 | ENSG00000113810 | -1.56 | 1.55E-52 |  |  |
| *BARD1* | BRCA1-associated RING domain protein 1 | ENSG00000138376 | -1.56 | 1.10E-46 |  |  |
| *TNF* | Tumor necrosis factor | ENSG00000232810 | -1.55 | 5.10E-46 |  |  |
| *APOBR* | Apolipoprotein B receptor | ENSG00000184730 | -1.55 | 2.85E-07 |  |  |
| *UPK2* | Uroplakin-2 | ENSG00000110375 | -1.55 | 0.01 |  |  |
| *FANCG* | Fanconi anemia group G protein | ENSG00000221829 | -1.55 | 1.49E-48 |  |  |
| *APOBEC3C* | DNA dC->dU-editing enzyme APOBEC-3C | ENSG00000244509 | -1.55 | 2.01E-110 |  |  |
| *PARPBP* | PCNA-interacting partner | ENSG00000185480 | -1.55 | 1.45E-43 |  |  |
| *CENPW* | Centromere protein W | ENSG00000203760 | -1.55 | 1.96E-22 |  |  |
| *SYT15* | Synaptotagmin-15 | ENSG00000204176 | -1.55 | 3.31E-06 |  |  |
| *SLC1A3* | Excitatory amino acid transporter 1 | ENSG00000079215 | -1.55 | 4.45E-47 |  |  |
| *FANCI* | Fanconi anemia group I protein | ENSG00000140525 | -1.55 | 1.03E-50 |  |  |
| *PLA2G7* | Platelet-activating factor acetylhydrolase | ENSG00000146070 | -1.55 | 5.87E-04 |  |  |
| *BMP4* | Bone morphogenetic protein 4 | ENSG00000125378 | -1.55 | 3.06E-06 |  |  |
| *CDH2* | Cadherin-2 | ENSG00000170558 | -1.54 | 1.96E-23 |  |  |
| *HROB* | Homologous recombination OB-fold protein | ENSG00000125319 | -1.54 | 1.49E-20 |  |  |
| *CLDN9* | Claudin-9 | ENSG00000213937 | -1.54 | 2.62E-05 |  |  |
| *RGS9BP* | Regulator of G-protein signaling 9-binding protein | ENSG00000186326 | -1.54 | 5.03E-03 |  |  |
| *TRIP13* | Pachytene checkpoint protein 2 homolog | ENSG00000071539 | -1.54 | 7.75E-48 |  |  |
| *FZD4* | Frizzled-4 | ENSG00000174804 | -1.54 | 3.16E-41 |  |  |
| *CACNG4* | Voltage-dependent calcium channel gamma-4 subunit | ENSG00000075461 | -1.54 | 2.34E-11 |  |  |
| *MNS1* | Meiosis-specific nuclear structural protein 1 | ENSG00000138587 | -1.54 | 1.72E-11 |  |  |
| *TMEM256* | Transmembrane protein 256 | ENSG00000205544 | -1.54 | 1.67E-44 |  |  |
| *CXCL10* | C-X-C motif chemokine 10 | ENSG00000169245 | -1.53 | 1.35E-10 |  |  |
| *BRIP1* | Fanconi anemia group J protein | ENSG00000136492 | -1.53 | 3.13E-30 |  |  |
| *KHK* | Ketohexokinase | ENSG00000138030 | -1.53 | 4.26E-06 |  |  |
| *ARHGAP19* | Rho GTPase-activating protein 19 | ENSG00000213390 | -1.53 | 5.74E-46 |  |  |
| *ATAD5* | ATPase family AAA domain-containing protein 5 | ENSG00000176208 | -1.53 | 4.49E-44 |  |  |
| *MFNG* | Beta-1,3-N-acetylglucosaminyltransferase manic fringe | ENSG00000100060 | -1.53 | 1.81E-06 |  |  |
| *ARHGAP11A-SCG5* | ARHGAP11A-SCG5 readthrough | ENSG00000288864 | -1.53 | 2.17E-05 |  |  |
| *CHAF1B* | Chromatin assembly factor 1 subunit B | ENSG00000159259 | -1.53 | 6.83E-46 |  |  |
| *CNTNAP1* | Contactin-associated protein 1 | ENSG00000108797 | -1.53 | 5.88E-16 |  |  |
| *EPHB6* | Ephrin type-B receptor 6 | ENSG00000106123 | -1.52 | 9.49E-10 |  |  |
| *AASS* | Alpha-aminoadipic semialdehyde synthase, mitochondrial | ENSG00000008311 | -1.52 | 3.13E-46 |  |  |
| *LRRC7* | Leucine-rich repeat-containing protein 7 | ENSG00000033122 | -1.52 | 1.76E-10 |  |  |
| *DBF4B* | Protein DBF4 homolog B | ENSG00000161692 | -1.52 | 7.80E-23 |  |  |
| *PHF19* | PHD finger protein 19 | ENSG00000119403 | -1.52 | 5.23E-46 |  |  |
| *HLA-DQB2* | HLA class II histocompatibility antigen, DQ beta 2 chain | ENSG00000232629 | -1.52 | 6.97E-03 |  |  |
| *BLM* | Bloom syndrome protein | ENSG00000197299 | -1.52 | 3.57E-43 |  |  |
| *FAM156B* | Family With Sequence Similarity 156 Member B | ENSG00000179304 | -1.52 | 5.03E-03 |  |  |
| *DMRT2* | Doublesex- and mab-3-related transcription factor 2 | ENSG00000173253 | -1.51 | 9.26E-06 |  |  |
| *GPR137C* | Integral membrane protein GPR137C | ENSG00000180998 | -1.51 | 1.19E-04 |  |  |
| *DZIP1L* | Zinc finger protein DZIP1L | ENSG00000158163 | -1.51 | 2.75E-07 |  |  |
| *DEPDC7* | DEP domain-containing protein 7 | ENSG00000121690 | -1.51 | 2.70E-17 |  |  |
| *GNAO1* | Guanine nucleotide-binding protein G(o) subunit alpha | ENSG00000087258 | -1.51 | 2.79E-14 |  |  |
| *KLRC3* | NKG2-E type II integral membrane protein | ENSG00000205810 | -1.51 | 0.01 |  |  |
| *SMC2* | Structural maintenance of chromosomes protein 2 | ENSG00000136824 | -1.51 | 1.56E-48 |  |  |
| *PKD1L2* | Polycystic kidney disease protein 1-like 2 | ENSG00000166473 | -1.5 | 3.67E-03 |  |  |
| *PALMD* | Palmdelphin | ENSG00000099260 | -1.5 | 3.59E-15 |  |  |
| *CYB5RL* | NADH-cytochrome b5 reductase-like | ENSG00000215883 | -1.5 | 1.73E-19 |  |  |
| *ADAMTS3* | A disintegrin and metalloproteinase with thrombospondin motifs 3 | ENSG00000156140 | -1.5 | 4.68E-05 |  |  |
| *CASP14* | Caspase-14 | ENSG00000105141 | -1.5 | 0.02 |  |  |
| *EDN1* | Endothelin-1 | ENSG00000078401 | -1.5 | 1.76E-20 |  |  |
| *SH2D3C* | SH2 domain-containing protein 3C | ENSG00000095370 | -1.5 | 2.88E-15 |  |  |
| *GALNT9* | Polypeptide N-acetylgalactosaminyltransferase 9 | ENSG00000182870 | -1.5 | 0.02 |  |  |
| *TMEM139* | Transmembrane protein 139 | ENSG00000178826 | -1.5 | 0.02 |  |  |
| *IFI30* | Gamma-interferon-inducible lysosomal thiol reductase | ENSG00000216490 | -1.49 | 7.72E-47 |  |  |
| *IQGAP3* | Ras GTPase-activating-like protein IQGAP3 | ENSG00000183856 | -1.49 | 1.81E-17 |  |  |
| *NALCN* | Sodium leak channel non-selective protein | ENSG00000102452 | -1.49 | 7.62E-15 |  |  |
| *C4orf46* | Renal cancer differentiation gene 1 protein | ENSG00000205208 | -1.49 | 4.76E-20 |  |  |
| *RNASEH2A* | Ribonuclease H2 subunit A | ENSG00000104889 | -1.48 | 1.38E-44 |  |  |
| *PFKFB4* | 6-phosphofructo-2-kinase/fructose-2,6-bisphosphatase 4 | ENSG00000114268 | -1.48 | 4.12E-08 |  |  |
| *SUV39H1* | Histone-lysine N-methyltransferase SUV39H1 | ENSG00000101945 | -1.47 | 2.74E-40 |  |  |
| *GPR61* | G-protein coupled receptor 61 | ENSG00000156097 | -1.46 | 0.03 |  |  |
| *SOWAHD* | Sosondowah Ankyrin Repeat Domain Family Member D | ENSG00000187808 | -1.46 | 0.01 |  |  |
| *KIF24* | Kinesin-like protein KIF24 | ENSG00000186638 | -1.46 | 3.46E-19 |  |  |
| *LYPD1* | Ly6/PLAUR domain-containing protein 1 | ENSG00000150551 | -1.46 | 5.43E-38 |  |  |
| *PDPN* | Podoplanin | ENSG00000162493 | -1.46 | 8.64E-43 |  |  |
| *GAS1* | Growth arrest-specific protein 1 | ENSG00000180447 | -1.46 | 3.27E-05 |  |  |
| *ARL4C* | ADP-ribosylation factor-like protein 4C | ENSG00000188042 | -1.46 | 7.52E-100 |  |  |
| *PPP2R2B* | Serine/threonine-protein phosphatase 2A 55 kDa regulatory subunit B beta isoform | ENSG00000156475 | -1.45 | 1.72E-40 |  |  |
| *SMTN* | Smoothelin | ENSG00000183963 | -1.45 | 6.16E-38 |  |  |
| *KLB* | Beta-klotho | ENSG00000134962 | -1.45 | 0.02 |  |  |
| *RARB* | Retinoic acid receptor beta | ENSG00000077092 | -1.45 | 5.79E-07 |  |  |
| *SLX1B* | *SLX1 Homolog B* | ENSG00000181625 | -1.45 | 2.65E-06 |  |  |
| *ST3GAL6* | Type 2 lactosamine alpha-2,3-sialyltransferase | ENSG00000064225 | -1.44 | 1.69E-05 |  |  |
| *C5orf34* | Chromosome 5 Open Reading Frame 34 | ENSG00000172244 | -1.44 | 7.47E-11 |  |  |
| *MATN2* | Matrilin-2 | ENSG00000132561 | -1.44 | 1.13E-29 |  |  |
| *ECT2* | Protein ECT2 | ENSG00000114346 | -1.44 | 8.21E-45 |  |  |
| *PHLDB1* | Pleckstrin homology-like domain family B member 1 | ENSG00000019144 | -1.44 | 1.74E-39 |  |  |
| *CORO6* | Coronin-6 | ENSG00000167549 | -1.44 | 5.30E-19 |  |  |
| *PTGS1* | Prostaglandin G/H synthase 1 | ENSG00000095303 | -1.44 | 5.40E-34 |  |  |
| *KIF22* | Kinesin-like protein KIF22 | ENSG00000079616 | -1.44 | 1.15E-43 |  |  |
| *PARP1* | Poly [ADP-ribose] polymerase 1 | ENSG00000143799 | -1.44 | 2.50E-100 |  |  |
| *DNAJC22* | DnaJ homolog subfamily C member 22 | ENSG00000178401 | -1.44 | 1.54E-04 |  |  |
| *LGALS9C* | Galectin-9C | ENSG00000171916 | -1.43 | 0.02 |  |  |
| *ZNF483* | Zinc finger protein 483 | ENSG00000173258 | -1.43 | 1.19E-20 |  |  |
| *HAUS4* | HAUS augmin-like complex subunit 4 | ENSG00000092036 | -1.43 | 7.41E-42 |  |  |
| *ALDH7A1* | Alpha-aminoadipic semialdehyde dehydrogenase | ENSG00000164904 | -1.43 | 3.22E-44 |  |  |
| *MCM6* | DNA replication licensing factor MCM6 | ENSG00000076003 | -1.43 | 1.26E-43 |  |  |
| *FILIP1L* | Filamin A-interacting protein 1-like | ENSG00000168386 | -1.43 | 1.72E-41 |  |  |
| *CENPN* | Centromere protein N | ENSG00000166451 | -1.43 | 3.27E-42 |  |  |
| *ATP5MC1* | ATP synthase F(0) complex subunit C1, mitochondrial | ENSG00000159199 | -1.43 | 6.60E-43 |  |  |
| *KLK1* | Kallikrein-1 | ENSG00000167748 | -1.43 | 4.34E-03 |  |  |
| *CCL5* | C-C motif chemokine 5 | ENSG00000271503 | -1.43 | 3.03E-04 |  |  |
| *CREG2* | Protein CREG2 | ENSG00000175874 | -1.43 | 5.72E-05 |  |  |
| *C1QL1* | C1q-related factor | ENSG00000131094 | -1.43 | 1.35E-05 |  |  |
| *MPP2* | MAGUK p55 subfamily member 2 | ENSG00000108852 | -1.43 | 1.79E-20 |  |  |
| *CAP2* | Adenylyl cyclase-associated protein 2 | ENSG00000112186 | -1.42 | 3.56E-41 |  |  |
| *HMGN3* | High mobility group nucleosome-binding domain-containing protein 3 | ENSG00000118418 | -1.42 | 2.85E-41 |  |  |
| *CCDC150* | Coiled-coil domain-containing protein 150 | ENSG00000144395 | -1.42 | 3.26E-07 |  |  |
| *KCNIP2* | Kv channel-interacting protein 2 | ENSG00000120049 | -1.42 | 8.51E-04 |  |  |
| *MMS22L* | Protein MMS22-like | ENSG00000146263 | -1.42 | 2.51E-40 |  |  |
| *STIL* | SCL-interrupting locus protein | ENSG00000123473 | -1.42 | 1.31E-41 |  |  |
| *MICB* | MHC class I polypeptide-related sequence B | ENSG00000204516 | -1.42 | 6.92E-84 |  |  |
| *FBXO5* | F-box only protein 5 | ENSG00000112029 | -1.42 | 1.14E-25 |  |  |
| *FOXN1* | Forkhead box protein N1 | ENSG00000109101 | -1.41 | 5.91E-03 |  |  |
| *EDIL3* | EGF-like repeat and discoidin I-like domain-containing protein 3 | ENSG00000164176 | -1.41 | 1.52E-21 |  |  |
| *NUDT7* | Peroxisomal coenzyme A diphosphatase NUDT7 | ENSG00000140876 | -1.41 | 1.10E-03 |  |  |
| *APOBEC3B* | DNA dC->dU-editing enzyme APOBEC-3B | ENSG00000179750 | -1.41 | 5.66E-21 |  |  |
| *TMCC2* | Transmembrane and coiled-coil domains protein 2 | ENSG00000133069 | -1.41 | 1.71E-13 |  |  |
| *DCST1* | E3 ubiquitin-protein ligase DCST1 | ENSG00000163357 | -1.41 | 6.88E-03 |  |  |
| *AKAP5* | A-kinase anchor protein 5 | ENSG00000179841 | -1.41 | 1.51E-03 |  |  |
| *CKAP2* | Cytoskeleton-associated protein 2 | ENSG00000136108 | -1.41 | 1.54E-42 |  |  |
| *MYO3B* | Myosin-IIIb | ENSG00000071909 | -1.41 | 1.25E-05 |  |  |
| *SMIM38* | Small integral membrane protein 38 | ENSG00000284713 | -1.41 | 7.65E-03 |  |  |
| *MCM2* | DNA replication licensing factor MCM2 | ENSG00000073111 | -1.41 | 1.22E-42 |  |  |
| *PABPC4L* | Polyadenylate-binding protein 4-like | ENSG00000254535 | -1.41 | 4.26E-06 |  |  |
| *DTL* | Denticleless protein homolog | ENSG00000143476 | -1.4 | 5.59E-41 |  |  |
| *DKK3* | Dickkopf-related protein 3 | ENSG00000050165 | -1.4 | 5.16E-42 |  |  |
| *GMNN* | Geminin | ENSG00000112312 | -1.4 | 6.71E-39 |  |  |
| *CIDEB* | Cell death activator CIDE-B | ENSG00000136305 | -1.4 | 5.05E-05 |  |  |
| *FANCA* | Fanconi anemia group A protein | ENSG00000187741 | -1.4 | 8.43E-39 |  |  |
| *CCN5* | CCN family member 5 | ENSG00000064205 | -1.4 | 4.59E-04 |  |  |
| *CHTF18* | Chromosome transmission fidelity protein 18 homolog | ENSG00000127586 | -1.4 | 5.26E-39 |  |  |
| *PXMP4* | Peroxisomal membrane protein 4 | ENSG00000101417 | -1.39 | 4.11E-37 |  |  |
| *LRRCC1* | Leucine-rich repeat and coiled-coil domain-containing protein 1 | ENSG00000133739 | -1.39 | 3.41E-35 |  |  |
| *TSPAN4* | Tetraspanin-4 | ENSG00000214063 | -1.39 | 4.63E-37 |  |  |
| *S100A2* | Protein S100-A2 | ENSG00000196754 | -1.38 | 6.74E-42 |  |  |
| *CCK* | Cholecystokinin | ENSG00000187094 | -1.38 | 7.83E-03 |  |  |
| *PASK* | PAS domain-containing serine/threonine-protein kinase | ENSG00000115687 | -1.38 | 2.40E-36 |  |  |
| *POLE* | DNA polymerase epsilon catalytic subunit A | ENSG00000177084 | -1.38 | 3.03E-87 |  |  |
| *PKD1L1* | Polycystic kidney disease protein 1-like 1 | ENSG00000158683 | -1.38 | 0.01 |  |  |
| *PIPOX* | Peroxisomal sarcosine oxidase | ENSG00000179761 | -1.37 | 0.02 |  |  |
| *HAUS8* | HAUS augmin-like complex subunit 8 | ENSG00000131351 | -1.37 | 1.77E-18 |  |  |
| *CCNE2* | G1/S-specific cyclin-E2 | ENSG00000175305 | -1.37 | 7.67E-11 |  |  |
| *MSLN* | Mesothelin | ENSG00000102854 | -1.37 | 6.84E-03 |  |  |
| *CIP2A* | Protein CIP2A | ENSG00000163507 | -1.37 | 4.62E-39 |  |  |
| *SCIN* | Adseverin | ENSG00000006747 | -1.37 | 1.67E-03 |  |  |
| *SAPCD2* | Suppressor APC domain-containing protein 2 | ENSG00000186193 | -1.36 | 1.30E-38 |  |  |
| *ROM1* | Rod outer segment membrane protein 1 | ENSG00000149489 | -1.36 | 1.67E-04 |  |  |
| *CENPU* | Centromere protein U | ENSG00000151725 | -1.36 | 2.38E-26 |  |  |
| *ELAVL2* | ELAV-like protein 2 | ENSG00000107105 | -1.36 | 1.37E-22 |  |  |
| *MCM7* | DNA replication licensing factor MCM7 | ENSG00000166508 | -1.36 | 2.48E-40 |  |  |
| *SNRNP25* | U11/U12 small nuclear ribonucleoprotein 25 kDa protein | ENSG00000161981 | -1.36 | 9.26E-29 |  |  |
| *CSPG5* | Chondroitin sulfate proteoglycan 5 | ENSG00000114646 | -1.36 | 7.30E-06 |  |  |
| *ATOX1* | Copper transport protein ATOX1 | ENSG00000177556 | -1.36 | 8.86E-38 |  |  |
| *FBN1* | Fibrillin-1 | ENSG00000166147 | -1.36 | 8.83E-05 |  |  |
| *DNMT1* | DNA (cytosine-5)-methyltransferase 1 | ENSG00000130816 | -1.36 | 1.95E-88 |  |  |
| *LRRC20* | Leucine-rich repeat-containing protein 20 | ENSG00000172731 | -1.35 | 4.66E-19 |  |  |
| *CBX1* | Chromobox protein homolog 1 | ENSG00000108468 | -1.35 | 5.19E-82 |  |  |
| *PCDH7* | Protocadherin-7 | ENSG00000169851 | -1.35 | 2.83E-38 |  |  |
| *CENPO* | Centromere protein O | ENSG00000138092 | -1.35 | 7.17E-36 |  |  |
| *ACYP1* | Acylphosphatase-1 | ENSG00000119640 | -1.35 | 4.97E-18 |  |  |
| *KBTBD3* | Kelch Repeat And BTB Domain Containing 3 | ENSG00000182359 | -1.35 | 6.30E-05 |  |  |
| *ALDH3A1* | Aldehyde dehydrogenase, dimeric NADP-preferring | ENSG00000108602 | -1.35 | 1.53E-34 |  |  |
| *ANKRD20A1* | Ankyrin repeat domain-containing protein 20A1 | ENSG00000260691 | -1.34 | 9.70E-06 |  |  |
| *GRAMD2A* | GRAM domain-containing protein 2A | ENSG00000175318 | -1.34 | 8.09E-34 |  |  |
| *RIBC2* | RIB43A-like with coiled-coils protein 2 | ENSG00000128408 | -1.34 | 8.37E-05 |  |  |
| *CENPS-CORT* | CENPS-CORT readthrough | ENSG00000251503 | -1.34 | 1.75E-03 |  |  |
| *KLHL13* | Kelch-like protein 13 | ENSG00000003096 | -1.34 | 6.37E-35 |  |  |
| *SLC2A4* | Solute carrier family 2, facilitated glucose transporter member 4 | ENSG00000181856 | -1.34 | 4.11E-04 |  |  |
| *CHEK2* | Serine/threonine-protein kinase Chk2 | ENSG00000183765 | -1.34 | 4.21E-35 |  |  |
| *MXD3* | Max dimerization protein 3 | ENSG00000213347 | -1.34 | 8.92E-17 |  |  |
| *ALDOC* | Fructose-bisphosphate aldolase C | ENSG00000109107 | -1.33 | 5.53E-17 |  |  |
| *EPN3* | Epsin-3 | ENSG00000049283 | -1.33 | 5.22E-36 |  |  |
| *PGR* | Progesterone receptor | ENSG00000082175 | -1.33 | 0.02 |  |  |
| *CDKN2D* | Cyclin-dependent kinase 4 inhibitor D | ENSG00000129355 | -1.33 | 3.48E-15 |  |  |
| *TIMELESS* | Protein timeless homolog | ENSG00000111602 | -1.33 | 1.21E-37 |  |  |
| *RAD51* | DNA repair protein RAD51 homolog 1 | ENSG00000051180 | -1.33 | 1.06E-24 |  |  |
| *SPTLC3* | Serine palmitoyltransferase 3 | ENSG00000172296 | -1.33 | 7.44E-16 |  |  |
| *CNN3* | Calponin-3 | ENSG00000117519 | -1.33 | 4.15E-81 |  |  |
| *EXOC3L1* | Exocyst complex component 3-like protein | ENSG00000179044 | -1.33 | 2.15E-03 |  |  |
| *PCOLCE* | Procollagen C-endopeptidase enhancer 1 | ENSG00000106333 | -1.33 | 5.69E-03 |  |  |
| *UPK3B* | Uroplakin-3b | ENSG00000243566 | -1.33 | 5.20E-16 |  |  |
| *RAB3B* | Ras-related protein Rab-3B | ENSG00000169213 | -1.33 | 3.02E-10 |  |  |
| *XRCC2* | DNA repair protein XRCC2 | ENSG00000196584 | -1.33 | 6.22E-34 |  |  |
| *PDZD7* | PDZ domain-containing protein 7 | ENSG00000186862 | -1.33 | 6.81E-04 |  |  |
| *MID1* | E3 ubiquitin-protein ligase Midline-1 | ENSG00000101871 | -1.32 | 4.11E-36 |  |  |
| *NGEF* | Ephexin-1 | ENSG00000066248 | -1.32 | 7.17E-07 |  |  |
| *TTF2* | Transcription termination factor 2 | ENSG00000116830 | -1.32 | 8.74E-81 |  |  |
| *CEP152* | Centrosomal protein of 152 kDa | ENSG00000103995 | -1.32 | 4.76E-35 |  |  |
| *SKA1* | Spindle and kinetochore-associated protein 1 | ENSG00000154839 | -1.32 | 4.97E-12 |  |  |
| *CCDC121* | Coiled-coil domain-containing protein 121 | ENSG00000176714 | -1.32 | 5.70E-06 |  |  |
| *AQP3* | Aquaporin-3 | ENSG00000165272 | -1.32 | 9.86E-12 |  |  |
| *RNFT2* | RING finger and transmembrane domain-containing protein 2 | ENSG00000135119 | -1.31 | 1.38E-04 |  |  |
| *ZNF367* | Zinc finger protein 367 | ENSG00000165244 | -1.31 | 1.45E-19 |  |  |
| *DSN1* | Kinetochore-associated protein DSN1 homolog | ENSG00000149636 | -1.31 | 7.35E-35 |  |  |
| *LCTL* | Lactase-like protein | ENSG00000188501 | -1.31 | 0.03 |  |  |
| *PRICKLE4* | Prickle-like protein 4 | ENSG00000278224 | -1.31 | 1.60E-07 |  |  |
| *CAT* | Catalase | ENSG00000121691 | -1.31 | 2.19E-37 |  |  |
| *ARHGEF26* | Rho guanine nucleotide exchange factor 26 | ENSG00000114790 | -1.31 | 6.08E-13 |  |  |
| *CNIH2* | Protein cornichon homolog 2 | ENSG00000174871 | -1.3 | 6.40E-03 |  |  |
| *H2AZ2* | Histone H2A.V | ENSG00000105968 | -1.3 | 3.46E-37 |  |  |
| *TEDC1* | Tubulin epsilon and delta complex protein 1 | ENSG00000185347 | -1.3 | 3.69E-15 |  |  |
| *DDN* | Dendrin | ENSG00000181418 | -1.3 | 1.19E-04 |  |  |
| *MAGEE1* | Melanoma-associated antigen E1 | ENSG00000198934 | -1.3 | 9.67E-08 |  |  |
| *DDB2* | DNA damage-binding protein 2 | ENSG00000134574 | -1.3 | 2.82E-32 |  |  |
| *IMPA2* | Inositol monophosphatase 2 | ENSG00000141401 | -1.3 | 3.16E-73 |  |  |
| *SNTB2* | Beta-2-syntrophin | ENSG00000168807 | -1.3 | 1.64E-76 |  |  |
| *BRI3BP* | BRI3-binding protein | ENSG00000184992 | -1.3 | 7.21E-36 |  |  |
| *CDK2* | Cyclin-dependent kinase 2 | ENSG00000123374 | -1.3 | 1.05E-75 |  |  |
| *FAM229B* | Protein FAM229B | ENSG00000203778 | -1.3 | 1.70E-04 |  |  |
| *TMPO* | Lamina-associated polypeptide 2, isoform alpha | ENSG00000120802 | -1.29 | 1.89E-36 |  |  |
| *TMEM37* | Voltage-dependent calcium channel gamma-like subunit | ENSG00000171227 | -1.29 | 3.01E-10 |  |  |
| *MCM3* | DNA replication licensing factor MCM3 | ENSG00000112118 | -1.29 | 9.37E-80 |  |  |
| *UGT1A6* | UDP-glucuronosyltransferase 1-6 | ENSG00000167165 | -1.29 | 1.93E-05 |  |  |
| *RMI2* | RecQ-mediated genome instability protein 2 | ENSG00000175643 | -1.29 | 8.54E-32 |  |  |
| *GPSM2* | G-protein-signaling modulator 2 | ENSG00000121957 | -1.29 | 1.75E-35 |  |  |
| *ITGA4* | Integrin alpha-4 | ENSG00000115232 | -1.29 | 1.32E-33 |  |  |
| *RNPEP* | Aminopeptidase B | ENSG00000176393 | -1.29 | 1.73E-77 |  |  |
| *GPATCH11* | G patch domain-containing protein 11 | ENSG00000152133 | -1.28 | 2.58E-32 |  |  |
| *BCAM* | Basal cell adhesion molecule | ENSG00000187244 | -1.28 | 2.73E-76 |  |  |
| *SCN1B* | Sodium channel subunit beta-1 | ENSG00000105711 | -1.28 | 4.82E-04 |  |  |
| *EMP2* | Epithelial membrane protein 2 | ENSG00000213853 | -1.28 | 3.00E-35 |  |  |
| *APAF1* | Apoptotic protease-activating factor 1 | ENSG00000120868 | -1.28 | 7.36E-33 |  |  |
| *TET1* | Methylcytosine dioxygenase TET1 | ENSG00000138336 | -1.28 | 6.94E-14 |  |  |
| *MCM4* | DNA replication licensing factor MCM4 | ENSG00000104738 | -1.28 | 1.30E-35 |  |  |
| *KCTD14* | BTB/POZ domain-containing protein KCTD14 | ENSG00000151364 | -1.28 | 2.33E-10 |  |  |
| *KRT15* | Keratin, type I cytoskeletal 15 | ENSG00000171346 | -1.27 | 8.80E-36 |  |  |
| *MYORG* | Myogenesis-regulating glycosidase | ENSG00000164976 | -1.27 | 2.02E-30 |  |  |
| *DDIAS* | DNA damage-induced apoptosis suppressor protein | ENSG00000165490 | -1.27 | 3.01E-32 |  |  |
| *HMCES* | Abasic site processing protein HMCES | ENSG00000183624 | -1.27 | 6.66E-34 |  |  |
| *CTNNAL1* | Alpha-catulin | ENSG00000119326 | -1.27 | 1.08E-34 |  |  |
| *PC* | Pyruvate carboxylase, mitochondrial | ENSG00000173599 | -1.27 | 5.04E-34 |  |  |
| *CGN* | Cingulin | ENSG00000143375 | -1.27 | 9.60E-32 |  |  |
| *EFNB3* | Ephrin-B3 | ENSG00000108947 | -1.26 | 0.02 |  |  |
| *CCHCR1* | Coiled-coil alpha-helical rod protein 1 | ENSG00000204536 | -1.26 | 1.66E-32 |  |  |
| *GASK1B* | Golgi-associated kinase 1B | ENSG00000164125 | -1.26 | 9.55E-30 |  |  |
| *LY6G5C* | Lymphocyte antigen 6 complex locus protein G5c | ENSG00000204428 | -1.26 | 0.03 |  |  |
| *SLC47A2* | Multidrug and toxin extrusion protein 2 | ENSG00000180638 | -1.26 | 7.72E-05 |  |  |
| *SKA2* | Spindle and kinetochore-associated protein 2 | ENSG00000182628 | -1.26 | 2.01E-33 |  |  |
| *MOB3B* | MOB kinase activator 3B | ENSG00000120162 | -1.26 | 4.24E-19 |  |  |
| *ORC6* | Origin recognition complex subunit 6 | ENSG00000091651 | -1.25 | 4.63E-31 |  |  |
| *CD27* | CD27 antigen | ENSG00000139193 | -1.25 | 0.03 |  |  |
| *RFC5* | Replication factor C subunit 5 | ENSG00000111445 | -1.25 | 5.66E-32 |  |  |
| *FMO4* | Dimethylaniline monooxygenase [N-oxide-forming] 4 | ENSG00000076258 | -1.25 | 3.48E-05 |  |  |
| *CHEK1* | Serine/threonine-protein kinase Chk1 | ENSG00000149554 | -1.25 | 3.36E-21 |  |  |
| *RFC2* | Replication factor C subunit 2 | ENSG00000049541 | -1.25 | 1.96E-32 |  |  |
| *IDH2* | Isocitrate dehydrogenase [NADP], mitochondrial | ENSG00000182054 | -1.25 | 3.83E-33 |  |  |
| *IRS1* | Insulin receptor substrate 1 | ENSG00000169047 | -1.25 | 7.70E-75 |  |  |
| *WDHD1* | WD repeat and HMG-box DNA-binding protein 1 | ENSG00000198554 | -1.25 | 8.03E-32 |  |  |
| *SLC25A40* | Solute carrier family 25 member 40 | ENSG00000075303 | -1.24 | 8.04E-65 |  |  |
| *KITLG* | Kit ligand | ENSG00000049130 | -1.24 | 8.03E-69 |  |  |
| *OASL* | 2'-5'-oligoadenylate synthase-like protein | ENSG00000135114 | -1.24 | 4.85E-13 |  |  |
| *FOXO6* | Forkhead box protein O6 | ENSG00000204060 | -1.24 | 1.33E-03 |  |  |
| *ANO2* | Anoctamin-2 | ENSG00000047617 | -1.24 | 0.05 |  |  |
| *CDH11* | Cadherin-11 | ENSG00000140937 | -1.24 | 0.01 |  |  |
| *CROT* | Peroxisomal carnitine O-octanoyltransferase | ENSG00000005469 | -1.24 | 1.79E-30 |  |  |
| *KRBA2* | KRAB-A domain-containing protein 2 | ENSG00000184619 | -1.24 | 4.72E-14 |  |  |
| *HERC3* | Probable E3 ubiquitin-protein ligase HERC3 | ENSG00000138641 | -1.24 | 8.63E-12 |  |  |
| *CENPJ* | Centromere protein J | ENSG00000151849 | -1.24 | 2.07E-30 |  |  |
| *GPRC5B* | G-protein coupled receptor family C group 5 member B | ENSG00000167191 | -1.23 | 1.72E-16 |  |  |
| *CRIP2* | Cysteine-rich protein 2 | ENSG00000182809 | -1.23 | 3.03E-09 |  |  |
| *GCNT1* | Beta-1,3-galactosyl-O-glycosyl-glycoprotein beta-1,6-N-acetylglucosaminyltransferase | ENSG00000187210 | -1.22 | 1.83E-31 |  |  |
| *GAS6* | Growth arrest-specific protein 6 | ENSG00000183087 | -1.22 | 1.32E-72 |  |  |
| *POC1A* | POC1 centriolar protein homolog A | ENSG00000164087 | -1.22 | 7.87E-13 |  |  |
| *TUBB8* | Tubulin beta-8 chain | ENSG00000261456 | -1.22 | 4.06E-04 |  |  |
| *PODNL1* | Podocan-like protein 1 | ENSG00000132000 | -1.22 | 1.47E-04 |  |  |
| *TXNDC16* | Thioredoxin domain-containing protein 16 | ENSG00000087301 | -1.22 | 3.24E-16 |  |  |
| *CETN3* | Centrin-3 | ENSG00000153140 | -1.22 | 5.77E-28 |  |  |
| *BTBD11* | Ankyrin repeat and BTB/POZ domain-containing protein BTBD11 | ENSG00000151136 | -1.22 | 1.76E-09 |  |  |
| *RBM14* | RNA-binding protein 14 | ENSG00000239306 | -1.22 | 8.40E-32 |  |  |
| *LMNB2* | Lamin-B2 | ENSG00000176619 | -1.22 | 1.02E-32 |  |  |
| *SLC11A1* | Natural resistance-associated macrophage protein 1 | ENSG00000018280 | -1.22 | 5.05E-03 |  |  |
| *HFE* | Hereditary hemochromatosis protein | ENSG00000010704 | -1.22 | 3.64E-30 |  |  |
| *CLDN8* | Claudin-8 | ENSG00000156284 | -1.22 | 0.02 |  |  |
| *FAM151B* | Protein FAM151B | ENSG00000152380 | -1.22 | 7.65E-04 |  |  |
| *DYNC2I2* | Cytoplasmic dynein 2 intermediate chain 2 | ENSG00000119333 | -1.21 | 1.14E-31 |  |  |
| *POU5F1* | POU domain, class 5, transcription factor 1 | ENSG00000204531 | -1.21 | 8.33E-04 |  |  |
| *BRCA1* | Breast cancer type 1 susceptibility protein | ENSG00000012048 | -1.21 | 9.58E-31 |  |  |
| *HAUS1* | HAUS augmin-like complex subunit 1 | ENSG00000152240 | -1.21 | 2.58E-22 |  |  |
| *HYLS1* | Hydrolethalus syndrome protein 1 | ENSG00000198331 | -1.21 | 1.23E-09 |  |  |
| *NASP* | Nuclear autoantigenic sperm protein | ENSG00000132780 | -1.21 | 6.07E-70 |  |  |
| *UBR7* | Putative E3 ubiquitin-protein ligase UBR7 | ENSG00000012963 | -1.21 | 3.45E-30 |  |  |
| *ANKRD34B* | Ankyrin repeat domain-containing protein 34B | ENSG00000189127 | -1.2 | 4.45E-04 |  |  |
| *MN1* | Transcriptional activator MN1 | ENSG00000169184 | -1.2 | 1.87E-10 |  |  |
| *ANAPC15* | Anaphase-promoting complex subunit 15 | ENSG00000110200 | -1.2 | 2.53E-29 |  |  |
| *NACAD* | NAC-alpha domain-containing protein 1 | ENSG00000136274 | -1.2 | 3.79E-04 |  |  |
| *ATP6V1G2-DDX39B* | ATP6V1G2-DDX39B readthrough | ENSG00000254870 | -1.2 | 9.93E-04 |  |  |
| *CDH24* | Cadherin-24 | ENSG00000139880 | -1.2 | 1.95E-21 |  |  |
| *FRRS1* | Ferric-chelate reductase 1 | ENSG00000156869 | -1.2 | 9.94E-30 |  |  |
| *NCAPD3* | Condensin-2 complex subunit D3 | ENSG00000151503 | -1.2 | 4.59E-30 |  |  |
| *TMEM221* | Transmembrane protein 221 | ENSG00000188051 | -1.2 | 0.04 |  |  |
| *GAL3ST4* | Galactose-3-O-sulfotransferase 4 | ENSG00000197093 | -1.2 | 8.40E-03 |  |  |
| *CA2* | Carbonic anhydrase 2 | ENSG00000104267 | -1.2 | 1.92E-64 |  |  |
| *MGAT5B* | Alpha-1,6-mannosylglycoprotein 6-beta-N-acetylglucosaminyltransferase B | ENSG00000167889 | -1.2 | 8.27E-12 |  |  |
| *MOSPD3* | Motile sperm domain-containing protein 3 | ENSG00000106330 | -1.2 | 6.64E-14 |  |  |
| *RBBP8NL* | RBBP8 N-terminal-like protein | ENSG00000130701 | -1.19 | 5.24E-08 |  |  |
| *GBP4* | Guanylate-binding protein 4 | ENSG00000162654 | -1.19 | 9.73E-31 |  |  |
| *PKN3* | Serine/threonine-protein kinase N3 | ENSG00000160447 | -1.19 | 5.20E-27 |  |  |
| *RIPK4* | Receptor-interacting serine/threonine-protein kinase 4 | ENSG00000183421 | -1.19 | 1.17E-68 |  |  |
| *APOBEC3F* | DNA dC->dU-editing enzyme APOBEC-3F | ENSG00000128394 | -1.19 | 1.20E-28 |  |  |
| *SAP30* | Histone deacetylase complex subunit SAP30 | ENSG00000164105 | -1.19 | 4.69E-10 |  |  |
| *HSPB8* | Heat shock protein beta-8 | ENSG00000152137 | -1.19 | 4.47E-07 |  |  |
| *CSAG3* | CSAG Family Member 3 | ENSG00000268916 | -1.19 | 4.26E-07 |  |  |
| *TCF4* | Transcription factor 4 | ENSG00000196628 | -1.19 | 3.38E-05 |  |  |
| *SLC39A10* | Zinc transporter ZIP10 | ENSG00000196950 | -1.19 | 6.89E-28 |  |  |
| *FANCM* | Fanconi anemia group M protein | ENSG00000187790 | -1.19 | 1.85E-26 |  |  |
| *HSPA4L* | Heat shock 70 kDa protein 4L | ENSG00000164070 | -1.19 | 3.45E-28 |  |  |
| *WNT9A* | Protein Wnt-9a | ENSG00000143816 | -1.19 | 8.11E-09 |  |  |
| *MEFV* | Pyrin | ENSG00000103313 | -1.19 | 0.03 |  |  |
| *TEAD2* | Transcriptional enhancer factor TEF-4 | ENSG00000074219 | -1.19 | 1.88E-26 |  |  |
| *ZNF618* | Zinc finger protein 618 | ENSG00000157657 | -1.18 | 5.03E-63 |  |  |
| *DUSP19* | Dual specificity protein phosphatase 19 | ENSG00000162999 | -1.18 | 1.98E-04 |  |  |
| *S100A10* | Protein S100-A10 | ENSG00000197747 | -1.18 | 9.74E-67 |  |  |
| *EFCAB1* | EF-hand calcium-binding domain-containing protein 1 | ENSG00000034239 | -1.18 | 3.07E-04 |  |  |
| *RPL3L* | 60S ribosomal protein L3-like | ENSG00000140986 | -1.18 | 0.03 |  |  |
| *SLURP2* | Secreted Ly-6/uPAR domain-containing protein 2 | ENSG00000283992 | -1.18 | 1.13E-03 |  |  |
| *THOC3* | THO complex subunit 3 | ENSG00000051596 | -1.18 | 1.08E-64 |  |  |
| *EZH2* | Histone-lysine N-methyltransferase EZH2 | ENSG00000106462 | -1.18 | 2.21E-28 |  |  |
| *SP8* | Transcription factor Sp8 | ENSG00000164651 | -1.18 | 1.26E-03 |  |  |
| *CHAF1A* | Chromatin assembly factor 1 subunit A | ENSG00000167670 | -1.18 | 2.65E-29 |  |  |
| *CRISPLD2* | Cysteine-rich secretory protein LCCL domain-containing 2 | ENSG00000103196 | -1.18 | 1.24E-24 |  |  |
| *ANXA2R* | Annexin-2 receptor | ENSG00000177721 | -1.18 | 1.50E-03 |  |  |
| *MDGA1* | MAM domain-containing glycosylphosphatidylinositol anchor protein 1 | ENSG00000112139 | -1.17 | 1.08E-10 |  |  |
| *HERC5* | E3 ISG15--protein ligase HERC5 | ENSG00000138646 | -1.17 | 2.92E-21 |  |  |
| *TUBB* | Tubulin beta chain | ENSG00000196230 | -1.17 | 1.72E-30 |  |  |
| *B3GNT8* | UDP-GlcNAc:betaGal beta-1,3-N-acetylglucosaminyltransferase 8 | ENSG00000177191 | -1.17 | 6.03E-03 |  |  |
| *TRIM55* | Tripartite motif-containing protein 55 | ENSG00000147573 | -1.17 | 4.47E-25 |  |  |
| *DOC2A* | Double C2-like domain-containing protein alpha | ENSG00000149927 | -1.17 | 0.01 |  |  |
| *LRRIQ3* | Leucine-rich repeat and IQ domain-containing protein 3 | ENSG00000162620 | -1.17 | 0.01 |  |  |
| *JRK* | Jerky protein homolog | ENSG00000234616 | -1.17 | 1.16E-26 |  |  |
| *DUT* | Deoxyuridine 5'-triphosphate nucleotidohydrolase, mitochondrial | ENSG00000128951 | -1.17 | 1.23E-61 |  |  |
| *PRRT2* | Proline-rich transmembrane protein 2 | ENSG00000167371 | -1.17 | 9.28E-04 |  |  |
| *DBI* | Acyl-CoA-binding protein | ENSG00000155368 | -1.17 | 4.05E-65 |  |  |
| *MPHOSPH9* | M-phase phosphoprotein 9 | ENSG00000051825 | -1.17 | 1.47E-57 |  |  |
| *NUP37* | Nucleoporin Nup37 | ENSG00000075188 | -1.17 | 2.58E-28 |  |  |
| *SLC16A14* | Monocarboxylate transporter 14 | ENSG00000163053 | -1.17 | 2.92E-12 |  |  |
| *HPGD* | 15-hydroxyprostaglandin dehydrogenase [NAD(+)] | ENSG00000164120 | -1.16 | 4.61E-03 |  |  |
| *AS3MT* | Arsenite methyltransferase | ENSG00000214435 | -1.16 | 1.59E-03 |  |  |
| *NTNG2* | Netrin-G2 | ENSG00000196358 | -1.16 | 2.68E-03 |  |  |
| *YBX2* | Y-box-binding protein 2 | ENSG00000006047 | -1.16 | 2.29E-03 |  |  |
| *FCHO1* | F-BAR domain only protein 1 | ENSG00000130475 | -1.16 | 0.02 |  |  |
| *RFC3* | Replication factor C subunit 3 | ENSG00000133119 | -1.16 | 4.14E-28 |  |  |
| *MAFB* | Transcription factor MafB | ENSG00000204103 | -1.16 | 8.06E-04 |  |  |
| *PRADC1* | Protease-associated domain-containing protein 1 | ENSG00000135617 | -1.16 | 3.27E-08 |  |  |
| *OTULINL* | Inactive ubiquitin thioesterase OTULINL | ENSG00000145569 | -1.16 | 7.40E-03 |  |  |
| *C12orf60* | Uncharacterized protein C12orf60 | ENSG00000182993 | -1.16 | 3.82E-03 |  |  |
| *IGFBP6* | Insulin-like growth factor-binding protein 6 | ENSG00000167779 | -1.16 | 4.75E-26 |  |  |
| *WNT4* | Protein Wnt-4 | ENSG00000162552 | -1.16 | 6.43E-15 |  |  |
| *PRIM2* | DNA primase large subunit | ENSG00000146143 | -1.16 | 3.21E-27 |  |  |
| *POLR2L* | DNA-directed RNA polymerases I, II, and III subunit RPABC5 | ENSG00000177700 | -1.16 | 7.67E-28 |  |  |
| *LYPD6* | Ly6/PLAUR domain-containing protein 6 | ENSG00000187123 | -1.15 | 2.54E-24 |  |  |
| *P2RY2* | P2Y purinoceptor 2 | ENSG00000175591 | -1.15 | 3.04E-28 |  |  |
| *ZNF273* | Zinc finger protein 273 | ENSG00000198039 | -1.15 | 3.04E-11 |  |  |
| *MCM8* | DNA helicase MCM8 | ENSG00000125885 | -1.15 | 1.02E-27 |  |  |
| *SLX1A* | Structure-specific endonuclease subunit SLX1 | ENSG00000132207 | -1.15 | 1.27E-28 |  |  |
| *MASTL* | Serine/threonine-protein kinase greatwall | ENSG00000120539 | -1.15 | 6.19E-26 |  |  |
| *SLC35F3* | Putative thiamine transporter SLC35F3 | ENSG00000183780 | -1.15 | 1.17E-05 |  |  |
| *SLC9A5* | Sodium/hydrogen exchanger 5 | ENSG00000135740 | -1.15 | 1.37E-03 |  |  |
| *CENPS* | Centromere protein S | ENSG00000175279 | -1.15 | 3.78E-24 |  |  |
| *PRRG4* | Transmembrane gamma-carboxyglutamic acid protein 4 | ENSG00000135378 | -1.15 | 4.51E-63 |  |  |
| *KLRG2* | Killer cell lectin-like receptor subfamily G member 2 | ENSG00000188883 | -1.14 | 2.39E-03 |  |  |
| *FAM167B* | Family With Sequence Similarity 167 Member B | ENSG00000183615 | -1.14 | 0.04 |  |  |
| *RASSF10* | Ras association domain-containing protein 10 | ENSG00000189431 | -1.14 | 2.99E-03 |  |  |
| *HAUS5* | HAUS augmin-like complex subunit 5 | ENSG00000249115 | -1.14 | 5.27E-26 |  |  |
| *LRRC19* | Leucine-rich repeat-containing protein 19 | ENSG00000184434 | -1.14 | 5.45E-03 |  |  |
| *SUOX* | Sulfite oxidase, mitochondrial | ENSG00000139531 | -1.14 | 1.94E-24 |  |  |
| *PNPLA3* | 1-acylglycerol-3-phosphate O-acyltransferase PNPLA3 | ENSG00000100344 | -1.14 | 7.40E-11 |  |  |
| *PLK2* | Serine/threonine-protein kinase PLK2 | ENSG00000145632 | -1.14 | 1.23E-28 |  |  |
| *C1orf112* | Uncharacterized protein C1orf112 | ENSG00000000460 | -1.14 | 8.87E-26 |  |  |
| *VRK1* | Serine/threonine-protein kinase VRK1 | ENSG00000100749 | -1.13 | 1.50E-26 |  |  |
| *EYA1* | Eyes absent homolog 1 | ENSG00000104313 | -1.13 | 1.89E-12 |  |  |
| *KYAT1* | Kynurenine--oxoglutarate transaminase 1 | ENSG00000171097 | -1.13 | 2.13E-15 |  |  |
| *FAXDC2* | Fatty acid hydroxylase domain-containing protein 2 | ENSG00000170271 | -1.13 | 1.97E-03 |  |  |
| *GPR85* | Probable G-protein coupled receptor 85 | ENSG00000164604 | -1.13 | 2.97E-14 |  |  |
| *POLA1* | DNA polymerase alpha catalytic subunit | ENSG00000101868 | -1.13 | 1.76E-26 |  |  |
| *CDCA4* | Cell division cycle-associated protein 4 | ENSG00000170779 | -1.13 | 6.10E-27 |  |  |
| *MTHFD1* | C-1-tetrahydrofolate synthase, cytoplasmic | ENSG00000100714 | -1.13 | 1.45E-61 |  |  |
| *ZNF684* | Zinc finger protein 684 | ENSG00000117010 | -1.13 | 6.24E-05 |  |  |
| *FAT3* | Protocadherin Fat 3 | ENSG00000165323 | -1.12 | 4.61E-03 |  |  |
| *SF3A3* | Splicing factor 3A subunit 3 | ENSG00000183431 | -1.12 | 2.79E-60 |  |  |
| *SH3D21* | SH3 domain-containing protein 21 | ENSG00000214193 | -1.12 | 9.31E-25 |  |  |
| *ISYNA1* | Inositol-3-phosphate synthase 1 | ENSG00000105655 | -1.12 | 3.39E-27 |  |  |
| *TMEM106C* | Transmembrane protein 106C | ENSG00000134291 | -1.12 | 5.94E-27 |  |  |
| *CCDC77* | Coiled-coil domain-containing protein 77 | ENSG00000120647 | -1.12 | 8.08E-19 |  |  |
| *CDH13* | Cadherin-13 | ENSG00000140945 | -1.12 | 5.14E-26 |  |  |
| *LSM7* | U6 snRNA-associated Sm-like protein LSm7 | ENSG00000130332 | -1.12 | 4.03E-52 |  |  |
| *H2AZ1* | Histone H2A.Z | ENSG00000164032 | -1.12 | 4.01E-27 |  |  |
| *CASTOR2* | Cytosolic arginine sensor for mTORC1 subunit 2 | ENSG00000274070 | -1.12 | 9.39E-24 |  |  |
| *CHRNA5* | Neuronal acetylcholine receptor subunit alpha-5 | ENSG00000169684 | -1.12 | 3.00E-10 |  |  |
| *NDUFA2* | NADH dehydrogenase [ubiquinone] 1 alpha subcomplex subunit 2 | ENSG00000131495 | -1.12 | 2.38E-25 |  |  |
| *ANP32E* | Acidic leucine-rich nuclear phosphoprotein 32 family member E | ENSG00000143401 | -1.12 | 5.44E-27 |  |  |
| *CEP128* | Centrosomal protein of 128 kDa | ENSG00000100629 | -1.12 | 1.14E-12 |  |  |
| *CCDC8* | Coiled-coil domain-containing protein 8 | ENSG00000169515 | -1.11 | 8.67E-15 |  |  |
| *KCNK2* | Potassium channel subfamily K member 2 | ENSG00000082482 | -1.11 | 6.44E-05 |  |  |
| *TAF5* | Transcription initiation factor TFIID subunit 5 | ENSG00000148835 | -1.11 | 7.65E-13 |  |  |
| *SLC44A3* | Choline transporter-like protein 3 | ENSG00000143036 | -1.11 | 1.79E-23 |  |  |
| *RAB30* | Ras-related protein Rab-30 | ENSG00000137502 | -1.11 | 1.99E-25 |  |  |
| *ICOSLG* | ICOS ligand | ENSG00000160223 | -1.11 | 3.70E-24 |  |  |
| *SLF1* | SMC5-SMC6 complex localization factor protein 1 | ENSG00000133302 | -1.11 | 4.98E-24 |  |  |
| *ZNF804A* | Zinc finger protein 804A | ENSG00000170396 | -1.11 | 0.01 |  |  |
| *DZIP3* | E3 ubiquitin-protein ligase DZIP3 | ENSG00000198919 | -1.11 | 5.98E-15 |  |  |
| *TMEM59L* | Transmembrane protein 59-like | ENSG00000105696 | -1.11 | 0.03 |  |  |
| *GPD2* | Glycerol-3-phosphate dehydrogenase, mitochondrial | ENSG00000115159 | -1.11 | 5.74E-60 |  |  |
| *WDR54* | WD repeat-containing protein 54 | ENSG00000005448 | -1.11 | 1.29E-23 |  |  |
| *PMEL* | Melanocyte protein PMEL | ENSG00000185664 | -1.11 | 2.09E-03 |  |  |
| *COL5A2* | Collagen alpha-2(V) chain | ENSG00000204262 | -1.11 | 6.99E-03 |  |  |
| *DTYMK* | Thymidylate kinase | ENSG00000168393 | -1.1 | 3.67E-25 |  |  |
| *APCDD1L* | Protein APCDD1-like | ENSG00000198768 | -1.1 | 1.33E-08 |  |  |
| *MVK* | Mevalonate kinase | ENSG00000110921 | -1.1 | 1.06E-53 |  |  |
| *CKS1B* | Cyclin-dependent kinases regulatory subunit 1 | ENSG00000173207 | -1.1 | 1.91E-26 |  |  |
| *ALX4* | Homeobox protein aristaless-like 4 | ENSG00000052850 | -1.1 | 2.87E-23 |  |  |
| *GJC1* | Gap junction gamma-1 protein | ENSG00000182963 | -1.1 | 1.56E-24 |  |  |
| *DONSON* | Protein downstream neighbor of Son | ENSG00000159147 | -1.1 | 2.61E-24 |  |  |
| *HMGB1* | High mobility group protein B1 | ENSG00000189403 | -1.1 | 8.45E-27 |  |  |
| *SEMA3F* | Semaphorin-3F | ENSG00000001617 | -1.1 | 9.00E-25 |  |  |
| *GVQW3* | GVQW Motif Containing 3 | ENSG00000179240 | -1.1 | 2.22E-12 |  |  |
| *COL13A1* | Collagen alpha-1(XIII) chain | ENSG00000197467 | -1.1 | 2.37E-03 |  |  |
| *HSP90AA1* | Heat shock protein HSP 90-alpha | ENSG00000080824 | -1.09 | 9.66E-27 |  |  |
| *ECM1* | Extracellular matrix protein 1 | ENSG00000143369 | -1.09 | 7.94E-03 |  |  |
| *HPSE* | Heparanase | ENSG00000173083 | -1.09 | 6.70E-22 |  |  |
| *PLA2G4A* | Cytosolic phospholipase A2 | ENSG00000116711 | -1.09 | 1.43E-09 |  |  |
| *CISH* | Cytokine-inducible SH2-containing protein | ENSG00000114737 | -1.09 | 2.59E-03 |  |  |
| *ADAM22* | Disintegrin and metalloproteinase domain-containing protein 22 | ENSG00000008277 | -1.09 | 5.11E-11 |  |  |
| *TCTN2* | Tectonic-2 | ENSG00000168778 | -1.09 | 6.53E-24 |  |  |
| *GPM6A* | Neuronal membrane glycoprotein M6-a | ENSG00000150625 | -1.08 | 0.03 |  |  |
| *PDLIM2* | PDZ and LIM domain protein 2 | ENSG00000120913 | -1.08 | 5.26E-06 |  |  |
| *USP30* | Ubiquitin carboxyl-terminal hydrolase 30 | ENSG00000135093 | -1.08 | 3.91E-22 |  |  |
| *CYP1B1* | Cytochrome P450 1B1 | ENSG00000138061 | -1.08 | 1.18E-56 |  |  |
| *ATP5MC3* | ATP synthase F(0) complex subunit C3, mitochondrial | ENSG00000154518 | -1.08 | 8.01E-57 |  |  |
| *TCF7L1* | Transcription factor 7-like 1 | ENSG00000152284 | -1.08 | 1.84E-21 |  |  |
| *TMEM97* | Sigma intracellular receptor 2 | ENSG00000109084 | -1.08 | 1.44E-23 |  |  |
| *KREMEN1* | Kremen protein 1 | ENSG00000183762 | -1.08 | 2.88E-53 |  |  |
| *TMEM143* | Transmembrane protein 143 | ENSG00000161558 | -1.08 | 3.07E-08 |  |  |
| *SIRT4* | NAD-dependent protein lipoamidase sirtuin-4, mitochondrial | ENSG00000089163 | -1.08 | 0.02 |  |  |
| *C8orf88* | Uncharacterized protein C8orf88 | ENSG00000253250 | -1.07 | 0.02 |  |  |
| *SERPINB13* | Serpin B13 | ENSG00000197641 | -1.07 | 7.69E-55 |  |  |
| *SH3BP1* | SH3 domain-binding protein 1 | ENSG00000100092 | -1.07 | 4.45E-25 |  |  |
| *SLC35D1* | UDP-glucuronic acid/UDP-N-acetylgalactosamine transporter | ENSG00000116704 | -1.07 | 4.14E-48 |  |  |
| *PRAME* | Melanoma antigen preferentially expressed in tumors | ENSG00000185686 | -1.07 | 4.71E-07 |  |  |
| *H2AX* | Histone H2AX | ENSG00000188486 | -1.07 | 1.95E-23 |  |  |
| *PXMP2* | Peroxisomal membrane protein 2 | ENSG00000176894 | -1.07 | 3.94E-14 |  |  |
| *C4orf33* | UPF0462 protein C4orf33 | ENSG00000151470 | -1.07 | 3.09E-09 |  |  |
| *GPC1* | Glypican-1 | ENSG00000063660 | -1.07 | 1.40E-55 |  |  |
| *SSBP3* | Single-stranded DNA-binding protein 3 | ENSG00000157216 | -1.06 | 1.82E-24 |  |  |
| *TRIM6* | Tripartite motif-containing protein 6 | ENSG00000121236 | -1.06 | 1.17E-22 |  |  |
| *BTG2* | Protein BTG2 | ENSG00000159388 | -1.06 | 1.12E-15 |  |  |
| *FHOD1* | FH1/FH2 domain-containing protein 1 | ENSG00000135723 | -1.06 | 7.89E-23 |  |  |
| *STARD5* | StAR-related lipid transfer protein 5 | ENSG00000172345 | -1.06 | 6.66E-12 |  |  |
| *NEMP1* | Nuclear envelope integral membrane protein 1 | ENSG00000166881 | -1.06 | 2.90E-24 |  |  |
| *POP1* | Ribonucleases P/MRP protein subunit POP1 | ENSG00000104356 | -1.06 | 7.14E-16 |  |  |
| *SMOC2* | SPARC-related modular calcium-binding protein 2 | ENSG00000112562 | -1.06 | 5.87E-03 |  |  |
| *FEN1* | Flap endonuclease 1 | ENSG00000168496 | -1.06 | 5.95E-24 |  |  |
| *DMBX1* | Diencephalon/mesencephalon homeobox protein 1 | ENSG00000197587 | -1.06 | 0.04 |  |  |
| *KANK2* | KN motif and ankyrin repeat domain-containing protein 2 | ENSG00000197256 | -1.06 | 1.24E-22 |  |  |
| *NAAA* | N-acylethanolamine-hydrolyzing acid amidase | ENSG00000138744 | -1.06 | 2.87E-11 |  |  |
| *RAD51AP2* | RAD51-associated protein 2 | ENSG00000214842 | -1.06 | 0.04 |  |  |
| *ZBED2* | Zinc finger BED domain-containing protein 2 | ENSG00000177494 | -1.06 | 8.59E-09 |  |  |
| *NT5DC2* | 5'-nucleotidase domain-containing protein 2 | ENSG00000168268 | -1.05 | 1.51E-22 |  |  |
| *COL7A1* | Collagen alpha-1(VII) chain | ENSG00000114270 | -1.05 | 2.81E-24 |  |  |
| *WRAP53* | Telomerase Cajal body protein 1 | ENSG00000141499 | -1.05 | 1.11E-21 |  |  |
| *NTHL1* | Endonuclease III-like protein 1 | ENSG00000065057 | -1.05 | 6.18E-21 |  |  |
| *CYGB* | Cytoglobin | ENSG00000161544 | -1.05 | 5.96E-05 |  |  |
| *VAMP5* | Vesicle-associated membrane protein 5 | ENSG00000168899 | -1.05 | 5.77E-07 |  |  |
| *HMGN2* | Non-histone chromosomal protein HMG-17 | ENSG00000198830 | -1.05 | 2.26E-24 |  |  |
| *CMC2* | COX assembly mitochondrial protein 2 homolog | ENSG00000103121 | -1.05 | 4.17E-22 |  |  |
| *EPB41L1* | Band 4.1-like protein 1 | ENSG00000088367 | -1.04 | 2.02E-51 |  |  |
| *RFX7* | DNA-binding protein RFX7 | ENSG00000181827 | -1.04 | 8.15E-22 |  |  |
| *CFAP126* | Protein Flattop | ENSG00000188931 | -1.04 | 5.67E-03 |  |  |
| *TRERF1* | Transcriptional-regulating factor 1 | ENSG00000124496 | -1.04 | 1.13E-22 |  |  |
| *KCNIP3* | Calsenilin | ENSG00000115041 | -1.04 | 8.30E-03 |  |  |
| *NDUFB6* | NADH dehydrogenase [ubiquinone] 1 beta subcomplex subunit 6 | ENSG00000165264 | -1.04 | 1.60E-44 |  |  |
| *ATP5ME* | ATP synthase subunit e, mitochondrial | ENSG00000169020 | -1.04 | 1.15E-21 |  |  |
| *LSS* | Lanosterol synthase | ENSG00000160285 | -1.04 | 2.86E-53 |  |  |
| *GPR37L1* | G-protein coupled receptor 37-like 1 | ENSG00000170075 | -1.04 | 0.04 |  |  |
| *CASTOR1* | Cytosolic arginine sensor for mTORC1 subunit 1 | ENSG00000239282 | -1.04 | 6.31E-10 |  |  |
| *TRNAU1AP* | tRNA selenocysteine 1-associated protein 1 | ENSG00000180098 | -1.04 | 2.34E-20 |  |  |
| *FAT2* | Protocadherin Fat 2 | ENSG00000086570 | -1.04 | 5.59E-24 |  |  |
| *ARRB1* | Beta-arrestin-1 | ENSG00000137486 | -1.03 | 3.62E-09 |  |  |
| *C12orf75* | Chromosome 12 Open Reading Frame 75 | ENSG00000235162 | -1.03 | 2.04E-21 |  |  |
| *IFITM10* | Interferon-induced transmembrane protein 10 | ENSG00000244242 | -1.03 | 0.04 |  |  |
| *SCD5* | Stearoyl-CoA desaturase 5 | ENSG00000145284 | -1.03 | 2.20E-20 |  |  |
| *ACLY* | ATP-citrate synthase | ENSG00000131473 | -1.03 | 1.20E-52 |  |  |
| *SYNM* | Synemin | ENSG00000182253 | -1.03 | 6.48E-21 |  |  |
| *CARD14* | Caspase recruitment domain-containing protein 14 | ENSG00000141527 | -1.03 | 3.85E-22 |  |  |
| *EEF1A2* | Elongation factor 1-alpha 2 | ENSG00000101210 | -1.03 | 3.01E-03 |  |  |
| *RNASEK-C17orf49* | RNASEK-C17orf49 readthrough (Fragment) | ENSG00000161939 | -1.03 | 0.02 |  |  |
| *APOBEC3G* | DNA dC->dU-editing enzyme APOBEC-3G | ENSG00000239713 | -1.03 | 3.73E-22 |  |  |
| *CASP6* | Caspase-6 | ENSG00000138794 | -1.03 | 3.08E-16 |  |  |
| *SNRPF* | Small nuclear ribonucleoprotein F | ENSG00000139343 | -1.03 | 6.68E-48 |  |  |
| *ZNF395* | Zinc finger protein 395 | ENSG00000186918 | -1.03 | 3.08E-48 |  |  |
| *SBSPON* | Somatomedin-B and thrombospondin type-1 domain-containing protein | ENSG00000164764 | -1.02 | 0.03 |  |  |
| *IL6R* | Interleukin-6 receptor subunit alpha | ENSG00000160712 | -1.02 | 3.67E-21 |  |  |
| *TMA7* | Translation Machinery Associated 7 Homolog | ENSG00000232112 | -1.02 | 1.84E-21 |  |  |
| *SHMT1* | Serine hydroxymethyltransferase, cytosolic | ENSG00000176974 | -1.02 | 4.25E-22 |  |  |
| *SNAI3* | Zinc finger protein SNAI3 | ENSG00000185669 | -1.02 | 6.81E-03 |  |  |
| *MREG* | Melanoregulin | ENSG00000118242 | -1.02 | 1.18E-21 |  |  |
| *TUBG1* | Tubulin gamma-1 chain | ENSG00000131462 | -1.02 | 1.81E-22 |  |  |
| *CXCL1* | Growth-regulated alpha protein | ENSG00000163739 | -1.02 | 3.87E-23 |  |  |
| *CSF1* | Macrophage colony-stimulating factor 1 | ENSG00000184371 | -1.02 | 5.28E-50 |  |  |
| *NDUFB3* | NADH dehydrogenase [ubiquinone] 1 beta subcomplex subunit 3 | ENSG00000119013 | -1.02 | 2.33E-21 |  |  |
| *CCDC34* | Coiled-Coil Domain Containing 34 | ENSG00000109881 | -1.02 | 2.35E-20 |  |  |
| *LIX1L* | LIX1-like protein | ENSG00000271601 | -1.02 | 3.05E-21 |  |  |
| *TERT* | Telomerase reverse transcriptase | ENSG00000164362 | -1.02 | 2.96E-05 |  |  |
| *KCNN4* | Intermediate conductance calcium-activated potassium channel protein 4 | ENSG00000104783 | -1.02 | 6.95E-08 |  |  |
| *GEN1* | Flap endonuclease GEN homolog 1 | ENSG00000178295 | -1.01 | 8.81E-21 |  |  |
| *TGIF2* | Homeobox protein TGIF2 | ENSG00000118707 | -1.01 | 1.51E-21 |  |  |
| *HSPB1* | Heat shock protein beta-1 | ENSG00000106211 | -1.01 | 5.22E-23 |  |  |
| *TLR5* | Toll-like receptor 5 | ENSG00000187554 | -1.01 | 1.28E-10 |  |  |
| *DEPDC4* | DEP domain-containing protein 4 | ENSG00000166153 | -1.01 | 4.52E-03 |  |  |
| *RFC4* | Replication factor C subunit 4 | ENSG00000163918 | -1.01 | 5.32E-46 |  |  |
| *GSTM2* | Glutathione S-transferase Mu 2 | ENSG00000213366 | -1.01 | 1.69E-03 |  |  |
| *MAGED1* | Melanoma-associated antigen D1 | ENSG00000179222 | -1.01 | 5.46E-08 |  |  |
| *IL17RE* | Interleukin-17 receptor E | ENSG00000163701 | -1.01 | 1.25E-05 |  |  |
| *HLA-DMA* | HLA class II histocompatibility antigen, DM alpha chain | ENSG00000204257 | -1.01 | 4.04E-20 |  |  |
| *UCN2* | Urocortin-2 | ENSG00000145040 | -1.01 | 7.66E-03 |  |  |
| *CYP4B1* | Cytochrome P450 4B1 | ENSG00000142973 | -1 | 0.02 |  |  |
| *LRRC45* | Leucine-rich repeat-containing protein 45 | ENSG00000169683 | -1 | 1.11E-19 |  |  |
| *POGLUT3* | Protein O-glucosyltransferase 3 | ENSG00000178202 | -1 | 2.62E-19 |  |  |
| *FBXO48* | F-box only protein 48 | ENSG00000204923 | -1 | 1.59E-08 |  |  |
| *SV2A* | Synaptic vesicle glycoprotein 2A | ENSG00000159164 | -1 | 7.23E-03 |  |  |
| *SOGA1* | Protein SOGA1 | ENSG00000149639 | -1 | 4.62E-22 |  |  |
