# Supplementary material for: EMILIN-1 Suppresses Cell Proliferation through Altered Cell Cycle Regulation in Head and Neck Squamous Cell Carcinoma
Source: Am J Pathol. 2025 Jan 30;195(5):995–1012. doi: 10.1016/j.ajpath.2025.01.010 (PMC12163418; doi:10.1016/j.ajpath.2025.01.010)
Supplement: Supplemental Table S5 [file mmc5.docx]

| **Supplemental Table S5** Upregulated genes of CAF2 cell with EMILIN-1 knockdown (Log2FC>0.6,FDR<0.05). (https://www.ensembl.org) | | | | |
| --- | --- | --- | --- | --- |
|  |  |  |  |  |
| **Gene** | **Database name** | **Identifier** | **Log2FC** | **FDR p-value** |
| *ADH1B* | All-trans-retinol dehydrogenase [NAD(+)] ADH1B | ENSG00000196616 | 8.805973 | 0.00000367 |
| *EGFL6* | Epidermal growth factor-like protein 6 | ENSG00000198759 | 5.910714 | 0.001084 |
| *DLL4* | Delta-like protein 4 | ENSG00000128917 | 5.279044 | 0.000792 |
| *FAM20A* | Pseudokinase FAM20A | ENSG00000108950 | 5.206192 | 0.0000926 |
| *XPNPEP2* | Xaa-Pro aminopeptidase 2 | ENSG00000122121 | 5.007826 | 0.003044 |
| *HBD* | Hemoglobin subunit delta | ENSG00000223609 | 4.96452 | 0.000335 |
| *GUCY1A1* | Guanylate cyclase soluble subunit alpha-1 | ENSG00000164116 | 4.877533 | 0.025566 |
| *GTF2A1L* | TFIIA-alpha and beta-like factor | ENSG00000242441 | 4.563811 | 0.045144 |
| *OMD* | Osteomodulin | ENSG00000127083 | 4.522722 | 0.008801 |
| *NTN1* | Netrin-1 | ENSG00000065320 | 3.502415 | 0.000881 |
| *TNXB* | Tenascin-X | ENSG00000168477 | 3.327097 | 0.007617 |
| *PLXNC1* | Plexin-C1 | ENSG00000136040 | 2.73101 | 0.024235 |
| *EPHB6* | Ephrin type-B receptor 6 | ENSG00000106123 | 2.528144 | 0.011201 |
| *DEPP1* | Protein DEPP1 | ENSG00000165507 | 2.453479 | 0.00996 |
| *PTGDS* | Prostaglandin-H2 D-isomerase | ENSG00000107317 | 2.385522 | 0.001583 |
| *MAN1C1* | Mannosyl-oligosaccharide 1,2-alpha-mannosidase IC | ENSG00000117643 | 2.263708 | 0.003891 |
| *A2M* | Alpha-2-macroglobulin | ENSG00000175899 | 2.012139 | 0.005757 |
| *VCAM1* | Vascular cell adhesion protein 1 | ENSG00000162692 | 1.848676 | 0.011201 |
| *GPX3* | Glutathione peroxidase 3 | ENSG00000211445 | 1.502995 | 0.027936 |
| *PLPP3* | Phospholipid phosphatase 3 | ENSG00000162407 | 1.296095 | 0.021863 |
| *SNAP23* | Synaptosomal-associated protein 23 | ENSG00000196616 | 0.966206 | 0.005391 |
| *NPR3* | Atrial natriuretic peptide receptor 3 | ENSG00000198759 | 0.852927 | 0.035904 |
