# Supplementary material for: EMILIN-1 Suppresses Cell Proliferation through Altered Cell Cycle Regulation in Head and Neck Squamous Cell Carcinoma
Source: Am J Pathol. 2025 Jan 30;195(5):995–1012. doi: 10.1016/j.ajpath.2025.01.010 (PMC12163418; doi:10.1016/j.ajpath.2025.01.010)
Supplement: Supplemental Table S6 [file mmc6.docx]

| **Supplemental Table S6** Downregulated genes of CAF2 cell with EMILIN-1 knockdown (Log2FC<-0.6,FDR<0.05). ( (https://www.ensembl.org) | | | | |
| --- | --- | --- | --- | --- |
|  |  |  |  |  |
| **Gene** | **Database name** | **Identifier** | **Log2FC** | **FDR p-value** |
| *EMILIN1* | Elastin microfibril interfacer 1 | ENSG00000138080 | -3.75 | 2.41E-15 |
| *CEND1* | Cell cycle exit and neuronal differentiation protein 1 | ENSG00000184524 | -1.99 | 0.03 |
| *TNC* | Tenascin | ENSG00000041982 | -1.91 | 1.30E-08 |
| *LMNB1* | Lamin-B1 | ENSG00000113368 | -1.58 | 6.68E-03 |
| *LRRC15* | Leucine-rich repeat-containing protein 15 | ENSG00000172061 | -1.56 | 1.31E-04 |
| *F3* | Tissue factor | ENSG00000117525 | -1.52 | 9.54E-04 |
| *MMP1* | Interstitial collagenase | ENSG00000196611 | -1.48 | 6.22E-04 |
| *CDCP1* | CUB domain-containing protein 1 | ENSG00000163814 | -1.45 | 2.81E-03 |
| *TMEM158* | Transmembrane protein 158 | ENSG00000249992 | -1.27 | 3.59E-04 |
| *CDKN2B* | Cyclin-dependent kinase 4 inhibitor B | ENSG00000147883 | -1.25 | 4.18E-04 |
| *TMEM130* | Transmembrane protein 130 | ENSG00000166448 | -1.14 | 8.56E-04 |
| *NES* | Nestin | ENSG00000132688 | -1.11 | 7.47E-04 |
| *AKT3* | RAC-gamma serine/threonine-protein kinase | ENSG00000117020 | -1.03 | 0.01 |
| *SOAT1* | Sterol O-acyltransferase 1 | ENSG00000057252 | -1.03 | 7.47E-04 |
| *PSG1* | Pregnancy-specific beta-1-glycoprotein 1 | ENSG00000231924 | -1.01 | 6.78E-03 |
