# Supplementary material for: EMILIN-1 Suppresses Cell Proliferation through Altered Cell Cycle Regulation in Head and Neck Squamous Cell Carcinoma
Source: Am J Pathol. 2025 Jan 30;195(5):995–1012. doi: 10.1016/j.ajpath.2025.01.010 (PMC12163418; doi:10.1016/j.ajpath.2025.01.010)
Supplement: Supplemental Table S7 [file mmc7.docx]

| **Supplemental Table S7** Upregulated genes of NF2 cell with EMILIN-1 knockdown (Log2FC>1,FDR<0.05). (https://www.ensembl.org) | | | |  |
| --- | --- | --- | --- | --- |
|  |  |  |  |  |
| **Gene** | **Database name** | **Identifier** | **Log2FC** | **FDR p-value** |
| *MAB21L2* | Protein mab-21-like 2 | ENSG00000181541 | 11.3 | 1.83E-03 |
| *TFAP2B* | Transcription factor AP-2-beta | ENSG00000008196 | 9.27 | 0.02 |
| *KRT14* | Keratin, type I cytoskeletal 14 | ENSG00000186847 | 8.13 | 0.05 |
| *PCSK2* | Neuroendocrine convertase 2 | ENSG00000125851 | 6.38 | 1.26E-05 |
| *IVL* | Involucrin | ENSG00000163207 | 6.2 | 0.02 |
| *PTPN20* | Tyrosine-protein phosphatase non-receptor type 20 | ENSG00000204179 | 6.06 | 0.02 |
| *SIM2* | Single-minded homolog 2 | ENSG00000159263 | 5.71 | 7.40E-06 |
| *NTSR1* | Neurotensin receptor type 1 | ENSG00000101188 | 5.65 | 2.33E-04 |
| *NR5A2* | Nuclear receptor subfamily 5 group A member 2 | ENSG00000116833 | 5.5 | 4.73E-05 |
| *TMEM200C* | Transmembrane protein 200C | ENSG00000206432 | 5.28 | 0.03 |
| *PROM1* | Prominin-1 | ENSG00000007062 | 4.91 | 3.12E-03 |
| *RELN* | Reelin | ENSG00000189056 | 4.65 | 8.37E-06 |
| *TENM2* | Teneurin-2 | ENSG00000145934 | 4.65 | 2.38E-04 |
| *SP9* | Transcription factor Sp9 | ENSG00000217236 | 4.62 | 4.27E-03 |
| *CYP24A1* | 1,25-dihydroxyvitamin D(3) 24-hydroxylase, mitochondrial | ENSG00000019186 | 4.55 | 5.70E-03 |
| *LCE1F* | Late cornified envelope protein 1F | ENSG00000240386 | 4.48 | 5.43E-03 |
| *CHL1* | Neural cell adhesion molecule L1-like protein | ENSG00000134121 | 4.45 | 6.05E-03 |
| *NEFM* | Neurofilament medium polypeptide | ENSG00000104722 | 4.36 | 4.73E-05 |
| *EPHA3* | Ephrin type-A receptor 3 | ENSG00000044524 | 4.27 | 0.04 |
| *PI16* | Peptidase inhibitor 16 | ENSG00000164530 | 4.21 | 1.12E-05 |
| *SALL3* | Sal-like protein 3 | ENSG00000256463 | 4.03 | 0.01 |
| *NETO1* | Neuropilin and tolloid-like protein 1 | ENSG00000166342 | 4 | 0.01 |
| *NPTX2* | Neuronal pentraxin-2 | ENSG00000106236 | 3.8 | 1.98E-03 |
| *GSTT2* | Glutathione S-transferase theta-2 | ENSG00000099984 | 3.77 | 0.04 |
| *IL13RA2* | Interleukin-13 receptor subunit alpha-2 | ENSG00000123496 | 3.69 | 2.20E-05 |
| *PITX1* | Pituitary homeobox 1 | ENSG00000069011 | 3.52 | 1.72E-03 |
| *SCIN* | Adseverin | ENSG00000006747 | 3.42 | 5.27E-03 |
| *TSPAN2* | Tetraspanin-2 | ENSG00000134198 | 3.37 | 3.08E-03 |
| *BARX1* | Homeobox protein BarH-like 1 | ENSG00000131668 | 3.3 | 6.82E-04 |
| *IGFBP3* | Insulin-like growth factor-binding protein 3 | ENSG00000146674 | 3.25 | 3.02E-05 |
| *PAX9* | Paired box protein Pax-9 | ENSG00000198807 | 3.24 | 2.64E-03 |
| *PODXL* | Podocalyxin | ENSG00000128567 | 3.19 | 1.08E-05 |
| *NPIPA8* | Nuclear pore complex-interacting protein family member A8 | ENSG00000214940 | 2.96 | 0.02 |
| *SEMA3E* | Semaphorin-3E | ENSG00000170381 | 2.94 | 3.83E-03 |
| *SCN5A* | Sodium channel protein type 5 subunit alpha | ENSG00000183873 | 2.78 | 0.04 |
| *KCNQ5* | Potassium voltage-gated channel subfamily KQT member 5 | ENSG00000185760 | 2.76 | 1.18E-04 |
| *THBD* | Thrombomodulin | ENSG00000178726 | 2.75 | 0.03 |
| *NEK2* | Serine/threonine-protein kinase Nek2 | ENSG00000117650 | 2.68 | 9.60E-05 |
| *TROAP* | Tastin | ENSG00000135451 | 2.67 | 1.08E-05 |
| *ASPM* | Abnormal spindle-like microcephaly-associated protein | ENSG00000066279 | 2.67 | 9.42E-06 |
| *PLK1* | Serine/threonine-protein kinase PLK1 | ENSG00000166851 | 2.66 | 1.05E-05 |
| *E2F8* | Transcription factor E2F8 | ENSG00000129173 | 2.66 | 9.75E-03 |
| *DLGAP5* | Disks large-associated protein 5 | ENSG00000126787 | 2.64 | 2.68E-05 |
| *GTSE1* | G2 and S phase-expressed protein 1 | ENSG00000075218 | 2.64 | 2.68E-05 |
| *KIF14* | Kinesin-like protein KIF14 | ENSG00000118193 | 2.62 | 2.68E-05 |
| *HJURP* | Holliday junction recognition protein | ENSG00000123485 | 2.61 | 2.11E-05 |
| *IQGAP3* | Ras GTPase-activating-like protein IQGAP3 | ENSG00000183856 | 2.59 | 8.37E-06 |
| *CDC25C* | M-phase inducer phosphatase 3 | ENSG00000158402 | 2.58 | 1.32E-03 |
| *BUB1B* | Mitotic checkpoint serine/threonine-protein kinase BUB1 beta | ENSG00000156970 | 2.57 | 2.72E-05 |
| *MKI67* | Proliferation marker protein Ki-67 | ENSG00000148773 | 2.54 | 2.03E-05 |
| *BUB1* | Mitotic checkpoint serine/threonine-protein kinase BUB1 | ENSG00000169679 | 2.53 | 1.35E-05 |
| *DES* | Desmin | ENSG00000175084 | 2.49 | 0.02 |
| *KIF18B* | Kinesin-like protein KIF18B | ENSG00000186185 | 2.48 | 5.15E-05 |
| *ESM1* | Endothelial cell-specific molecule 1 | ENSG00000164283 | 2.48 | 1.33E-03 |
| *NUF2* | Kinetochore protein Nuf2 | ENSG00000143228 | 2.48 | 1.45E-04 |
| *APOBEC3B* | DNA dC->dU-editing enzyme APOBEC-3B | ENSG00000179750 | 2.47 | 4.62E-04 |
| *ANLN* | Anillin | ENSG00000011426 | 2.47 | 4.73E-05 |
| *ACTC1* | Actin, alpha cardiac muscle 1 | ENSG00000159251 | 2.46 | 0.02 |
| *TFPI2* | Tissue factor pathway inhibitor 2 | ENSG00000105825 | 2.45 | 0.02 |
| *ERCC6L* | DNA excision repair protein ERCC-6-like | ENSG00000186871 | 2.45 | 3.38E-04 |
| *FAM83D* | Protein FAM83D | ENSG00000101447 | 2.44 | 1.08E-05 |
| *KNL1* | Kinetochore scaffold 1 | ENSG00000137812 | 2.44 | 6.45E-05 |
| *TTK* | Dual specificity protein kinase TTK | ENSG00000112742 | 2.43 | 2.68E-05 |
| *DSG2* | Desmoglein-2 | ENSG00000046604 | 2.43 | 0.05 |
| *TOP2A* | DNA topoisomerase 2-alpha | ENSG00000131747 | 2.42 | 1.11E-05 |
| *NUSAP1* | Nucleolar and spindle-associated protein 1 | ENSG00000137804 | 2.41 | 1.35E-05 |
| *HASPIN* | Serine/threonine-protein kinase haspin | ENSG00000177602 | 2.4 | 1.02E-03 |
| *KIF4A* | Chromosome-associated kinesin KIF4A | ENSG00000090889 | 2.39 | 2.11E-05 |
| *CEP55* | Centrosomal protein of 55 kDa | ENSG00000138180 | 2.39 | 1.31E-05 |
| *CDCA8* | Borealin | ENSG00000134690 | 2.37 | 4.73E-05 |
| *SPC25* | Kinetochore protein Spc25 | ENSG00000152253 | 2.37 | 1.43E-03 |
| *PIMREG* | Protein PIMREG | ENSG00000129195 | 2.35 | 2.01E-04 |
| *CDC20* | Cell division cycle protein 20 homolog | ENSG00000117399 | 2.35 | 9.60E-05 |
| *CENPU* | Centromere protein U | ENSG00000151725 | 2.34 | 1.24E-04 |
| *DEPDC1* | DEP domain-containing protein 1A | ENSG00000024526 | 2.33 | 4.73E-05 |
| *UBE2C* | Ubiquitin-conjugating enzyme E2 C | ENSG00000175063 | 2.33 | 3.66E-05 |
| *CENPF* | Centromere protein F | ENSG00000117724 | 2.32 | 1.08E-05 |
| *SKA1* | Spindle and kinetochore-associated protein 1 | ENSG00000154839 | 2.32 | 1.63E-04 |
| *CCNB2* | G2/mitotic-specific cyclin-B2 | ENSG00000157456 | 2.31 | 7.24E-05 |
| *CKAP2L* | Cytoskeleton-associated protein 2-like | ENSG00000169607 | 2.31 | 4.68E-05 |
| *DDIAS* | DNA damage-induced apoptosis suppressor protein | ENSG00000165490 | 2.3 | 2.96E-04 |
| *PBK* | Lymphokine-activated killer T-cell-originated protein kinase | ENSG00000168078 | 2.29 | 1.31E-05 |
| *DEPDC1B* | DEP domain-containing protein 1B | ENSG00000035499 | 2.28 | 0.01 |
| *CCNA2* | Cyclin-A2 | ENSG00000145386 | 2.27 | 4.80E-05 |
| *E2F2* | Transcription factor E2F2 | ENSG00000007968 | 2.27 | 0.01 |
| *KIF20A* | Kinesin-like protein KIF20A | ENSG00000112984 | 2.26 | 1.44E-04 |
| *PRC1* | Protein regulator of cytokinesis 1 | ENSG00000198901 | 2.26 | 5.15E-05 |
| *PLK4* | Serine/threonine-protein kinase PLK4 | ENSG00000142731 | 2.26 | 1.44E-04 |
| *NCAPH* | Condensin complex subunit 2 | ENSG00000121152 | 2.25 | 1.81E-04 |
| *BIRC5* | Baculoviral IAP repeat-containing protein 5 | ENSG00000089685 | 2.24 | 1.17E-04 |
| *XRCC2* | DNA repair protein XRCC2 | ENSG00000196584 | 2.24 | 3.86E-03 |
| *SAPCD2* | Suppressor APC domain-containing protein 2 | ENSG00000186193 | 2.24 | 1.33E-03 |
| *KIFC1* | Kinesin-like protein KIFC1 | ENSG00000237649 | 2.2 | 4.73E-05 |
| *CCNB1* | G2/mitotic-specific cyclin-B1 | ENSG00000134057 | 2.2 | 1.29E-04 |
| *KIF23* | Kinesin-like protein KIF23 | ENSG00000137807 | 2.19 | 2.45E-05 |
| *KRT34* | Keratin, type I cuticular Ha4 | ENSG00000131737 | 2.18 | 0.03 |
| *CDK1* | Cyclin-dependent kinase 1 | ENSG00000170312 | 2.18 | 5.33E-05 |
| *KRT19* | Keratin, type I cytoskeletal 19 | ENSG00000171345 | 2.18 | 1.25E-05 |
| *POLQ* | DNA polymerase theta | ENSG00000051341 | 2.17 | 5.44E-04 |
| *AURKA* | Aurora kinase A | ENSG00000087586 | 2.17 | 3.37E-05 |
| *SHCBP1* | SHC SH2 domain-binding protein 1 | ENSG00000171241 | 2.16 | 1.44E-04 |
| *CENPA* | Histone H3-like centromeric protein A | ENSG00000115163 | 2.16 | 1.43E-03 |
| *ESPL1* | Separin | ENSG00000135476 | 2.16 | 1.96E-04 |
| *SPAG5* | Sperm-associated antigen 5 | ENSG00000076382 | 2.16 | 1.44E-04 |
| *KIF2C* | Kinesin-like protein KIF2C | ENSG00000142945 | 2.15 | 3.20E-04 |
| *SGO1* | Shugoshin 1 | ENSG00000129810 | 2.15 | 7.97E-03 |
| *CDCA2* | Cell division cycle-associated protein 2 | ENSG00000184661 | 2.14 | 1.44E-04 |
| *AURKB* | Aurora kinase B | ENSG00000178999 | 2.14 | 1.25E-03 |
| *BLM* | Bloom syndrome protein | ENSG00000197299 | 2.13 | 7.91E-03 |
| *BDKRB1* | B1 bradykinin receptor | ENSG00000100739 | 2.13 | 1.86E-03 |
| *SKA3* | Spindle and kinetochore-associated protein 3 | ENSG00000165480 | 2.12 | 1.19E-03 |
| *SPC24* | Kinetochore protein Spc24 | ENSG00000161888 | 2.11 | 9.95E-04 |
| *CDCA3* | Cell division cycle-associated protein 3 | ENSG00000111665 | 2.11 | 8.73E-04 |
| *NCAPG* | Condensin complex subunit 3 | ENSG00000109805 | 2.11 | 5.33E-05 |
| *PRR11* | Proline-rich protein 11 | ENSG00000068489 | 2.1 | 6.16E-04 |
| *KIF18A* | Kinesin-like protein KIF18A | ENSG00000121621 | 2.1 | 1.24E-04 |
| *SYNPO2* | Synaptopodin-2 | ENSG00000172403 | 2.09 | 0.01 |
| *ERFE* | Erythroferrone | ENSG00000178752 | 2.09 | 0.01 |
| *CENPE* | Centromere-associated protein E | ENSG00000138778 | 2.07 | 8.09E-05 |
| *GBP3* | Guanylate-binding protein 3 | ENSG00000117226 | 2.07 | 0.02 |
| *FAM72D* | Protein FAM72D | ENSG00000215784 | 2.06 | 0.04 |
| *CIT* | Citron Rho-interacting kinase | ENSG00000122966 | 2.05 | 7.24E-05 |
| *NDC80* | Kinetochore protein NDC80 homolog | ENSG00000080986 | 2.04 | 2.81E-04 |
| *COMP* | Cartilage oligomeric matrix protein | ENSG00000105664 | 2.04 | 0.04 |
| *BRIP1* | Fanconi anemia group J protein | ENSG00000136492 | 2 | 7.54E-04 |
| *TPX2* | Targeting protein for Xklp2 | ENSG00000088325 | 1.99 | 4.73E-05 |
| *KIF20B* | Kinesin-like protein KIF20B | ENSG00000138182 | 1.99 | 1.23E-04 |
| *PKMYT1* | Membrane-associated tyrosine- and threonine-specific cdc2-inhibitory kinase | ENSG00000127564 | 1.99 | 5.15E-04 |
| *ESCO2* | N-acetyltransferase ESCO2 | ENSG00000171320 | 1.98 | 5.64E-03 |
| *WDR62* | WD repeat-containing protein 62 | ENSG00000075702 | 1.98 | 5.82E-04 |
| *RAD51AP1* | RAD51-associated protein 1 | ENSG00000111247 | 1.95 | 7.81E-04 |
| *RAD54L* | DNA repair and recombination protein RAD54-like | ENSG00000085999 | 1.94 | 4.56E-03 |
| *FANCD2* | Fanconi anemia group D2 protein | ENSG00000144554 | 1.94 | 2.01E-03 |
| *KIF11* | Kinesin-like protein KIF11 | ENSG00000138160 | 1.94 | 1.41E-04 |
| *HMMR* | Hyaluronan mediated motility receptor | ENSG00000072571 | 1.93 | 5.32E-04 |
| *TACC3* | Transforming acidic coiled-coil-containing protein 3 | ENSG00000013810 | 1.92 | 4.77E-04 |
| *TRIP13* | Pachytene checkpoint protein 2 homolog | ENSG00000071539 | 1.92 | 9.35E-04 |
| *CDT1* | DNA replication factor Cdt1 | ENSG00000167513 | 1.92 | 1.22E-04 |
| *APCDD1L* | Protein APCDD1-like | ENSG00000198768 | 1.91 | 1.31E-05 |
| *KIF15* | Kinesin-like protein KIF15 | ENSG00000163808 | 1.9 | 0.01 |
| *LRRC2* | Leucine-rich repeat-containing protein 2 | ENSG00000163827 | 1.88 | 1.09E-03 |
| *LAMC2* | Laminin subunit gamma-2 | ENSG00000058085 | 1.88 | 4.28E-03 |
| *CCKAR* | Cholecystokinin receptor type A | ENSG00000163394 | 1.87 | 0.02 |
| *BRINP1* | BMP/retinoic acid-inducible neural-specific protein 1 | ENSG00000078725 | 1.86 | 0.03 |
| *NPAS1* | Neuronal PAS domain-containing protein 1 | ENSG00000130751 | 1.84 | 0.02 |
| *RACGAP1* | Rac GTPase-activating protein 1 | ENSG00000161800 | 1.83 | 9.60E-05 |
| *HSPB3* | Heat shock protein beta-3 | ENSG00000169271 | 1.83 | 7.48E-03 |
| *NEURL1B* | E3 ubiquitin-protein ligase NEURL1B | ENSG00000214357 | 1.82 | 0.03 |
| *PTTG1* | Securin | ENSG00000164611 | 1.81 | 3.44E-04 |
| *LCE2A* | Late cornified envelope protein 2A | ENSG00000187173 | 1.8 | 0.04 |
| *PLCXD3* | PI-PLC X domain-containing protein 3 | ENSG00000182836 | 1.8 | 5.70E-03 |
| *PSRC1* | Proline/serine-rich coiled-coil protein 1 | ENSG00000134222 | 1.8 | 3.86E-03 |
| *CDCA5* | Sororin | ENSG00000146670 | 1.79 | 1.70E-03 |
| *CLSPN* | Claspin | ENSG00000092853 | 1.79 | 1.49E-03 |
| *BARD1* | BRCA1-associated RING domain protein 1 | ENSG00000138376 | 1.78 | 4.66E-03 |
| *ENPP2* | Ectonucleotide pyrophosphatase/phosphodiesterase family member 2 | ENSG00000136960 | 1.78 | 0.02 |
| *CENPK* | Centromere protein K | ENSG00000123219 | 1.78 | 2.92E-03 |
| *MYBL2* | Myb-related protein B | ENSG00000101057 | 1.78 | 5.25E-04 |
| *HMGA2* | High mobility group protein HMGI-C | ENSG00000149948 | 1.78 | 1.56E-09 |
| *MAD2L1* | Mitotic spindle assembly checkpoint protein MAD2A | ENSG00000164109 | 1.77 | 7.35E-04 |
| *KIF24* | Kinesin-like protein KIF24 | ENSG00000186638 | 1.77 | 0.02 |
| *GINS2* | DNA replication complex GINS protein PSF2 | ENSG00000131153 | 1.75 | 9.68E-03 |
| *SERPINB2* | Plasminogen activator inhibitor 2 | ENSG00000197632 | 1.75 | 2.00E-05 |
| *ZNF367* | Zinc finger protein 367 | ENSG00000165244 | 1.74 | 6.16E-04 |
| *MALL* | MAL-like protein | ENSG00000144063 | 1.74 | 6.16E-04 |
| *SGO2* | Shugoshin 2 | ENSG00000163535 | 1.73 | 1.40E-03 |
| *ARHGAP11A* | Rho GTPase-activating protein 11A | ENSG00000198826 | 1.71 | 5.37E-04 |
| *FAM111B* | Serine protease FAM111B | ENSG00000189057 | 1.71 | 7.94E-03 |
| *FANCI* | Fanconi anemia group I protein | ENSG00000140525 | 1.7 | 4.85E-04 |
| *CIP2A* | Protein CIP2A | ENSG00000163507 | 1.68 | 1.85E-03 |
| *KRT18* | Keratin, type I cytoskeletal 18 | ENSG00000111057 | 1.68 | 0.03 |
| *EXO1* | Exonuclease 1 | ENSG00000174371 | 1.68 | 0.01 |
| *ATAD5* | ATPase family AAA domain-containing protein 5 | ENSG00000176208 | 1.67 | 0.03 |
| *RRM2* | Ribonucleoside-diphosphate reductase subunit M2 | ENSG00000171848 | 1.66 | 1.19E-03 |
| *MARCHF4* | E3 ubiquitin-protein ligase MARCHF4 | ENSG00000144583 | 1.66 | 1.09E-03 |
| *ORC1* | Origin recognition complex subunit 1 | ENSG00000085840 | 1.65 | 0.03 |
| *MCM10* | Protein MCM10 homolog | ENSG00000065328 | 1.65 | 0.02 |
| *ASF1B* | Histone chaperone ASF1B | ENSG00000105011 | 1.64 | 3.92E-03 |
| *TCF19* | Transcription factor 19 | ENSG00000137310 | 1.63 | 1.19E-03 |
| *CDKN3* | Cyclin-dependent kinase inhibitor 3 | ENSG00000100526 | 1.62 | 7.02E-03 |
| *DTL* | Denticleless protein homolog | ENSG00000143476 | 1.62 | 1.43E-03 |
| *ABI3BP* | Target of Nesh-SH3 | ENSG00000154175 | 1.61 | 1.19E-03 |
| *MELK* | Maternal embryonic leucine zipper kinase | ENSG00000165304 | 1.61 | 1.13E-03 |
| *NCAPG2* | Condensin-2 complex subunit G2 | ENSG00000146918 | 1.61 | 3.46E-03 |
| *CCNF* | Cyclin-F | ENSG00000162063 | 1.59 | 6.49E-03 |
| *MYBL1* | Myb-related protein A | ENSG00000185697 | 1.59 | 7.37E-03 |
| *PSG2* | Pregnancy-specific beta-1-glycoprotein 2 | ENSG00000242221 | 1.59 | 1.98E-03 |
| *CDC45* | Cell division control protein 45 homolog | ENSG00000093009 | 1.58 | 0.02 |
| *CENPI* | Centromere protein I | ENSG00000102384 | 1.57 | 0.02 |
| *TK1* | Thymidine kinase, cytosolic | ENSG00000167900 | 1.56 | 1.17E-03 |
| *RFC3* | Replication factor C subunit 3 | ENSG00000133119 | 1.56 | 7.48E-03 |
| *DMKN* | Dermokine | ENSG00000161249 | 1.54 | 4.54E-03 |
| *FOXM1* | Forkhead box protein M1 | ENSG00000111206 | 1.54 | 1.02E-03 |
| *GINS4* | DNA replication complex GINS protein SLD5 | ENSG00000147536 | 1.53 | 0.01 |
| *KIF22* | Kinesin-like protein KIF22 | ENSG00000079616 | 1.53 | 3.08E-03 |
| *KNSTRN* | Small kinetochore-associated protein | ENSG00000128944 | 1.53 | 1.32E-03 |
| *MCM5* | DNA replication licensing factor MCM5 | ENSG00000100297 | 1.52 | 6.16E-04 |
| *BRCA2* | Breast cancer type 2 susceptibility protein | ENSG00000139618 | 1.52 | 0.01 |
| *ZWINT* | ZW10 interactor | ENSG00000122952 | 1.51 | 3.27E-03 |
| *CENPH* | Centromere protein H | ENSG00000153044 | 1.48 | 0.04 |
| *HMGA1* | High mobility group protein HMG-I/HMG-Y | ENSG00000137309 | 1.46 | 5.70E-03 |
| *GLIPR1* | Glioma pathogenesis-related protein 1 | ENSG00000139278 | 1.46 | 0.02 |
| *CCDC18* |  | ENSG00000122483 | 1.45 | 0.02 |
| *POC1A* | POC1 centriolar protein homolog A | ENSG00000164087 | 1.45 | 0.03 |
| *CLN6* | Ceroid-lipofuscinosis neuronal protein 6 | ENSG00000128973 | 1.44 | 1.12E-05 |
| *BRCA1* | Breast cancer type 1 susceptibility protein | ENSG00000012048 | 1.42 | 5.84E-03 |
| *TRNP1* | TMF-regulated nuclear protein 1 | ENSG00000253368 | 1.42 | 0.02 |
| *TMPO* | Lamina-associated polypeptide 2, isoform alpha | ENSG00000120802 | 1.41 | 3.18E-03 |
| *STIL* | SCL-interrupting locus protein | ENSG00000123473 | 1.41 | 0.02 |
| *FOXQ1* | Forkhead box protein Q1 | ENSG00000164379 | 1.41 | 0.05 |
| *SHROOM3* | Protein Shroom3 | ENSG00000138771 | 1.41 | 7.00E-03 |
| *FBXO5* | F-box only protein 5 | ENSG00000112029 | 1.38 | 0.03 |
| *INCENP* | Inner centromere protein | ENSG00000149503 | 1.37 | 7.70E-03 |
| *RAB3B* | Ras-related protein Rab-3B | ENSG00000169213 | 1.37 | 5.84E-03 |
| *NEIL3* | Endonuclease 8-like 3 | ENSG00000109674 | 1.35 | 0.04 |
| *TNC* | Tenascin | ENSG00000041982 | 1.34 | 0.02 |
| *MT2A* | Metallothionein-2 | ENSG00000125148 | 1.33 | 0.02 |
| *TCF7* | Transcription factor 7 | ENSG00000081059 | 1.32 | 0.02 |
| *UHRF1* | E3 ubiquitin-protein ligase UHRF1 | ENSG00000276043 | 1.32 | 9.66E-03 |
| *SPOCD1* | SPOC domain-containing protein 1 | ENSG00000134668 | 1.31 | 0.02 |
| *CKAP2* | Cytoskeleton-associated protein 2 | ENSG00000136108 | 1.31 | 5.70E-03 |
| *KNTC1* | Kinetochore-associated protein 1 | ENSG00000184445 | 1.3 | 8.44E-03 |
| *ARHGAP19* | Rho GTPase-activating protein 19 | ENSG00000213390 | 1.29 | 0.04 |
| *ATAD2* | ATPase family AAA domain-containing protein 2 | ENSG00000156802 | 1.27 | 7.91E-03 |
| *FANCA* | Fanconi anemia group A protein | ENSG00000187741 | 1.27 | 0.02 |
| *CHAF1B* | Chromatin assembly factor 1 subunit B | ENSG00000159259 | 1.27 | 0.04 |
| *DBF4* | Protein DBF4 homolog A | ENSG00000006634 | 1.26 | 9.68E-03 |
| *KIAA1549L* | UPF0606 protein KIAA1549L | ENSG00000110427 | 1.25 | 0.02 |
| *ECT2* | Protein ECT2 | ENSG00000114346 | 1.22 | 0.01 |
| *CDC6* | Cell division control protein 6 homolog | ENSG00000094804 | 1.2 | 0.03 |
| *WDHD1* | WD repeat and HMG-box DNA-binding protein 1 | ENSG00000198554 | 1.2 | 7.03E-03 |
| *HELLS* | Lymphoid-specific helicase | ENSG00000119969 | 1.19 | 0.04 |
| *EZR* | Ezrin | ENSG00000092820 | 1.16 | 2.15E-04 |
| *POLE* | DNA polymerase epsilon catalytic subunit A | ENSG00000177084 | 1.16 | 3.14E-03 |
| *MICAL2* | [F-actin]-monooxygenase MICAL2 | ENSG00000133816 | 1.16 | 0.01 |
| *LIG1* | DNA ligase 1 | ENSG00000105486 | 1.15 | 0.02 |
| *CNTRL* | Centriolin | ENSG00000119397 | 1.15 | 0.03 |
| *CKS1B* | Cyclin-dependent kinases regulatory subunit 1 | ENSG00000173207 | 1.14 | 0.03 |
| *MCM4* | DNA replication licensing factor MCM4 | ENSG00000104738 | 1.14 | 7.24E-03 |
| *CHAF1A* | Chromatin assembly factor 1 subunit A | ENSG00000167670 | 1.13 | 0.01 |
| *CENPN* | Centromere protein N | ENSG00000166451 | 1.12 | 0.02 |
| *TIMELESS* | Protein timeless homolog | ENSG00000111602 | 1.11 | 0.03 |
| *DNAJC9* | DnaJ homolog subfamily C member 9 | ENSG00000213551 | 1.11 | 0.04 |
| *MASTL* | Serine/threonine-protein kinase greatwall | ENSG00000120539 | 1.09 | 0.03 |
| *KRTAP1-5* | Keratin-associated protein 1-5 | ENSG00000221852 | 1.09 | 0.01 |
| *RFWD3* | E3 ubiquitin-protein ligase RFWD3 | ENSG00000168411 | 1.08 | 0.01 |
| *SMC4* | Structural maintenance of chromosomes protein 4 | ENSG00000113810 | 1.07 | 0.03 |
| *NCAPD2* | Condensin complex subunit 1 | ENSG00000010292 | 1.06 | 0.03 |
| *FEN1* | Flap endonuclease 1 | ENSG00000168496 | 1.04 | 0.02 |
| *TYMS* | Thymidylate synthase | ENSG00000176890 | 1.04 | 0.03 |
| *GAS2L3* | GAS2-like protein 3 | ENSG00000139354 | 1.04 | 0.05 |
| *MCM3* | DNA replication licensing factor MCM3 | ENSG00000112118 | 1.02 | 0.02 |
| *MCM7* | DNA replication licensing factor MCM7 | ENSG00000166508 | 1 | 0.01 |
