# Supplementary material for: EMILIN-1 Suppresses Cell Proliferation through Altered Cell Cycle Regulation in Head and Neck Squamous Cell Carcinoma
Source: Am J Pathol. 2025 Jan 30;195(5):995–1012. doi: 10.1016/j.ajpath.2025.01.010 (PMC12163418; doi:10.1016/j.ajpath.2025.01.010)
Supplement: Supplemental Table S8 [file mmc8.docx]

| **Supplemental Table S8**. Downregulated genes of NF2 cell with EMILIN-1 knockdown (Log2FC<-1,FDR<0.05). (https://www.ensembl.org) | | | |  |
| --- | --- | --- | --- | --- |
|  |  |  |  |  |
| **Gene** | **Database name** | **Identifier** | **Log2FC** | **FDR p-value** |
| *PF4V1* | Platelet factor 4 variant | ENSG00000109272 | -4.39 | 2.97E-04 |
| *EMILIN1* | Elastin microfibril interfacer 1 | ENSG00000138080 | -4.14 | 9.87E-30 |
| *C6* | Complement component C6 | ENSG00000039537 | -4.11 | 0.02 |
| *JAKMIP2* | Janus kinase and microtubule-interacting protein 2 | ENSG00000176049 | -3.85 | 0.01 |
| *PDZRN4* | PDZ domain-containing RING finger protein 4 | ENSG00000165966 | -3.76 | 1.44E-04 |
| *COL15A1* | Collagen alpha-1(XV) chain | ENSG00000204291 | -3.75 | 1.14E-12 |
| *SCRG1* | Scrapie-responsive protein 1 | ENSG00000164106 | -3.69 | 1.12E-09 |
| *SLITRK6* | SLIT and NTRK-like protein 6 | ENSG00000184564 | -3.58 | 1.91E-03 |
| *P2RY14* | P2Y purinoceptor 14 | ENSG00000174944 | -3.51 | 3.39E-03 |
| *CXCL6* | C-X-C motif chemokine 6 | ENSG00000124875 | -3.05 | 7.14E-11 |
| *ITIH3* | Inter-alpha-trypsin inhibitor heavy chain H3 | ENSG00000162267 | -2.95 | 7.24E-03 |
| *FAIM2* | Protein lifeguard 2 | ENSG00000135472 | -2.79 | 2.91E-11 |
| *GCK* | Hexokinase-4 | ENSG00000106633 | -2.63 | 1.68E-03 |
| *RAB39B* | Ras-related protein Rab-39B | ENSG00000155961 | -2.48 | 2.24E-04 |
| *OMD* | Osteomodulin | ENSG00000127083 | -2.44 | 1.05E-05 |
| *C7* | Complement component C7 | ENSG00000112936 | -2.4 | 0.05 |
| *SERPINA3* | Alpha-1-antichymotrypsin | ENSG00000196136 | -2.35 | 4.05E-04 |
| *CXCL1* | Growth-regulated alpha protein | ENSG00000163739 | -2.33 | 4.09E-06 |
| *FRZB* | Secreted frizzled-related protein 3 | ENSG00000162998 | -2.32 | 1.85E-03 |
| *HSD17B6* | 17-beta-hydroxysteroid dehydrogenase type 6 | ENSG00000025423 | -2.16 | 0.04 |
| *SLPI* | Antileukoproteinase | ENSG00000124107 | -2.11 | 0.04 |
| *ALDH3A1* | Aldehyde dehydrogenase, dimeric NADP-preferring | ENSG00000108602 | -2.1 | 0.01 |
| *CEND1* | Cell cycle exit and neuronal differentiation protein 1 | ENSG00000184524 | -2.1 | 4.73E-05 |
| *MAP2K6* | Dual specificity mitogen-activated protein kinase kinase 6 | ENSG00000108984 | -1.89 | 5.70E-03 |
| *COL14A1* | Collagen alpha-1(XIV) chain | ENSG00000187955 | -1.88 | 1.27E-06 |
| *DKK2* | Dickkopf-related protein 2 | ENSG00000155011 | -1.87 | 5.30E-03 |
| *INMT* | Indolethylamine N-methyltransferase | ENSG00000241644 | -1.81 | 3.66E-05 |
| *MMP27* | Matrix metalloproteinase-27 | ENSG00000137675 | -1.78 | 0.03 |
| *CLSTN2* | Calsyntenin-2 | ENSG00000158258 | -1.78 | 5.70E-03 |
| *SEMA3B* | Semaphorin-3B | ENSG00000012171 | -1.77 | 1.84E-05 |
| *SFRP2* | Secreted frizzled-related protein 2 | ENSG00000145423 | -1.77 | 0.01 |
| *TSPAN12* | Tetraspanin-12 | ENSG00000106025 | -1.77 | 0.02 |
| *GXYLT2* | Glucoside xylosyltransferase 2 | ENSG00000172986 | -1.69 | 2.34E-06 |
| *PRR5L* | Proline-rich protein 5-like | ENSG00000135362 | -1.68 | 0.05 |
| *TPPP3* | Tubulin polymerization-promoting protein family member 3 | ENSG00000159713 | -1.66 | 0.02 |
| *KCNT2* | Potassium channel subfamily T member 2 | ENSG00000162687 | -1.66 | 0.03 |
| *GALNT16* | Polypeptide N-acetylgalactosaminyltransferase 16 | ENSG00000100626 | -1.59 | 5.33E-05 |
| *ADAMTS8* | A disintegrin and metalloproteinase with thrombospondin motifs 8 | ENSG00000134917 | -1.52 | 0.03 |
| *NXPH4* | Neurexophilin-4 | ENSG00000182379 | -1.44 | 0.03 |
| *TNFSF13B* | Tumor necrosis factor ligand superfamily member 13B | ENSG00000102524 | -1.43 | 2.05E-03 |
| *SEMA3F* | Semaphorin-3F | ENSG00000001617 | -1.43 | 0.01 |
| *TMOD1* | Tropomodulin-1 | ENSG00000136842 | -1.42 | 0.02 |
| *JAM2* | Junctional adhesion molecule B | ENSG00000154721 | -1.42 | 0.02 |
| *GAS1* | Growth arrest-specific protein 1 | ENSG00000180447 | -1.41 | 7.75E-03 |
| *CDKN2B* | Cyclin-dependent kinase 4 inhibitor B | ENSG00000147883 | -1.39 | 4.74E-03 |
| *CHAC1* | Glutathione-specific gamma-glutamylcyclotransferase 1 | ENSG00000128965 | -1.38 | 0.04 |
| *BMP4* | Bone morphogenetic protein 4 | ENSG00000125378 | -1.38 | 9.15E-05 |
| *IL17D* | Interleukin-17D | ENSG00000172458 | -1.37 | 0.05 |
| *ATP8B4* | Probable phospholipid-transporting ATPase IM | ENSG00000104043 | -1.37 | 5.34E-03 |
| *ASPN* | Asporin | ENSG00000106819 | -1.36 | 0.01 |
| *ITIH5* | Inter-alpha-trypsin inhibitor heavy chain H5 | ENSG00000123243 | -1.35 | 0.03 |
| *SLC2A12* | Solute carrier family 2, facilitated glucose transporter member 12 | ENSG00000146411 | -1.33 | 0.01 |
| *TMTC2* | Protein O-mannosyl-transferase TMTC2 | ENSG00000179104 | -1.32 | 0.01 |
| *PSAT1* | Phosphoserine aminotransferase | ENSG00000135069 | -1.31 | 8.93E-03 |
| *COL3A1* | Collagen alpha-1(III) chain | ENSG00000168542 | -1.31 | 0.02 |
| *SLC6A9* | Sodium- and chloride-dependent glycine transporter 1 | ENSG00000196517 | -1.3 | 1.49E-03 |
| *SLC8B1* | Mitochondrial sodium/calcium exchanger protein | ENSG00000089060 | -1.29 | 5.23E-03 |
| *CRABP2* | Cellular retinoic acid-binding protein 2 | ENSG00000143320 | -1.29 | 9.99E-03 |
| *WNT5A* | Protein Wnt-5a | ENSG00000114251 | -1.29 | 0.02 |
| *OLFML1* | Olfactomedin-like protein 1 | ENSG00000183801 | -1.28 | 6.97E-03 |
| *PRELP* | Prolargin | ENSG00000188783 | -1.27 | 7.32E-03 |
| *COL4A5* | Collagen alpha-5(IV) chain | ENSG00000188153 | -1.27 | 0.04 |
| *ENPP1* | Ectonucleotide pyrophosphatase/phosphodiesterase family member 1 | ENSG00000197594 | -1.27 | 2.92E-03 |
| *PCSK5* | Proprotein convertase subtilisin/kexin type 5 | ENSG00000099139 | -1.26 | 3.67E-03 |
| *SAMD5* | Sterile alpha motif domain-containing protein 5 | ENSG00000203727 | -1.24 | 3.82E-03 |
| *ADAMTS5* | A disintegrin and metalloproteinase with thrombospondin motifs 5 | ENSG00000154736 | -1.24 | 0.05 |
| *ITGA11* | Integrin alpha-11 | ENSG00000137809 | -1.22 | 3.89E-05 |
| *ADM2* | Protein ADM2 | ENSG00000128165 | -1.22 | 7.00E-03 |
| *CHST2* | Carbohydrate sulfotransferase 2 | ENSG00000175040 | -1.2 | 4.15E-03 |
| *CCN4* | CCN family member 4 | ENSG00000104415 | -1.18 | 0.03 |
| *SSTR1* | Somatostatin receptor type 1 | ENSG00000139874 | -1.17 | 3.94E-04 |
| *REEP2* | Receptor expression-enhancing protein 2 | ENSG00000132563 | -1.15 | 3.38E-03 |
| *LAMA5* | Laminin subunit alpha-5 | ENSG00000130702 | -1.14 | 0.02 |
| *CD82* | CD82 antigen | ENSG00000085117 | -1.11 | 0.05 |
| *TOMM20* | Mitochondrial import receptor subunit TOM20 homolog | ENSG00000173726 | -1.1 | 2.05E-03 |
| *NAP1L5* | Nucleosome assembly protein 1-like 5 | ENSG00000177432 | -1.09 | 0.04 |
| *FAM3C* | Protein FAM3C | ENSG00000196937 | -1.07 | 0.02 |
| *SHC3* | SHC-transforming protein 3 | ENSG00000148082 | -1.06 | 0.04 |
| *APOD* | Apolipoprotein D | ENSG00000189058 | -1.04 | 0.02 |
| *C1QTNF6* | Complement C1q tumor necrosis factor-related protein 6 | ENSG00000133466 | -1.04 | 5.91E-03 |
| *TNFAIP6* | Tumor necrosis factor-inducible gene 6 protein | ENSG00000123610 | -1.03 | 0.01 |
