# Supplementary material for: EMILIN-1 Suppresses Cell Proliferation through Altered Cell Cycle Regulation in Head and Neck Squamous Cell Carcinoma
Source: Am J Pathol. 2025 Jan 30;195(5):995–1012. doi: 10.1016/j.ajpath.2025.01.010 (PMC12163418; doi:10.1016/j.ajpath.2025.01.010)
Supplement: Supplemental Table S9 [file mmc9.docx]

| **Supplemental Table S9** Upregulated genes of CAF3 cell with EMILIN-1 knockdown (Log2FC>1,FDR<0.05).  (https://www.ensembl.org) | | | | |
| --- | --- | --- | --- | --- |
|  |  |  |  |  |
| **Gene** | **Database name** | **Identifier** | **Log2FC** | **FDR p-value** |
| *EPGN* | Epigen | ENSG00000182585 | 5.36 | 1.33E-06 |
| *ACTC1* | Actin, alpha cardiac muscle 1 | ENSG00000159251 | 5.26 | 4.24E-08 |
| *KISS1* | Metastasis-suppressor KiSS-1 | ENSG00000170498 | 4.9 | 5.70E-05 |
| *DLX3* | Homeobox protein DLX-3 | ENSG00000064195 | 4.61 | 0.02 |
| *RPTN* | Repetin | ENSG00000215853 | 4.6 | 9.01E-03 |
| *CHMP4C* | Charged multivesicular body protein 4c | ENSG00000164695 | 4.38 | 0.04 |
| *NCF2* | Neutrophil cytosol factor 2 | ENSG00000116701 | 3.59 | 0.02 |
| *MDFI* | MyoD family inhibitor | ENSG00000112559 | 3.54 | 0.02 |
| *CSF2RB* | Cytokine receptor common subunit beta | ENSG00000100368 | 3.54 | 6.75E-05 |
| *VANGL2* | Vang-like protein 2 | ENSG00000162738 | 3.53 | 0.04 |
| *PCDH17* | Protocadherin-17 | ENSG00000118946 | 3.51 | 2.83E-04 |
| *RASD2* | GTP-binding protein Rhes | ENSG00000100302 | 3.15 | 3.73E-04 |
| *GAL* | Galanin peptides | ENSG00000069482 | 2.91 | 0.02 |
| *CHL1* | Neural cell adhesion molecule L1-like protein | ENSG00000134121 | 2.91 | 1.46E-04 |
| *CNGA3* | Cyclic nucleotide-gated cation channel alpha-3 | ENSG00000144191 | 2.83 | 0.01 |
| *SERPINB2* | Plasminogen activator inhibitor 2 | ENSG00000197632 | 2.68 | 9.72E-04 |
| *ADAMTS18* | A disintegrin and metalloproteinase with thrombospondin motifs 18 | ENSG00000140873 | 2.55 | 8.03E-03 |
| *TRIM55* | Tripartite motif-containing protein 55 | ENSG00000147573 | 2.48 | 0.01 |
| *FGFR3* | Fibroblast growth factor receptor 3 | ENSG00000068078 | 2.47 | 0.01 |
| *LDLRAD4* | Low-density lipoprotein receptor class A domain-containing protein 4 | ENSG00000168675 | 2.42 | 0.05 |
| *ADRA2C* | Alpha-2C adrenergic receptor | ENSG00000184160 | 2.4 | 1.74E-03 |
| *PCDH19* | Protocadherin-19 | ENSG00000165194 | 2.39 | 0.03 |
| *CTNND2* | Catenin delta-2 | ENSG00000169862 | 2.35 | 3.93E-03 |
| *FGD5* | FYVE, RhoGEF and PH domain-containing protein 5 | ENSG00000154783 | 2.29 | 7.14E-03 |
| *KRTAP2-3* | Keratin-associated protein 2-3 | ENSG00000212724 | 2.09 | 1.83E-03 |
| *ESM1* | Endothelial cell-specific molecule 1 | ENSG00000164283 | 2.08 | 3.98E-03 |
| *RUNDC3A* | RUN domain-containing protein 3A | ENSG00000108309 | 2.08 | 0.02 |
| *MYOCD* | Myocardin | ENSG00000141052 | 2.04 | 0.01 |
| *CHODL* | Chondrolectin | ENSG00000154645 | 2.01 | 1.08E-04 |
| *SAMD11* | Sterile alpha motif domain-containing protein 11 | ENSG00000187634 | 2 | 1.30E-03 |
| *PODXL* | Podocalyxin | ENSG00000128567 | 1.99 | 3.73E-04 |
| *KRTAP1-5* | Keratin-associated protein 1-5 | ENSG00000221852 | 1.97 | 2.51E-04 |
| *EPHB1* | Ephrin type-B receptor 1 | ENSG00000154928 | 1.91 | 0.01 |
| *UNC13A* | Protein unc-13 homolog A | ENSG00000130477 | 1.89 | 6.40E-05 |
| *NPAS1* | Neuronal PAS domain-containing protein 1 | ENSG00000130751 | 1.88 | 6.21E-04 |
| *IGFBPL1* | Insulin-like growth factor-binding protein-like 1 | ENSG00000137142 | 1.86 | 0.03 |
| *TMEM179* | Transmembrane protein 179 | ENSG00000258986 | 1.84 | 0.04 |
| *PLCB4* | 1-phosphatidylinositol 4,5-bisphosphate phosphodiesterase beta-4 | ENSG00000101333 | 1.83 | 1.09E-05 |
| *KRT34* | Keratin, type I cuticular Ha4 | ENSG00000131737 | 1.81 | 0.03 |
| *NOG* | Noggin | ENSG00000183691 | 1.79 | 0.02 |
| *ABCA13* | ATP-binding cassette sub-family A member 13 | ENSG00000179869 | 1.76 | 0.01 |
| *SCN5A* | Sodium channel protein type 5 subunit alpha | ENSG00000183873 | 1.76 | 0.04 |
| *CARMIL2* | Capping protein, Arp2/3 and myosin-I linker protein 2 | ENSG00000159753 | 1.75 | 0.03 |
| *ANO3* | Anoctamin-3 | ENSG00000134343 | 1.74 | 3.43E-03 |
| *GPR137C* | Integral membrane protein GPR137C | ENSG00000180998 | 1.74 | 0.03 |
| *ALDH3A1* | Aldehyde dehydrogenase, dimeric NADP-preferring | ENSG00000108602 | 1.72 | 0.04 |
| *MYPN* | Myopalladin | ENSG00000138347 | 1.72 | 0.04 |
| *HSPB7* | Heat shock protein beta-7 | ENSG00000173641 | 1.71 | 6.40E-05 |
| *OGDHL* | 2-oxoglutarate dehydrogenase-like, mitochondrial | ENSG00000197444 | 1.68 | 0.02 |
| *KRT7* | Keratin, type II cytoskeletal 7 | ENSG00000135480 | 1.65 | 3.67E-03 |
| *HAS2* | Hyaluronan synthase 2 | ENSG00000170961 | 1.62 | 1.32E-03 |
| *SULT1B1* | Sulfotransferase 1B1 | ENSG00000173597 | 1.61 | 0.04 |
| *COMP* | Cartilage oligomeric matrix protein | ENSG00000105664 | 1.61 | 0.05 |
| *EDN1* | Endothelin-1 | ENSG00000078401 | 1.61 | 2.97E-03 |
| *ALS2CL* | ALS2 C-terminal-like protein | ENSG00000178038 | 1.58 | 0.01 |
| *FAM83H* | Protein FAM83H | ENSG00000180921 | 1.57 | 2.83E-04 |
| *AKAP6* | A-kinase anchor protein 6 | ENSG00000151320 | 1.56 | 6.75E-05 |
| *ATOH8* | Protein atonal homolog 8 | ENSG00000168874 | 1.55 | 2.02E-04 |
| *TCF21* | Transcription factor 21 | ENSG00000118526 | 1.55 | 6.40E-05 |
| *PTPRO* | Receptor-type tyrosine-protein phosphatase O | ENSG00000151490 | 1.51 | 0.05 |
| *SLC6A17* | Sodium-dependent neutral amino acid transporter SLC6A17 | ENSG00000197106 | 1.47 | 7.14E-03 |
| *DEPDC1B* | DEP domain-containing protein 1B | ENSG00000035499 | 1.46 | 0.01 |
| *CCDC81* | Coiled-coil domain-containing protein 81 | ENSG00000149201 | 1.45 | 0.04 |
| *SKA1* | Spindle and kinetochore-associated protein 1 | ENSG00000154839 | 1.44 | 0.01 |
| *HJURP* | Holliday junction recognition protein | ENSG00000123485 | 1.44 | 0.01 |
| *ZNF185* | Zinc finger protein 185 | ENSG00000147394 | 1.44 | 6.01E-03 |
| *NEK2* | Serine/threonine-protein kinase Nek2 | ENSG00000117650 | 1.43 | 0.02 |
| *ANLN* | Anillin | ENSG00000011426 | 1.42 | 0.01 |
| *NCEH1* | Neutral cholesterol ester hydrolase 1 | ENSG00000144959 | 1.42 | 1.06E-03 |
| *KIAA0040* | Uncharacterized protein KIAA0040 | ENSG00000235750 | 1.41 | 0.05 |
| *GTSE1* | G2 and S phase-expressed protein 1 | ENSG00000075218 | 1.41 | 9.99E-03 |
| *FOXQ1* | Forkhead box protein Q1 | ENSG00000164379 | 1.4 | 5.44E-03 |
| *DEPDC1* | DEP domain-containing protein 1A | ENSG00000024526 | 1.4 | 0.02 |
| *CDCA2* | Cell division cycle-associated protein 2 | ENSG00000184661 | 1.39 | 8.76E-03 |
| *ADRB2* | Beta-2 adrenergic receptor | ENSG00000169252 | 1.38 | 0.04 |
| *KNL1* | Kinetochore scaffold 1 | ENSG00000137812 | 1.37 | 0.02 |
| *P2RX5* | P2X purinoceptor 5 | ENSG00000083454 | 1.36 | 0.03 |
| *CDCA8* | Borealin | ENSG00000134690 | 1.35 | 0.02 |
| *ASPM* | Abnormal spindle-like microcephaly-associated protein | ENSG00000066279 | 1.35 | 0.01 |
| *HSD11B1* | Corticosteroid 11-beta-dehydrogenase isozyme 1 | ENSG00000117594 | 1.35 | 0.04 |
| *ANOS1* | Anosmin-1 | ENSG00000011201 | 1.35 | 0.01 |
| *CCNA2* | Cyclin-A2 | ENSG00000145386 | 1.34 | 0.01 |
| *LAMC2* | Laminin subunit gamma-2 | ENSG00000058085 | 1.33 | 3.02E-03 |
| *POSTN* | Periostin | ENSG00000133110 | 1.33 | 1.12E-04 |
| *DLGAP5* | Disks large-associated protein 5 | ENSG00000126787 | 1.32 | 0.02 |
| *KIF23* | Kinesin-like protein KIF23 | ENSG00000137807 | 1.32 | 4.75E-03 |
| *PLPP4* | Phospholipid phosphatase 4 | ENSG00000203805 | 1.31 | 0.05 |
| *CENPE* | Centromere-associated protein E | ENSG00000138778 | 1.31 | 0.02 |
| *TNC* | Tenascin | ENSG00000041982 | 1.3 | 0.02 |
| *PLCXD3* | PI-PLC X domain-containing protein 3 | ENSG00000182836 | 1.3 | 2.88E-03 |
| *MCAM* | Cell surface glycoprotein MUC18 | ENSG00000076706 | 1.3 | 0.03 |
| *AURKA* | Aurora kinase A | ENSG00000087586 | 1.29 | 0.01 |
| *CCNB2* | G2/mitotic-specific cyclin-B2 | ENSG00000157456 | 1.28 | 0.01 |
| *PLK1* | Serine/threonine-protein kinase PLK1 | ENSG00000166851 | 1.28 | 0.03 |
| *PIMREG* | Protein PIMREG | ENSG00000129195 | 1.28 | 0.02 |
| *PDE1C* | Calcium/calmodulin-dependent 3',5'-cyclic nucleotide phosphodiesterase 1C | ENSG00000154678 | 1.27 | 4.01E-03 |
| *PBK* | Lymphokine-activated killer T-cell-originated protein kinase | ENSG00000168078 | 1.27 | 0.02 |
| *MYCBP* | c-Myc-binding protein | ENSG00000214114 | 1.26 | 1.95E-03 |
| *DKK1* | Dickkopf-related protein 1 | ENSG00000107984 | 1.26 | 9.06E-03 |
| *NDC80* | Kinetochore protein NDC80 homolog | ENSG00000080986 | 1.26 | 0.02 |
| *SPAG5* | Sperm-associated antigen 5 | ENSG00000076382 | 1.26 | 0.02 |
| *CXCL14* | C-X-C motif chemokine 14 | ENSG00000145824 | 1.26 | 0.04 |
| *CEP55* | Centrosomal protein of 55 kDa | ENSG00000138180 | 1.26 | 0.02 |
| *E2F7* | Transcription factor E2F7 | ENSG00000165891 | 1.26 | 7.47E-03 |
| *KIF18A* | Kinesin-like protein KIF18A | ENSG00000121621 | 1.26 | 0.04 |
| *CKAP2L* | Cytoskeleton-associated protein 2-like | ENSG00000169607 | 1.26 | 0.04 |
| *HMMR* | Hyaluronan mediated motility receptor | ENSG00000072571 | 1.26 | 0.04 |
| *FAM83D* | Protein FAM83D | ENSG00000101447 | 1.25 | 0.02 |
| *BUB1* | Mitotic checkpoint serine/threonine-protein kinase BUB1 | ENSG00000169679 | 1.25 | 0.02 |
| *SEMA3E* | Semaphorin-3E | ENSG00000170381 | 1.25 | 3.96E-03 |
| *TOP2A* | DNA topoisomerase 2-alpha | ENSG00000131747 | 1.24 | 0.04 |
| *MYBL1* | Myb-related protein A | ENSG00000185697 | 1.24 | 0.03 |
| *TSPAN13* | Tetraspanin-13 | ENSG00000106537 | 1.24 | 0.01 |
| *MT1X* | Metallothionein-1X | ENSG00000187193 | 1.24 | 9.01E-03 |
| *GLIPR1* | Glioma pathogenesis-related protein 1 | ENSG00000139278 | 1.22 | 1.60E-04 |
| *TRNP1* | TMF-regulated nuclear protein 1 | ENSG00000253368 | 1.22 | 0.01 |
| *DIAPH3* | Protein diaphanous homolog 3 | ENSG00000139734 | 1.22 | 4.21E-03 |
| *PRC1* | Protein regulator of cytokinesis 1 | ENSG00000198901 | 1.21 | 0.02 |
| *PRR11* | Proline-rich protein 11 | ENSG00000068489 | 1.21 | 0.02 |
| *MMP24* | Matrix metalloproteinase-24 | ENSG00000125966 | 1.2 | 0.04 |
| *NUF2* | Kinetochore protein Nuf2 | ENSG00000143228 | 1.2 | 0.03 |
| *EEF1A2* | Elongation factor 1-alpha 2 | ENSG00000101210 | 1.2 | 0.02 |
| *IL7R* | Interleukin-7 receptor subunit alpha | ENSG00000168685 | 1.2 | 0.01 |
| *HAS3* | Hyaluronan synthase 3 | ENSG00000103044 | 1.2 | 0.01 |
| *PSRC1* | Proline/serine-rich coiled-coil protein 1 | ENSG00000134222 | 1.2 | 0.02 |
| *ATAD5* | ATPase family AAA domain-containing protein 5 | ENSG00000176208 | 1.2 | 0.04 |
| *KIF4A* | Chromosome-associated kinesin KIF4A | ENSG00000090889 | 1.2 | 0.04 |
| *SHCBP1* | SHC SH2 domain-binding protein 1 | ENSG00000171241 | 1.19 | 0.04 |
| *SPDL1* | Protein Spindly | ENSG00000040275 | 1.19 | 6.34E-03 |
| *CDC20* | Cell division cycle protein 20 homolog | ENSG00000117399 | 1.18 | 0.04 |
| *KIF18B* | Kinesin-like protein KIF18B | ENSG00000186185 | 1.18 | 0.05 |
| *KCNB1* | Potassium voltage-gated channel subfamily B member 1 | ENSG00000158445 | 1.18 | 0.04 |
| *NCAPG* | Condensin complex subunit 3 | ENSG00000109805 | 1.18 | 0.02 |
| *CDC25A* | M-phase inducer phosphatase 1 | ENSG00000164045 | 1.18 | 0.05 |
| *TTK* | Dual specificity protein kinase TTK | ENSG00000112742 | 1.18 | 0.04 |
| *BRIP1* | Fanconi anemia group J protein | ENSG00000136492 | 1.18 | 0.02 |
| *ARHGAP11A* | Rho GTPase-activating protein 11A | ENSG00000198826 | 1.18 | 7.47E-03 |
| *CIT* | Citron Rho-interacting kinase | ENSG00000122966 | 1.17 | 0.02 |
| *CCNB1* | G2/mitotic-specific cyclin-B1 | ENSG00000134057 | 1.17 | 0.02 |
| *CDK1* | Cyclin-dependent kinase 1 | ENSG00000170312 | 1.16 | 0.02 |
| *TROAP* | Tastin | ENSG00000135451 | 1.16 | 0.04 |
| *APCDD1L* | Protein APCDD1-like | ENSG00000198768 | 1.16 | 7.47E-03 |
| *ESPL1* | Separin | ENSG00000135476 | 1.16 | 0.04 |
| *TPX2* | Targeting protein for Xklp2 | ENSG00000088325 | 1.16 | 0.02 |
| *MARCHF4* | E3 ubiquitin-protein ligase MARCHF4 | ENSG00000144583 | 1.15 | 4.21E-03 |
| *CNN1* | Calponin-1 | ENSG00000130176 | 1.15 | 8.92E-04 |
| *CENPF* | Centromere protein F | ENSG00000117724 | 1.15 | 0.03 |
| *GALNT3* | Polypeptide N-acetylgalactosaminyltransferase 3 | ENSG00000115339 | 1.15 | 0.05 |
| *BIRC5* | Baculoviral IAP repeat-containing protein 5 | ENSG00000089685 | 1.14 | 0.03 |
| *ZNF714* | Zinc finger protein 714 | ENSG00000160352 | 1.14 | 0.04 |
| *POLQ* | DNA polymerase theta | ENSG00000051341 | 1.13 | 0.04 |
| *ANGPTL4* | Angiopoietin-related protein 4 | ENSG00000167772 | 1.13 | 0.04 |
| *BARD1* | BRCA1-associated RING domain protein 1 | ENSG00000138376 | 1.12 | 0.03 |
| *SERPINE1* | Plasminogen activator inhibitor 1 | ENSG00000106366 | 1.12 | 9.72E-04 |
| *BRCA2* | Breast cancer type 2 susceptibility protein | ENSG00000139618 | 1.12 | 0.02 |
| *RAD51AP1* | RAD51-associated protein 1 | ENSG00000111247 | 1.12 | 0.04 |
| *KIF2C* | Kinesin-like protein KIF2C | ENSG00000142945 | 1.12 | 0.04 |
| *FST* | Follistatin | ENSG00000134363 | 1.11 | 2.90E-04 |
| *ZNF367* | Zinc finger protein 367 | ENSG00000165244 | 1.1 | 0.03 |
| *CKAP2* | Cytoskeleton-associated protein 2 | ENSG00000136108 | 1.1 | 0.01 |
| *RACGAP1* | Rac GTPase-activating protein 1 | ENSG00000161800 | 1.1 | 0.02 |
| *CIP2A* | Protein CIP2A | ENSG00000163507 | 1.09 | 0.03 |
| *KIF20B* | Kinesin-like protein KIF20B | ENSG00000138182 | 1.09 | 0.04 |
| *FGF5* | Fibroblast growth factor 5 | ENSG00000138675 | 1.09 | 3.47E-04 |
| *GEN1* | Flap endonuclease GEN homolog 1 | ENSG00000178295 | 1.08 | 0.01 |
| *CCNF* | Cyclin-F | ENSG00000162063 | 1.08 | 0.04 |
| *KIF20A* | Kinesin-like protein KIF20A | ENSG00000112984 | 1.07 | 0.05 |
| *CENPU* | Centromere protein U | ENSG00000151725 | 1.06 | 0.02 |
| *NUSAP1* | Nucleolar and spindle-associated protein 1 | ENSG00000137804 | 1.06 | 0.04 |
| *TIPARP* | Protein mono-ADP-ribosyltransferase TIPARP | ENSG00000163659 | 1.06 | 0.02 |
| *RAB3B* | Ras-related protein Rab-3B | ENSG00000169213 | 1.06 | 0.01 |
| *FANCA* | Fanconi anemia group A protein | ENSG00000187741 | 1.06 | 0.03 |
| *SGO2* | Shugoshin 2 | ENSG00000163535 | 1.05 | 0.04 |
| *KIF11* | Kinesin-like protein KIF11 | ENSG00000138160 | 1.04 | 0.04 |
| *ZNF578* | Zinc finger protein 578 | ENSG00000258405 | 1.04 | 0.03 |
| *DTL* | Denticleless protein homolog | ENSG00000143476 | 1.04 | 0.04 |
| *TACC3* | Transforming acidic coiled-coil-containing protein 3 | ENSG00000013810 | 1.04 | 0.04 |
| *LRRC3* | Leucine-rich repeat-containing protein 3 | ENSG00000160233 | 1.04 | 0.03 |
| *ELL2* | RNA polymerase II elongation factor ELL2 | ENSG00000118985 | 1.03 | 5.50E-03 |
| *CDT1* | DNA replication factor Cdt1 | ENSG00000167513 | 1.03 | 0.04 |
| *FANCI* | Fanconi anemia group I protein | ENSG00000140525 | 1.03 | 0.03 |
| *CCDC18* | Coiled-Coil Domain Containing 18 | ENSG00000122483 | 1.02 | 0.04 |
| *ORC6* | Origin recognition complex subunit 6 | ENSG00000091651 | 1.02 | 0.03 |
| *MAD2L1* | Mitotic spindle assembly checkpoint protein MAD2A | ENSG00000164109 | 1.01 | 0.04 |
| *SMAD9* | Mothers against decapentaplegic homolog 9 | ENSG00000120693 | 1.01 | 0.04 |
| *MYEF2* | Myelin expression factor 2 | ENSG00000104177 | 1.01 | 0.01 |
