# Supplementary material for: EMILIN-1 Suppresses Cell Proliferation through Altered Cell Cycle Regulation in Head and Neck Squamous Cell Carcinoma
Source: Am J Pathol. 2025 Jan 30;195(5):995–1012. doi: 10.1016/j.ajpath.2025.01.010 (PMC12163418; doi:10.1016/j.ajpath.2025.01.010)
Supplement: Supplemental Table S10 [file mmc10.docx]

| **Supplemental Table S10** Downregulated genes of CAF3 cell with EMILIN-1 knockdown (Log2FC<-1,FDR<0.05).  (https://www.ensembl.org) | | | |  |
| --- | --- | --- | --- | --- |
|  |  |  |  |  |
| **Gene** | **Database name** | **Identifier** | **Log2FC** | **FDR p-value** |
| *TEN1-CDK3* | TEN1-CDK3 Readthrough | ENSG00000261408 | -4.43 | 4.06E-03 |
| *JAKMIP2* | Janus kinase and microtubule-interacting protein 2 | ENSG00000176049 | -4.08 | 6.17E-03 |
| *NKD2* | Protein naked cuticle homolog 2 | ENSG00000145506 | -4.07 | 9.94E-07 |
| *INSRR* | Insulin receptor-related protein | ENSG00000027644 | -3.72 | 9.94E-07 |
| *NTRK1* | High affinity nerve growth factor receptor | ENSG00000198400 | -3.55 | 8.14E-03 |
| *EMILIN1* | Elastin microfibril interfacer 1 | ENSG00000138080 | -3.36 | 3.20E-54 |
| *CCDC169-SOHLH2* | CCDC169-SOHLH2 readthrough | ENSG00000250709 | -3.29 | 3.28E-03 |
| *C11orf53* | Chromosome 11 open reading frame 53 | ENSG00000150750 | -3.07 | 0.01 |
| *OGN* | Mimecan | ENSG00000106809 | -3.07 | 1.60E-04 |
| *COL15A1* | Collagen alpha-1(XV) chain | ENSG00000204291 | -2.96 | 1.33E-06 |
| *FGF10* | Fibroblast growth factor 10 | ENSG00000070193 | -2.89 | 0.02 |
| *ABCD2* | ATP-binding cassette sub-family D member 2 | ENSG00000173208 | -2.8 | 0.02 |
| *COL21A1* | Collagen alpha-1(XXI) chain | ENSG00000124749 | -2.59 | 7.55E-03 |
| *CFI* | Complement factor I | ENSG00000205403 | -2.58 | 2.82E-06 |
| *CHRDL2* | Chordin-like protein 2 | ENSG00000054938 | -2.56 | 1.30E-03 |
| *FAIM2* | Protein lifeguard 2 | ENSG00000135472 | -2.46 | 4.24E-08 |
| *COL14A1* | Collagen alpha-1(XIV) chain | ENSG00000187955 | -2.45 | 5.70E-05 |
| *TNFSF12-TNFSF13* | TNFSF12-TNFSF13 Readthrough | ENSG00000248871 | -2.42 | 6.60E-03 |
| *RAB4B-EGLN2* | RAB4B-EGLN2 Readthrough | ENSG00000171570 | -2.42 | 6.40E-05 |
| *SERPINA3* | Alpha-1-antichymotrypsin | ENSG00000196136 | -2.38 | 6.65E-03 |
| *NTRK3* | NT-3 growth factor receptor | ENSG00000140538 | -2.38 | 1.18E-03 |
| *CXCL1* | Growth-regulated alpha protein | ENSG00000163739 | -2.29 | 2.59E-06 |
| *CXCL2* | C-X-C motif chemokine 2 | ENSG00000081041 | -2.23 | 1.04E-03 |
| *EGFL6* | Epidermal growth factor-like protein 6 | ENSG00000198759 | -2.2 | 4.66E-04 |
| *INHBE* | Inhibin beta E chain | ENSG00000139269 | -2.13 | 0.01 |
| *OMD* | Osteomodulin | ENSG00000127083 | -2.11 | 6.40E-05 |
| *GRIA1* | Glutamate receptor 1 | ENSG00000155511 | -2.07 | 5.70E-05 |
| *RSPO1* | R-spondin-1 | ENSG00000169218 | -2.04 | 0.02 |
| *IL16* | Pro-interleukin-16 | ENSG00000172349 | -2.04 | 1.41E-03 |
| *TMCC3* | Transmembrane and coiled-coil domain protein 3 | ENSG00000057704 | -2 | 8.97E-03 |
| *SERPINA5* | Plasma serine protease inhibitor | ENSG00000188488 | -2 | 0.03 |
| *CSTA* | Cystatin-A | ENSG00000121552 | -1.99 | 7.67E-03 |
| *CACNG7* | Voltage-dependent calcium channel gamma-7 subunit | ENSG00000105605 | -1.97 | 6.21E-04 |
| *DNM1* | Dynamin-1 | ENSG00000106976 | -1.93 | 3.50E-07 |
| *KCNT2* | Potassium channel subfamily T member 2 | ENSG00000162687 | -1.92 | 1.04E-03 |
| *GDF7* | Growth/differentiation factor 7 | ENSG00000143869 | -1.88 | 2.55E-03 |
| *METTL24* | Probable methyltransferase-like protein 24 | ENSG00000053328 | -1.86 | 7.93E-04 |
| *STRA6* | Receptor for retinol uptake STRA6 | ENSG00000137868 | -1.85 | 9.94E-07 |
| *CXCL6* | C-X-C motif chemokine 6 | ENSG00000124875 | -1.85 | 7.29E-03 |
| *VEGFD* | Vascular endothelial growth factor D | ENSG00000165197 | -1.85 | 0.01 |
| *TNFSF18* | Tumor necrosis factor ligand superfamily member 18 | ENSG00000120337 | -1.84 | 3.93E-03 |
| *ST8SIA1* | Alpha-N-acetylneuraminide alpha-2,8-sialyltransferase | ENSG00000111728 | -1.81 | 0.04 |
| *FRZB* | Secreted frizzled-related protein 3 | ENSG00000162998 | -1.8 | 6.57E-04 |
| *CRABP2* | Cellular retinoic acid-binding protein 2 | ENSG00000143320 | -1.8 | 5.70E-05 |
| *ADAMTS9* | A disintegrin and metalloproteinase with thrombospondin motifs 9 | ENSG00000163638 | -1.8 | 0.04 |
| *SYT1* | Synaptotagmin-1 | ENSG00000067715 | -1.77 | 0.04 |
| *TMEM130* | Transmembrane protein 130 | ENSG00000166448 | -1.77 | 6.78E-06 |
| *ADAM22* | Disintegrin and metalloproteinase domain-containing protein 22 | ENSG00000008277 | -1.77 | 0.02 |
| *CDON* | Cell adhesion molecule-related/down-regulated by oncogenes | ENSG00000064309 | -1.76 | 4.87E-03 |
| *ADAMTS8* | A disintegrin and metalloproteinase with thrombospondin motifs 8 | ENSG00000134917 | -1.75 | 0.01 |
| *LMTK3* | Serine/threonine-protein kinase LMTK3 | ENSG00000142235 | -1.73 | 0.03 |
| *SCN2A* | Sodium channel protein type 2 subunit alpha | ENSG00000136531 | -1.71 | 5.52E-04 |
| *SPON1* | Spondin-1 | ENSG00000262655 | -1.7 | 2.24E-04 |
| *DPT* | Dermatopontin | ENSG00000143196 | -1.68 | 1.09E-03 |
| *TMEM100* | Transmembrane protein 100 | ENSG00000166292 | -1.67 | 0.02 |
| *GAS1* | Growth arrest-specific protein 1 | ENSG00000180447 | -1.67 | 7.74E-05 |
| *ITGA9* | Integrin alpha-9 | ENSG00000144668 | -1.66 | 0.04 |
| *APOD* | Apolipoprotein D | ENSG00000189058 | -1.65 | 2.73E-03 |
| *BMP4* | Bone morphogenetic protein 4 | ENSG00000125378 | -1.64 | 2.12E-03 |
| *SORL1* | Sortilin-related receptor | ENSG00000137642 | -1.63 | 1.30E-03 |
| *GDF1* | Embryonic growth/differentiation factor 1 | ENSG00000130283 | -1.63 | 0.03 |
| *RAB39B* | Ras-related protein Rab-39B | ENSG00000155961 | -1.63 | 0.02 |
| *SNCA* | Alpha-synuclein | ENSG00000145335 | -1.62 | 7.02E-04 |
| *GALNT16* | Polypeptide N-acetylgalactosaminyltransferase 16 | ENSG00000100626 | -1.61 | 8.92E-04 |
| *TRIL* | TLR4 interactor with leucine rich repeats | ENSG00000255690 | -1.61 | 5.08E-03 |
| *WNT2* | Protein Wnt-2 | ENSG00000105989 | -1.6 | 4.94E-03 |
| *SORBS2* | Sorbin and SH3 domain-containing protein 2 | ENSG00000154556 | -1.6 | 0.04 |
| *PRELP* | Prolargin | ENSG00000188783 | -1.57 | 7.85E-03 |
| *EPHB2* | Ephrin type-B receptor 2 | ENSG00000133216 | -1.55 | 7.41E-03 |
| *IGSF10* | Immunoglobulin superfamily member 10 | ENSG00000152580 | -1.51 | 6.99E-03 |
| *CNTN1* | Contactin-1 | ENSG00000018236 | -1.5 | 4.27E-03 |
| *NR4A2* | Nuclear receptor subfamily 4 group A member 2 | ENSG00000153234 | -1.5 | 0.04 |
| *PDGFD* | Platelet-derived growth factor D | ENSG00000170962 | -1.5 | 2.48E-03 |
| *PTHLH* | Parathyroid hormone-related protein | ENSG00000087494 | -1.49 | 9.96E-03 |
| *EGR3* | Early growth response protein 3 | ENSG00000179388 | -1.49 | 0.04 |
| *IL7* | Interleukin-7 | ENSG00000104432 | -1.48 | 0.04 |
| *SLIT3* | Slit homolog 3 protein | ENSG00000184347 | -1.47 | 1.05E-03 |
| *TMEFF2* | Tomoregulin-2 | ENSG00000144339 | -1.46 | 0.01 |
| *GGT5* | Glutathione hydrolase 5 proenzyme | ENSG00000099998 | -1.45 | 0.04 |
| *ACKR3* | Atypical chemokine receptor 3 | ENSG00000144476 | -1.44 | 0.02 |
| *SLC6A9* | Sodium- and chloride-dependent glycine transporter 1 | ENSG00000196517 | -1.42 | 3.03E-03 |
| *TM7SF2* | Delta(14)-sterol reductase TM7SF2 | ENSG00000149809 | -1.42 | 0.04 |
| *ASPN* | Asporin | ENSG00000106819 | -1.41 | 9.98E-04 |
| *SCARA5* | Scavenger receptor class A member 5 | ENSG00000168079 | -1.41 | 0.03 |
| *IL17RD* | Interleukin-17 receptor D | ENSG00000144730 | -1.41 | 6.40E-05 |
| *CCN4* | CCN family member 4 | ENSG00000104415 | -1.39 | 0.04 |
| *NEFL* | Neurofilament light polypeptide | ENSG00000277586 | -1.38 | 1.55E-03 |
| *PDGFRL* | Platelet-derived growth factor receptor-like protein | ENSG00000104213 | -1.38 | 0.02 |
| *OLFM2* | Noelin-2 | ENSG00000105088 | -1.37 | 0.01 |
| *CDO1* | Cysteine dioxygenase type 1 | ENSG00000129596 | -1.35 | 4.20E-03 |
| *DBP* | D site-binding protein | ENSG00000105516 | -1.35 | 0.03 |
| *ENPP5* | Ectonucleotide pyrophosphatase/phosphodiesterase family member 5 | ENSG00000112796 | -1.35 | 0.01 |
| *GPR162* | Probable G-protein coupled receptor 162 | ENSG00000250510 | -1.34 | 0.04 |
| *EGR1* | Early growth response protein 1 | ENSG00000120738 | -1.31 | 0.03 |
| *TNFSF4* | Tumor necrosis factor ligand superfamily member 4 | ENSG00000117586 | -1.3 | 0.02 |
| *TNFAIP6* | Tumor necrosis factor-inducible gene 6 protein | ENSG00000123610 | -1.29 | 0.04 |
| *KSR1* | Kinase suppressor of Ras 1 | ENSG00000141068 | -1.29 | 7.23E-04 |
| *NPIPA9* | Nuclear pore complex-interacting protein family, member A9 | ENSG00000233024 | -1.29 | 0.01 |
| *SLC24A3* | Sodium/potassium/calcium exchanger 3 | ENSG00000185052 | -1.28 | 0.04 |
| *NR4A1* | Nuclear receptor subfamily 4 group A member 1 | ENSG00000123358 | -1.27 | 9.01E-03 |
| *TRIB3* | Tribbles homolog 3 | ENSG00000101255 | -1.27 | 3.47E-04 |
| *GNG2* | Guanine nucleotide-binding protein G(I)/G(S)/G(O) subunit gamma-2 | ENSG00000186469 | -1.26 | 3.93E-03 |
| *PIK3C2B* | Phosphatidylinositol 4-phosphate 3-kinase C2 domain-containing subunit beta | ENSG00000133056 | -1.25 | 7.40E-03 |
| *INMT* | Indolethylamine N-methyltransferase | ENSG00000241644 | -1.25 | 0.03 |
| *C1QTNF6* | Complement C1q tumor necrosis factor-related protein 6 | ENSG00000133466 | -1.25 | 7.96E-06 |
| *NPTX1* | Neuronal pentraxin-1 | ENSG00000171246 | -1.24 | 2.48E-03 |
| *CEBPD* | CCAAT/enhancer-binding protein delta | ENSG00000221869 | -1.24 | 2.56E-05 |
| *AQP1* | Aquaporin-1 | ENSG00000240583 | -1.24 | 5.42E-03 |
| *CPZ* | Carboxypeptidase Z | ENSG00000109625 | -1.24 | 0.01 |
| *JUNB* | Transcription factor jun-B | ENSG00000171223 | -1.22 | 5.98E-03 |
| *FMOD* | Fibromodulin | ENSG00000122176 | -1.22 | 1.06E-03 |
| *HES1* | Transcription factor HES-1 | ENSG00000114315 | -1.22 | 0.05 |
| *THRB* | Thyroid hormone receptor beta | ENSG00000151090 | -1.2 | 0.01 |
| *SSC5D* | Soluble scavenger receptor cysteine-rich domain-containing protein SSC5D | ENSG00000179954 | -1.18 | 6.21E-04 |
| *SLC6A15* | Sodium-dependent neutral amino acid transporter B(0)AT2 | ENSG00000072041 | -1.18 | 0.03 |
| *PLEKHA6* | Pleckstrin homology domain-containing family A member 6 | ENSG00000143850 | -1.17 | 0.02 |
| *SHC3* | SHC-transforming protein 3 | ENSG00000148082 | -1.17 | 5.10E-04 |
| *COL5A1* | Collagen alpha-1(V) chain | ENSG00000130635 | -1.17 | 1.59E-03 |
| *TMTC2* | Protein O-mannosyl-transferase TMTC2 | ENSG00000179104 | -1.16 | 6.21E-04 |
| *SOCS1* | Suppressor of cytokine signaling 1 | ENSG00000185338 | -1.16 | 0.02 |
| *IGF2* | Insulin-like growth factor II | ENSG00000167244 | -1.16 | 1.47E-03 |
| *ADRA1B* | Alpha-1B adrenergic receptor | ENSG00000170214 | -1.15 | 0.02 |
| *GXYLT2* | Glucoside xylosyltransferase 2 | ENSG00000172986 | -1.14 | 2.98E-03 |
| *PCDHGB6* | Protocadherin gamma-B6 | ENSG00000253305 | -1.13 | 6.52E-03 |
| *BHLHE40* | Class E basic helix-loop-helix protein 40 | ENSG00000134107 | -1.13 | 0.04 |
| *IL17D* | Interleukin-17D | ENSG00000172458 | -1.13 | 0.03 |
| *HR* | Lysine-specific demethylase hairless | ENSG00000168453 | -1.12 | 8.14E-03 |
| *ADAMTS5* | A disintegrin and metalloproteinase with thrombospondin motifs 5 | ENSG00000154736 | -1.12 | 7.07E-04 |
| *GPM6B* | Neuronal membrane glycoprotein M6-b | ENSG00000046653 | -1.12 | 0.05 |
| *DCHS1* | Protocadherin-16 | ENSG00000166341 | -1.11 | 3.67E-03 |
| *JAM2* | Junctional adhesion molecule B | ENSG00000154721 | -1.11 | 0.01 |
| *LAMA5* | Laminin subunit alpha-5 | ENSG00000130702 | -1.1 | 0.03 |
| *THBS3* | Thrombospondin-3 | ENSG00000169231 | -1.1 | 6.75E-05 |
| *TENM4* | Teneurin-4 | ENSG00000149256 | -1.09 | 9.72E-04 |
| *SETBP1* | SET-binding protein | ENSG00000152217 | -1.07 | 7.47E-03 |
| *PSAT1* | Phosphoserine aminotransferase | ENSG00000135069 | -1.07 | 1.30E-03 |
| *PCSK5* | Proprotein convertase subtilisin/kexin type 5 | ENSG00000099139 | -1.06 | 7.41E-03 |
| *TMEM119* | Transmembrane protein 119 | ENSG00000183160 | -1.06 | 0.01 |
| *MXRA5* | Matrix-remodeling-associated protein 5 | ENSG00000101825 | -1.06 | 0.01 |
| *SEMA3B* | Semaphorin-3B | ENSG00000012171 | -1.06 | 1.45E-03 |
| *ANKRD13B* | Ankyrin repeat domain-containing protein 13B | ENSG00000198720 | -1.05 | 9.11E-03 |
| *FIBIN* | Fin bud initiation factor homolog | ENSG00000176971 | -1.05 | 0.01 |
| *DAB1* | Disabled homolog 1 | ENSG00000173406 | -1.05 | 0.02 |
| *SERPINF1* | Pigment epithelium-derived factor | ENSG00000132386 | -1.04 | 7.41E-03 |
| *NID1* | Nidogen-1 | ENSG00000116962 | -1.04 | 1.30E-03 |
| *ANGPTL2* | Angiopoietin-related protein 2 | ENSG00000136859 | -1.04 | 0.02 |
| *HCFC1R1* | Host cell factor C1 regulator 1 | ENSG00000103145 | -1.04 | 0.02 |
| *FBLN2* | Fibulin-2 | ENSG00000163520 | -1.03 | 3.04E-03 |
| *TCN2* | Transcobalamin-2 | ENSG00000185339 | -1.03 | 9.78E-03 |
| *ITPKB* | Inositol-trisphosphate 3-kinase B | ENSG00000143772 | -1.02 | 0.04 |
| *KIAA1755* | KIAA1755 | ENSG00000149633 | -1.02 | 8.92E-04 |
| *CFH* | Complement factor H | ENSG00000000971 | -1.02 | 3.41E-04 |
| *TENM3* | Teneurin-3 | ENSG00000218336 | -1.02 | 2.60E-03 |
| *ISLR* | Immunoglobulin superfamily containing leucine-rich repeat protein | ENSG00000129009 | -1.02 | 2.48E-03 |
| *MFAP4* | Microfibril-associated glycoprotein 4 | ENSG00000166482 | -1.02 | 1.45E-03 |
| *VASH1* | Tubulinyl-Tyr carboxypeptidase 1 | ENSG00000071246 | -1.01 | 6.40E-03 |
| *UNC5B* | Netrin receptor UNC5B | ENSG00000107731 | -1.01 | 0.04 |
| *LTBP4* | Latent-transforming growth factor beta-binding protein 4 | ENSG00000090006 | -1 | 2.40E-04 |
