# Supplementary material for: EMILIN-1 Suppresses Cell Proliferation through Altered Cell Cycle Regulation in Head and Neck Squamous Cell Carcinoma
Source: Am J Pathol. 2025 Jan 30;195(5):995–1012. doi: 10.1016/j.ajpath.2025.01.010 (PMC12163418; doi:10.1016/j.ajpath.2025.01.010)
Supplement: Supplemental Table S11 [file mmc11.docx]

| **Supplemental Table S11** Upregulated genes of NF3 cell with EMILIN-1 knockdown (Log2FC>1,FDR<0.05).  (https://www.ensembl.org) | | | |  |
| --- | --- | --- | --- | --- |
|  |  |  |  |  |
| **Gene** | **Database name** | **Identifier** | **Log2FC** | **FDR p-value** |
| *TBC1D3* | TBC1 domain family member 3 | ENSG00000274611 | 9.68 | 0.03 |
| *OR2I1P* | Putative olfactory receptor 2I1 | ENSG00000237988 | 9.07 | 0.05 |
| *KRT14* | Keratin, type I cytoskeletal 14 | ENSG00000186847 | 8.72 | 0.03 |
| *UBD* | Ubiquitin D | ENSG00000213886 | 7.17 | 1.66E-06 |
| *HOXD3* | Homeobox protein Hox-D3 | ENSG00000128652 | 6.95 | 1.18E-05 |
| *HOXA4* | Homeobox protein Hox-A4 | ENSG00000197576 | 5.5 | 0.03 |
| *POU3F3* | POU domain, class 3, transcription factor 3 | ENSG00000198914 | 5.19 | 3.92E-03 |
| *TBC1D3D* | TBC1 domain family member 3D | ENSG00000274419 | 5.13 | 0.03 |
| *HOXD4* | Homeobox protein Hox-D4 | ENSG00000170166 | 4.91 | 1.78E-04 |
| *PIP* | Prolactin-inducible protein | ENSG00000159763 | 4.87 | 7.61E-03 |
| *VCAM1* | Vascular cell adhesion protein 1 | ENSG00000162692 | 4.79 | 0.03 |
| *CHRNA7* | Neuronal acetylcholine receptor subunit alpha-7 | ENSG00000175344 | 4.69 | 3.28E-05 |
| *OTULINL* | Inactive ubiquitin thioesterase OTULINL | ENSG00000145569 | 4.22 | 5.54E-05 |
| *GSTM1* | Glutathione S-transferase Mu 1 | ENSG00000134184 | 4.06 | 2.34E-03 |
| *FGF9* | Fibroblast growth factor 9 | ENSG00000102678 | 3.99 | 0.02 |
| *HOXA3* | Homeobox protein Hox-A3 | ENSG00000105997 | 3.87 | 3.41E-03 |
| *HAPLN1* | Hyaluronan and proteoglycan link protein 1 | ENSG00000145681 | 3.75 | 6.14E-06 |
| *TAC1* | Protachykinin-1 | ENSG00000006128 | 3.61 | 6.61E-03 |
| *EPGN* | Epigen | ENSG00000182585 | 3.53 | 0.02 |
| *SLC14A1* | Urea transporter 1 | ENSG00000141469 | 3.51 | 4.89E-03 |
| *CTNND2* | Catenin delta-2 | ENSG00000169862 | 3.48 | 4.23E-04 |
| *CYSLTR1* | Cysteinyl leukotriene receptor 1 | ENSG00000173198 | 3.41 | 0.01 |
| *FLG* | Filaggrin | ENSG00000143631 | 3.41 | 4.23E-03 |
| *RUNDC3A* | RUN domain-containing protein 3A | ENSG00000108309 | 3.36 | 7.81E-05 |
| *RBP4* | Retinol-binding protein 4 | ENSG00000138207 | 3.2 | 0.01 |
| *GATD3A* |  | ENSG00000160221 | 3.05 | 7.71E-03 |
| *ACTG2* | Actin, gamma-enteric smooth muscle | ENSG00000163017 | 3.02 | 3.44E-18 |
| *ALDH3A1* | Aldehyde dehydrogenase, dimeric NADP-preferring | ENSG00000108602 | 2.95 | 3.92E-03 |
| *HSD17B6* | 17-beta-hydroxysteroid dehydrogenase type 6 | ENSG00000025423 | 2.91 | 0.03 |
| *GAL* | Galanin peptides | ENSG00000069482 | 2.81 | 0.02 |
| *TCEAL2* | Transcription elongation factor A protein-like 2 | ENSG00000184905 | 2.65 | 0.05 |
| *SLC27A6* | Long-chain fatty acid transport protein 6 | ENSG00000113396 | 2.59 | 0.01 |
| *ESM1* | Endothelial cell-specific molecule 1 | ENSG00000164283 | 2.56 | 5.34E-09 |
| *CHRFAM7A* | CHRNA7-FAM7A fusion protein | ENSG00000166664 | 2.52 | 0.02 |
| *COL22A1* | Collagen alpha-1(XXII) chain | ENSG00000169436 | 2.44 | 0.03 |
| *LAMC3* | Laminin subunit gamma-3 | ENSG00000050555 | 2.36 | 3.92E-03 |
| *GRIK2* | Glutamate receptor ionotropic, kainate 2 | ENSG00000164418 | 2.36 | 4.31E-03 |
| *SHE* | SH2 domain-containing adapter protein E | ENSG00000169291 | 2.34 | 0.03 |
| *CXCL14* | C-X-C motif chemokine 14 | ENSG00000145824 | 2.18 | 4.16E-04 |
| *ALDH1A1* | Aldehyde dehydrogenase 1A1 | ENSG00000165092 | 2.1 | 7.73E-03 |
| *WNT2* | Protein Wnt-2 | ENSG00000105989 | 2.09 | 1.88E-04 |
| *ZNF560* | Zinc finger protein 560 | ENSG00000198028 | 2.05 | 0.02 |
| *LAMC2* | Laminin subunit gamma-2 | ENSG00000058085 | 1.96 | 1.43E-03 |
| *EDIL3* | EGF-like repeat and discoidin I-like domain-containing protein 3 | ENSG00000164176 | 1.95 | 0.04 |
| *SRGN* | Serglycin | ENSG00000122862 | 1.91 | 5.59E-03 |
| *NPIPA5* | Nuclear pore complex-interacting protein family member A5 | ENSG00000183793 | 1.83 | 0.01 |
| *IGFBP3* | Insulin-like growth factor-binding protein 3 | ENSG00000146674 | 1.8 | 3.47E-04 |
| *MMP12* | Macrophage metalloelastase | ENSG00000262406 | 1.79 | 0.02 |
| *TMEM132B* | Transmembrane protein 132B | ENSG00000139364 | 1.77 | 3.01E-03 |
| *DEPP1* | Protein DEPP1 | ENSG00000165507 | 1.75 | 7.12E-04 |
| *TMEM176A* | Transmembrane protein 176A | ENSG00000002933 | 1.74 | 0.02 |
| *NPAS1* | Neuronal PAS domain-containing protein 1 | ENSG00000130751 | 1.73 | 0.04 |
| *TAGLN* | Transgelin | ENSG00000149591 | 1.66 | 4.47E-03 |
| *ENPP4* | Bis(5'-adenosyl)-triphosphatase ENPP4 | ENSG00000001561 | 1.65 | 0.02 |
| *MASP1* | Mannan-binding lectin serine protease 1 | ENSG00000127241 | 1.63 | 7.12E-04 |
| *RNF227* | RING finger protein 227 | ENSG00000179859 | 1.6 | 4.39E-03 |
| *IRAG1* | Inositol 1,4,5-triphosphate receptor associated 1 | ENSG00000072952 | 1.57 | 1.96E-04 |
| *ASPM* | Abnormal spindle-like microcephaly-associated protein | ENSG00000066279 | 1.57 | 6.66E-04 |
| *TNC* | Tenascin | ENSG00000041982 | 1.57 | 1.31E-05 |
| *HMMR* | Hyaluronan mediated motility receptor | ENSG00000072571 | 1.55 | 8.17E-03 |
| *KIF14* | Kinesin-like protein KIF14 | ENSG00000118193 | 1.53 | 4.11E-03 |
| *LOX* | Protein-lysine 6-oxidase | ENSG00000113083 | 1.51 | 3.13E-03 |
| *ANO3* | Anoctamin-3 | ENSG00000134343 | 1.5 | 4.80E-03 |
| *BRIP1* | Fanconi anemia group J protein | ENSG00000136492 | 1.42 | 0.01 |
| *ALDH1A3* | Aldehyde dehydrogenase family 1 member A3 | ENSG00000184254 | 1.42 | 0.01 |
| *TCAF2* | TRPM8 channel-associated factor 2 | ENSG00000170379 | 1.4 | 5.57E-03 |
| *CENPE* | Centromere-associated protein E | ENSG00000138778 | 1.38 | 5.55E-04 |
| *TOP2A* | DNA topoisomerase 2-alpha | ENSG00000131747 | 1.36 | 9.17E-03 |
| *TMEM176B* | Transmembrane protein 176B | ENSG00000106565 | 1.36 | 0.03 |
| *APCDD1L* | Protein APCDD1-like | ENSG00000198768 | 1.34 | 1.94E-03 |
| *KNL1* | Kinetochore scaffold 1 | ENSG00000137812 | 1.33 | 0.01 |
| *LMOD1* | Leiomodin-1 | ENSG00000163431 | 1.32 | 2.69E-05 |
| *MALL* | MAL-like protein | ENSG00000144063 | 1.29 | 0.05 |
| *KIAA1549L* | UPF0606 protein KIAA1549L | ENSG00000110427 | 1.29 | 8.56E-03 |
| *DSP* | Desmoplakin | ENSG00000096696 | 1.27 | 1.51E-03 |
| *KIF20B* | Kinesin-like protein KIF20B | ENSG00000138182 | 1.26 | 3.01E-03 |
| *ACTA2* | Actin, aortic smooth muscle | ENSG00000107796 | 1.25 | 0.05 |
| *MET* | Hepatocyte growth factor receptor | ENSG00000105976 | 1.23 | 6.61E-03 |
| *ABAT* | 4-aminobutyrate aminotransferase, mitochondrial | ENSG00000183044 | 1.22 | 3.84E-03 |
| *SULF1* | Extracellular sulfatase Sulf-1 | ENSG00000137573 | 1.21 | 0.02 |
| *BUB1B* | Mitotic checkpoint serine/threonine-protein kinase BUB1 beta | ENSG00000156970 | 1.21 | 0.05 |
| *ANLN* | Anillin | ENSG00000011426 | 1.19 | 0.05 |
| *FN1* | Fibronectin | ENSG00000115414 | 1.19 | 0.05 |
| *USP53* | Inactive ubiquitin carboxyl-terminal hydrolase 53 | ENSG00000145390 | 1.18 | 0.03 |
| *NCAPG* | Condensin complex subunit 3 | ENSG00000109805 | 1.17 | 0.05 |
| *CIT* | Citron Rho-interacting kinase | ENSG00000122966 | 1.17 | 2.73E-03 |
| *ARHGAP11A* | Rho GTPase-activating protein 11A | ENSG00000198826 | 1.17 | 0.03 |
| *IFI27* | Interferon alpha-inducible protein 27, mitochondrial | ENSG00000165949 | 1.17 | 0.02 |
| *KIF18A* | Kinesin-like protein KIF18A | ENSG00000121621 | 1.15 | 0.05 |
| *SGO2* | Shugoshin 2 | ENSG00000163535 | 1.1 | 0.03 |
| *GALNT15* | Polypeptide N-acetylgalactosaminyltransferase 15 | ENSG00000131386 | 1.09 | 3.92E-03 |
| *CKAP2* | Cytoskeleton-associated protein 2 | ENSG00000136108 | 1.08 | 0.02 |
| *DNMBP* | Dynamin-binding protein | ENSG00000107554 | 1.07 | 0.02 |
| *LMCD1* | LIM and cysteine-rich domains protein 1 | ENSG00000071282 | 1.05 | 0.03 |
| *CEMIP2* | Cell surface hyaluronidase | ENSG00000135048 | 1.02 | 0.02 |
