# Supplementary material for: EMILIN-1 Suppresses Cell Proliferation through Altered Cell Cycle Regulation in Head and Neck Squamous Cell Carcinoma
Source: Am J Pathol. 2025 Jan 30;195(5):995–1012. doi: 10.1016/j.ajpath.2025.01.010 (PMC12163418; doi:10.1016/j.ajpath.2025.01.010)
Supplement: Supplemental Table S12 [file mmc12.docx]

| **Supplemental Table S12** Downregulated genes of NF3 cell with EMILIN-1 knockdown (Log2FC<-1,FDR<0.05).  (https://www.ensembl.org) | | | | |
| --- | --- | --- | --- | --- |
|  |  |  |  |  |
| **Gene** | **Database name** | **Identifier** | **Log2FC** | **FDR p-value** |
| *RAMP3* | Receptor activity-modifying protein 3 | ENSG00000122679 | -4.91 | 0.02 |
| *FMO1* | Dimethylaniline monooxygenase [N-oxide-forming] 1 | ENSG00000010932 | -4.75 | 1.21E-07 |
| *COL15A1* | Collagen alpha-1(XV) chain | ENSG00000204291 | -4.64 | 6.87E-17 |
| *FABP4* | Fatty acid-binding protein, adipocyte | ENSG00000170323 | -4.32 | 4.41E-04 |
| *CCL8* | C-C motif chemokine 8 | ENSG00000108700 | -4.23 | 6.14E-06 |
| *IL24* | Interleukin-24 | ENSG00000162892 | -4.06 | 0.01 |
| *CXCL2* | C-X-C motif chemokine 2 | ENSG00000081041 | -4.03 | 2.42E-11 |
| *COL21A1* | Collagen alpha-1(XXI) chain | ENSG00000124749 | -3.96 | 1.98E-07 |
| *FAM180B* | Protein FAM180B | ENSG00000196666 | -3.9 | 2.34E-03 |
| *EGR2* | E3 SUMO-protein ligase EGR2 | ENSG00000122877 | -3.88 | 7.01E-05 |
| *NR4A2* | Nuclear receptor subfamily 4 group A member 2 | ENSG00000153234 | -3.64 | 1.81E-14 |
| *SPON1* | Spondin-1 | ENSG00000262655 | -3.6 | 4.11E-11 |
| *SCHIP1* | Schwannomin-interacting protein 1 | ENSG00000151967 | -3.59 | 0.04 |
| *FAIM2* | Protein lifeguard 2 | ENSG00000135472 | -3.59 | 2.68E-18 |
| *KCNC1* | Potassium voltage-gated channel subfamily C member 1 | ENSG00000129159 | -3.58 | 3.01E-03 |
| *SPARCL1* | SPARC-like protein 1 | ENSG00000152583 | -3.56 | 0.01 |
| *CCL11* | Eotaxin | ENSG00000172156 | -3.56 | 1.89E-08 |
| *EMILIN1* | Elastin microfibril interfacer 1 | ENSG00000138080 | -3.55 | 2.04E-37 |
| *HES1* | Transcription factor HES-1 | ENSG00000114315 | -3.34 | 1.09E-14 |
| *NTRK3* | NT-3 growth factor receptor | ENSG00000140538 | -3.2 | 3.11E-03 |
| *DCC* | Netrin receptor DCC | ENSG00000187323 | -3.14 | 0.02 |
| *IL6* | Interleukin-6 | ENSG00000136244 | -3.09 | 4.39E-27 |
| *HSD17B2* | 17-beta-hydroxysteroid dehydrogenase type 2 | ENSG00000086696 | -3.08 | 7.50E-05 |
| *CXCL1* | Growth-regulated alpha protein | ENSG00000163739 | -3.05 | 4.08E-09 |
| *TLL1* | Tolloid-like protein 1 | ENSG00000038295 | -3.05 | 0.01 |
| *PF4V1* | Platelet factor 4 variant | ENSG00000109272 | -3.02 | 0.02 |
| *NR4A1* | Nuclear receptor subfamily 4 group A member 1 | ENSG00000123358 | -2.9 | 4.34E-19 |
| *TMEM130* | Transmembrane protein 130 | ENSG00000166448 | -2.83 | 7.60E-07 |
| *SCARA5* | Scavenger receptor class A member 5 | ENSG00000168079 | -2.78 | 8.27E-04 |
| *SYTL5* | Synaptotagmin-like protein 5 | ENSG00000147041 | -2.74 | 4.80E-03 |
| *AQP1* | Aquaporin-1 | ENSG00000240583 | -2.73 | 7.60E-07 |
| *PLXDC1* | Plexin domain-containing protein 1 | ENSG00000161381 | -2.68 | 3.69E-03 |
| *TMEFF2* | Tomoregulin-2 | ENSG00000144339 | -2.65 | 2.35E-04 |
| *EGR3* | Early growth response protein 3 | ENSG00000179388 | -2.63 | 2.31E-04 |
| *CP* | Ceruloplasmin | ENSG00000047457 | -2.59 | 4.16E-04 |
| *COL14A1* | Collagen alpha-1(XIV) chain | ENSG00000187955 | -2.56 | 5.09E-03 |
| *SYT1* | Synaptotagmin-1 | ENSG00000067715 | -2.55 | 5.95E-03 |
| *NPR1* | Atrial natriuretic peptide receptor 1 | ENSG00000169418 | -2.51 | 6.61E-03 |
| *IL18R1* | Interleukin-18 receptor 1 | ENSG00000115604 | -2.5 | 0.03 |
| *OGN* | Mimecan | ENSG00000106809 | -2.49 | 0.05 |
| *FAM107A* | Actin-associated protein FAM107A | ENSG00000168309 | -2.47 | 3.83E-06 |
| *SLC24A3* | Sodium/potassium/calcium exchanger 3 | ENSG00000185052 | -2.38 | 2.87E-03 |
| *SLC6A15* | Sodium-dependent neutral amino acid transporter B(0)AT2 | ENSG00000072041 | -2.34 | 1.65E-05 |
| *MAP2K6* | Dual specificity mitogen-activated protein kinase kinase 6 | ENSG00000108984 | -2.34 | 0.02 |
| *TLR5* | Toll-like receptor 5 | ENSG00000187554 | -2.33 | 0.04 |
| *SYNGR3* | Synaptogyrin-3 | ENSG00000127561 | -2.3 | 0.03 |
| *PCSK9* | Proprotein convertase subtilisin/kexin type 9 | ENSG00000169174 | -2.28 | 4.13E-04 |
| *FABP3* | Fatty acid-binding protein, heart | ENSG00000121769 | -2.27 | 4.83E-10 |
| *RNF112* | RING finger protein 112 | ENSG00000128482 | -2.26 | 2.72E-03 |
| *NRCAM* | Neuronal cell adhesion molecule | ENSG00000091129 | -2.25 | 6.61E-03 |
| *VEGFD* | Vascular endothelial growth factor D | ENSG00000165197 | -2.25 | 0.04 |
| *PKD1L2* | Polycystic kidney disease protein 1-like 2 | ENSG00000166473 | -2.25 | 3.71E-03 |
| *CSRNP1* | Cysteine/serine-rich nuclear protein 1 | ENSG00000144655 | -2.21 | 3.28E-05 |
| *RAB39B* | Ras-related protein Rab-39B | ENSG00000155961 | -2.2 | 0.02 |
| *C15orf48* | Chromosome 15 Open Reading Frame 48 | ENSG00000166920 | -2.19 | 0.01 |
| *GALNT16* | Polypeptide N-acetylgalactosaminyltransferase 16 | ENSG00000100626 | -2.18 | 9.11E-05 |
| *TMEM100* | Transmembrane protein 100 | ENSG00000166292 | -2.16 | 0.03 |
| *MMP27* | Matrix metalloproteinase-27 | ENSG00000137675 | -2.12 | 0.04 |
| *OMD* | Osteomodulin | ENSG00000127083 | -2.06 | 0.04 |
| *ST8SIA1* | Alpha-N-acetylneuraminide alpha-2,8-sialyltransferase | ENSG00000111728 | -2.05 | 1.99E-03 |
| *IER3* | Radiation-inducible immediate-early gene IEX-1 | ENSG00000137331 | -2.04 | 5.22E-07 |
| *PPARGC1A* | Peroxisome proliferator-activated receptor gamma coactivator 1-alpha | ENSG00000109819 | -2.04 | 3.92E-03 |
| *PIANP* | PILR alpha-associated neural protein | ENSG00000139200 | -2.03 | 1.57E-04 |
| *CXCL6* | C-X-C motif chemokine 6 | ENSG00000124875 | -2.03 | 0.02 |
| *CEND1* | Cell cycle exit and neuronal differentiation protein 1 | ENSG00000184524 | -2.01 | 3.58E-06 |
| *FADS2* | Acyl-CoA 6-desaturase | ENSG00000134824 | -1.96 | 2.25E-10 |
| *PTGS2* | Prostaglandin G/H synthase 2 | ENSG00000073756 | -1.95 | 4.32E-09 |
| *AMT* | Aminomethyltransferase, mitochondrial | ENSG00000145020 | -1.94 | 0.03 |
| *ITGA11* | Integrin alpha-11 | ENSG00000137809 | -1.93 | 1.48E-03 |
| *ATF3* | Cyclic AMP-dependent transcription factor ATF-3 | ENSG00000162772 | -1.88 | 4.91E-04 |
| *PRELP* | Prolargin | ENSG00000188783 | -1.85 | 2.20E-03 |
| *PDGFD* | Platelet-derived growth factor D | ENSG00000170962 | -1.83 | 4.61E-03 |
| *INMT* | Indolethylamine N-methyltransferase | ENSG00000241644 | -1.82 | 3.69E-04 |
| *CACNG7* | Voltage-dependent calcium channel gamma-7 subunit | ENSG00000105605 | -1.81 | 0.01 |
| *APOD* | Apolipoprotein D | ENSG00000189058 | -1.81 | 5.09E-03 |
| *GADD45B* | Growth arrest and DNA damage-inducible protein GADD45 beta | ENSG00000099860 | -1.8 | 6.47E-09 |
| *SCD* | Stearoyl-CoA desaturase | ENSG00000099194 | -1.8 | 9.11E-05 |
| *GPR162* | Probable G-protein coupled receptor 162 | ENSG00000250510 | -1.8 | 5.11E-03 |
| *DUSP5* | Dual specificity protein phosphatase 5 | ENSG00000138166 | -1.79 | 9.05E-05 |
| *STC1* | Stanniocalcin-1 | ENSG00000159167 | -1.79 | 4.87E-03 |
| *SNCA* | Alpha-synuclein | ENSG00000145335 | -1.79 | 4.80E-03 |
| *ID1* | DNA-binding protein inhibitor ID-1 | ENSG00000125968 | -1.76 | 3.01E-03 |
| *HSF4* | Heat shock factor protein 4 | ENSG00000102878 | -1.75 | 5.03E-03 |
| *CCL2* | C-C motif chemokine 2 | ENSG00000108691 | -1.74 | 2.13E-06 |
| *FMO4* | Dimethylaniline monooxygenase [N-oxide-forming] 4 | ENSG00000076258 | -1.72 | 0.02 |
| *EGR1* | Early growth response protein 1 | ENSG00000120738 | -1.67 | 3.03E-04 |
| *RSPO3* | R-spondin-3 | ENSG00000146374 | -1.65 | 6.38E-05 |
| *EPHB2* | Ephrin type-B receptor 2 | ENSG00000133216 | -1.64 | 0.01 |
| *EVI2B* | Protein EVI2B | ENSG00000185862 | -1.62 | 0.02 |
| *CRABP2* | Cellular retinoic acid-binding protein 2 | ENSG00000143320 | -1.62 | 1.73E-07 |
| *TRABD2A* | Metalloprotease TIKI1 | ENSG00000186854 | -1.61 | 3.38E-03 |
| *CCN4* | CCN family member 4 | ENSG00000104415 | -1.61 | 0.04 |
| *DNM1* | Dynamin-1 | ENSG00000106976 | -1.6 | 5.71E-04 |
| *EDNRA* | Endothelin-1 receptor | ENSG00000151617 | -1.58 | 6.90E-04 |
| *NEFL* | Neurofilament light polypeptide | ENSG00000277586 | -1.53 | 3.92E-03 |
| *ID4* | DNA-binding protein inhibitor ID-4 | ENSG00000172201 | -1.52 | 0.01 |
| *DBP* | D site-binding protein | ENSG00000105516 | -1.5 | 0.05 |
| *PTHLH* | Parathyroid hormone-related protein | ENSG00000087494 | -1.5 | 0.03 |
| *FOSB* | Protein fosB | ENSG00000125740 | -1.48 | 1.45E-06 |
| *MAML3* | Mastermind-like protein 3 | ENSG00000196782 | -1.47 | 0.03 |
| *ID3* | DNA-binding protein inhibitor ID-3 | ENSG00000117318 | -1.47 | 0.04 |
| *TGFB3* | Transforming growth factor beta-3 proprotein | ENSG00000119699 | -1.46 | 0.02 |
| *MILR1* | Allergin-1 | ENSG00000271605 | -1.45 | 0.02 |
| *CCN1* | CCN family member 1 | ENSG00000142871 | -1.45 | 1.48E-03 |
| *PDGFRL* | Platelet-derived growth factor receptor-like protein | ENSG00000104213 | -1.44 | 0.03 |
| *HR* | Lysine-specific demethylase hairless | ENSG00000168453 | -1.44 | 0.02 |
| *LIF* | Leukemia inhibitory factor | ENSG00000128342 | -1.44 | 0.01 |
| *RGS2* | Regulator of G-protein signaling 2 | ENSG00000116741 | -1.44 | 3.41E-03 |
| *C1QTNF6* | Complement C1q tumor necrosis factor-related protein 6 | ENSG00000133466 | -1.43 | 7.61E-03 |
| *SCN1B* | Sodium channel subunit beta-1 | ENSG00000105711 | -1.43 | 5.59E-03 |
| *SSC5D* | Soluble scavenger receptor cysteine-rich domain-containing protein SSC5D | ENSG00000179954 | -1.42 | 2.57E-03 |
| *BMP4* | Bone morphogenetic protein 4 | ENSG00000125378 | -1.42 | 3.41E-03 |
| *LIN7A* | Protein lin-7 homolog A | ENSG00000111052 | -1.42 | 0.04 |
| *OLFML1* | Olfactomedin-like protein 1 | ENSG00000183801 | -1.4 | 8.29E-03 |
| *TMTC2* | Protein O-mannosyl-transferase TMTC2 | ENSG00000179104 | -1.39 | 7.73E-03 |
| *NR1H3* | Oxysterols receptor LXR-alpha | ENSG00000025434 | -1.36 | 0.01 |
| *SOBP* | Sine oculis-binding protein homolog | ENSG00000112320 | -1.35 | 0.03 |
| *GPC6* | Glypican-6 | ENSG00000183098 | -1.33 | 0.01 |
| *ACP5* | Tartrate-resistant acid phosphatase type 5 | ENSG00000102575 | -1.33 | 0.04 |
| *PRRT2* | Proline-rich transmembrane protein 2 | ENSG00000167371 | -1.32 | 0.03 |
| *PER1* | Period circadian protein homolog 1 | ENSG00000179094 | -1.32 | 0.04 |
| *FADS1* | Acyl-CoA (8-3)-desaturase | ENSG00000149485 | -1.31 | 1.57E-04 |
| *SLC27A3* | Solute carrier family 27 member 3 | ENSG00000143554 | -1.31 | 6.17E-03 |
| *MFAP2* | Microfibrillar-associated protein 2 | ENSG00000117122 | -1.3 | 1.71E-04 |
| *TRIB3* | Tribbles homolog 3 | ENSG00000101255 | -1.3 | 0.02 |
| *BHLHE40* | Class E basic helix-loop-helix protein 40 | ENSG00000134107 | -1.29 | 3.92E-03 |
| *INSIG1* | Insulin-induced gene 1 protein | ENSG00000186480 | -1.27 | 3.41E-03 |
| *HSD17B14* | 17-beta-hydroxysteroid dehydrogenase 14 | ENSG00000087076 | -1.25 | 0.01 |
| *NFKBIZ* | NF-kappa-B inhibitor zeta | ENSG00000144802 | -1.25 | 3.20E-03 |
| *NUAK2* | NUAK family SNF1-like kinase 2 | ENSG00000163545 | -1.24 | 0.04 |
| *SLC16A4* | Monocarboxylate transporter 5 | ENSG00000168679 | -1.23 | 9.72E-03 |
| *KLF2* | Krueppel-like factor 2 | ENSG00000127528 | -1.22 | 0.02 |
| *MFSD9* | Major facilitator superfamily domain-containing protein 9 | ENSG00000135953 | -1.21 | 0.02 |
| *NR1D1* | Nuclear receptor subfamily 1 group D member 1 | ENSG00000126368 | -1.2 | 8.56E-03 |
| *SEMA3B* | Semaphorin-3B | ENSG00000012171 | -1.2 | 0.02 |
| *SREBF1* | Sterol regulatory element-binding protein 1 | ENSG00000072310 | -1.19 | 2.34E-03 |
| *FOS* | Proto-oncogene c-Fos | ENSG00000170345 | -1.18 | 0.04 |
| *IER2* | Immediate early response gene 2 protein | ENSG00000160888 | -1.16 | 1.34E-03 |
| *PLAU* | Urokinase-type plasminogen activator | ENSG00000122861 | -1.16 | 5.99E-03 |
| *RASD1* | Dexamethasone-induced Ras-related protein 1 | ENSG00000108551 | -1.15 | 0.03 |
| *FAM131B* | Protein FAM131B | ENSG00000159784 | -1.14 | 0.03 |
| *GDF15* | Growth/differentiation factor 15 | ENSG00000130513 | -1.12 | 0.03 |
| *C1QTNF1* | Complement C1q tumor necrosis factor-related protein 1 | ENSG00000173918 | -1.11 | 9.37E-03 |
| *DHCR7* | 7-dehydrocholesterol reductase | ENSG00000172893 | -1.11 | 4.41E-04 |
| *ACKR4* | Atypical chemokine receptor 4 | ENSG00000129048 | -1.1 | 6.66E-04 |
| *DUSP6* | Dual specificity protein phosphatase 6 | ENSG00000139318 | -1.09 | 0.02 |
| *OAF* | Out At First Homolog | ENSG00000184232 | -1.08 | 0.03 |
| *JUNB* | Transcription factor jun-B | ENSG00000171223 | -1.07 | 0.01 |
| *ENPP1* | Ectonucleotide pyrophosphatase/phosphodiesterase family member 1 | ENSG00000197594 | -1.07 | 6.17E-03 |
| *RGS4* | Regulator of G-protein signaling 4 | ENSG00000117152 | -1.05 | 0.01 |
| *PAMR1* | Inactive serine protease PAMR1 | ENSG00000149090 | -1.05 | 5.66E-03 |
| *HCFC1R1* | Host cell factor C1 regulator 1 | ENSG00000103145 | -1.04 | 0.01 |
| *MYC* | Myc proto-oncogene protein | ENSG00000136997 | -1.04 | 6.17E-03 |
| *KLF10* | Krueppel-like factor 10 | ENSG00000155090 | -1.03 | 0.02 |
| *GRAMD4* | GRAM domain-containing protein 4 | ENSG00000075240 | -1.03 | 0.04 |
| *DUSP1* | Dual specificity protein phosphatase 1 | ENSG00000120129 | -1.02 | 0.05 |
